# Stratification of radiosensitive brain metastases based on an actionable S100A9/RAGE resistance mechanism

**DOI:** 10.1038/s41591-022-01749-8

**Published:** 2022-04-11

**Authors:** Cátia Monteiro, Lauritz Miarka, María Perea-García, Neibla Priego, Pedro García-Gómez, Laura Álvaro-Espinosa, Ana de Pablos-Aragoneses, Natalia Yebra, Diana Retana, Patricia Baena, Coral Fustero-Torre, Osvaldo Graña-Castro, Kevin Troulé, Eduardo Caleiras, Patricia Tezanos, Pablo Muela, Elisa Cintado, José Luis Trejo, Juan Manuel Sepúlveda, Pedro González-León, Luis Jiménez-Roldán, Luis Miguel Moreno, Olga Esteban, Ángel Pérez-Núñez, Aurelio Hernández-Lain, José Mazarico Gallego, Irene Ferrer, Rocío Suárez, Eva M. Garrido-Martín, Luis Paz-Ares, Celine Dalmasso, Elizabeth Cohen-Jonathan Moyal, Aurore Siegfried, Aisling Hegarty, Stephen Keelan, Damir Varešlija, Leonie S. Young, Malte Mohme, Yvonne Goy, Harriet Wikman, Jose Fernández-Alén, Guillermo Blasco, Lucía Alcázar, Clara Cabañuz, Sergei I. Grivennikov, Andrada Ianus, Noam Shemesh, Claudia C. Faria, Rebecca Lee, Paul Lorigan, Emilie Le Rhun, Michael Weller, Riccardo Soffietti, Luca Bertero, Umberto Ricardi, Joaquim Bosch-Barrera, Elia Sais, Eduard Teixidor, Alejandro Hernández-Martínez, Alfonso Calvo, Javier Aristu, Santiago M. Martin, Alvaro Gonzalez, Omer Adler, Neta Erez, RENACER, Cecilia Sobrino, Nuria Ajenjo, Maria-Jesus Artiga, Eva Ortega-Paino, Manuel Valiente

**Affiliations:** 1 Brain Metastasis Group, CNIO, Madrid, Spain; 2 Bioinformatics Unit, CNIO, Madrid, Spain; 3 Histopathology Core Unit, CNIO, Madrid, Spain; 4 Department of Translational Neuroscience, Cajal Institute, CSIC, Madrid, Spain; 5 Neuro-Oncology Unit, Hospital Universitario 12 de Octubre, Madrid, Spain; 6 Neurosurgery Unit, Hospital Universitario 12 de Octubre, Madrid, Spain; 7 Department of Surgery, Universidad Complutense de Madrid, Madrid, Spain; 8 Neuropathology Unit, Hospital Universitario 12 de Octubre, Madrid, Spain; 9 Medical Oncology, Hospital Universitario 12 de Octubre, Madrid, Spain; 10 CNIO-H12O Clinical Cancer Research Unit, Fundación de Investigación Biomédica i+12 and CNIO, Madrid, Spain; 11 CIBERONC, https://www.ciberonc.es; 12 Department of Medicine, Universidad Complutense de Madrid, Madrid, Spain; 13 Radiation Oncology Department, Institut Claudius Regaud, IUCT-Oncopole, Toulouse, France; 14 Anatomopathology Department, CHU Toulouse, IUCT-Oncopole, Toulouse, France; 15 Endocrine Oncology Research Group, RCSI University of Medicine and Health Sciences, Dublin, Ireland; 16 Department of Neurosurgery, University Medical Center Hamburg-Eppendorf, Hamburg, Germany; 17 Department of Radiation Oncology, University Medical Center Hamburg-Eppendorf, Hamburg, Germany; 18 Department of Tumor Biology, University Medical Center Hamburg-Eppendorf, Hamburg, Germany; 19 Department of Neurosurgery, Hospital Universitario de La Princesa, Madrid, Spain; 20 Department of Pathology, Hospital Universitario La Paz, Madrid, Spain; 21 Cancer Prevention and Control Program, Fox Chase Cancer Center, Philadelphia, PA USA; 22 Champalimaud Research, Champalimaud Centre for the Unknown, Lisbon, Portugal; 23 Instituto de Medicina Molecular João Lobo Antunes, Facultade de Medicina, Universidade de Lisboa, Lisboa, Portugal; 24 Department of Neurosurgery, Hospital de Santa Maria, Centro Hospitalar Universitário Lisboa Norte (CHULN), Lisboa, Portugal; 25 Faculty of Biology Medicine and Health, The University of Manchester, Manchester, UK; 26 The Christie NHS Foundation Trust, Manchester, UK; 27 Department of Neurology, University Hospital Zurich, Zurich, Switzerland; 28 Division of Neuro-Oncology, Department of Neuroscience Rita Levi Montalcini, University of Turin, Turin, Italy; 29 Department of Medical Sciences, University of Turin, Turin, Italy; 30 Department of Oncology, University of Turin, Turin, Italy; 31 Department of Medical Oncology, Catalan Institute of Oncology, Doctor Josep Trueta University Hospital, Girona, Spain; 32 Girona Biomedical Research Institute (IDIBGi), Salt, Spain; 33 Department of Medical Sciences, University of Girona, Girona, Spain; 34 IDISNA, Program in Solid Tumors, Center for Applied Medical Research (CIMA), University of Navarra, Pamplona, Spain; 35 Department of Oncology, University Clinic of Navarra, Madrid, Spain; 36 Department of Oncology, University Clinic of Navarra, Pamplona, Spain; 37 Department of Biochemistry, University Clinic of Navarra, Pamplona, Spain; 38 Department of Pathology, Sackler Faculty of Medicine, Tel Aviv University, Tel Aviv, Israel; 39 Present Address: Departments of Medicine and Biomedical Sciences, Cedars-Sinai Cancer, Cedars-Sinai Medical Center, Los Angeles, CA USA; 40 Biobank, CNIO, Madrid, Spain

**Keywords:** CNS cancer, Metastasis

## Abstract

Whole-brain radiotherapy (WBRT) is the treatment backbone for many patients with brain metastasis; however, its efficacy in preventing disease progression and the associated toxicity have questioned the clinical impact of this approach and emphasized the need for alternative treatments. Given the limited therapeutic options available for these patients and the poor understanding of the molecular mechanisms underlying the resistance of metastatic lesions to WBRT, we sought to uncover actionable targets and biomarkers that could help to refine patient selection. Through an unbiased analysis of experimental in vivo models of brain metastasis resistant to WBRT, we identified activation of the S100A9–RAGE–NF-κB–JunB pathway in brain metastases as a potential mediator of resistance in this organ. Targeting this pathway genetically or pharmacologically was sufficient to revert the WBRT resistance and increase therapeutic benefits in vivo at lower doses of radiation. In patients with primary melanoma, lung or breast adenocarcinoma developing brain metastasis, endogenous S100A9 levels in brain lesions correlated with clinical response to WBRT and underscored the potential of S100A9 levels in the blood as a noninvasive biomarker. Collectively, we provide a molecular framework to personalize WBRT and improve its efficacy through combination with a radiosensitizer that balances therapeutic benefit and toxicity.

## Abstract (web summary)

A comprehensive analysis of models of brain metastasis and multiple cohorts of patient samples identifies a targetable molecular mechanism underlying the resistance to whole-brain radiotherapy that can inform patient selection for personalized radiotherapy.

## Main

The brain is a common metastatic location, and 20–40% of patients with solid tumors eventually develop lesions in the central nervous system, mostly spreading from lung and breast cancer or melanoma^1^. The development of effective therapeutic agents has remained a challenge, and most patients succumb to the disease less than 12 months after diagnosis^2–4^. Upon diagnosis, current clinical management of brain metastasis frequently includes radiotherapy, owing to its superior ability to access the brain and treat both local and distant intracranial lesions^5^.

Historically, whole-brain radiation therapy (WBRT) was the gold standard in the management of patients with brain metastasis. Although WBRT may control symptoms and decrease the incidence of intracranial failure as well as local recurrences, clinical trials did not demonstrate an effect on overall survival (OS) or quality of life when compared with supportive care^6–9^. Adding to this controversial role of WBRT, patients receiving radiotherapy usually show a high incidence of neurocognitive decline due to irradiation of healthy brain tissue^10–12^. Owing to the concerns associated with WBRT, clinical practice adopted stereotactic radiosurgery (SRS), a strategy to deliver a high dose of radiation specifically to metastatic lesions, as new standard of care for numerous indications in the management of brain metastasis^10,12,13^. However, WBRT continues to be an important treatment option. Patients may present with multifocal disease with lesions that are too large in size for SRS^14–16^. In addition, the use of SRS is associated with a higher incidence of radionecrosis than WBRT^17–19^. Thus, the limited efficacy of WBRT and the frequent recurrence of metastatic lesions within the irradiated field suggest the emergence of a profound resistance to irradiation.

Limited research has been devoted to the study of radiotherapy in preclinical brain metastasis models. In a triple-negative breast cancer (TNBC) and a lung cancer brain metastasis model a single dose of irradiation delivered to established metastases confirmed the clinically observed resistance to WBRT^20,21^. However, these and other studies^22,23^ do not faithfully recapitulate the clinical scenario, in which WBRT is not delivered in a single dose of radiation but as a hypofractionated protocol^7^. Previous works reported increased survival in mice with brain metastasis treated with a fractionated chemoradiotherapy protocol, but the phenotype was not reproduced with radiotherapy only^24^. A similar finding was made in established brain lesions with an experimental model of breast cancer brain metastasis using clinically adapted WBRT protocol of 3 Gy per day for 10 days^25^. None of these studies provided any molecular explanation for the emergence of radioresistance.

To address this clinically relevant problem, we characterized the resistance to radiation using mouse models, brain metastasis organotypic cultures established from patients who are radioresistant and several clinically annotated cohorts of patients with melanoma, lung or breast cancer with brain metastasis.

Here, we report that brain metastatic cancer cells from different primary tumors are induced to highly express S100A9 within the brain microenvironment, which mediates resistance to radiotherapy by downstream activation of NF-κB. Genetic or pharmacological targeting of S100A9 by the blood–brain barrier–permeable inhibitor FPS-ZM1 of its receptor, RAGE (receptor for advanced glycation end products), potently sensitizes brain metastasis to irradiation in experimental models of brain metastasis as well as in patient-derived organotypic cultures. Furthermore, S100A9 expression in human brain metastasis from patients with lung cancer, breast cancer or melanoma correlates with benefit from radiotherapy. Taken together, our findings suggest the use of S100A9 as a clinically relevant biomarker to personalize radiotherapy for patients with brain metastasis and the inhibitor FPS-ZM1 as a radiosensitizing agent.

## Results

### Experimental brain metastases do not respond to WBRT

**Fig. 1:**
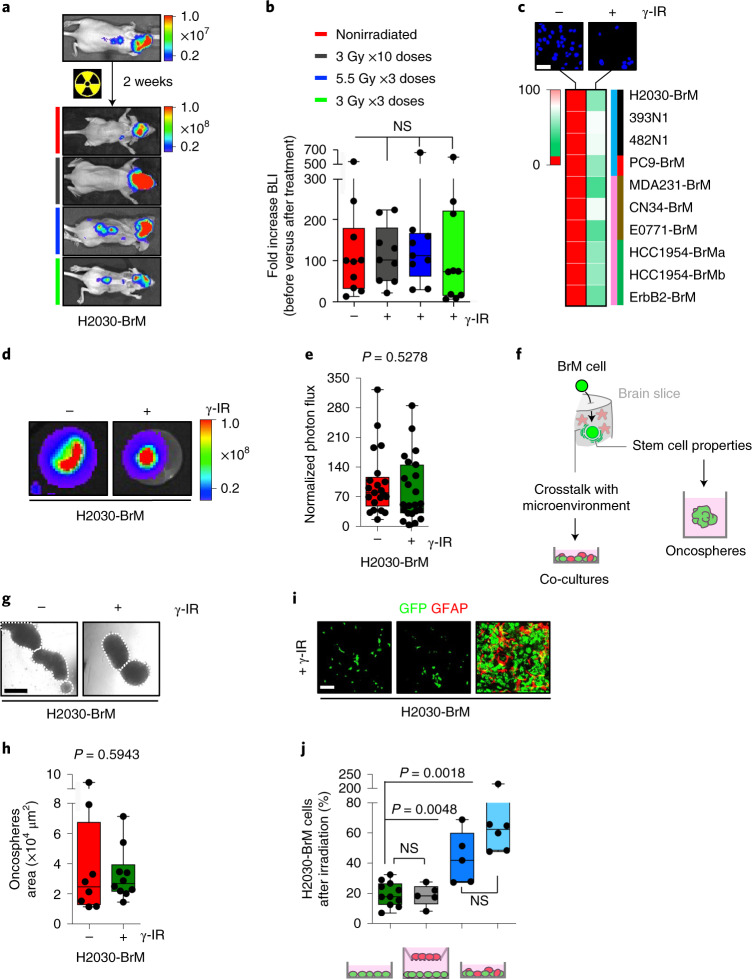
Acquired radioresistance in experimental brain metastasis. **a**, Representative BLI of mice injected IC with H2030-BrM before and after completing different radiation schedules. The colored lines indicate the specific condition/irradiation protocol as specified in **b**. Color bars show BLI intensity in p s^−1^ cm^−^^2^ sr^−1^). **b**, Quantification of BLI in the head of mice. Values correspond to the fold increase for each mouse before, week 2 after IC injection of H2030-BrM and after completing each radiation schedule week 4 after IC injection. Values are shown in box-and-whisker plots, where each dot is a mouse and the line in the box corresponds to the median. The boxes go from the upper to the lower quartiles, and the whiskers go from the minimum to the maximum value (*n* = 10, mice nonirradiated; *n* = 9, mice irradiated with 10 × 3 Gy; *n* = 9, mice irradiated with 3 × 5.5 Gy; *n* = 10, mice irradiated with 3 × 3 Gy). *P* value was calculated using two-tailed *t*-test between nonirradiated and irradiated experimental groups (nonirradiated versus 10 × 3 Gy, *P* = 0.7065; nonirradiated versus 3 × 5.5 Gy, *P* = 0.7109; nonirradiated versus 3 × 3 Gy, *P* = 0.9556). γ-IR, gamma irradiation; NS, not significant. **c**, Representative images of the cell density (blue, bisbenzimide) 72 h after irradiating H2030-BrM cells compared with nonirradiated control. Heatmap showing the sensitivity of ten different brain tropic cancer cell lines to irradiation 72 h after a single dose of 10 Gy. Heatmap colors correspond to the percentage of cells remaining after irradiation normalized to the nonirradiated control. Values were obtained from three replicates per experimental condition (mean percentage of viable cells after irradiation ± s.e.m.; *P* value was calculated using a two-tailed *t*-test (1, H2030-BrM: 27.63 ± 1.25, *P* = 0.0046; 2, 393N1: 48.77 ± 12.34, *P* = 0.0200; 3, 482N1: 47.25 ± 2.57, *P* = 0.0154; 4, PC9-BrM: 28.66 ± 6.81, *P* = 0.0013; 5, MDA231-BrM: 14.12 ± 3.35, *P* = 0.0010; 6, CN34-BrM: 47.46 ± 1.98, *P* = 0.0068; 7, E0771-BrM: 27.63 ± 1.25, *P* = 0.0046; 8, HCC1954-BrMa: 28.28 ± 3.87, *P* = 0.0023; 9, HCC1954-BrMb: 29.78 ± 1.25, *P* = 0.0064; 10, ErbB2-BrM: 34.38 ± 4.78, *P* = 0.0155)). Colored bars: blue, lung cancer-derived brain tropic models (1, H2030-BrM and 4, PC9-BrM: human cell lines; 2, 393N1 and 3, 482N1: mouse cell lines); pink, breast cancer-derived brain tropic models (5, MDA231-BrM, 6, CN34-BrM, 8, HCC1954-BrMa and 9, HCC1954-BrMb: human cell lines; 7, E0771-BrM and 10, ErbB2-BrM: mouse cell lines); black, *KRAS/Kras* and *TRP53/Trp53* mutants; red, *EGFR* mutant; brown, TNBC; green, HER2^+^ breast cancer. Scale bar, 5 μm. **d**, Representative images of brain organotypic cultures with metastatic cells 72 h after irradiation at 10 Gy. Color bar shows BLI intensity in p s^−1^ cm^−2^ sr^−1^). **e**, Quantification of photon flux values from metastatic cells growing in organotypic brain cultures after irradiation normalized to preirradiated BLI values. Values are shown in box-and-whisker plots where every dot represents an independent culture and the line in the box corresponds to the median. The boxes go from the upper to the lower quartiles, and the whiskers go from the minimum to the maximum value (*n* = 23, nonirradiated brain slices with H2030-BrM; *n* = 24, irradiated brain slices with H2030-BrM). *P* value was calculated using a two-tailed *t*-test. **f**, Working model suggesting potential sources of radioresistance and how to model them in vitro. **g**, Representative images of oncospheres 72 h after irradiation at 10 Gy. Scale bar, 250 µm. **h**, Quantification of oncosphere area. Values are shown in box-and-whisker plots, where every dot represents a different well from which the mean area of all oncospheres were quantified, from an independent culture and the line in the box corresponds to the median. The boxes go from the upper to the lower quartiles, and the whiskers go from the minimum to the maximum value (*n* = 8, nonirradiated wells with H2030-BrM oncospheres; *n* = 10, irradiated wells with H2030-BrM oncospheres). *P* value was calculated using a two-tailed *t*-test. **i**, Representative images of co-cultures between H2030-BrM (GFP^+^) and glial cells (astrocytes, GFAP^+^) 72 h after irradiation (10 Gy). Scale bar, 250 μm. **j**, Quantification of GFP^+^ BrM cells after radiation normalized to their respective nonirradiated controls from the experiment shown in **i**. Values are shown in box-and-whisker plots, where every dot represents an independent culture and the line in the box corresponds to the median. The boxes go from the upper to the lower quartiles, and the whiskers go from the minimum to the maximum value (*n* = 11, H2030-BrM; *n* = 5 H2030-BrM in indirect co-culture with glia cells; *n* = 5, H2030-BrM in direct co-culture with glia cells; *n* = 6, H2030-BrM in direct co-culture with astrocytes). *P* values were calculated using a two-tailed *t*-test.

**Extended Data Fig. 1:**
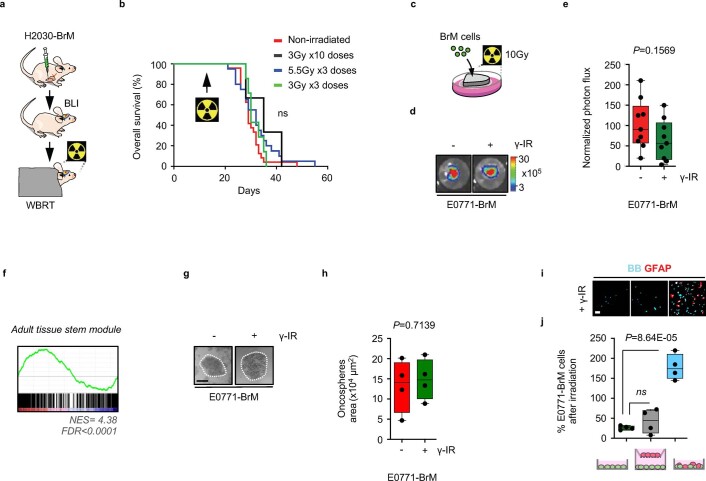
Acquired radioresistance in experimental brain metastasis. **a**, Schema of the experimental design. BLI: bioluminescence imaging. **b**, Kaplan–Meier curves showing survival in nonirradiated and irradiated experimental groups described in Fig. 1b (*n* = 10, mice nonirradiated; *n* = 9, mice irradiated with 10 x 3 Gy; *n* = 9, mice irradiated with 3 × 5.5 Gy; *n* = 10, mice irradiated with 3 x 3 Gy). *P* value was calculated using log-rank (Mantel–Cox) test between nonirradiated and irradiated experimental groups (nonirradiated versus 10 x 3 Gy, *P* = 0.2019; nonirradiated versus 3 × 5.5 Gy, *P* = 0.1649; nonirradiated versus 3 x 3 Gy, *P* = 0.3057). **c**, Schema of experimental design. **d**, Representative images of brain organotypic cultures with metastatic cells 72 hours after irradiation at 10 Gy. **e**, Quantification of photon flux values from metastatic cells growing in organotypic brain cultures after irradiation normalized to preirradiated BLI values. Values are shown in box-and-whisker plots where every dot represents an independent culture and the line in the box corresponds to the median. The boxes go from the upper to the lower quartiles and the whiskers go from the minimum to the maximum value (n = 9, nonirradiated brain slices with E0771-BrM; *n* = 9, irradiated brain slices with E0771-BrM). *P* value is calculated using two-tailed *t* test. **f**, Representative graph of positively enriched gene sets depicting induction of a stem cell signature among a preranked list of differentially expressed genes from resistant culture preparations ex vivo (Supplementary Table 1). The green curve corresponds to the Enrichment Score, while the normalized enrichment score (NES) and the FDR are shown below the graph. **g**, Representative images of oncospheres 72 hours after irradiation at 10 Gy. Scale bar, 250 µm. **h**, Quantification of oncosphere area. Values are shown in box-and-whisker plots where every dot represents a different well from which the mean area of all oncospheres were quantified, from an independent culture and the line in the box corresponds to the median. The boxes go from the upper to the lower quartiles and the whiskers go from the minimum to the maximum value (*n* = 4, nonirradiated wells with E0771-BrM oncospheres; *n* = 4, irradiated wells with E0771-BrM oncospheres). *P* value is calculated using two-tailed t test. **i**, Representative images of co-cultures between E0771-BrM and astrocytes (GFAP + ) 72 h after irradiation (10 Gy). Scale bar, 100 μm **j**, Quantification of GFAP- BrM cells after irradiation normalized to their respective nonirradiated controls from experiment in **i**. Values are shown in box-and-whisker plots where every dot represents an independent culture and the line in the box corresponds to the median. The boxes go from the upper to the lower quartiles and the whiskers go from the minimum to the maximum value (*n* = 4, in each respective experimental condition). *P* value is calculated using two-tailed t test.

Three different protocols of hypofractionated WBRT^24–26^ were applied to established brain metastases after intracardiac (IC) inoculation of the lung adenocarcinoma cell line H2030-BrM (ref. ^27^) (Extended Data Fig. 1a and Fig. 1a). None of them halted the progression of the disease (Fig. 1b), and mice died at the same time as nonirradiated controls (Extended Data Fig. 1b). Histological analysis of irradiated brain metastasis confirmed maintenance of the proliferative rate in contrast to the significant decrease observed when radiation was applied to extracranial tumors (Supplementary Fig. 1a–c). To confirm this finding, we irradiated established E0771-BrM brain metastases, a organotropic syngeneic model (Supplementary Fig. 1e and f) derived from a TNBC cell line^28^. In this case, we had to perform intracranial injection of the cancer cells providing sufficient time to complete the WBRT schedule (Supplementary Fig. 1g). This is necessary because IC injection of E0771-BrM leads to very extensive extracranial disease (Supplementary Fig. 1h) that develops faster than intracranial metastasis. Similarly, irradiation was not effective to control the progression of E0771-BrM in the brain (Supplementary Fig. 1i,j) in contrast to extracranial metastases (Supplementary Fig. 1k,l). Given the radioresistance of brain metastasis in vivo, we evaluated the response to irradiation in vitro. Irradiation of H2030-BrM, E0771-BrM and eight additional BrM cell lines in vitro (Fig. 1c and Supplementary Fig. 1d) reproduced the high radiosensitivity of BrM cells outside the brain.

### A transcriptional signature correlates with radioresistance

We asked whether the mere contact with the brain tissue might unlock the activation of radioresistance mechanisms. H2030-BrM or E0771-BrM cells plated on live brain slices cultured ex vivo^29^ (Extended Data Fig. 1c) and treated with the same irradiation protocol that reduced their viability under standard culture conditions in vitro (Fig. 1c) did not reproduce a therapeutic benefit (Fig. 1d,e and Extended Data Fig. 1d,e). BrM cells plated on brain slices adapt to this culture by interacting with the surrounding microenvironment^29,30^ (Fig. 1f) that, by the reanalysis of published data^31^, involved transcriptional changes compatible with the acquisition of stem cell-like properties (Extended Data Fig. 1f and Supplementary Table 1), which might be required to reinitiate the tumor at secondary organs^32,33^. Consequently, we hypothesized that in vitro protocols enriching tumor-initiating properties and co-culturing BrM cells with components of the microenvironment might also induce radioresistance (Fig. 1f).

Oncospheres, which are thought to have increased cancer stem cell properties and thus metastatic ability^34^, from H2030-BrM and E0771-BrM cells treated with the same irradiation dose tested under standard culture conditions in vitro (Fig. 1c) showed radioresistance (Fig. 1g,h and Extended Data Fig. 1g,h).

The ability of metastatic cells to interact with glial cells seems to be critical to colonize the brain^35^ and can involve both physical contact^36,37^ and paracrine interactions^38,39^. Interestingly, co-culture between BrM cells and glial cells^40^, particularly astrocytes, increased resistance to radiation only when the culture included direct heterotypic cell–cell contacts (Fig. 1i,j and Extended Data Fig. 1i,j).

**Fig. 2:**
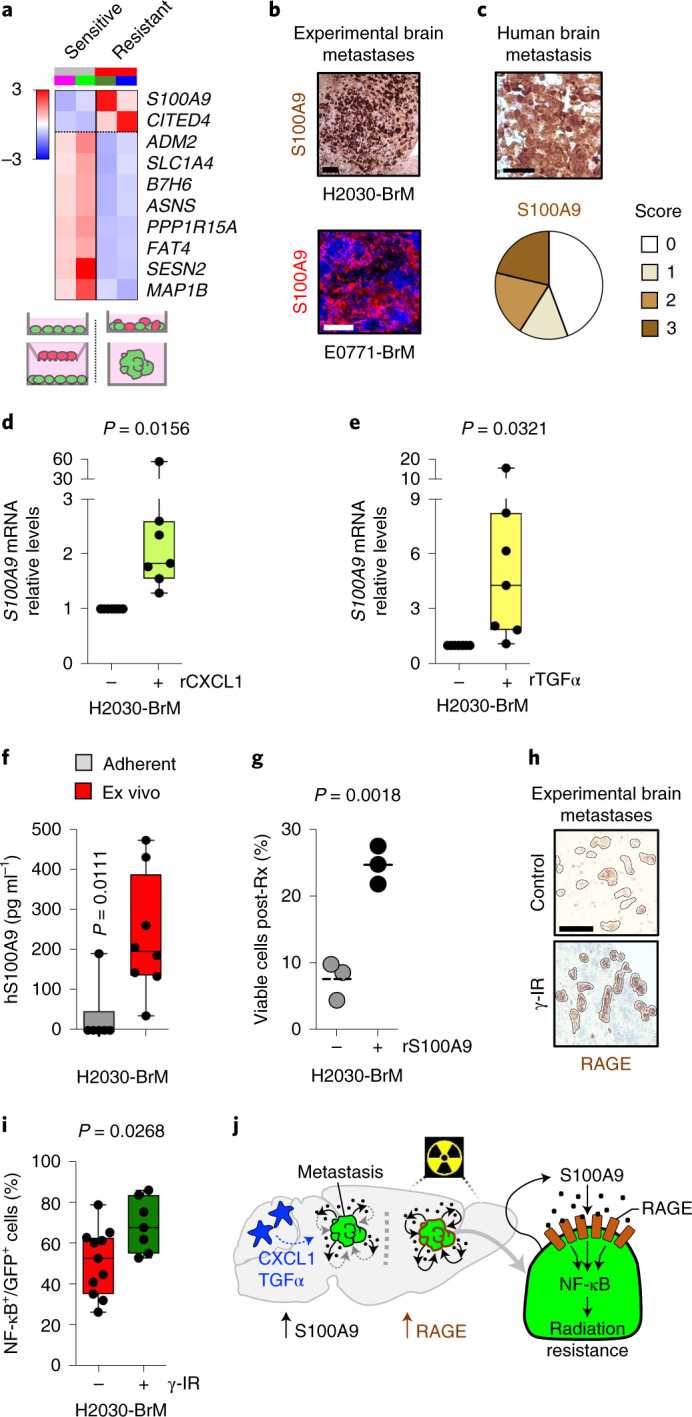
Contact-dependent astrocyte-released cytokines induce S100A9 secretion in cancer cells, triggering NF-κB activation. **a**, Heatmap representing color-coded expression levels of commonly deregulated genes in H2030-BrM cell line when cultured under radiosensitive (purple line, adherent; light green line, co-culture with inserts) compared with radioresistant (dark green line, oncospheres; blue line, cell–cell co-culture) conditions in vitro. Only genes with false discovery rate (FDR) < 0.05 and a log_2_ ratio >1 were considered. **b**, Representative images of S100A9 protein expression levels in metastatic lesions growing in brains from mice IC injected with H2030-BrM and E0771-BrM. Three brains were analyzed in each BrM model. Scale bars, 120 µm (H2030-BrM) and 50 µm (E0771-BrM). **c**, Sixty-six human brain metastases from patients with lung cancer (33 cases) or breast cancer (30 cases) or other primary tumors (3 cases) were stained for S100A9 by immunohistochemistry. Representative images are shown. Scale bar, 50 µm. Quantification of different histological scoring of cancer cells is shown in pie charts. Five cases had to be excluded; 27 of 61 were scored with no staining (score 0), 9 of 61 with weak staining (score 1), 12 of 61 with moderate staining (score 2) and 13 of 61 with strong staining (score 3). **d**, Quantification of *S100A9* expression levels in H2030-BrM after stimulation with 100 ng ml^−1^ recombinant CXCL1 (rCXCL1) or control. Values are shown in dot plots, and dots represent independent experiments. The line in the box corresponds to the median. The boxes go from the upper to the lower quartiles, and the whiskers go from the minimum to the maximum value (*n* = 7, each experimental condition). *P* value was calculated Wilcoxon signed rank test, two sided. **e**, Quantification of *S100A9* expression levels in H2030-BrM after stimulation with recombinant transforming growth factor α (rTGF-α) or control. Values are shown in dot plots and dots represent independent experiments. The line in the box corresponds to the median. The boxes go from the upper to the lower quartiles, and the whiskers go from the minimum to the maximum value (*n* = 7, each experimental condition). *P* value was calculated using two-tailed *t*-test. **f**, Quantification by enzyme-linked immunosorbent assay (ELISA) of human S100A9 (hS100A9) in the supernatant of H2030-BrM grown either under adherent conditions in vitro or in organotypic cultures ex vivo. Values are shown in box-and-whisker plots, where every dot represents an independent experiment and the line in the box corresponds to the median. The boxes go from the upper to the lower quartiles, and the whiskers go from the minimum to the maximum value (*n* = 6, H2030-BrM adherent cultures; *n* = 8, H2030-BrM growing in organotypic brain cultures). *P* value was calculated using two-tailed Mann–Whitney test. **g**, Quantification of in vitro viable cell fraction after irradiation at 10 Gy and 200 ng ml^−1^ recombinant hS100A9 or control, as determined by manual cell counting of bisbenzimide-positive nuclei. Values are percentages of viable cells respect to unirradiated controls and shown in a dot plot, where each dot represents an independent experiment and the line in the box corresponds to the median (*n* = 3, each experimental condition). *P* value was calculated using a two-tailed *t*-test. **h**, Representative pictures of RAGE immunohistochemistry from unirradiated or irradiated established H2030-BrM metastases in vivo. Scale bar, 50 µm. **i**, Quantification of the percentage of NF-κB^+^ GFP^+^ H2030-BrM cells identified by the expression of an engineered mCherry NF-κB activity reporter. Brain slices with cancer cells were evaluated 72 h after treatment with radiotherapy. Values are shown in box-and-whisker plots where each dot is a brain organotypic culture (*n* = 11, nonirradiated; *n* = 7, irradiated with a single dose of 10 Gy) and the line in the box corresponds to the median. The boxes go from the upper to the lower quartiles and the whiskers go from the minimum to the maximum value. *P* value was calculated using a two-tailed *t*-test. **j**, Schema of working model of radioresistance in brain metastasis.

**Extended Data Fig. 2:**
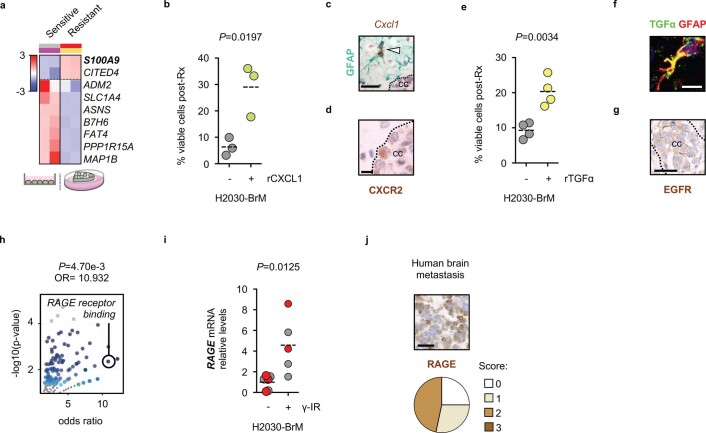
Contact-dependent astrocyte-released cytokines induce S100A9 secretion in cancer cells triggering NF-κB activation. **a**, Heatmap depicting the gene signature in Fig. 2a scored in the comparison between H2030-BrM growing in adherent conditions *in vitro* and in brain organotypic cultures *ex vivo*. Only genes with FDR < 0.05 and a log_2_ ratio >1 were considered. **b**, Quantification of *in vitro* viable cell fraction after irradiation at 10 Gy and stimulation with 100 ng/ml rCXCL1 or control, as determined by manual cell counting of bisbenzimide+ nuclei. Values are percentages of viable cells respect to unirradiated controls and shown in a dot plot where each dot represents an independent experiment and the line in the box corresponds to the median and whiskers go from the minimum to the maximum value (n = 3, each experimental condition). *P* value was calculated using two-tailed t test. **c**, Representative image of the peritumoral microenvironment where CXCL1 mRNA is labelled by RNA *in situ* hybridization (brown) and GFAP by immunohistochemistry (purple). The dotted line surrounds the metastasis. cc: cancer cells. Scale bar: 50 µm. The experiment was independently repeated three times with similar results. **d**, Representative image of CXCR2 protein in H2030-BrM brain metastasis. The dotted line surrounds the metastasis. cc: cancer cells. Scale bar: 25 µm. The experiment was independently repeated three times with similar results. **e**, Quantification of *in vitro* viable cell fraction after irradiation at 10 Gy and stimulation with rTGFα or control, as determined by manual cell counting of bisbenzimide+ nuclei. Values are percentages of viable cells respect to unirradiated controls and shown in a dot plot where each dot represents an independent experiment and the line in the box corresponds to the median median and whiskers go from the minimum to the maximum value (n = 4, each experimental condition). *P* value was calculated using two-tailed t test. **f**, Representative image of the peritumoral microenvironment where TGFα co-localizes with astrocytes (GFAP + ). Scale bar: 10 µm. The experiment was independently repeated three times with similar results. **g**, Representative image of EGFR immunohistochemistry in experimental H2030-BrM brain metastasis. Scale bar: 50 µm. The experiment was independently repeated three times with similar results. **h**, Differentially expressed genes in resistant culture preparations (2 biological independent replicas used for each radioresistant preparation (oncospheres, n = 2; co-cultures (cell–cell contact), n = 2), and for radiosensitive preparations, (adherent cultures, n = 2; co-cultures (insert), n = 2)) were used to perform Gene Ontology (GO) analysis using the EnrichR software. The volcano plot represents the significance of each gene set from GO Molecular Function versus its odds ratio. *P* value is computed from the Fisher exact test. Larger blue points represent significant terms (*P* value<0.05); smaller grey points represent non-significant terms. The darker the blue color of a point, the more significant it is. **i**, Quantification of *RAGE* expression levels in H2030-BrM 24 hours after a single dose (10 Gy) of radiation. Both adherent (grey) and oncospheres (red) are plotted together. Each circle represents independent cultures (n = 3 nonirradiated adherent cells, n = 3 nonirradiated oncospheres, n = 3 nonirradiated adherent cells, n = 2 nonirradiated oncospheres). Expression values were normalized to their respective nonirradiated control for each culture condition and the line represents the mean. *P* value was calculated using two-tailed t test. Nonirradiated adherent cells versus irradiated adherent cells, *P* = 0.1521; nonirradiated oncospheres versus irradiated oncospheres, *P* = 0.0522. **j**, 66 human brain metastases from lung cancer patients (33 cases), breast cancer patients (30 cases) or patients with other primary tumors (3 cases) were stained for RAGE by immunohistochemistry. Representative images are shown. Scale bar: 50 µm. Quantification of different histological scoring of cancer cells is shown in pie charts. 6 cases had to be excluded, 15/60 were scored with no staining, 17/60 with weak staining and 28/60 with moderate staining.

Based on these findings, we performed RNA sequencing (RNA-seq) on all sensitive and resistant in vitro conditions 24 h after applying radiation, when cell viability was not compromised (Supplementary Fig. 2a,b). Although transcriptomic differences were evident between the two radioresistant culture conditions (oncospheres and co-cultures) (Supplementary Fig. 2c and Supplementary Table 2), we also identified a discrete commonly deregulated gene signature (Fig. 2a, Supplementary Fig. 2d,e and Supplementary Table 3). Interestingly, this signature was also present in cancer cells plated on brain organotypic cultures (Supplementary Table 4)^31^, denoting *S100A9* as the top upregulated gene (Extended Data Fig. 2a and Supplementary Table 5). We further validated the induction of S100A9 in cancer cells ex vivo (Supplementary Fig. 2f,g) and in vivo in experimental (Fig. 2b) and human (Fig. 2c) brain metastases.

Thus, our data suggest that S100A9 expression is induced in cancer cells under specific contexts correlating with the acquisition of radioresistance.

### Astrocyte-released cytokines induce S100A9 secretion in cancer cells

Our in vitro and ex vivo radioresistance paradigms might involve different mechanisms mediating the induction of S100A9 in cancer cells (that is, acquisition of stem cell properties versus the influence of the microenvironment) (Fig. 1f). When we explored changes of murine cytokines in the brain microenvironment affected by metastases, we identified CXCL1, CCL2 and CCL4 as the only ones that were significantly upregulated compared with the normal brain (Supplementary Fig. 2i). Among these, only CXCL1 secretion was also increased in astrocytes by direct co-culture with cancer cells (Supplementary Fig. 2j). Additionally, our previous research identified tumor-necrosis factor α (TNF-α), interferon-α and TGF-α as the top upregulated cytokines in astrocytes upon direct co-culture with brain metastatic cells^36^. Because interferon-α and TGF-α were not included in our unbiased approach (Supplementary Fig. 2i), we interrogated whether any of them induced *S100A9* expression in cancer cells and concluded that only TGF-α did so (Fig. 2e and Supplementary Fig. 2k). Similarly, recombinant CXCL1 was confirmed to induce *S100A9* expression in brain metastatic cells (Fig. 2d). Addition of each individual cytokine was sufficient to increase the radioresistance of H2030-BrM cells in vitro (Extended Data Fig. 2b,e). Finally, we validated the presence of CXCL1 and TGF-α in metastasis-associated astrocytes (Extended Data Fig. 2c,f) and the corresponding receptors, CXCR2 and EGFR, respectively, in metastatic cells in situ (Extended Data Fig. 2d,g). Overall, our data suggest that the induction of S100A9 in brain metastasis might be the consequence of an inflammatory response initiated by the direct contact between reactive astrocytes and cancer cells (Fig. 2j).

S100A9 is frequently secreted into the extracellular space and binds to Toll-like receptor 4 or RAGE^41^. We first hypothesized that the presence of S100A9 in the extracellular space might be involved in the acquisition of radioresistance because the protein generated by cancer cells could only be detected in the conditioned media (CM) of organotypic brain cultures, but not in the one obtained from adherent cultures in vitro of the same BrM cell line (Fig. 2f). In addition, when recombinant S100A9 was added to radiosensitive BrM cells growing under adherent conditions in vitro, a threefold induction of resistance to radiotherapy was detected (Fig. 2g). The transcriptomic profile of radioresistant preparations suggested that RAGE activity was induced upon radiation (Extended Data Fig. 2h,i), which we validated in situ in experimental (Fig. 2h) and human brain metastases (Extended Data Fig. 2j). Binding of S100A9 to RAGE has been shown to activate numerous signaling cascades, including NF-κB^42,43^. Although this signaling pathway was previously reported to be involved in radioresistance of glioblastoma and other contexts^44–46^, it has not been linked to S100A9 or therapeutic resistance in brain metastasis. Remarkably, the transcriptomic profile of radioresistant preparations showed enriched NF-κB signaling pathways (Supplementary Fig. 2h and Supplementary Table 6) that was confirmed in metastatic cells receiving irradiation in brain organotypic cultures (Fig. 2i).

Thus, our data suggest that metastatic cells in the brain induce the expression and secretion of S100A9, which, through radiation-induced RAGE receptor expression, might activate NF-κB-mediated radioresistance (Fig. 2j). We subsequently decided to functionally validate this working model.

### Targeting S100A9 radiosensitizes experimental brain metastases

**Fig. 3:**
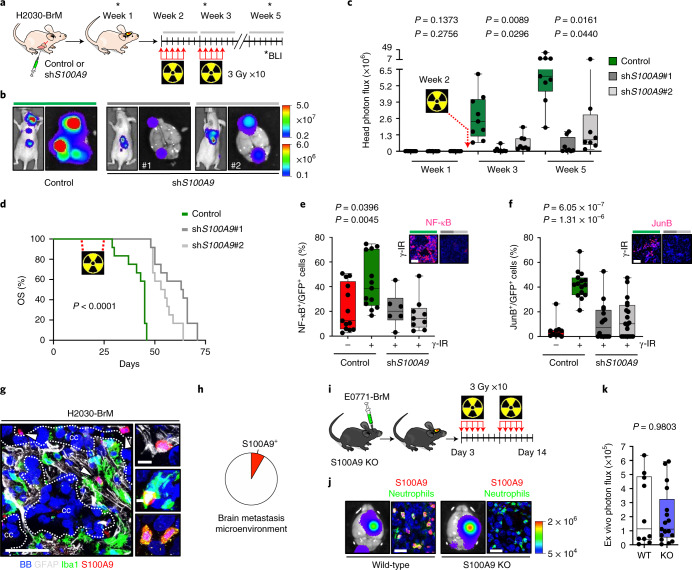
Targeting S100A9 in cancer cells radiosensitizes experimental lung and breast cancer brain metastases in a NF-κB–JunB-dependent manner. **a**, Schema of experimental design. **b**, Representative BLI of mice 5 weeks after being inoculated IC with H2030-BrM control (left), sh*S100A9*#1 (middle) or sh*S100A9*#2 (right) cells and treated with 10 × 3 Gy irradiation using the WBRT protocol depicted in **a**. Ex vivo brain BLI is also shown for each condition. Color bars show BLI intensity in p s^−1^ cm^−2^ sr^−1^. BLI colour bars correspond to in vivo (top) and ex vivo (bottom). **c**, Quantification of in vivo photon flux values from the head of mice inoculated with H2030-BrM control, sh*S100A9*#1 or sh*S100A9*#2 cells that received WBRT, as depicted in **a**. BLI was performed at three different time points during the course of treatment (weeks 1, 3 and 5). Values are shown in box-and-whisker plots, where every dot represents a different brain and the line in the box corresponds to the median. The boxes go from the upper to the lower quartiles, and the whiskers go from the minimum to the maximum value (*n* = 9, H2030-BrM control; *n* = 8, H2030-BrM sh*S100A9*#1; *n* = 8, H2030-BrM sh*S100A9*#2). *P* value was calculated using two-tailed *t*-test. **d**, Kaplan–Meier plot showing survival proportions of mice inoculated with H2030-BrM control, sh*S100A9*#1 or sh*S100A9*#2 cells that received WBRT as depicted in **a** (*n* = 12, each experimental condition). *P* value was calculated using a log-rank (Mantel–Cox) test. OS, overall survival. **e**, Quantification of the percentage of NF-κB^+^ GFP^+^ positive H2030-BrM control, shS*100A9*#1 or sh*S100A9*#2 cells in organotypic cultures, which received no or 10 Gy irradiation. Values are shown in box-and-whisker plots, where every dot represents an independent culture and the line in the box corresponds to the median. The boxes go from the upper to the lower quartiles and the whiskers go from the minimum to the maximum value (*n* = 13, H2030-BrM control; *n* = 13, H2030-BrM control + γ-IR; *n* = 6, sh*S100A9*#1 + γ-IR; *n* = 9, sh*S100A9*#2 + γ-IR). *P* values were calculated using two-tailed *t*-tests. Upper panel shows representative organotypic cultures from the experiment in the panel. Scale bar, 75 µm. **f**, Quantification of the percentage of JunB^+^ GFP^+^ BrM cells in nonirradiated H2030-BrM control and irradiated H2030-BrM control, H2030-BrM shS*100A9*#1 or H2030-BrM sh*S100A9*#2 brain metastatic lesions. Values are shown in box-and-whisker plots, where every dot represents a metastatic lesion and the line in the box corresponds to the median. The boxes go from the upper to the lower quartiles, and the whiskers go from the minimum to the maximum value (*n* = 10 metastases from 2 animals, H2030-BrM control; *n* = 15 metastases from 2 animals, H2030-BrM control + γ-IR; *n* = 16 metastases from 3 animals, H2030-BrM sh*S100A9*#1 + γ-IR; *n* = 19 metastases from 3 animals, H2030-BrM sh*S100A9*#2 + γ-IR). *P* values were calculated using two-tailed Mann–Whitney tests. Upper panel shows representative organotypic cultures from the experiment in the panel. Scale bar, 75 µm. **g**, Representative images of an established H2030-BrM brain metastasis and its microenvironment. The S100A9 antibody used for this staining is rodent specific to stain only the microenvironment and not human cancer cells. Arrowheads point to S100A9^+^ cells in the microenvironment. CC, cancer cells. The image is representative of the five independent brains evaluated. Scale bars, 50 µm (left panel) and 10 µm (right panels). **h**, Quantification of S100A9^+^ BB^+^ cells in the microenvironment of brain metastasis. Only GFP^−^ cells were quantified to exclude cancer cells. Pie chart represents S100A9^+^ GFP^−^ BB^+^ cells as the red slice (8.29%) and S100A9^−^ GFP^−^ BB^+^ cells as the white slice. Cells were quantified in nine fields of view (FOVs) representing equally different sizes of metastasis from three mice brains. **i**, Schema of experimental design. **j**, Representative bioluminescence and immunofluorescent images of ex vivo brains from S100A9^+/+^ or S100A9^−/−^ mice 2 weeks after intracranial injection of E0771-BrM cells. After inoculation, mice were consequently treated with WBRT as shown in **i**. Neutrophils are labeled with NIMP-R14 antibody (green). The bioluminescence images are representative of the 15 brains analyzed, and the immunofluorescence images are representative of the six brains analyzed. Color bar shows BLI intensity in p s^−1^ cm^−2^ sr^−1^). Scale bar, 25 µm. **k**, Quantification of ex vivo brain photon flux values from S100A9^+/+^ or S100A9^−/−^ mice inoculated with E0771-BrM cells and treated with WBRT as depicted in **i**. Values are shown in box-and-whisker plots, where every dot represents a different brain and the line in the box corresponds to the median. The boxes go from the upper to the lower quartiles, and the whiskers go from the minimum to the maximum value (*n* = 10, S100A9^+/+^ mice; *n* = 16, S100A9^−/−^ mice). *P* value was calculated using Mann–Whitney test, two sided. KO, knockout; WT, wild-type.

**Extended Data Fig. 3:**
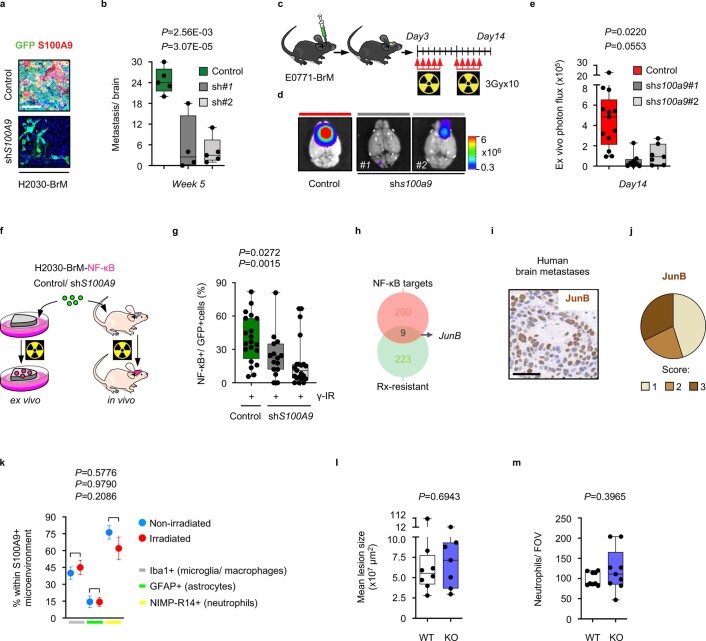
Targeting S100A9 in cancer cells radiosensitizes experimental lung and breast cancer brain metastases in a NF-κB–JunB-dependent manner. **a**, Representative images of histology of brain metastatic lesions from experiment in Fig. 3a-c. GFP: cancer cells. Scale bar: 100 µm. **b**, Quantification of the number of brain metastatic lesions in mice inoculated with H2030-BrM control, shS*100A9#1* or sh*S100A9#2* cells and treated with WBRT. Metastatic burden was assessed at endpoint by histology. Values are shown in box-and-whisker plots where every dot represents a different brain and the line in the box corresponds to the median. The boxes go from the upper to the lower quartiles and the whiskers go from the minimum to the maximum value (n = 5, H2030-BrM Control; n = 4, H2030-BrM sh*S100A9*#1; n = 5, H2030-BrM sh*S100A9*#2). *P* value is calculated using two-tailed t test. **c**, Schema of experimental design. **d**, Representative bioluminescence images of *ex vivo* brains from mice two weeks after intracranial injection of E0771-BrM cells transduced with either a scrambled shRNA as Control, or one of two different shRNA against murine *S100a9* sh#1 or sh#2. After inoculation, mice were consequently treated with WBRT as shown in (**c**). **e**, Quantification of *ex vivo* brain photon flux values from irradiated mice inoculated with E0771-BrM Control, sh*S100a9*#1 or sh*S100a9*#2. Values are shown in box-and-whisker plots where every dot represents a different brain and the line in the box corresponds to the median. The boxes go from the upper to the lower quartiles and the whiskers go from the minimum to the maximum value (n = 13, E0771-BrM Control; n = 8, E0771-BrM sh*s100a9*#1; n = 7, E0771-BrM sh*s100a9*#2). *P* value is calculated using two-tailed t test. **f**, Schema of experimental design. H2030-BrM control or sh*S100A9* cells engineered with a NF-κB-mCherry reporter were used *ex vivo* in organotypic cultures or IC injected in mice. **g**, Quantification of the percentage of NF-κB + GFP + positive cells in irradiated brain metastatic lesions *in vivo*, generated from IC injection of H2030-BrM control, H2030-BrM shS*100A9#1* or H2030-BrM sh*S100A9#2* cells (n = 4 mice, each experimental condition). Values are shown in box-and-whisker plots where every dot represents a metastatic lesion and the line in the box corresponds to the median. The boxes go from the upper to the lower quartiles and the whiskers go from the minimum to the maximum value (n = 20 FOV, H2030-BrM Control + γ-IR; n = 13 FOV, H2030-BrM sh*S100A9*#1 + γ-IR; n = 21 FOV, H2030-BrM sh*S100A9*#2 + γ-IR). *P* value was calculated using two-tailed Mann–Whitney Test. **h**, Venn diagram showing the overlap of 9 genes (including *JunB*) between established NF-κB targets and genes significantly upregulated in both *in vitro* and *ex vivo* radioresistant culture preparations (Supplementary Table 12). **i**, Representative image of JunB staining by immunohistochemistry in human brain metastases. Scale bar: 50 µm. This analysis was repeated 62 independent samples. **j**, 62 human brain metastases from lung cancer patients (33 cases), breast cancer patients (27 cases) or patients with other primary tumors (2 cases) were stained for JunB. Quantification of histological scoring of cancer cells is shown in a pie chart. 28/62 were scored with weak staining (score 1), 14/62 with moderate staining (score 2) and 20/62 with strong staining (score 3). **k**, Quantification of S100A9 + GFAP + , S100A9 + Iba1+ and S100A9 + NIMP-R14 + cells in the microenvironment of H2030-BrM brain metastasis by double-immunofluorescent staining of unirradiated and irradiated mice brains. Values shown are percentages of all microenvironmental S100A9 + cells. Dots correspond to the mean and error bars correspond to sem. 6-10 FOV per condition from 3 (nonirradiated) and 2 (irradiated) brains were quantified. *P* values were calculated using two-tailed t test. **l**, Quantification of brain metastatic lesion size by histology of brains obtained from experiment depicted in Fig. 3i. Values are shown in box-and-whisker plots where every dot represents a different brain and the line in the box corresponds to the median. Whiskers go from the minimum to the maximum value (n = 8, S100A9^+/+^ mice; n = 7, S100A9^-/-^ mice). *P* value was calculated using Mann–Whitney test, two sided. **m**, Quantification of neutrophils by histology of brains obtained from experiment depicted in Fig. 3i. Values are shown in box-and-whisker plots where every dot represents a different field of view and the line in the box corresponds to the median. Whiskers go from the minimum to the maximum value (n = 9 fields of view obtained from 3 S100A9^+/+^ and 3 S100A9^+/+^ mice). *P* value was calculated using Mann–Whitney test, two sided.

Cancer-related phenotypes ascribed to S100A9 include its involvement as an inducer of invasion^47,48^ that has been linked to the premetastatic niche^49,50^ as well as to chemoresistance driven by the recruitment of myeloid cells to lung metastases^51^ or in a cell-autonomous manner^52^. Knockdown of *S100A9* in H2030-BrM cells (Supplementary Fig. 3a) did not impair the growth rate of metastatic cells ex vivo in organotypic cultures (Supplementary Fig. 3b–d) or in vivo in brain metastasis (Supplementary Fig. 3e,f). Remarkably, *S100A9* genetic loss of function sensitized brain metastasis to radiation in organotypic culture conditions (Supplementary Fig. 3b–d). Encouraged by these results, we performed an in vivo experiment applying a protocol of WBRT mimicking the clinical approach (Fig. 1b) to our preclinical model once lung cancer brain metastasis with downregulated *S100A9* levels were established (Fig. 3a)^53^. This genetic loss of function reverted the acquired resistance of H2030-BrM brain metastases to radiotherapy in vivo (Fig. 3b,c), even to half dose of radiation (Fig. 3c). At the end of the experiment, histological analyses confirmed a major reduction in the number of metastases with barely detectable levels of S100A9 (Extended Data Fig. 3a,b). Given the notable reduction in brain metastasis burden, we wondered whether the genetic strategy could even provide a survival benefit in spite of the concomitant progressive disease extracranially (Fig. 3b). Remarkably, both knockdown targeting *S100A9* in H2030-BrM cancer cells were sufficient to extend OS when combined with radiotherapy (Fig. 3d).

Given that our previous data suggested that S100A9-dependent radioresistance could also apply to other experimental BrM models independently of primary origin (Extended Data Fig. 1d,e,g–j), we evaluated the role of S100A9 in E0771-BrM, a TNBC brain metastasis syngeneic model (Supplementary Fig. 1e–h) resistant to WBRT (Supplementary Fig. 1i,j). Similar to the lung adenocarcinoma model, *s100a9* loss of function (Supplementary Fig. 3g) did not generate any phenotype in vivo before treatment (Supplementary Fig. 3h,i). However, when the WBRT protocol was applied to established E0771-BrM intracranial tumors with reduced S100A9 levels (Extended Data Fig. 3c), a significant reduction of brain metastases was observed (Extended Data Fig. 3d,e).

In order to determine whether S100A9 was the main contributor to NF-κB activity under radioresistant conditions (Fig. 2i and Supplementary Fig. 2h), we evaluated a pathway-dependent fluorescent reporter for NF-κB activity (mCherry^+^)^54,55^ in the context of S100A9 loss of function (Extended Data Fig. 3f) in H2030-BrM cells expressing constitutively GFP. The increased percentage of double fluorescent mCherry^+^/GFP^+^ cancer cells after irradiation ex vivo was abrogated when S100A9 was targeted (Fig. 3e). Similarly, analysis of cancer cells with activated NF-κB signaling (mCherry^+^/ GFP^+^) after irradiation in vivo confirmed their dependency on S100A9 (Extended Data Fig. 3g). Furthermore, we identified nine NF-κB targets upregulated under radioresistant conditions (Extended Data Fig. 3h and Supplementary Table 12). Out of these, we confirmed S100A9-dependent JunB induction upon irradiation (Fig. 3f) and presence in human brain metastasis (Extended Data Fig. 3i,j).

S100A9 has also been reported in neutrophils and monocytes besides cancer cells^42,43^. Indeed, we detected S100A9 in a small fraction (8.3%) of the brain metastasis microenvironment (Fig. 3g,h), mainly represented by neutrophils but with measurable contributions from Iba1^+^ and GFAP^+^ cells, namely microglia/macrophages and reactive astrocytes, respectively (Fig. 3g and Extended Data Fig. 3k). Given that our previous loss-of-function approach (Fig. 3a, Extended Data Fig. 3c) did not consider the noncancer compartment and that S100A9^+^ cells in the microenvironment are represented by multiple cell types (Fig. 3g), we generated a genetically engineered mouse model with the *S100a9* gene knocked out (Supplementary Fig. 3m) to evaluate its potential contribution to the resistance phenotype. Interestingly, the E0771-BrM cell line, which colonized the brain of knockout mice indistinguishably from control mice (Supplementary Fig. 3j,k), remained resistant to WBRT when the microenvironment expressed no S100A9 (Fig. 3i–k and Extended Data Fig. 3l,m). Complementary, the significant reduction in brain metastases upon *S100A9* knockdown in cancer cells did not correlate with changes in the number of infiltrating neutrophils (Supplementary Fig. 3l). Thus, cancer-derived S100A9 in brain metastasis appears to be necessary and sufficient for radioresistance in experimental lung and breast cancer models.

### S100A9-mediated radioresistance is actionable and linked to cancer stem cell properties

**Extended Data Fig. 4:**
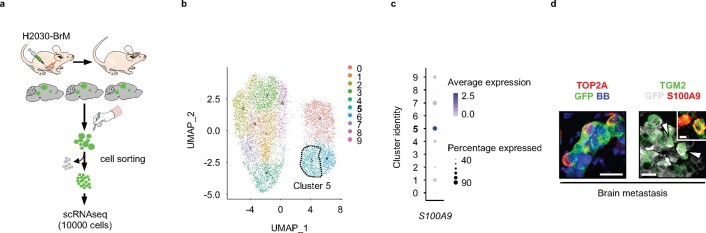
S100A9-mediated radioresistance is linked to cancer stem cell properties and sensitivity to RAGE and NF-κB inhibition. **a**, Schema of experimental design. **b**, UMAP plot of all cancer cells analyzed showing the existence of 9 clusters. Colors represent each cluster. Dotted line surrounds cluster 5. **c**, Expression of *S100A9* in each cluster. In a given cluster, size of the circle corresponds to the percentage of cells expressing the gene, while the shade of blue indicates the average expression of the gene. **d**, Representative images of immunofluorescent staining of selected candidates from single-cell analysis of Fig. 1a in H2030-BrM brain metastasis. Scale bars: 25 µm (low magnification) and 12.5 µm (high magnification). This experiment was repeated three times with similar results.

Although local aggressiveness of metastatic cells could increase their exposure to the microenvironment, we found S100A9 in an indolent metastasis model^56^ (Supplementary Fig. 4a) and, complementary, variably expressed at the invasive front of human brain metastases (Supplementary Fig. 4b). Thus, we explored the identity of S100A9^+^ cells in situ using single-cell RNA-seq (scRNA-seq)^57^ (Extended Data Fig. 4a).

**Fig. 4:**
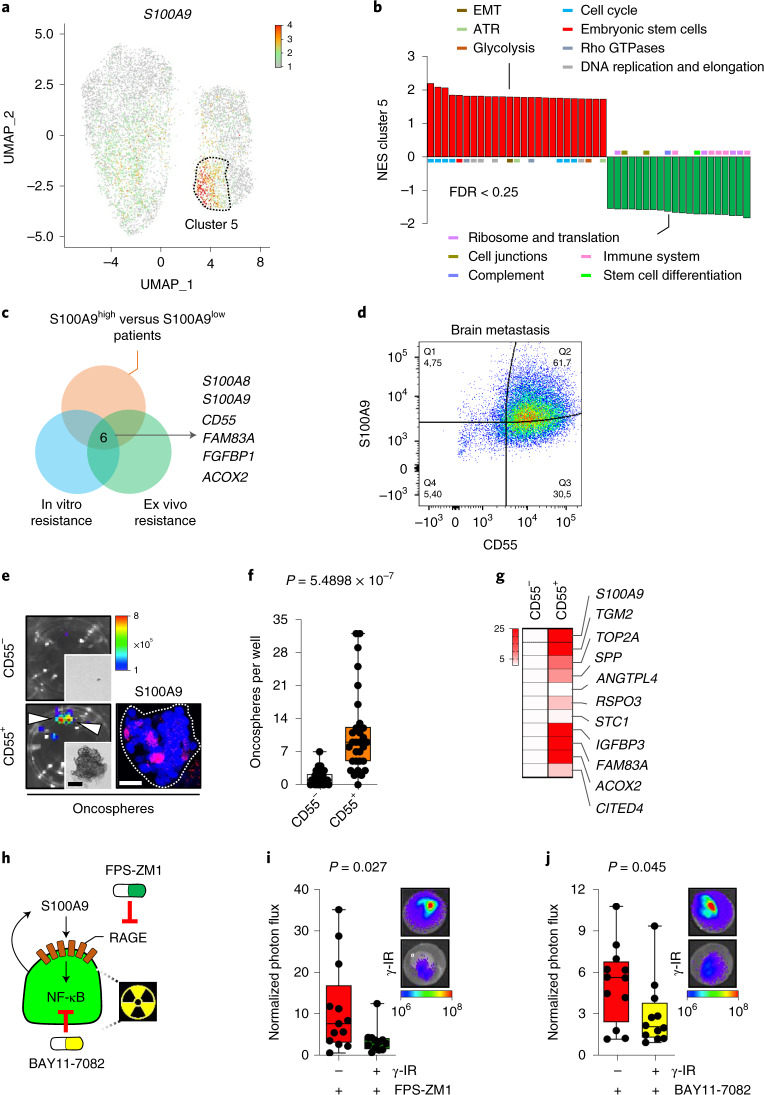
S100A9-mediated radioresistance is linked to cancer stem cell properties and sensitivity to RAGE and NF-κB inhibition. **a**, Uniform manifold approximation and projection (UMAP) plot of all analyzed cancer cells showing *S100A9* gene expression. Color corresponds to the gene expression in each cell. Dotted line surrounds cluster 5. **b**, GSEA of genes deregulated in cluster 5 versus all other clusters, corresponding to Supplementary Table 10. Displayed gene sets are the 25 highest ranking up- or downregulated gene sets according to the normalized enrichment score (NES) and a cutoff of *P* value < 0.05 and FDR < 0.25. Colored bars correspond to the biological category these gene sets belong to. EMT, epithelial–mesenchymal transition. ATR, ataxia aelangiectasia and Rad3-related protein. **c**, Venn diagram showing the strategy delineating CD55 by intersecting genes upregulated in S100A9^high^ patients with genes upregulated in in vitro and ex vivo radioresistant culture conditions (Supplementary Table 11). **d**, Representative flow cytometry dot plot illustrating the double staining CD55/S100A9 in permeabilized cancer cells sorted from H2030-BrM brain metastases. Q, quartile. **e**, Left: representative BLI images of oncospheres generated from CD55^−^ and CD55^+^ sorted cells from H2030-BrM brain metastases. Representative brightfield images of respective oncospheres are also shown in the smaller panels. Arrowheads point to oncospheres. Scale bar, 125 µm. Right: representative immunofluorescent image of S100A9 staining in CD55^+^ oncospheres. Color bar shows BLI intensity in p s^−1^ cm^−2^ sr^−1^). Scale bar, 25 µm. **f**, Quantification of numbers of oncospheres per well from CD55^−^ and CD55^+^ sorted cells. Values are shown in box-and-whisker plots, where every dot represents an individual well from three independent experiments and the line in the box corresponds to the median. The boxes go from the upper to the lower quartiles, and the whiskers go from the minimum to the maximum value (*n* = 30 wells, each experimental condition). *P* value was calculated using a two-tailed *t*-test. **g**, Heatmap displaying gene expression values of 11 genes, selected from highly upregulated genes of cluster 5 of the single-cell analysis in **a**, in CD55^+^ and CD55^−^ oncospheres. Color-coded values depicted are fold-changes normalized to CD55^−^ oncospheres (fold increase, CD55^+^/CD55^−^: *S100A9*, 36.91; *TGM2*, 50.96; *TOP2A*, 10.01; *SPP*, 7.52; *ANGPTL4*, 0.82; *RSPO3*, 4.63; *STC1*, 1.32; *IGFBP3*, 167.68; *FAM83A*, 148.97; *ACOX2*, 34.60; *CITED4*, 4.19). **h**, Schema illustrating pharmacological approaches to evaluate the working model of radioresistance. **i**, Representative images and quantification of photon flux values from H2030-BrM cells growing in organotypic brain cultures treated with 10 µM of FPS-ZM1 after irradiation. Values at endpoint (day 3) were first normalized to values from the same culture before treatment (day 0). Values are shown in box-and-whisker plots, where every dot represents an independent culture and the line in the box corresponds to the median. The boxes go from the upper to the lower quartiles, and the whiskers go from the minimum to the maximum value (*n* = 13, nonirradiated H2030-BrM; *n* = 12, irradiated H2030-BrM, both treated with FPS-ZM1). *P* value was calculated using a two-tailed *t*-test. **j**, Representative images and quantification of brain organotypic cultures with H2030-BrM cells 72 h after treatment with 50 µM BAY-117081, an inhibitor of IκB-α phosphorylation, and 10 Gy irradiation or no irradiation. Quantification of photon flux values at endpoint (day 3) were normalized to values from the same culture before treatment (day 0). Values are shown in box-and-whisker plots, where every dot represents an independent culture and the line in the box corresponds to the median. The boxes go from the upper to the lower quartiles, and the whiskers go from the minimum to the maximum value (*n* = 12, nonirradiated H2030-BrM; *n* = 12, irradiated H2030-BrM, both treated with BAY-117081). *P* value was calculated using a two-tailed *t*-test. Color bar in **i** and **j** shows BLI intensity in p s^−1^ cm^−2^ sr^−1^).

Metastatic cells were allocated into nine clusters (Extended Data Fig. 4b and Supplementary Table 7), with six of them showing detectable expression levels of *S100A9* (Extended Data Fig. 4c), in agreement with a more abundant expression in cancer cells than in the microenvironment (Fig. 3h). However, *S100A9*, together with *S100A8*, were the two genes with the most variable expression pattern among cancer cells (Supplementary Fig. 4d and Supplementary Table 8). Indeed, we validated the established^43^ functional dependency between them (Supplementary Fig. 4e,f). Interestingly, the highest values of *S100A9* expression correspond to cluster 5 (Fig. 4a and Supplementary Table 9), which was enriched in stem cell signatures among other features previously linked to radioresistance, such as epithelial-mesenchymal transition, glycolytic metabolism and DNA repair^58^ (Fig. 4b, Extended Data Fig. 4d and Supplementary Table 10). Hence, to test whether S100A9^+^ cells have cancer stem cell properties, we evaluated their ability to form oncospheres^34^. We first identified a cell surface marker from S100A9^+^ cancer cells. CD55 was selected as part of the overlap between differentially expressed genes in patients with high levels of *S100A9*, scored in a published cohort of breast cancer brain metastases^59^, and the in vitro and ex vivo radioresistant surrogates (Fig. 4c, Supplementary Fig. 2d and Supplementary Table 11). After confirming the correlation of CD55 and S100A9 in H2030-BrM brain metastases (Fig. 4d), sorted CD55^+^ and CD55^−^ cancer cells (Supplementary Fig. 4c) were subjected to oncosphere assays. Only the CD55^+^ fraction of cancer cells formed oncospheres (Fig. 4e,f), contained S100A9^+^ cells (Fig. 4e) independently of the culture condition (Supplementary Fig. 4g) and recapitulated the gene expression signature of cluster 5 (Fig. 4g and Supplementary Table 9).

In spite of the high therapeutic resistance presumably linked to S100A9 (Fig. 4b and Supplementary Table 10), we tested pharmacological inhibitors against various pathway components (Fig. 4h). We treated organotypic cultures containing brain metastatic cells with inhibitors targeting RAGE (FPS-ZM1) or NF-κB (BAY117082) in combination with radiotherapy (Fig. 4h). The results obtained demonstrate the ability of both inhibitors to revert acquired radioresistance (Fig. 4i,j and Supplementary Fig. 4h,i).

### S100A9 is a brain metastasis biomarker of therapeutic response to WBRT

**Fig. 5:**
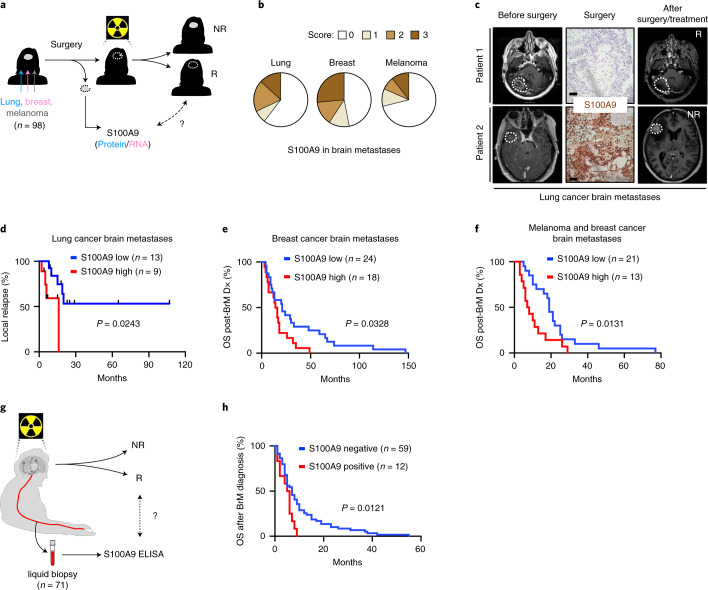
S100A9 is a brain metastasis biomarker of therapeutic response to WBRT. **a**, Schema of study design. S100A9 protein or mRNA levels, respectively, were assessed in 22 patients with lung cancer brain metastases, 62 patients with breast cancer brain metastases and 14 patients with melanoma brain metastases who underwent neurosurgery and were subsequently treated with radiotherapy. S100A9 was correlated with response to radiotherapy. R, responder; NR, nonresponder. **b**, A total of 140 human brain metastases from patients with lung cancer (53 cases), breast cancer (49 cases) or melanoma (38 cases) were stained for S100A9 by immunohistochemistry. Quantification of different histological scoring of cancer cells is shown in pie charts separate for each primary tumor. For lung cancer, 32 of 53 were scored with no staining (score 0), 4 of 53 with weak staining (score 1), 10 of 53 with moderate staining (score 2) and 7 of 53 with strong staining (score 3). For breast cancer, 23 of 49 were scored with no staining, 6 of 49 with weak staining, 7 of 49 with moderate staining and 13 of 49 with strong staining. For melanoma, 27 of 38 were scored with no staining, 4 of 38 with weak staining, 3 of 38 with moderate staining and 4 of 38 with strong staining. **c**, Representative MRI images of two lung cancer brain metastasis patients before and after neurosurgery and WBRT. For each patient the corresponding S100A9 immunohistochemical staining is shown. The images shown are representative of the 22 patients analyzed. Scale bar, 100 µm. **d**, Analysis of time to local relapse, as evaluated by follow-up MRI, after neurosurgery and WBRT in a cohort of 22 patients with lung cancer brain metastases. Data are shown as a Kaplan–Meier plot, and two groups of patients (S100A9 low/high) were delineated by using 5% of S100A9 immunohistochemical staining positivity as a cutoff. *P* value was calculated using a log-rank (Mantel–Cox) test. **e**, Analysis of survival after brain metastasis diagnosis in a cohort of 42 patients with breast cancer brain metastases. Only patients who received adjuvant radiotherapy were included. Data are shown as a Kaplan–Meier plot, and two groups of patients (S100A9 low/high) were delineated according to their *S100A9* mRNA expression levels in brain metastasis. *P* value was calculated using a log-rank (Mantel–Cox) test. **f**, Analysis of survival after brain metastasis diagnosis in a cohort of 34 patients with brain metastasis from breast cancer (20 cases) and melanoma (14 cases). Data are shown as a Kaplan–Meier plot, and two groups of patients (S100A9 low/high) were delineated by using 5% of S100A9 immunohistochemical staining positivity as a cutoff. *P* value was calculated using a log-rank (Mantel–Cox) test. **g**, Schema of study design. S100A9 protein was measured in liquid biopsy specimens from 71 patients with brain metastases who received WBRT. S100A9 positivity in serum was correlated with response to radiotherapy. **h**, Analysis of survival after brain metastasis diagnosis in a cohort of 71 patients with brain metastases from lung cancer (43 cases), breast cancer (14 cases), melanoma (10 cases) or other primary tumors (4 cases). Data are shown as a Kaplan–Meier plot, and two groups of patients (positive/negative) were delineated according to their S100A9 positivity in serum samples, collected before or within 2.5 months of receiving WBRT. *P* value was calculated using log-rank (Mantel–Cox) test. BrM, brain metastasis.

Given the correlation between S100A9 and radioresistance in brain metastasis models and the presence of the pathway in human samples (Fig. 2c, Extended Data Fig. 2j, Extended Data Fig. 3i and Supplementary Table 13), we explored a potential correlation with radioresistance in patients. First, we confirmed the presence of S100A9^+^ cancer cells independently of the primary source of the metastasis (Fig. 5b and Supplementary Table 14); of note, the percentage was lower in melanoma brain metastases than in lung or breast cancer (Fig. 5b). Interestingly, in contrast to experimental models of brain metastasis derived from lung cancer or breast cancer (Fig. 2b, Supplementary Fig. 2f,g, Extended Data Figs. 3a and 4d and Supplementary Fig. 4a), we were unable to find a melanoma brain metastasis model expressing S100A9 (Supplementary Fig. 5a), potentially suggesting that a specific subtype of melanoma brain metastases, not represented by available mouse models, could be the main contributor to S100A9 positivity. To score the clinical correlation with S100A9, several cohorts of patients with brain metastasis and lung cancer (*n* = 22), breast cancer^59,60^ (*n* = 42) or melanoma (*n* = 34) were selected based on the presence of patients who received neurosurgery followed by radiotherapy (Fig. 5a and Supplementary Tables 15–17). S100A9 levels in lung cancer brain metastases obtained by neurosurgery correlated with the time for brain relapse after WBRT, as measured by follow-up magnetic resonance imaging (MRI) (Fig. 5c,d and Supplementary Table 15). However, patients treated with SRS, a different modality of radiotherapy involving high-dose radiation targeted to small and highly localized areas, could not be correlated with S100A9 levels, because no relapses were detected (Supplementary Fig. 5b and Supplementary Table 18). Analysis of the cohort of patients with breast cancer brain metastases^59,60^ who were treated with radiotherapy confirmed the correlation between *S100A9* expression levels and survival from brain metastasis diagnosis (Fig. 5e and Supplementary Table 16), which was reproduced by *S100A8* (Supplementary Fig. 5c,d and Supplementary Table 16), as in experimental models (Supplementary Fig 4e,f). Of note, the clinical correlation was independent of *S100A9* levels in the primary tumor^59^ (Supplementary Fig. 5e,f). An additional cohort including patients with brain metastases from breast cancer and melanoma reproduced the clinical correlation between S100A9 and radiation response (Fig. 5f and Supplementary Table 17).

Furthermore, a similar correlation of *S100A9* with shorter survival was also detected in two independent cohorts of glioblastoma (Supplementary Fig. 5g,h and Supplementary Table 19), a primary brain tumor including focal brain radiotherapy as part of the standard of care^61^. In contrast, The Cancer Genome Atlas (TCGA) data including irradiated primary tumors from lung (Supplementary Fig. 5i) or breast cancer (Supplementary Fig. 5j) did not show a correlation between progression-free survival and *S100A9* levels (Supplementary Table 20).

Thus, S100A9 should be considered as a biomarker to predict therapeutic response of brain metastases to WBRT. In addition, our findings indicate that the same resistance mechanism might be extended to primary brain tumors, but not necessarily to extracranial ones.

Only a subpopulation of patients with secondary brain tumors are candidates for neurosurgery given the limitations imposed by the number and location of metastases as well as the presence of extracranial systemic disease and associated overall status of the patient^16^. Consequently, to expand the biomarker strategy to patients not eligible for neurosurgery and given the secretory nature of S100A9 (Fig. 2f), we tested the clinical correlation between S100A9 and response to radiotherapy in noninvasive liquid biopsy specimens. A total of 71 patients with brain metastases from any cancer type who received WBRT and had available blood samples as liquid biopsy specimens were analyzed (Fig. 5g and Supplementary Table 21). Strikingly, circulating S100A9 levels before or immediately after receiving WBRT correlated with survival from the diagnosis of brain metastasis (Fig. 5h and Supplementary Table 21). Of note, S100A9 levels in the blood did not show any clinical correlation with survival in those patients with brain metastases that did not receive WBRT (Supplementary Fig. 5k and Supplementary Table 21). No other variable such as age or Karnofsky Performance Status score correlate with S100A9 (Supplementary Fig 5l,m and Supplementary Tables 15–17, 21 and 22). However, to be conclusive about these additional variables, a larger prospective clinical study is required.

### FPS-ZM1 radiosensitizes experimental and human brain metastases

**Fig. 6:**
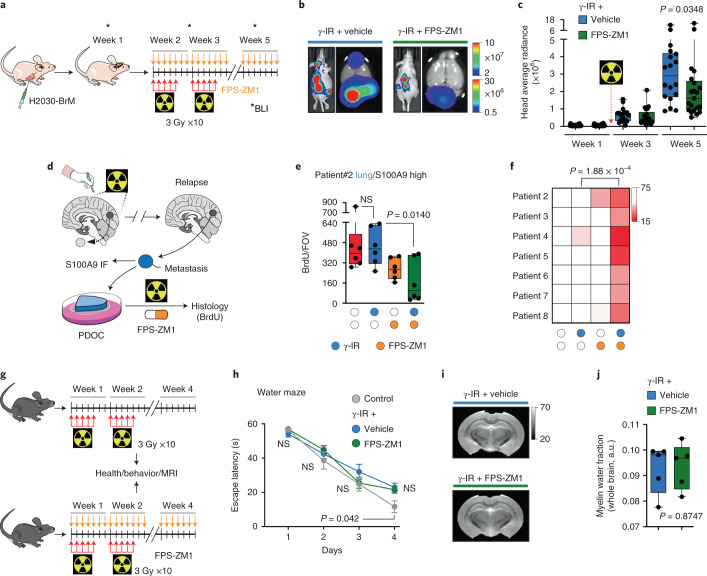
FPS-ZM1 radiosensitizes experimental and human brain metastases. **a**, Schema of experimental design. H2030-BrM cells were inoculated IC into nude mice, and 2 weeks later, mice received 10 doses of 3 Gy WBRT plus 500 mg kg^−1^ per day FPS-ZM1 or vehicle until the end of the experiment. Intracranial tumor growth was monitored weekly with BLI. **b**, Representative bioluminescence images in vivo and ex vivo (brains) of control and experimental arms at endpoint, 5 weeks after IC injection. BLI scale bars correspond to in vivo (top) and ex vivo (bottom). Colors bar show BLI intensity in p s^−1^ cm^−2^ sr^−1^). **c**, Quantification of in vivo photon flux values from the head of mice inoculated with H2030-BrM cells that received WBRT plus vehicle or FPS-ZM1, as depicted in **a**. BLI was performed at three different time points during the course of treatment (weeks 1, 3 and 5). Values are shown in box-and-whisker plots, where every dot represents a different brain and the line in the box corresponds to the median. The boxes go from the upper to the lower quartiles, and the whiskers go from the minimum to the maximum value (*n* = 18, vehicle + γ-IR; n = 21, FPS-ZM1 + γ-IR). *P* value was calculated using two-tailed Mann–Whitney test. **d**, Schema of experimental design. Surgically resected human brain metastases from patients who relapsed after receiving previous local treatments, including WBRT, were used to measure S100A9 levels by immunofluorescence (IF) and establish PDOCs, which were treated with FPS-ZM1 (10 µM) with or without irradiation and the therapeutic benefit evaluated 3 days later by 5-bromodeoxyuridine (BrdU) incorporation in cancer cells. **e**, Quantification of BrdU^+^ cancer cells in PDOCs from patient 2 treated with FPS-ZM1 and/or irradiation. Values are shown in box-and-whisker plots, where every dot represents an independent culture and the line in the box corresponds to the median. The boxes go from the upper to the lower quartiles, and the whiskers go from the minimum to the maximum value (*n* = 6, DMSO; *n* = 6, FPS-ZM1, *n* = 6, γ-IR; *n* = 6, FPS-ZM1 + γ-IR). *P* values were calculated using a two-tailed *t*-test. **f**, Heatmap depicting the quantification of BrdU^+^ cancer cells in PDOCs derived from seven patients with relapsed brain metastases, which have S100A9 high levels. Each row represents an individual patient, and each column represents different treatment conditions. Color-coded values are percentages of BrdU^+^ cancer cells normalized to the DMSO condition of each patient. *P* value was calculated using a two-tailed *t*-test. **g**, Schema of experimental design. Tumor-naive C57BL/6 mice received 10 doses of 3 Gy WBRT plus 500 mg kg^−1^ per day FPS-ZM1 or vehicle for 3 weeks. One month later, mice underwent health assessment, imaging and behavioral testing. **h**, Quantification of the time spent to escape the water to the platform (escape latency) from different groups of mice. Values are shown in a temporal scale, where each dot represents the mean value for each group in a given day of the test and the error bars represent s.e.m. (*n* = 6, control; *n* = 13, vehicle + γ-IR, *n* = 20, FPS-ZM1 + γ-IR). Calculation of *P* values is detailed in Supplementary Table 24. **i**, Representative ex vivo ultrahigh-field MRI images of brains from mice treated with either WBRT + vehicle or WBRT + FPS-ZM1 are shown. Greyscale shows the values from the long T2 component by ex vivo ultrahigh-field MRI. **j**, Quantification of whole-brain myelin water fraction by ex vivo ultrahigh-field MRI in brains from experiment depicted in **i**. Values are shown in box-and-whisker plots, where every dot represents a brain and the line in the box corresponds to the median. The boxes go from the upper to the lower quartiles, and the whiskers go from the minimum to the maximum value (*n* = 5, each experimental condition). *P* value was calculated using a two-tailed *t*-test.

**Extended Data Fig. 5:**
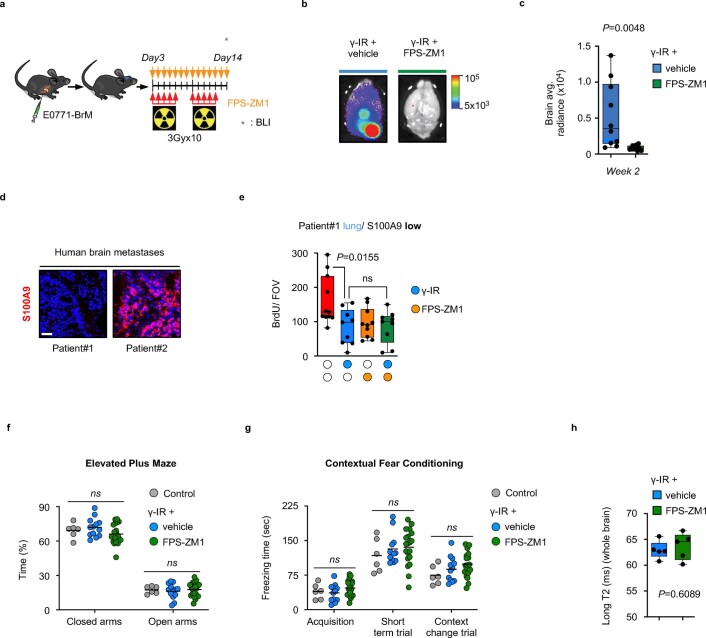
FPS-ZM1 radiosensitizes experimental and human brain metastases. **a**, Schema of experimental design. E0771-BrM were inoculated IC into C57BL/6 mice and three days later mice received 10 doses of 3 Gy WBRT plus 500 mg/Kg/day FPS-ZM1 or vehicle until the end of the experiment. Intracranial tumor growth was measured *ex vivo* at the end of the experiment. **b**, Representative bioluminescence images of *ex vivo* brains of control and experimental arms at endpoint, 2 weeks after IC injection. **c**, Quantification of *ex vivo* photon flux values from brains from mice inoculated with E0771-BrM that received WBRT plus vehicle or FPS-ZM1, as depicted in (**a**). Values are shown in box-and-whisker plots where every dot represents a different brain and the line in the box corresponds to the median. The boxes go from the upper to the lower quartiles and the whiskers go from the minimum to the maximum value (n = 10, Vehicle + γ-IR; n = 11, FPS-ZM1 + γ-IR). *P* value was calculated using two-tailed t test. **d**, Representative S100A9 immunofluorescence from two human brain metastasis that were processed as PDOCs. Patient#1 corresponds to a previously untreated brain metastasis that was negative for S100A9. Patient#2 corresponds to a relapsed metastasis. Scale bar: 50 µm. **e**, Quantification of BrdU+ cancer cells in PDOC from patient#1 treated with FPS-ZM1 and/or γ-IR. Values are shown in box-and-whisker plots where every dot represents an independent culture and the line in the box corresponds to the median. The boxes go from the upper to the lower quartiles and the whiskers go from the minimum to the maximum value (n = 11, DMSO; n = 9, FPS-ZM1; n = 10, γ-IR; n = 9, FPS-ZM1 + γ-IR). *P* value was calculated using two-tailed t test. **f**, Quantification of percentage of time mice from different groups spent in open or closed arms in the elevated plus maze. Values are shown in a scatter dot plot where every dot represents a mouse and the line corresponds to the mean (n = 6, Control; n = 13, Vehicle + γ-IR, n = 20, FPS-ZM1 + γ-IR). Calculation of *P* values are detailed in Supplementary Table 24. **g**, Quantification of freezing time comparing different groups when trained (acquisition), when they were tested for the association between the context and the mild electric shock, recalling the information during the short-term trial, and when a substantial change of context was used to test a reduction in the behavior (Context change trial). Values are shown in a scatter dot plot where every dot represents a mouse and the line corresponds to the mean (n = 6, Control; n = 13, Vehicle + γ-IR, n = 20, FPS-ZM1 + γ-IR). Calculation of *P* values are detailed in Supplementary Table 24. **h**, Quantification of the brains long T2 component by *ex vivo* ultrahigh-field MRI in brains from experiment depicted in Fig. 6i. Values are shown in box-and-whisker plots where every dot represents a brain and the line in the box corresponds to the median. The boxes go from the upper to the lower quartiles and the whisker go from the minimum to the maximum value (n = 5, each experimental condition). *P* value was calculated using two-tailed t test.

To explore the possibility of providing an additional therapeutic option for those patients with high levels of S100A9 rather than just neglect the use of WBRT, we took advantage of our ex vivo results showing that the RAGE inhibitor FPS-ZM1 radiosensitized brain metastasis (Fig. 4i and Supplementary Fig 4h). Given the extraordinary ability of FPS-ZM1 to cross the blood–brain barrier^62^, we tested it in combination with WBRT. Once lung adenocarcinoma brain metastases were established, we provided WBRT together with the RAGE inhibitor (Fig. 6a). Both bioluminescence imaging (BLI) (Fig. 6b,c) and histology (Supplementary Fig. 6a–c) confirmed the ability of FPS-ZM1 to potentiate the benefits of radiation without evidence of increased toxicity (Supplementary Fig. 6d) while effectively decreasing the levels of the radioresistance pathway component JunB (Supplementary Fig. 6e,f). As reported with the genetic approach (Supplementary Fig. 3l), the reduction in brain metastasis with the combination therapy did not decrease the presence of neutrophils (Supplementary Fig. 6g). As a validation to further considering FPS-ZM1 as a radiosensitizer for brain metastases independently of the primary origin, we confirmed the therapeutic benefit over radiation alone in the TNBC model E0771-BrM (Extended Data Fig. 5a–c and Supplementary Fig. 6h,i).

To extend the proof-of-concept to human metastases, we applied the combination therapy ex vivo to brain metastases that relapsed after surgery and WBRT (Fig. 6d and Supplementary Table 22). Relapsed metastases obtained by a second neurosurgery were evaluated for S100A9 levels and processed to generate patient-derived organotypic cultures (PDOCs), which is an ideal platform for testing combination therapies, as we previously reported^53^. In addition to relapsed samples, we included a newly diagnosed, S100A9-negative brain metastasis with no previous local treatment (Extended Data Fig. 5d and Supplementary Table 22), which was sensitive to irradiation alone (Extended Data Fig. 5e) with no additional effects when radiation was combined with FPS-ZM1 (Extended Data Fig. 5e and Supplementary Table 22). In contrast, all relapsed metastases (*n* = 7) showed high S100A9 levels (Extended Data Fig. 5d, Supplementary Fig. 6j–o and Supplementary Table 22), and, although resistant to radiation alone, when irradiation was combined with FPS-ZM1, all PDOCs were sensitized (Fig. 6e,f, Supplementary Fig. 6j–o and Supplementary Table 22).

In summary, we report a comprehensive strategy that not only identifies patients who could benefit from WBRT but also provide a combination therapy to overcome radioresistance.

Radiosensitizers have the main risk of affecting noncancer cells, which generates toxicity^63^. Therefore, we performed a comprehensive evaluation of both general and organ-specific aspects to conclude whether FPS-ZM1 acts as a nonspecific radiosensitizer in the brain. Two cohorts of mice were treated with the same radiotherapy schedule as the one applied to experimental metastases (Fig. 1b, Fig. 6g). The impact of radiotherapy in the normal brain is not immediate as established by previous publications^64^. Consequently, 1 month after finishing the treatment with radiation and FPS-ZM1, as a reference time point established by previous literature^65,66^, mice were evaluated for their health status^67^, trained in behavioral tests^68^ measuring motor and neurocognitive functions (including anxiety/stress, learning and memory) and their brains scanned by ultrahigh-field MRI to evaluate subtle anatomical changes^69,70^. The health status (Supplementary Table 23), rotarod test (Supplementary Fig. 6p), elevated plus maze test (Extended Data Fig. 5f), water maze test (Fig. 6h), contextual fear conditioning test (Extended Data Fig. 5g), novel object recognition (NOR) test (Supplementary Fig. 6q) and pattern separation test (Supplementary Fig. 6r) results were indistinguishable between groups (Supplementary Table 24). Of note, the fourth day of the water maze test (Fig. 6h and Supplementary Table 24) might reflect a potential impact of irradiation on the ability to use hippocampal-dependent spatial maps, which would be an expected consequence of this therapy on neural stem cells^64^. Interestingly, FPS-ZM1 did not potentiate this deleterious consequence of irradiation (Fig. 6h and Supplementary Table 24). MRI analysis detected no specific anatomical differences between groups (Fig. 6i). A quantitative estimation of the brains’ long T2 component and myelin water fraction also reached the same conclusion (Fig. 6j and Extended Data Fig. 5h).

Thus, because FPS-ZM1 did not generate any undesired neurotoxic effect when combined with radiotherapy in mice, its radiosensitizing effect does not seem to affect the healthy brain tissue and its function.

## Discussion

In this report, we identify S100A9 as a common mediator of radioresistance in brain metastasis that is induced in cancer cells by interaction with the brain microenvironment. When irradiated, membranous expression of RAGE increases in metastatic cells, rendering them susceptible to S100A9-mediated resistance to WBRT. Strikingly, targeting the S100A9–RAGE axis pharmacologically with the blood–brain barrier-permeable RAGE inhibitor FPS-ZM1 restores sensitivity to radiotherapy in experimental models of brain metastasis in vivo as well as in patient-derived organotypic cultures. Because S100A9 expression correlates with poor response to radiotherapy, our findings present a novel approach to personalize radiotherapy. Based on S100A9 expression on surgical specimens or circulating levels detected by liquid biopsy, patients who would benefit from radiotherapy could be selected while patients with high resistance could be spared, thereby avoiding neurocognitive decline. Furthermore, the use of RAGE inhibitors could be used to lower the radiation dose necessary for killing tumor cells, thereby minimizing effects of irradiation on normal brain tissue and increasing the survival benefit in these patients.

In support of the highly translational nature of our findings, azeliragon or TTP488, a RAGE antagonist, has been under active investigation in clinical trials for Alzheimer’s disease^71–73^. Here, it has been shown to be clinically safe at effective doses^71,73^ and could therefore easily be repurposed to investigate its potential as a radiosensitizing agent in brain metastatic disease. Bearing the investigated effect of RAGE inhibition on Alzheimer’s disease in mind, it has been demonstrated that RAGE is also expressed on neurons and glial cells^74,75^. Hence, our comprehensive analysis discards that RAGE inhibitors sensitize healthy brain tissue to ionizing radiation in agreement with the increased neuroprotection described in RAGE knockout mice when affected by ischemic brain damage^76,77^.

Our results, together with recent data^52^, suggest that the acquisition of therapeutic resistance in brain metastasis might be linked to the induction of cellular plasticity mechanisms activated by the crosstalk with the microenvironment during colonization. The interface between the microenvironmental influence on cell plasticity and the pre-existing heterogeneity within cancer cells might promote the emergence of specific cellular and functional phenotypes (that is, cancer stem cells) that could be therapeutically relevant. Such context-specific mechanisms of cancer cell plasticity could inspire novel strategies aiming to expand personalized cancer care beyond those targeting the genomic alterations of the tumor, thus contributing to increase the limited therapeutic options available for the majority of patients with tumors in the central nervous system.

Although further studies are warranted to dissect the S100A9-dependent molecular mechanisms promoting radioresistance, a multicentric prospective study and subsequent clinical trial within the National Network of Brain Metastasis (RENACER) will further evaluate our strategy to personalize the use of radiotherapy.

## Methods

### Cell culture

Human and mouse BrM cell lines have been previously described^29,56,78^. The murine breast cancer cell line E0771-P (parental)^28^ was injected intracardially and subjected to multiple rounds of in vivo passaging to obtain BrM derivatives. Briefly, a cell suspension containing 10^5^ E0771-P cells expressing a Luciferase construct (blasticidin resistance; 19166, Addgene)^79^ in a volume of 100 μl was injected in the left cardiac ventricle of anesthetized 4- to 6-week-old C57BL/6 mice. Tumor development was monitored every 3 days by BLI using the IVIS-200 imaging system. Brain lesions were localized by ex vivo BLI and resected under sterile conditions. Tissue was minced and placed in a culture medium containing a DMEM supplemented with 0.125% collagenase III and 0.1% hyaluronidase. Samples were incubated at 37 °C for 1 h. After collagenase treatment, cells were briefly centrifuged, resuspended in 0.25% trypsin and incubated at 37**°** C for 15 min. Cells were resuspended in culture media and allowed to grow to confluence on a 10-cm dish. Two additional rounds of in vivo selection were performed. BrM3 cells were fluorescently labeled with a lentiviral vector encoding ZsGreen (632187, Clontech) and sorted for further propagation in culture or inoculation in mice. GFP expression was lost in vivo, presumably derived from immune-related responses.

MDA231-BrM2 (abbreviated as MDA231-BrM), ErbB2-BrM2 (abbreviated as ErbB2-BrM), 393N1, B16/F10-BrM3 (abbreviated as B16/F10-BrM), YUMM1.1 and 482N1 cells were cultured in DMEM supplemented with 10% FBS, 2 mM L-glutamine, 100 IU ml^−1^ penicillin/streptomycin and 1 mg ml^−1^ amphotericin B. H2030-BrM3 (abbreviated as H2030-BrM), PC9-BrM3 (abbreviated as PC9-BrM) and HCC1954-BrM1 (abbreviated as HCC1954-BrMa and HCC1954-BrMb, both sublines from HCC1954-BrM1^56^) were cultured in RPMI1640 medium supplemented with 10% FBS, 2 mM L-glutamine, 100 IU ml^−1^ penicillin/streptomycin and 1 mg ml^−1^ amphotericin B. BT-RMS cells were derived from mCherry^+^ RMS^39^ after two rounds of IC injection. BT-RMS cells were cultured in RPMI1640 medium supplemented with 10% FBS, 2 mM L-glutamine and 100 IU ml^−1^ penicillin/streptomycin. E0771-BrM3 cells (abbreviated as E0771-BrM) were cultured in RPMI1640 medium supplemented with 10% FBS, 1% HEPES, 2 mM L-glutamine, 100 IU ml^−1^ penicillin/streptomycin and 1 mg ml^−1^ amphotericin B. CN34-BrM2 (abbreviated as CN34-BrM) were cultured in M199 medium supplemented with 2.5% FBS, 10 μg ml^−1^ insulin, 0.5 μg ml^−1^ hydrocortisone, 20 ng ml^−1^ EGF, 100 ng ml^−1^ cholera toxin, 1 μg ml^−1^ amphotericin B, and 100 U ml^−1^ penicillin/streptomycin. 293 T cells were cultured in DMEM supplemented with 10% FBS, 2 mM L-glutamine, 100 IU ml^−1^ penicillin/streptomycin, and 1 mg ml^−1^ amphotericin B. Mouse glia cells were obtained from 1- to 3-day-old pups^40^. In brief, brains were mechanically dissociated and filtered through 70-µm filters. The resulting cell suspension was cultured in a petri dish for 7 days in DMEM supplemented with 10% FBS and 2 nM L-glutamine. To specifically enrich for astrocytes, at day 7, the dish was incubated overnight at 37 °C with gentle shaking. The medium was changed the next day, and predominant presence of astrocytes was confirmed by >90% GFAP staining.

### Lentiviral production and generation of stable cell lines

For lentiviral production, 293 T cells at 50–70% confluency were transfected with The RNAi Consortium (TRC) lentiviral short hairpin RNA (human or mouse) mixed with VSVG, RRE and REV packaging vectors in a 1:1 ratio using Lipofectamine 2000 (11668-030, Invitrogen). For the knockdown of human *S100A9* in H2030-BrM or mouse *s100a9* in E0771-BrM, respectively, cancer cells were infected with lentiviruses carrying pLKO.1 (control vector) or pLKO.1 sh*S100A9*#1 (clone ID: TRCN0000053803; 5′-TTGTCTGCATTTGTGTCCAGG-3′), s*hS100A9*#2 (clone ID: TRCN0000053805; 5′-ATGAACTCCTCGAAGCTCAGC-3′), sh*s100a9*#1 (clone ID: TRCN0000072043; 5′-ATACACTCCTCAAAGCTCAGC-3′) or sh*s100a9*#2 (clone ID: TRCN0000072044; 5′-TTCTTCATAAAGGTTGCCAAC-3′) (all purchased from Dharmacon) in the presence of 1 µg ml^−1^ polybrene. After infection, selection with 2 mg ml^−1^ puromycin was carried out until noninfected control cells were dead. Knockdown was verified by qRT-PCR as described below, and selected cells were maintained in culture with a supplement of puromycin for 2 weeks after selection.

To measure activation of NF-κB, cells were infected with lentivirus carrying a reporter (either cdc-5NF (gifted by C. Badr^80^) or 6xNF-κBp (gifted by A. Rodriguez^54^)) and a fluorescent color marker (mCherry). Selection was carried out by stimulation of cells with 100 ng ml^−1^ recombinant human TNF-α for 28 h and consequently sorting out mCherry^+^ cells using an Influx cell sorter (BD).

### Cell survival

A total of 1.5 × 10^4^ BrM cells were plated in 24-well-plates on poly-lysine-treated glass coverslips. After 18 h, cells were treated with 0 Gy as control or a single dose of 10 Gy γ-irradiation using an Irradiator Mark I 30 A (JL Shepherd) and Cs-137 (662 keV E_max_) as a source. Cells were fixed 72 h later in 4% paraformaldehyde (PFA), and nuclei were stained with bisbenzimide. For long-term cultures, individual cultures at specific time points were fixed. Cell survival was evaluated by manually counting nuclei in three different FOVs per coverslip.

For stimulation experiments, 200 ng ml^−1^ recombinant human S100A9 (9254-S9, R&D Systems), 20 ng ml^−1^ recombinant mouse TNF-α (315-01 A, Peprotech), 50 ng ml^−1^ recombinant mouse interferon-α (752802, BioLegend), 100 ng ml^−1^ recombinant TGF-α (239-A-100, R&D Systems) or 100 ng ml^−1^ recombinant KC/CXCL1 (250-11, Peprotech) was added to the media directly when cells were plated and again when media was changed after irradiation.

### Glia cell co-culture

A total of 1.5 × 10^4^ BrM cells were plated in the lower chamber of 24-well-plates on poly-lysine-treated glass coverslips. Co-culture media consisted of RPMI1640 media supplemented with 0.25% FBS, 2 mM L-glutamine, 100 IU ml^−1^ penicillin/streptomycin and 1 mg ml^−1^ amphotericin B. For indirect co-culture, 4.5 × 10^4^ primary glial cells were plated in a cell culture insert (0.4 µm pore size; 353495, Falcon). For direct co-culture, 4.5 × 10^4^ primary glia cells or astrocytes were plated in the lower chamber together with BrM cells. Co-cultures were irradiated with a single dose 10 Gy 18 h later. Cells were fixed 72 h later in 4% PFA and stained with bisbenzimide and anti-GFP (1:1,000; GFP-1020, Aves Labs). Cell survival was evaluated by manually counting GFP^+^ BrM cells in three different FOVs per coverslip. For RNA-seq experiments of direct co-culture, cancer cells were sorted using a BD Influx cell sorter based on their GFP expression.

### Oncosphere generation

A total of 1 × 10^3^ BrM cells were plated in low-attachment plates in Humec medium (12753018, Gibco) supplemented with 10 ng ml^−1^ basic human fibroblast growth factor (13256-029, Gibco), 20 ng ml^−1^ epidermal growth factor (EGF; E9644, Sigma-Aldrich), 5 µg ml^−1^ insulin solution from bovine pancreas (IGF1; I0516, Sigma-Aldrich), and B27 supplement (17500-044, Gibco). H2030-BrM cells were grown for 7 days and E0771-BrM cells for 4 days, so formed oncospheres were approximately the same size or number per well. After this period, oncospheres were irradiated with a single dose of 10 Gy; 72 h later, oncosphere size was evaluated using ImageJ.

### Brain organotypic cultures

Organotypic slice cultures from healthy adult mouse brains or mouse brains at the endpoint of metastatic disease (5–7 weeks in human H2030-BrM model and 2 weeks in mouse E0771-BrM model) were prepared as previously described^29,30,53^. In brief, brains were dissected in HBSS supplemented with HEPES (pH 7.4, 2.5 mM), D-glucose (30 mM), CaCl_2_ (1 mM), MgCl_2_ (1 mM) and NaHCO_3_ (4 mM), and embedded in 4% low-melting agarose (Lonza) preheated at 42 °C. The embedded organs were cut into 250-μm slices using a vibratome (Leica). Brain slices were divided at the hemisphere into two pieces. Slices were placed with flat spatulas on top of 0.8-μm-pore membranes (Sigma-Aldrich) floating on slice culture media (DMEM, supplemented HBSS, FBS 5%, L-glutamine (1 mM) and 100 IU ml^−1^ penicillin/streptomycin)). Irradiation (10 Gy, single dose) of brain slices was applied 18 h after plating. In case of treatment with inhibitors (50 µM BAY-11-7082, S2913, Selleck Chemicals; 10 µM FPS-ZM1, S8185, Selleck Chemicals), compounds were added directly into the medium when plating the brain slices. BLI was acquired 18 h after plating before irradiation (day 0) and again 72 h later (day 3). A BrdU pulse (0.2 mg ml^−1^, B9285, Sigma-Aldrich) was given by adding it into the medium 2 h before fixation. Brain slices were fixed in 4% PFA overnight at 4 °C, and then free-floating immunofluorescence was performed.

### Immunofluorescence and immunohistochemistry

Tissue for immunofluorescence was obtained after overnight fixation in 4% PFA at 4 °C. Brain tissue sections with metastases from BT-RMS cells were obtained from perfused mice following 1-h fixation with 4% PFA and 1% PFA overnight fixation at 4 °C. Slicing of the brain was done by using a vibratome (Leica) or sliding microtome (Thermo Fisher Scientific). Thickness of the slices was 250 µm or 80 µm, respectively, or 10 µm for BT-RMS, which was sectioned on a cryostat. Staining procedure was performed as described previously^30,53^. Primary antibodies were GFP (1:1,000; GFP-1020, Aves Labs), BrdU (1:500; ab6326, Abcam), Ki67 (1:500; ab15580, Abcam), GFAP (1:1,000; MAB360, Millipore), S100A9 (1:200; M0747, Dako), rodent-specific S100A9 (1:100; 73425, Cell Signaling Technology), JunB (1:100; C37F9, Cell Signaling Technology), mCherry (1:500; ab167453, Abcam), HMB-45 (1:500; ab732, Abcam), S100A8 (1:200; ab92331, Abcam), Iba1 (1:500; 19-19741, Wako), NIMP-R14 (1:100; ab2557, Abcam), TGF-α (1:100; ab9585, Abcam), Topo IIα (1:100; sc-365916, Santa Cruz Biotechnology), transglutaminase II (1:100; 3557, Cell Signaling Technology) and CXCR2 (1:100; ab65968, Abcam). Secondary antibodies were Alexa Fluor anti-chicken 488 (A11039), anti-rabbit 555 (A21429), anti-rat 633 (A21094) and anti-mouse 555 (A21422; all 1:300, Invitrogen). Immunohistochemistry against S100A9, JunB or RAGE (1:50; sc-365154, Santa Cruz Biotechnology) was performed in paraffin-embedded brain sections (5 µm) using standardized automated protocols at the CNIO Histopathology Core Facility, as described below.

### Detection of secreted S100A9 by ELISA

For detection of S100A9 in CM from different culture preparations, CM was concentrated using Amicon Ultra-15 centrifugal filter units with a 3-kDa molecular weight cutoff (C7715, Merck). Concentrated CM or serum from brain metastasis patients was assayed undiluted, and the human S100A9 DuoSet ELISA (DY5578) in combination with the respective Ancillary Reagent Kit (DY008, both R&D Systems) was used according to the manufacturer’s instruction.

### Multiplex immunoassay for detection of cytokines

The FirePlex-96 key cytokines (mouse) immunoassay panel (ab235656, Abcam) was used for unbiased detection of 17 different murine cytokines in CM from different culture preparations and lysates of brain tissue according to the manufacturer’s instructions. For generation of brain tissue lysates from tumor-naive and metastatic mouse brains, brain metastasis were macrodissected according to BLI signal and immediately snap-frozen in liquid nitrogen. Frozen tissue was subsequently homogenized and reconstituted in 1× Cell Lysis Buffer (9803, Cell Signaling Technology) plus protease inhibitors. Lysates were centrifuged for 20 min at 14,000 × *g*, and supernatants were used for the multiplex immunoassay at a concentration of 250 µg ml^−1^.

### Detection of cytokines in human and mouse tissue by RNA in situ hybridization

Paraffin-embedded tissue from mouse brain metastases was sectioned and RNAscope 2.5 VS probes for Mm-Cxcl1 (407729, ACDbio) was assayed on the Roche Ventana Discovery XT using the standardized automated protocols at the CNIO Histopathology Core Facility.

### Cell sorting and flow cytometry staining

Brains were digested in RPMI 2%FBS with collagenase IV (C5138, Sigma-Aldrich) for 30 min at 37 °C. Red blood cells were lysed with ACK Lysing Buffer (10-548E, Lonza). Myelin was removed with Percoll 22%. Single-cell suspensions were resuspended in D-PBS containing 2% FBS and 1 mM EDTA and incubated with FC-Block (553141, BD Biosciences). CD55^+^ and CD55^−^ cells were isolated using BD Influx cell sorter and anti-human CD55-APC (1:200; 555696, BD). For intracellular staining of S100A9, cells were fixed and permeabilized (BD Fixation/Permeablization Kit, 554714) and stained with primary-conjugated anti-MRP14 antibody (1:200; 350705, BioLegend). Samples were acquired in a LSR Fortessa (BD Bioscience). Doublets and DAPI^+^/ Aqua^+^ cells were excluded from analyses using FlowJo software.

### qRT-PCR

Whole RNA was isolated using the RNAeasy Mini Kit (Qiagen). RNA (1,000 ng) was used to generate complementary DNA (cDNA) using the iScript cDNA Synthesis Kit (1708890, Bio-Rad). RNA from BrM cell lines was obtained from a confluent well from a six-well plate. Gene expression was analyzed using SYBR green gene expression assays (GoTaq qPCR Master Mix, A6002, Promega). For cDNA preamplification, 7.5 ng cDNA was amplified using the PreAmp Supermix (1725160, Bio-Rad) and a Preamplification assay pool with the following primers: ACT, S100A9, TOP2A, TGM2, SPP1, ACOX2, FAM83A, IGFBP3, STC1, RSPO3, ANGTPL4 and CITED4.

The following primers were used for human genes (5′ to 3′, forward; reverse):

*S100A9* (TGGAACGCAACATAGAGACCA; CGCCATCAGCATGATGAACT),

*RAGE* (GCAGTCGGAGCTAATGGTGA; TCCACCACCAATTGGACCTC).

*TOP2A* (GACCGTCACCATGGAAGTGT; TGTTTGTTGTCCGCAGCATT),

*TGM2* (GAGATAGGACCCCTGGTTGC; TCCAGCTCCAGATCACACCT),

*SPP1* (AGCAGAATCTCCTAGCCCCA; TGGTCATGGCTTTCGTTGGA),

*ACOX2* (GACACAGGACAGAGGGGAGC; CAAGGATGTTGGTGAGCCGT),

*FAM83A* (GTGGGGTGTTCGTTTGTGTG; CGGATTTGGCCAGCGAATTT),

*IGFBP3* (CGGCATCTACACCGAGCG; TTCCTCCGACTCACTAGCAT),

*STC1* (GGCGACCACCAAAGTCAAAC; TACTTGTCGCATTGGGGTCC),

*RSPO3* (CAGCACGCCTATCGGATGT; CTCCTTGGCAGCCTTGACTA),

*ANGPTL4* (CTTGGGACCAGGATCACGAC; AGAGTCACCGTCTTTCGTGG),

*CITED4* (GGTTTCGGAGCACTACAGGT; GCAAAACCAAACCCGACTGG).The f

The following primers were used for mouse genes (5′ to 3′, forward; reverse):

*S100a9* (CACAGTTGGCAACCTTTATG; CAGCTGATTGTCCTGGTTTG)

The relative gene expression was normalized to a ‘housekeeping’ gene:

Primers used for human genes (5′ to 3′, forward; reverse):

*B2M* (AGATGAGTATGCCTGCCGTG; TCATCCAATCCAAATGCGGC) and

*ACT* (CAAGGCCAACCGCGAGAAGAT; CCAGAGGCGTACAGGGATAGCAC).

The following primers were used for mouse genes (5′ to 3′, forward; reverse):

*B2m* (GACCGGCCTGTATGCTATCC; CAGTAGACGGTCTTGGGCTC).

qPCR reaction was performed on QuantStudio 6 Flex Real-Time PCR System (Applied Biosystems) and analyzed using the corresponding QuantStudio 6 and 7 Flex software.

### Bulk RNA-seq

We used 500 ng total RNA samples. Sample RNA integrity numbers were 8.7 on average (range, 7.6–9.4) when assayed on an Agilent 2100 Bioanalyzer. Sequencing libraries were prepared with the TruSeq Stranded mRNA Sample Preparation Kit (15031047, Illumina) following the manufacturer’s instructions. This kit incorporates dUTP during second-strand cDNA synthesis, which implies that only the cDNA strand generated during first-strand synthesis is eventually sequenced. An adapter-ligated library was completed by PCR with Illumina PE primers (nine cycles). The resulting purified cDNA library was applied to an Illumina flow cell for cluster generation and sequenced on HiSeq2500 (Illumina) following the manufacturer’s protocols. Image analysis, per-cycle base calling and quality score assignment were performed with Illumina Real Time Analysis (RTA v2) software. Conversion of Illumina BCL files to BAM format was performed using Illumina2bam. Single-read sequences were analyzed by Nextpresso pipeline^81^ as follows: sequencing quality was analyzed with FastQC (http://www.bioinformatics.babraham.ac.uk/projects/fastqc/); reads were aligned to the human genome (GRCh37/hg19) using TopHat-2.0.10 (ref. ^82^), Bowtie 1.0.0 (ref. ^83^) and Samtools 0.1.19.0 (ref. ^84^); transcripts assembly, abundances estimation and differential expression were calculated with Cufflinks 2.2.1 (ref. ^85^). The estimated significance level (*P* value) was corrected to account for multiple hypotheses testing using a Benjamin and Hochberg FDR adjustment. Genes with a FDR less than or equal to 0.05 were selected as differentially expressed. Principal-component analysis plots, hierarchical clustering (Pearson’s distance) and heatmaps were generated in R and GENE-E. Furthermore, a Venn diagram of the commonly upregulated and downregulated genes between resistant and sensitive conditions was obtained using Venny (bioinfogp.cnb.csic.es/tools/venny). To this end, upregulated and downregulated programs were analyzed separately, and only genes with a FDR < 0.05 and a log_2_ ratio >1 were taken into account. Access to RNA-seq data is provided by the Gene Expression Omnibus under the ID GSE173554.

### Preparation of single-cell suspension from established metastatic lesions and scRNA-seq using 10x Genomics

Mice with IC-injected H2030-BrM cells at endpoint (5 weeks) were sacrificed and brains were extracted in precooled D-PBS 1×. Established metastatic lesions were dissected and processed with the Brain Tumor Dissociation Kit (130-095-942, Miltenyi) using gentleMACS C Tubes (130-093-237, Miltenyi) and the gentleMACS Octo Dissociator (130-096-427, Miltenyi). Mice brains were washed in cold D-PBS, cut into eight sagittal slices and transferred into a gentleMACS C Tube, with the enzymatic mixed provided by the kit, to be digested in the gentleMACS Octo Dissociator with Heaters (gentleMACS Program 37C_BTDK_01). Cell suspension was filtered with a 70-μm strainer and centrifuged at 300 × *g* for 10 min at 4 °C. For myelin removal, debris removal solution included in the Adult Brain Dissociation Kit (130-107-677, Miltenyi) was applied. The supernatant was discarded, and the cells ready for fluorescence-activated cell sorting were diluted in cold D-PBS/BSA buffer 0.04%. Cells of interest were isolated according to GFP expression using the BD Influx cell sorter. Cell suspension was centrifuged at 300 × *g* for 10 min, and the pellet was finally resuspended in 1× PBS containing 0.04% ultrapure BSA (AM2616, Thermo Fisher Scientific) at a concentration of 7 × 10^5^ cells ml^−1^, placing the cells on ice. Cells suspended in PBS-BSA were tested for the optimal viability and free of debris and aggregates. Cell sample was loaded onto a 10x Chromium Single Cell controller chip B (10x Genomics) as described in the manufacturer’s protocol (PN-1000075, Chromium Single Cell 3′GEM, Library & Gel Bead Kit v3), with an intended targeted cell recovery of ~10,000 cells. Generation of gel beads in emulsion, barcoding, gel beads in emulsion-RT clean-up, cDNA amplification and library construction were all performed as recommended by the manufacturer. scRNA-seq libraries were sequenced with an Illumina NextSeq 550 (using v2.5 reagent kits) in paired-end fashion (28 bp + 56 bp bases). Xenocell version 1.0 (ref. ^86^) was used to classify reads between host (mouse) and graft (human). The bollito pipeline^87^ was used to perform read analysis as follows: sequencing quality was checked with FastQC (http://www.bioinformatics.babraham.ac.uk/projects/fastqc/); reads were aligned to the human reference genome (GRCh38_p13 from GENCODE^88^) with STARsolo (STAR 2.7.3a (ref. ^89^)); Seurat 3.2.2 (ref. ^90^) was used to check the quality of sequenced cells, explore and quantify single-cell data, obtain cell clusters and specific gene markers; and GSEAPreranked^91^ was used to perform gene set enrichment analysis (GSEA) for the selected signature collections on a preranked gene list, setting 1,000 gene set permutations. Only those gene sets with significant enrichment levels (FDR *q*-value < 0.25) were considered.

### Intersection of NF-κB target genes and genes deregulated in radioresistant culture preparations

Established NF-κB target genes were extracted from the Hallmark Gene Set ‘HALLMARK_TNFA_SIGNALING_VIA_NFKB,’ downloaded from the Molecular Signature Database^91^. This list was intersected with genes that were significantly upregulated (adjusted *P* < 0.05; positive fold change >0) from all individual comparisons between radiosensitive and radioresistant in vitro and ex vivo culture preparations.

### Gene set enrichment analysis and Gene Ontology analysis

GSEAPreranked^91^ was used to perform GSEA for several gene signatures on a preranked gene list according to the *t*-statistic, setting 1,000 gene set permutations. Only those gene sets with significant enrichment levels (FDR *q*-value < 0.25) were considered. Gene Ontology analysis was performed using EnrichR^92,93^.

### Patient cohort of WBRT, MRI and evaluation of response

A total of 22 patients with lung cancer brain metastasis were included in this retrospective, monocentric study. All of the regulatory procedures required to comply with the laws in force in France were respected (declaration to the Health Data Hub under MR-004). All patients were treated at the radiation oncology department service of Claudius Regaud Institut (Toulouse, France) between January 2011 and February 2019. Inclusion criteria were presence of brain metastases, surgery of at least one brain metastasis, histological diagnosis of lung cancer (all histological subtypes were included) and administration of WBRT within 2.5 months after surgery as well as follow-up MRI. Patients underwent neurosurgical excision, and partial or complete character of the resection was evaluated by postoperative imaging and the neurosurgeon. Patients were included even if not all metastases were resected, and we considered all irradiated lesions (operated and non-operated) for the event of progression. WBRT was performed in 3D conformational radiation therapy at standard doses of 30 Gy in 10 fractions or 37.5 Gy in 15 fractions.

MRI was performed for the diagnosis of brain metastasis using either T1-weighted, T1-weighted with enhancement by gadolinium injection and/or T2-fluid attenuated inversion recovery (FLAIR) sequences. Postoperative imaging was routinely performed with MRI or CT within 48 h following neurosurgery. Follow-up clinical evaluation and MRI was performed at 2 months after surgery and then every 2–3 months using T1-weighted MRI with gadolinium injection. Relapse was defined following RECIST as progression of an irradiated lesion or occurrence of a brain metastatic lesion on MRI. Progression was distinguished from radionecrosis by MRI perfusion or biopsy, if clinically relevant.

### Clinical samples and immunohistochemistry

A total of 28 brain metastases from patients with lung cancer (17 cases), breast cancer (8 cases) or other primary tumors (3 cases) were obtained from the Hospital 12 de Octubre, Madrid and Hospital La Princesa, Madrid; 22 brain metastases from patients with lung cancer patients with WBRT were obtained from IUCT-Oncopole Toulouse; 22 brain metastases from patients with breast cancer were obtained from University Hospital of Turin; and 77 brain metastases from patients with melanoma (38 cases), lung cancer (20 cases) or breast cancer (19 cases) were obtained from University of Manchester. All samples were in compliance with protocols approved by their respective Institutional Review Board (IRB) and/ or national laws (4270-CEI22/20, Hospital de La Princesa; CEI PI 64_2016-v3 and CEI PI 25_2020-v2, Hospital 12 de Octubre-CNIO; Institutional Review Board of Department of Neuroscience, University of Turin; 8/NW/0092 and 13_RIMA_01, Manchester Cancer Research Centre (MCRC) Biobank ethics application 18/NW/0092 with written informed consent from the patients at The Christie NHS Foundation Trust, the study was approved by MCRC Biobank Access Committee application 13_RIMA_01, 20/EE/0002 (Preston cohort)). Immunohistochemistry was performed at the CNIO Histopathology Core Facility using standardized automated protocols. Single immunohistochemistry (Autostainer Link, Dako or Ventana Discovery XT, Roche) was initiated by performing antigen retrieval with high- or low-pH buffer (depending on the primary antibody) and, after endogenous peroxidase was blocked (peroxide hydrogen peroxidase at 3%), and slides were then incubated with the respective primary antibodies(S100A9 (1:200; M0747, Dako), RAGE (1:100; AF1179, R&D Systems) and JunB (1:100; C37F9, Cell Signaling Technology)). After the primary antibody was added, slides were incubated with the corresponding secondary antibodies conjugated with horseradish peroxidase. Immunohistochemical reactions were developed using 3,3-diaminobenzidine tetrahydrochloride. Nuclei were counterstained with Carazzi’s hematoxylin. Finally, the slides were dehydrated, cleared and mounted with a permanent mounting medium for microscopic evaluation. Positive control sections known to be primary antibody positive were included for each staining run. Immunostainings were blindly evaluated for the therapeutic response and scored by two independent researchers (L.M. and E.C.), one of them a clinical pathologist (E.C.). For the 22 samples from Toulouse, staining intensity of cancer cells was blindly evaluated for the therapeutic response and scored by a clinical neuropathologist (A.S.). For clinical correlation in samples from Toulouse, Turin and Manchester, staining intensity was evaluated as a categorial variable (negative or positive). A sample was considered positive if the number of positive cancer cells was 5% or higher. For samples from Toulouse, the S100A9 staining score was then correlated with time to relapse after radiotherapy as determined by MRI. For samples from Turin and Manchester, the S100A9 staining score was correlated with OS after brain metastasis diagnosis. For correlations between S100A9 score and clinical response, only patients who received WBRT were included. Three patients were excluded because they underwent complete surgical resection before receiving WBRT.

### Evaluation of survival in RNA-sequenced cohorts

A previously published RNA-seq dataset of 21 brain metastases from patients with breast cancer with clinical annotation^59^ has since been expanded to 45 cases (*N* = 90 patient matched samples) (GSE184869). Only patients who received radiotherapy were included in the analysis (42 cases). *S100A9* and *S100A8* gene expression was assessed using log_2_ transformed trimmed M of means-normalized counts per million (log_2_(TMM-CPM + 1)). Two groups of patients with low or high gene expression were delineated using the maximally selected rank statistics^94^, as implemented in the ‘survminer’ R package^95^ (using the RStudio 1.3 environment), and Kaplan–Meier curves were generated depicting survival after brain metastasis. All samples were in compliance with protocols approved by the corresponding IRB (University of Pittsburgh IRB#PRO15050502, Royal College of Surgeons in Ireland IRB#13/09/ICORG09/07 and the Mayo Clinic Cancer Center IRB).

For glioblastoma patient samples, the publicly available databases from the TCGA program (*n* = 262; TCGA^96^) and The Chinese Glioma Genome Atlas (*n* = 188; CGGA^97^) were interrogated and visualized using the GlioVis portal^98^. Only glioblastoma was considered as histological subtype, but all tumor types (primary, recurrence and secondary) and all subtypes (mesenchymal, proneural and classical) were included. Two groups of patients with low or high *S100A9* gene expression were delineated using the first and fourth quartile, respectively. Kaplan–Meier survival curves were generated depicting OS.

For analysis of response to radiotherapy in primary lung and breast cancers, the publicly available TCGA^96^ was interrogated using cBioPortal^99,100^. Only patients who were annotated to have received radiotherapy were included. Two groups of patients with low and high *S100A9* gene expression were delineated using the median gene expression. Kaplan–Meier plots were generated depicting progression-free survival.

### Patient cohort for liquid biopsies

A total of 71 serum samples from patients with irradiated lung cancer brain metastasis (43 cases), breast cancer brain metastasis (14 cases), melanoma brain metastasis (10 cases) and brain metastasis with other primary tumors (4 cases) were obtained from the UKE (Hamburg), RCSI (Dublin), Hospital 12 Octubre (Madrid), University of Manchester (Manchester), University Hospital Zurich (Zurich), University of Navarra (Pamplona) and IDBGI (Girona). All samples were in compliance with protocols approved by their respective institutional review board (IRB) (PV4904/PV5392, University Medical Center Hamburg-Eppendorf; REC reference 13/09, RCSI Beumont Hospital; CEI PI 25_2020-v2, Hospital 12 de Octubre-CNIO; C.0003132, Clínical Universitaria de Navarra; KEK 2021-00652, University Hospital Zurich; 017/2021, Doctor Josep Trueta University Hospital; 8/NW/0092 and 13_RIMA_01, Manchester Cancer Research Centre (MCRC) Biobank ethics application 18/NW/0092 with written informed consent from the patients at The Christie NHS Foundation Trust, the study was approved by MCRC Biobank Access Committee application 13_RIMA_01, 20/EE/0002 (Preston cohort)). Inclusion criteria were presence of brain metastasis, confirmed administration of WBRT, annotated clinical history related to time of death from diagnosis of brain metastasis and collection of serum sample before or within 2.5 months of radiotherapy. To evaluate definite survival, only patients who had died were included. Additionally, 28 patients with confirmed brain metastasis (3 cases lung cancer brain metastasis patients obtained from Hospital 12 Octubre Madrid and 25 cases breast cancer brain metastasis patients obtained from RCSI Dublin) who received no WBRT were also evaluated. Peripheral blood samples were collected in nonheparinized tubes, and blood was allowed to clot at room temperature for 30 min before separation in a centrifuge at 2,000 × *g* for 10 min. Human S100A9 ELISA was performed as described above. Serum S100A9 positivity was correlated with survival after brain metastasis diagnosis.

### Patient-derived organotypic brain cultures

Nine brain metastases from patients with lung cancer (five cases) or breast cancer (four cases) were obtained from Hospital 12 de Octubre, Madrid or Hospital La Princesa, Madrid. All samples were in compliance with protocols approved by their respective IRB (4270-CEI22/20, Hospital de La Princesa; CEI PI 64_2016-v3 and CEI PI 25_2020-v2, Hospital 12 de Octubre-CNIO). PDOCs were generated as described previously^53^. Briefly, after neurosurgical resection, brain metastasis samples were directly collected in Neurobasal-A media (21103049, Thermo Fisher Scientific) supplemented with 1 µg ml^−1^ amphotericin B, 100 IU ml^−^1 penicillin/streptomycin, 25 ng ml^−1^ basic human fibroblast growth factor, 100 ng ml^−1^ IGF1, 25 ng ml^−1^ EGF, 10 ng ml^−1^ neuroregulin-1 β1 (NRG1; 396-HB, R&D Systems) 1× N-2 supplement (17502048, Gibco) and 1× B27 supplement. Organotypic brain cultures were prepared as described above. PDOCs were treated with either DMSO or 10 µM FPS-ZM1 and 0 Gy or a single dose of 10 Gy irradiation after plating. Then, 72 h later, a BrdU pulse (0.2 mg ml^−1^) was given by adding it into the medium 4 h before fixation. Brain slices were fixed in 4% PFA overnight at 4 °C, and then free-floating immunofluorescence was performed. Proliferation was evaluated by manually counting BrdU^+^ cells in 6–11 independent cultures.

### Animal studies

All animal experiments were performed in accordance with protocol approved by the CNIO, Cajal Institute, Instituto de Salud Carlos III, CSIC and Comunidad de Madrid Institutional Animal Care and Use Committee (PROEX 168/15, PROEX 211/17, PROEX 157.0/20, PROEX 135/19). Athymic nu/nu (Harlan) and C57BL/6 mice 4–6 weeks of age were used. S100A9 ‘reporter/whole body knockout’ mice were generated by Sergei Grivennikov lab using C57Bl6 Agouti embryonic stem cell line C57BL/6N-A/a JM8A3.N1; allele S100a9tm1a(EUCOMM)Wtsi, which is originally generated by and obtained from EUCOMM/EuMMCR (clone used for injection: EPD0772_4_G10; IKMC project 85556; https://www.mousephenotype.org/data/alleles/MGI:1338947/tm1a%28EUCOMM%29Wtsi/). These embryonic stem cells were microinjected into C57Bl6 Albino blastocysts in Fox Chase Cancer Center Transgenic Facility. Mice with a greater degree of potential chimerism were further bred to C57Bl6 Albino strain (Jackson Laboratories), and germline transmission was determined by coat color and confirmed by PCR. Next, the germline transmitted mice were crossed with ubiquitous Cre-deleter strain to achieve deletion between the first and third loxP sites, including the deletion of pivotal exon 3 but leaving LacZ reporter in place, thereby creating a ‘knockout/reporter’ allele. Cre-deleter was then removed by subsequent backcrossing of mice with C57BL/6 N mice and selecting Cre-negative progeny. Mice were genotyped for floxed, wild-type, reporter/LacZ or loxP-deleted alleles by PCR using the following primers: P1:s100a9-LacZ-forward, 5′-TCAGCCGCTACAGTCAACAG-3′; P2:s100a9-fw2 5′-GGTGGGGTATGACTGCAAGA-3′, P3:s100a9-rv9 5′-AACTGATGGCGAGCTCAGAC-3′, P4:s100a9-rv2 5′-ACAAATAGAAATGGAAACACCTTCT-3′. The presence of wild-type allele resulted in an approximately 200-bp band, floxed allele (370 bp); deletion of the floxed allele was detected by an approximately 230-bp band and LacZ allele at approximately 510 bp. For experiments using this model, only littermate controls were used.

A brain metastatic derivative of the syngeneic E0771 model (E0771-BrM3) was established according to a previously described protocol^27,78^. For IC models of brain colonization, 100 μl PBS containing 10^5^ cancer cells was injected into the left ventricle. Alternatively, a volume of 2 μl PBS containing 2.5 × 10^4^ cancer cells was IC injected (right frontal cortex, approximately 1.5 mm lateral and 1 mm caudal to the bregma, and to a depth of 2 mm) using a gas-tight Hamilton syringe and a stereotactic apparatus. Brain colonization was analyzed in vivo and ex vivo by BLI. Briefly, mice were anesthetized using 3% isoflurane, injected retro-orbitally with D-Luciferin (150 mg kg^−1^) and imaged with an IVIS Spectrum Xenogen machine (Caliper Life Sciences). Bioluminescence analysis was performed using Living Image software v3.

To study the radiation response of BrM cells injected subcutaneously in the head of the animal, 5 × 10^5^ cells were resuspended in 100 µl PBS mixed with 50% reduced growth factor Matrigel (356238, Corning) and injected subcutaneously. To study the radiation response of experimental lung metastasis, 5 × 10^5^ E0771-BrM cells were resuspended in 200 µl PBS and injected into the lateral tail vein.

### Radiotherapy in vivo

Two weeks after IC injection of H2030-BrM cells, the presence of established brain metastases was confirmed by BLI, defined as head photon flux values >10^4^ (measured as p s^−1^ cm^−^^2^ sr^−1^). Animals were placed in a methacrylate chamber and anesthetized using isoflurane as described above. The irradiator Mark I 30 A was used for irradiation of anesthetized mice. Cranial irradiation was delivered using an opposed lateral-beam geometry, collimated to produce a 0.5-cm-diameter field at the depth of interest. The body was protected by a 3-cm custom-made shield device that covers the mice except for the head. None of the immobilization devices were in the beam path. The doses were not corrected for surface curvature or tissue heterogeneities, but dosimetry standardization was adequate for reproducibility of our findings.

The WBRT protocols applied include one mimicking the exact clinical procedure (fractionated dose of 3 Gy per day for 5 consecutive days, followed by 2 days without irradiation. Afterwards, 3 Gy per day for additional 5 days were given. The total applied dose was 30 Gy per mouse^25^. Another published strategy from which we eliminated the chemotherapy included fractionated doses of 5.5 Gy per day for 3 consecutive days. The total applied dose was 16.5 Gy per mouse^24^. A third protocol followed a mathematical algorithm previously applied to preclinical glioma model (day 1: 3 Gy at 8 am; day 2: 1 Gy at 4 pm; day 3: no radiation; day 4: 1 Gy at 9 am, 1 pm, 5 pm; day 5: 1 Gy at 9 am, 1 pm, 5 pm). The total applied dose was 10 Gy per mouse^26^.

For the syngeneic brain metastasis model using E0771-BrM, WBRT^25^ was initiated 3 days after intracranial injection of cancer cells, as determined by confirmed presence of brain metastasis by BLI.

In experiments using RAGE inhibitor FPS-ZM1 (500 mg kg^−1^ per day in 5% DMSO/corn oil intraperitoneal), treatment was initialized either on the day 12, in case of human H2030-BrM, or on day 3, for the syngeneic E0771-BrM model, after mice were randomized according to BLI signal.

In all models, mice were followed up with regular imaging by BLI until the humane endpoint was reached.

### Evaluation of health status and rotarod test in treated mice

General health status was assessed by a husbandry caregiver blinded to experimental groups that evaluated mice one month after finishing the treatments. The parameters scored included evaluation of the skin, fur and eyes, movement and the response of mice after manipulation.

Rotarod test was used for assessment of motor coordination of mice. Animals were transferred to the behavioral testing room 10 days before the actual experiment for acclimatization and training. Mice were trained three times before the actual experiment in order for each mice to achieve 1 min at 5 r.p.m. speed on the rotarod. For the final experiment, mice were placed on the rotarod in a blinded manner, and we waited for 5 s before starting the clock to avoid failure due to misplacement of the mice by the handler. Mice were tested at three different static speeds and given three trials per speed, with enough resting time between speeds. If a mouse achieved 60 s on the rotarod at a given speed, it was considered a success, and no further trials were conducted; If not, the latency to fall in all three trials was averaged. After each trial and between mice, the instrument was cleaned with 75% ethanol solution-wetted tissue paper.

### Behavioral assessment

#### Activity assessment

The activity cage experiment was performed to evaluate general locomotor function. The cage consists of an open field arena (21 × 21 cm) with horizontal and vertical beam sensors. Disruptions of the beam were recorded as activity counts. The animals were habituated to the room for at least 30 min and then placed individually in the center of the arena and allowed to freely explore for 5 min. In the first day of the protocol, the spontaneous locomotor activity was measured for 5 min in a novel arena. The following day, animals were placed in the same arena for another 5 min. Behavioral measures included total distance traveled, horizontal activity (counts) and vertical activity (counts) were recorded. The movement of the animals was recorded and analyzed using MUX_XYZ16L-8 Animal Activity software (Cibertec Actimeter XYZ-8). After each animal, the arena was cleaned with 0,03% acetic acid dilution between trials.

#### Elevated plus maze

Anxiety-like behavior was evaluated using the video elevated plus maze test and software. This test takes advantage of the conflict between the innate fear mice have for open elevated areas and their desire to explore new environments. The natural tendency of anxious rodents to spend more time in dark, closed versus open spaces. The maze is elevated 40 cm from the floor and consists of a central platform (5 m wide and 35 cm long) with two open arms (5 cm wide and 330 cm long) and two closed arms (5 cm wide, 330 cm long and 315 cm high). After habituation to the experimental room, animals were placed at the center of the maze, facing an open arm and were allowed to explore the maze freely for 5 min. The time exploring the maze was recorded, and the duration in each arm was manually scored. The arena was cleaned with 0.03% acetic acid dilution between trials.

#### NOR

A NOR protocol was applied in an open wooden box of 42 × 32 × 31 cm. The test consisted of three phases: training, short-term memory evaluation and long-term memory evaluation. During the training phase, each animal was placed in the arena containing two different objects in the center of the box (objects A and B) and was allowed to explore them freely for 15 min. Next, each animal was removed and put back in the home cage. One hour after training, animals were placed back in the NOR arena containing a familiar object (object A) and a novel object (object C) and allowed to explore them for 10 min. At the end of this phase, all animals were placed in their home cage. Twenty-four hours after training, animals were tested again for 10 min in the arena containing the familiar object (A) and a novel object (object D). The box was cleaned with water with 0.03% acetic acid dilution between trials. Time exploring each object was manually scored. The discrimination index (DI) was calculated using the following formula: DI = (time exploring B, C or D − time exploring A)/(time exploring B, C or D + time exploring A).

#### Object pattern separation

Object pattern separation protocol was applied in a polyvinyl chloride (PVC) circular arena (35 cm in diameter and 20 cm high)^101^. The test consisted of two phases: training and test. In the training phase, animals were left to explore the arena for 4 min. Two equal plastic columns were placed symmetrically along the diameter, at the same distance from the walls. At the end of the phase, animals were placed again in their home cage. Sixty minutes after the beginning of the training phase, animals were put back in the circular arena for 4 min (test phase). One of the columns was displaced three positions. The arena was cleaned with water with 0.03% acetic acid dilution between trials. Time exploring each column was manually scored. DI was calculated using the following formula: DI = (time exploring moved − time exploring fixed)/(time exploring moved + time exploring fixed).

#### Contextual fear conditioning

A fear conditioning apparatus (Ugo Basile Fear Conditioning 2.1, 46003 Mouse Cage) was used to test contextual aversive memory and context discrimination abilities. The test consisted of three phases: training, test and context change. In the training phase (5 min), animals were left to freely explore the conditioning chamber (17 × 17 × 25 cm) with walls with checkers pattern (context A) for 3 min. At minutes 3:00, 3:30 and 4:00, a floor shock (0.5 mA, 2 s duration) was administered through the floor grid. The animal was left another minute in the cage before the ending of the test. Test phase was conducted 24 h after the training phase. Animals were put back in the conditioning chamber with checkers pattern (context A) and left to explore for 5 min, no shock applied. Then, 24 h after the test phase, the context change phase was performed. The animals were placed again in the conditioning chamber but with white walls (context B). Freezing time was automatically scored with ANY-MAZE (v6.0).

#### Morris water maze

The Morris water maze is a well-recognized test used to study spatial memory and learning. Briefly, animals were placed in a pool of water where they had to swim to a hidden platform. After a 1-day habituation trial, in which preferences between quadrants in the different experimental groups were ruled out, animals learned to find a hidden platform at a fixed position over the following four acquisition days (two trials on acquisition day 1, three trials per day from days 2 to 4, 60 s each trial, plus 15 s in the platform). If an animal failed to reach the platform, then it was placed on it by the experimenter. The positions to enter the maze were randomly selected each day and the same positions used for all animals each day. The time spent to escape the water to the platform (escape latency) was used to evaluate the performance. The data were compared by a repeated measures analysis of variance (ANOVA) and a one-way ANOVA to test differences between groups if the repeated measures ANOVA yielded significant results. SPSS v.27 was used to run statistical comparisons. After acquisition, the animals were tested on a probe test 24 h after the last acquisition day. Probe trial consisted of a single trial 90 s long without platform. The time spent swimming in each quadrant of the maze, and the number of crossings over the virtual position of the platform (both in the platform quadrant and in homologous virtual positions in the rest of quadrants) were used to evaluate the performance. One-way ANOVA was used to analyze the data.

### MRI of brains from treated mice

All experiments were preapproved by the competent institutional and national authorities and were carried out in accordance with European Directive 2010/63. MRI experiments were conducted on a 16.4 T ultrahigh-field MRI scanner (Bruker) resonating at 700.34 MHz, equipped with a micro2.5 imaging probe with gradients capable of producing gradients up to 1,500 mT/m in all directions, and a transmit/receive 15 mm coil. Following routine adjustments, the MRI scans consisted of multi-spin echo experiments with a repetition time of 11.5 s and 100 echo times spaced by 2.3 ms from 2.3 ms to 230 ms. The field of view was 11 × 11 mm^2^ and the matrix size was 130 × 130, leading to an in-plane resolution of 85 × 85 µm2, and the slice thickness was 600 µm (25 slices). The data were analyzed in MATLAB (MathWorks). Briefly, data were reconstructed, transformed to real-valued data^102^, denoised using the Marcenko-Pastur PCA approach^103,104^ (2D denoising window [10 10]), Gibbs unrung^105^, and the thus preprocessed data were then voxel-wise fitted to a two-component system using a nonlinear least squares constraining the upper and lower bounds of parameters.

### Image acquisition and analysis

Images were acquired with a Leica SP5 upright confocal microscope with ×10, ×20, ×40 and ×63 objectives and analyzed with Fiji software 1.0 and Definiens developer XD 2.5. Immunohistochemistry images were captured with Zen Blue Software v3 (Zeiss), and whole slides were acquired with a slide scanner (AxioScan Z1, Zeiss). Oncospheres were imaged with a Leica DMi1 inverted microscope.

### Statistical analysis

Data were analyzed using GraphPad Prism 8 software (GraphPad Software). Distribution of datasets was assessed using a Shapiro–Wilk test. If datasets followed a normal distribution and comparisons were done between two experimental groups, then an unpaired, two-tailed Student’s *t*-test was used. For nonparametric datasets, an unpaired, two-tailed Mann–Whitney test was performed. For survival curves, *P* values were obtained with log-rank (Mantel–Cox) two-sided tests.

### Reporting Summary

Further information on research design is available in the Nature Research Reporting Summary linked to this article.

## Online content

Any methods, additional references, Nature Research reporting summaries, source data, extended data, supplementary information, acknowledgements, peer review information; details of author contributions and competing interests; and statements of data and code availability are available at 10.1038/s41591-022-01749-8.

## Supplementary information

Supplementary Information Supplementary Figs. 1–6 and Tables 1–23.

Reporting Summary

## Data Availability

Data shown are available in the main text or the Supplementary Informatiom. Access to RNA-seq data is provided by the Gene Expression Omnibus under the ID GSE173554. Access to scRNA-seq data is provided by the Gene Expression Omnibus under the ID GSE189024. Access to extended RNA-seq of breast cancer patients is provided by the Gene Expression Omnibus under the ID GSE184869. References genomes used (GRCh38_p13 and GRCh37/hg19) are available at https://www.ncbi.nlm.nih.gov/assembly/GCF_000001405.39 and https://www.ncbi.nlm.nih.gov/assembly/GCF_000001405.13/. The TCGA and CGGA glioblastoma data used are available at http://gliovis.bioinfo.cnio.es. TCGA lung and breast primary cancer data used are available at https://www.cbioportal.org.
