# Supplementary material for: Stratification of radiosensitive brain metastases based on an actionable S100A9/RAGE resistance mechanism
Source: Nat Med. 2022 Apr 11;28(4):752–65. doi: 10.1038/s41591-022-01749-8 (PMC9018424; doi:10.1038/s41591-022-01749-8)
Supplement: Supplementary file 1 — Supplementary Figs. 1–6 and Tables 1–23. [file 41591_2022_1749_MOESM1_ESM.pdf]

nature portfolio

https://doi.org/10.1038/s41591-022-01749-8

Supplementary information

Stratification of radiosensitive brain metastases based on an actionable S100A9/RAGE resistance mechanism

In the format provided by the authors and unedited

Supplementary Information

Title: Stratification of radiosensitive brain metastases based on an actionable S100A9/RAGE resistance mechanism.

Author list:

Cátia Monteiro[1**], Lauritz Miarka[1**], María Perea-García[1], Neibla Priego[1], Pedro García-Gómez[1], Laura Álvaro-Espinosa[1], Ana de Pablos-Aragoneses[1], Natalia Yebra[1], Diana Retana[1], Patricia Baena[1], Coral Fustero-Torre[2], Osvaldo Graña-Castro[2], Kevin Troulé[2], Eduardo Caleiras[3], Patricia Tezanos[4], Pablo Muela[4], Elisa Cintado[4], José Luis Trejo[4], Juan Manuel Sepúlveda[5], Pedro González-León[6], Luis Jiménez-Roldán[6,7], Luis Miguel Moreno[6], Olga Esteban[6], Ángel Pérez-Núñez[6,7], Aurelio Hernández-Lain[8], José Mazarico Gallego[9], Irene Ferrer[10,11], Rocío Suárez[10,11], Eva M. Garrido-Martín[10,11], Luis Paz-Ares[9,10,11,12], Celine Dalmasso[13], Elizabeth Cohen-Jonathan Moyal[13], Aurore Siegfried[14], Aisling Hegarty[15], Stephen Keelan[15], Damir Varešlija[15], Leonie S. Young[15], Malte Mohme[16], Yvonne Goy[17], Harriet Wikman[18], Jose Fernández-Alén[19], Guillermo Blasco[19], Lucía Alcázar[19], Clara Cabañuz[20], Sergei I. Grivennikov[21,†], Andrada Ianus[22], Noam Shemesh[22], Claudia C. Faria[23,24], Rebecca Lee[25,26], Paul Lorigan[25,26], Emilie Le Rhun[27], Michael Weller[27], Riccardo Soffietti[28], Luca Bertero[29], Umberto Ricardi[30], Joaquim Bosch-Barrera[31,32,33], Elia Sais[31,32], Eduard Teixidor[31,32], Alejandro Hernández-Martínez[31,32], Alfonso Calvo[11,34], Javier Aristu[35], Santiago M. Martin[36], Alvaro Gonzalez[37], Omer Adler[38], Neta Erez[38], RENACER[39], Manuel Valiente[1*].

Affiliations:

[1]Brain Metastasis Group, CNIO; Madrid, 28029, Spain.

[2]Bioinformatics Unit, CNIO; Madrid, 28029, Spain.

[3]Histopathology Core Unit, CNIO; Madrid, 28029, Spain.

[4]Department of Translational Neuroscience, Cajal Institute, CSIC; Madrid, 28002, Spain.

[5]Neuro-Oncology Unit, Hospital Universitario 12 de Octubre; Madrid, 28041, Spain.

[6]Neurosurgery Unit, Hospital Universitario 12 de Octubre; Madrid, 28041, Spain.

[7]Department of Surgery, Universidad Complutense de Madrid, Madrid, 28040; Spain.

[8]Neuropathology Unit, Hospital Universitario 12 de Octubre; Madrid, 28041, Spain.

[9]Medical Oncology, Hospital Universitario 12 de Octubre; Madrid, 28041, Spain.

[10]CNIO-H12O Clinical Cancer Research Unit, Fundación de Investigación Biomédica i+12 and CNIO, Madrid, 28029, Spain.

[11]CIBERONC

[12]Department of Medicine, Universidad Complutense de Madrid, Madrid, 28040, Spain.

[13]Radiation Oncology Department, Institut Claudius Regaud, IUCT-Oncopole; Toulouse, 31100, France.

[14]Anatomopathology Department, CHU Toulouse, IUCT-Oncopole; Toulouse, 31100, France.

[15]Endocrine Oncology Research Group, RCSI University of Medicine and Health Sciences; Dublin, D02 YN77, Ireland.

[16]Department of Neurosurgery, University Medical Center Hamburg-Eppendorf; Hamburg, 20246, Germany.

[17]Department of Radiation Oncology, University Medical Center Hamburg-Eppendorf; Hamburg, 20246, Germany.

[18]Department of Tumor Biology, University Medical Center Hamburg-Eppendorf; Hamburg, 20246, Germany.

[19]Department of Neurosurgery, Hospital Universitario de La Princesa; Madrid, 28006, Spain.

[20]Department of Pathology, Hospital Universitario La Paz; Madrid, 28046, Spain.

[21]Cancer Prevention and Control Program, Fox Chase Cancer Center, 333 Cottman Ave, Philadelphia, PA, 19111, United States of America.

[22]Champalimaud Research, Champalimaud Centre for the Unknown; Lisbon, 1400-038, Portugal.

[23]Instituto de Medicina Molecular João Lobo Antunes, Facultade de Medicina, Universidade de Lisboa; Lisboa, 1649-028, Portugal.

[24]Department of Neurosurgery, Hospital de Santa Maria, Centro Hospitalar Universitário Lisboa Norte (CHULN); Lisboa, 1649-028, Portugal.

[25]Faculty of Biology Medicine and Health, The University of Manchester; Manchester, M13 9PL, United Kingdom.

[26]The Christie NHS Foundation Trust; Manchester, M20 4BX, United Kingdom.

[27]Department of Neurology, University Hospital Zurich; Zurich, 8091, Switzerland.

[28]Division of Neuro-Oncology, Department of Neuroscience Rita Levi Montalcini, University of Turin; Turin, 10126, Italy.

[29]Department of Medical Sciences, University of Turin; Turin, 10126, Italy.

[30]Department of Oncology, University of Turin; Turin, 10126, Italy.

[31]Department of Medical Oncology, Catalan Institute of Oncology, Doctor Josep Trueta University Hospital; Girona, 17007, Spain.

[32]Girona Biomedical Research Institute (IDIBGi); Salt, 17190, Spain.

[33]Department of Medical Sciences, University of Girona; Girona, 17003, Spain.

[34]IDISNA, Program in Solid Tumors, Center for Applied Medical Research (CIMA), University of Navarra; Pamplona, 31008, Spain.

[35]Department of Oncology, University Clinic of Navarra; Madrid, 28027, Spain.

[36]Department of Oncology, University Clinic of Navarra; Pamplona, 31008, Spain.

[37]Department of Biochemistry, University Clinic of Navarra; Pamplona, 31008, Spain.

[38]Department of Pathology, Sackler Faculty of Medicine, Tel Aviv University; Tel Aviv, 69978, Israel.

[39]Biobank; CNIO; Madrid, 28029; Spain.

**These authors contributed equally to this work.

*Corresponding author. Email: mvaliente@cnio.es.

[†]Current address: Departments of Medicine and of Biomedical Sciences, Cedars-Sinai Cancer, Cedars-Sinai Medical Center; Los Angeles, CA, 90048, United States of America.

Supplementary Figures.

Supplementary Figure 1.

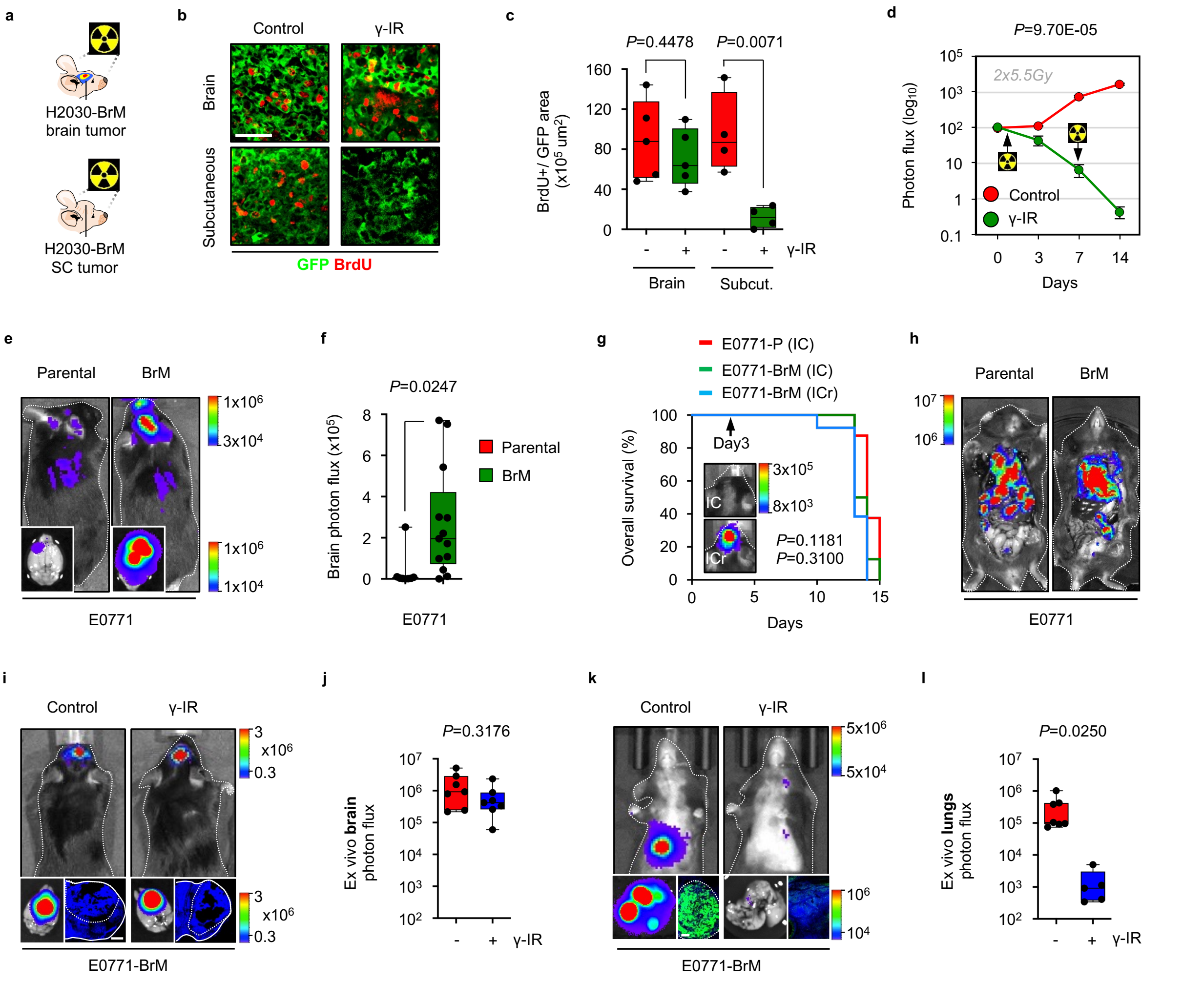

Acquired radioresistance in experimental brain metastasis. a, Schema of experimental design. **b**, Representative images of H2030-BrM tumors (GFP+) before and after 72 hours after irradiation with a single dose of 10Gy. Scale bar: 50 µm. **c**, Quantification of BrdU+ cancer cells from experiment in (**b**). Values are shown in box-and-whisker plots where every dot represents an independent brain metastasis or subcutaneous tumor and the line in the box corresponds to the median. The boxes go from the upper to the lower quartiles and the whiskers go from the minimum to the maximum value (n=4, non-irradiated brain metastases; n=4, irradiated brain metastases; n=4, non-irradiated subcutaneous tumors; n=4, irradiated subcutaneous tumors). *P* value is calculated using two-tailed t test. **d**, Quantification of H2030-BrM cells after long-term culture and

fractionated doses of irradiation. A total dose of 11Gy was delivered into two doses of 5.5Gy. Each dot indicates the mean bioluminescence value at each time point normalized for the initial value of the control ± sem (n=3 wells per condition). *P* value is calculated using two-tailed t test at the last time point. **e**, Representative images of C57BL/6 mice two weeks after being inoculated with E0771-P or E0771-BrM cells IC. Images in the lower left corner show the BLI of brains *ex vivo*. Dotted line indicates the mouse body. **f**, Quantification of BLI in the head of mice intracardially injected with E0771-P or E0771-BrM cells. Values are shown in box-and-whisker plots where every dot represents a different brain and the line in the box corresponds to the median. The boxes go from the upper to the lower quartiles and the whiskers go from the minimum to the maximum value (n=8 mice, E0771-P; n=13 mice, E0771-BrM). *P* value is calculated using two-tailed t test. **g**, Survival curve comparing E0771-P (n=8) with E0771-BrM (n=8) cell lines inoculated IC and the E0771-BrM inoculated intracranially (Icr) (n=13). *P* value is calculated using log rank (Mantel-Cox) test two-sided (E0771-P versus E0771-BrM from IC injection, P=0.1181; E0771-BrM from IC versus E0771-BrM from Icr injection, P=0.3100). Representative BLI images of mice inoculated with E0771-BrM IC versus Icr are also shown at 3 days post-inoculation. **h**, Representative images of mice inoculated with E0771-P or E0771-BrM IC showing the extensive burden of extracranial metastases with both cell lines. **i**, Representative images of C57BL/6 mice two weeks after being inoculated with E0771-BrM cells iCr and left untreated or treated with WBRT (10 x 3Gy). Dotted line indicated the body of mice. Additional images show the BLI of brains *ex vivo* as well as the histology where similar tumor masses are detected. Dotted line indicated the metastasis. Scale bar: 10 mm. **j**, Quantification of BLI in the brain *ex vivo*. Values are shown in box-and-whisker plots where every dot represents a different brain and the line in the box corresponds to the median. The boxes go from the upper to the lower quartiles and the whiskers go from the minimum to the maximum value (n=7 mice in each experimental condition). Y-axis is scaled logarithmic. *P* value is calculated using two-tailed t test. **k**, Representative images of C57BL/6 mice 3 weeks after being inoculated with E0771-BrM cells into the lateral tail-vein and left untreated or treated with irradiation to the lung only (10 x 3Gy). Dotted line indicated the body of mice. Additional images show the BLI of lungs *ex vivo* as well as the histology. Dotted line indicated the metastasis. Scale bar: 100 μm. **l**, Quantification of bioluminescence of the lungs *ex vivo*. Values are shown in box-and-whisker plots where every dot represents a different lung and the line in the box corresponds to the median. The boxes go from the upper to the lower quartiles

and the whiskers go from minimum to maximum value (n=7, non-irradiated mice; n=5, irradiated mice). Y-axis is scaled logarithmic. *P* value is calculated using Mann-Whitney test, two-sided.

Supplementary Figure 2.

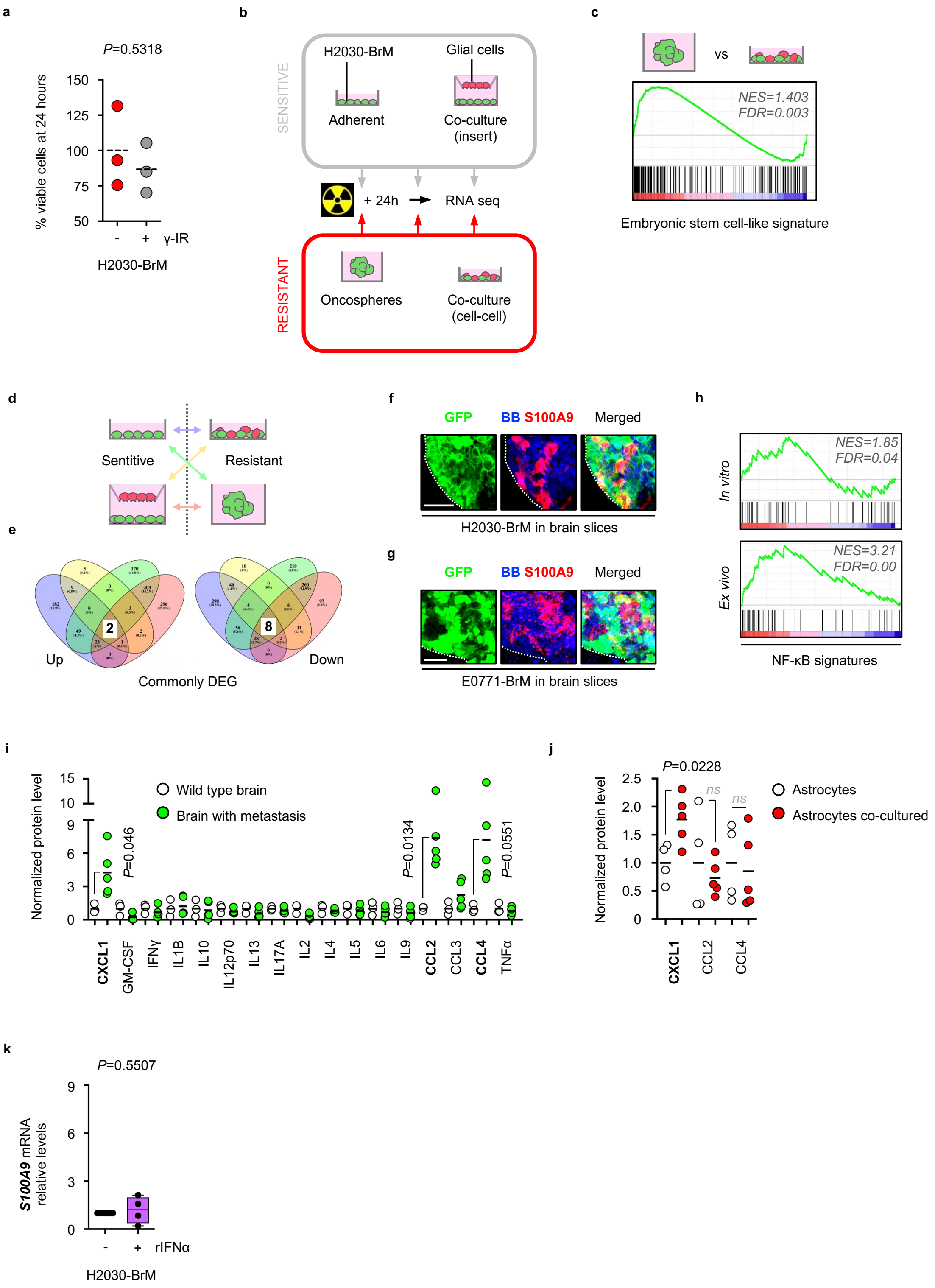

Contact-dependent astrocyte-released cytokines induce S100A9 secretion in cancer cells

triggering NF-κB activation. a, Quantification of *in vitro* viable cell fraction 24 hours after 10Gy irradiation as determined by manual cell counting of DAPI+ nuclei. Values are percentages of un-irradiated controls and shown in a dot plot where each dot represents an independent culture and the line in the box corresponds to the median (each n=3). *P* value was calculated using two-tailed t-test. **b**, Schema of experimental design. **c**, Gene Set Enrichment Analysis (GSEA) was performed on a pre-ranked list of differentially expressed genes between resistant culture preparations including oncospheres and co-cultures. The embryonic stem cell-like signature is significantly enriched in oncospheres (Supplementary Table 2). The green curve corresponds to the Enrichment Score, while the Normalized Enrichment Score (NES) and the False Discovery Rate (FDR) are shown in the graph. **d**, Schema of the different comparisons done with the transcriptomes from resistant and sensitive preparations to identify commonly deregulated genes. **e**, Venn diagram indicating commonly upregulated and downregulated genes in resistant *in vitro* surrogates under the condition shown in (**b**). Colors in the circles represent the comparison indicated with the arrows in (**d**) with the same color code. **f**, **g**, Representative immunofluorescent images of H2030-BrM and E0771-BrM cells plated on organotypic brain slices *ex vivo.* Scale bars: 50 µm. This experiment was repeated three times with similar results. **h**, Representative graphs of positively enriched gene sets depicting induction of NF-κB among a pre-ranked list of differentially expressed genes from resistant culture preparations *in vitro* and *ex vivo* (Supplementary Table 6). The green curve corresponds to the Enrichment Score, while the Normalized Enrichment Score (NES) and the False Discovery Rate (FDR) are shown in each graph. **i**, Quantification of 17 murine cytokines in wild type mice brains and mice brains with H2030-BrM brain metastasis by multiplex cytokine assay. Values indicate cytokine concentration and are shown in a dot plot where each dot represents a brain and the line corresponds to the median (n=3, normal brains; n=5, brain metastasis brains). *P* values are calculated using two-tailed t test. **j**, Quantification of 3 selected murine cytokines in conditioned media (CM) from astrocytes cultured alone and astrocyte-H2030-BrM co-cultures *in vitro* by multiplex cytokine assay. Values indicate relative cytokine concentration to the astrocyte culture alone and are shown in a dot plot where each dot represents an independent experiment and the line corresponds to the median (n=4, CM from astrocytes; n=5, CM from astrocyte-H2030-BrM co-cultures). *P* values are calculated using two-tailed t test. **k**, Quantification of *S100A9* expression levels in H2030-BrM 3d after a single dose (10Gy) of radiation and treatment with either control or rIFNα. Expression values were normalized to their

respective non-irradiated control for each culture condition. Values are shown as box-and-whisker plots where every dot represents an independent experiment and the line in the box corresponds to the median (n=4, each experimental condition). Whiskers go from minimum to maximum values. *P* value is calculated using two-tailed t test.

Supplementary Figure 3.

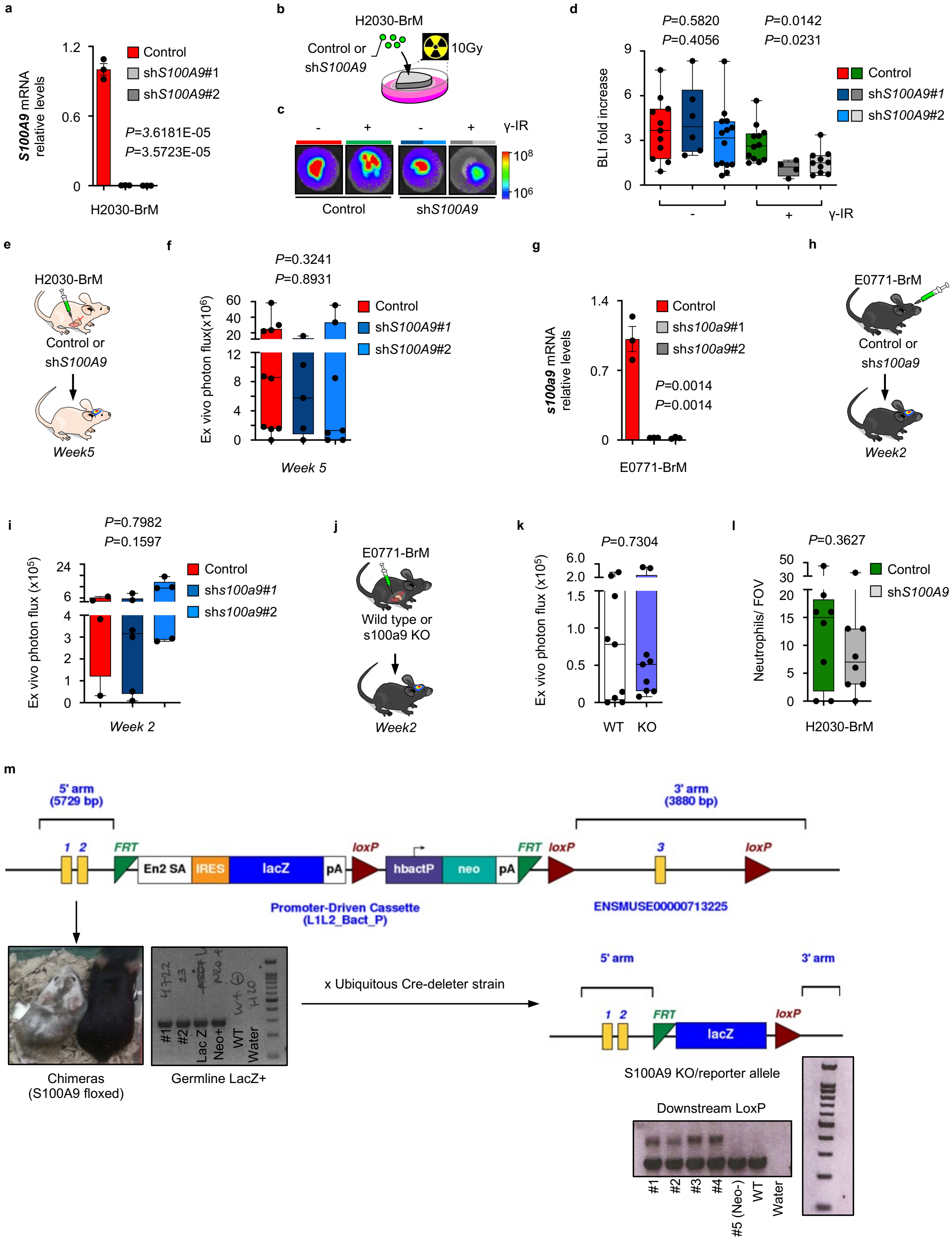

Targeting S100A9 in cancer cells radiosensitizes experimental lung and breast cancer brain

metastases in a NF-κB-JunB-dependent manner. a and **g**, Quantification of human *S100A9* (**a**) or mouse *S100a9* (**g**) gene expression by qRT-PCR in H2030-BrM and E0771-BrM cells transduced with a scrambled shRNA as control or two different shRNA against human *S100A9* (sh*S100A9*#1 and sh*S100A9*#2) or mouse *S100a9* (sh*S100a9*#1 and sh*S100a9*#2), respectively. Bars represents the mean ± s.e.m where every dot represents an independent culture (n = 3 cultures per experimental condition). P value was calculated using two-tailed t-test. **b**, Schema of experimental design. **c**, Representative bioluminescence images of brain organotypic cultures with metastatic cancer cells, transduced with a shRNA against *S100A9* or a scrambled shRNA as control, 72 hours after irradiation (10Gy, single dose) or no irradiation (0Gy). Color coded lines correlates with the corresponding bars in (**d**). **d**, Quantification of BLI from metastatic cells, transduced with either a scrambled shRNA as control (control), or one of two different shRNAs against *S100A9* (sh*S100A9*#1, sh*S100A9*#2), growing in organotypic brain cultures 72 hours after irradiation (10Gy, single dose) or no irradiation. Values were normalized to their respective non-irradiated controls and are shown in box-and-whisker plots where every dot represents an independent culture and the line in the box corresponds to the median. The boxes go from the upper to the lower quartiles and the whiskers go from the minimum to the maximum value (n=11, non-irradiated brain slices with H2030-BrM control; n=6, non-irradiated brain slices with H2030-BrM sh*S100A9#1*; n=14, non-irradiated brain slices with H2030-BrM sh*S100A9#2*; n=12, irradiated brain slices with H2030-BrM control; n=4, irradiated brain slices with H2030-BrM sh*S100A9#1*; n=10, irradiated brain slices with H2030-BrM sh*S100A9#2*). *P* value is calculated using two-tailed t test. **e** and **h**, Schema of experimental design. **f** and **i**, Quantification of the bioluminescence at the endpoint of the experiments depicted in (**e**, **h**) comparing brains from non-irradiated mice injected with H2030-BrM (**f**) or E0771-BrM (**i**) transduced with corresponding shRNA. Values are shown in box-and-whisker plots where every dot represents a different brain and the line in the box corresponds to the median. The boxes go from the upper to the lower quartiles and the whiskers go from the minimum to the maximum value (n=10, H2030-BrM control; n=5, H2030-BrM sh*S100A9*#1; n=7, H2030-BrM sh*S100A9*#2; n=4, E0771-BrM control; n=6, E0771-BrM sh*S100a9*#1; n=5, E0771-BrM sh*S100a9*#2). *P* value is calculated using two-tailed t test. **j**, Schema of experimental design. **k**, Quantification of the *ex vivo* photon flux at the endpoint of the experiment depicted in (**j**) comparing brains from S100A9$^{+/+}$ and S100A9$^{-/-}$ mice injected intracardially with E0771-BrM. Values are shown in box-and-whisker plots where every

dot represents a different brain and the line in the box corresponds to the median (n=9, S100A9$^{+/+}$ mice; n=9, S100A9$^{-/-}$ mice). The boxes go from the upper to the lower quartiles and the whiskers go from the minimum to the maximum value. *P* value was calculated using Mann-Whitney test, two-sided. **l**, Quantification of the numbers of neutrophils per FOV in brain metastasis from H2030-BrM control or sh*S100A9,* using the NIMP-R14 staining. For both conditions equal numbers of lesions of different sizes were chosen. Values are shown in box-and-whisker plots where every dot represents a metastatic lesion and the line in the box corresponds to the median. The boxes go from the upper to the lower quartiles and the whiskers go from minimum to maximum value (n=8 FOV from 2 brains, H2030-BrM control; n=8 FOV from three brains, H2030-BrM sh*S100A9*). *P* value was calculated using two-tailed t test.

Supplementary Figure 4.

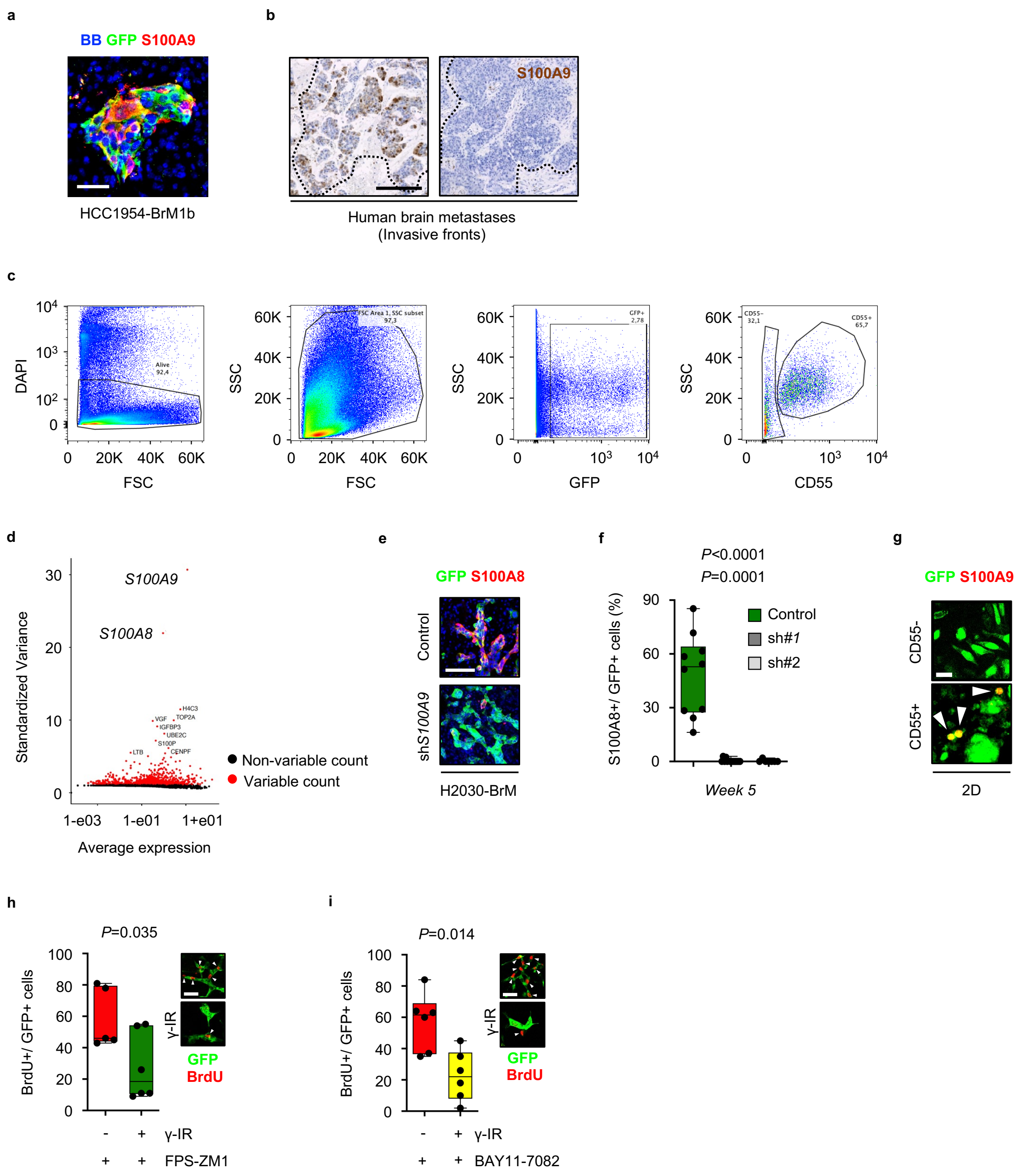

S100A9-mediated radioresistance is linked to cancer stem cell properties and sensitivity to RAGE and NF-κB inhibition. a, Representative image of a brain metastasis generated by HCC1954-BrMb. Scale bar: 50 µm. This experiment was repeated three times with similar results. **b**, Representative images of invasive fronts from human brain metastases (dotted lines). Scale bar: 200 µm. This experiment was performed once using the available samples with invasive fronts. **c**,

Gating strategy for fluorescence activated cell sorting (FACS) of CD55- and CD55+ cells from H2030-BrM brain metastases. **d**, Graph shows a subset of genes that exhibit high cell-to-cell variation in the dataset

obtained after modelling the mean-variance relationship inherent in single-cell data with Seurat[21]. **e**, Representative images of H2030-BrM Control and sh*S100A9* brain metastases. Scale bar: 100 µm. **f**, Quantification of the percentage of S100A8+ GFP+ positive cells in irradiated brain metastatic lesions *in vivo,* generated from intracardiac injection of H2030-BrM Control, H2030-BrM shS*100A9#1* or H2030-BrM sh*S100A9#2* cells. Values are shown in box-and-whisker plots where every dot represents a metastatic lesion and the line in the box corresponds to the median. The boxes go from the upper to the lower quartiles and the whiskers go from the minimum to the maximum value (n=10 FOV, 2 brains, H2030-BrM Control + γ-IR; n=14 FOV, 3 brains, H2030-BrM sh*S100A9*#1 + γ-IR; n=7 FOV, 2 brains, H2030-BrM sh*S100A9*#2 + γ-IR). *P* value was calculated using two-tailed Mann-Whitney Test. **g**, Representative immunofluorescent images of sorted CD55- and CD55+ cells grown *in vitro.* Scale bar: 25 µm. This experiment was performed three times with similar results. **h**, Representative images and quantification of the mean percentage of BrdU+ cells per GFP+ cells in brain organotypic cultures with H2030-BrM after treatment with 10 µM of FPS-ZM1 and 10Gy irradiation or no irradiation. Values are shown in box-and-whisker plots where every dot represents an independent culture and the line in the box corresponds to the median. The boxes go from the upper to the lower quartiles and the whiskers go from the minimum to the maximum value (n=5, non-irradiated H2030-BrM; n=6 irradiated H2030-BrM, both treated with FPS-ZM1). *P* value is calculated using two-tailed t test. Scale bar: 50 µm. **i**, Representative images and quantification of the mean percentage of BrdU+ cells per GFP+ cells in brain organotypic cultures with H2030-BrM after treatment with 50 µM of BAY-117081 and 10Gy irradiation or no irradiation. Values are shown in box-and-whisker plots where every dot represents an independent culture and the line in the box corresponds to the median. The boxes go from the upper to the lower quartiles and the whiskers go from the minimum to the maximum value (n=6, non-irradiated H2030-BrM; n=6, irradiated H2030-BrM, both treated with BAY-117081). *P* value is calculated using two-tailed t test. Scale bar: 50 µm.

Supplementary Figure 5.

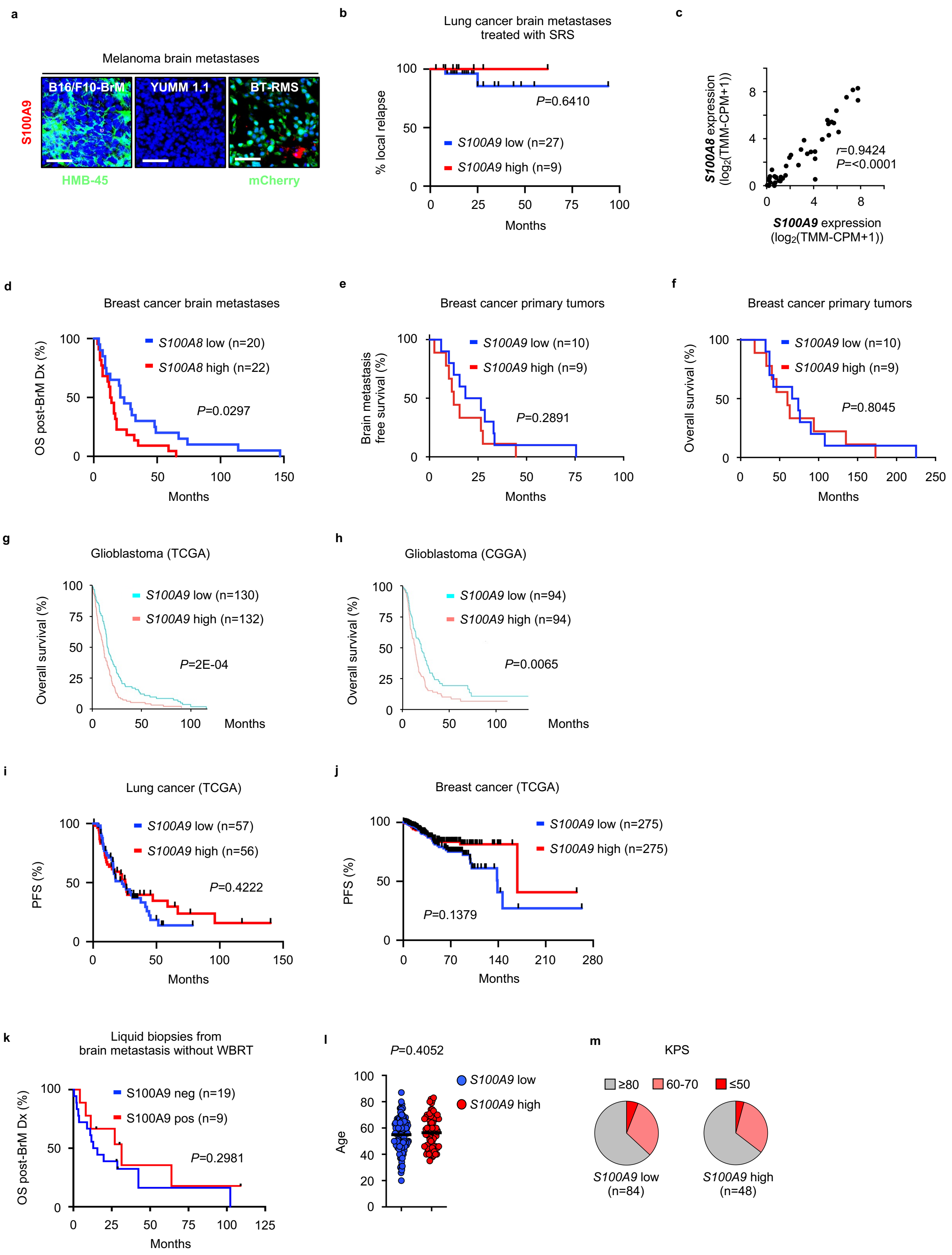

S100A9 is a brain metastasis biomarker of therapeutic response to WBRT. a, Representative images of immunofluorescent staining for S100A9 in brain metastases from three different murine brain metastatic melanoma models (B16/F10-BrM, YUMM1.1, BT-RMS). Scale bars: 50 µm. The experiment was performed three times for each model with similar results. **b**, Analysis of time to relapse in a cohort of 36 lung cancer brain metastasis patients treated with stereotactic radiosurgery (SRS). Data is shown as a Kaplan-Meier plot and two groups of patients (S100A9 low/high) were delineated by using 5% of S100A9 immunohistochemical staining positivity as a cut-off. *P* value was calculated using log rank (Mantel-Cox) test. **c**, Correlation of *S100A9* and *S100A8* gene expression in each respective patient from a cohort of 42 breast cancer brain metastases. Pearson R correlation coefficient and corresponding *P* value were calculated. **d**, Analysis of survival post-brain metastasis diagnosis in a cohort of 42 breast cancer brain metastasis patients. Only patients that received radiotherapy were included. Data is shown as a Kaplan-Meier plot and two groups of patients (S100A8 low/high) were delineated according to their *S100A8* mRNA expression levels in brain metastasis. *P* value was calculated using log rank (Mantel-Cox) test. **e**, Analysis of brain metastasis-free survival from primary tumor diagnosis in a previously published cohort of 19 breast cancer brain metastasis patients. Data is shown as a Kaplan-Meier plot and two groups of patients (S100A9 low/high) were delineated according to their *S100A9* mRNA expression levels in brain metastasis by using the median of *S100A9* expression as a cut-off. *P* value was calculated using log rank (Mantel-Cox) test. **f**, Analysis of overall survival post-primary tumor diagnosis in a previously published cohort of 19 breast cancer brain metastasis patients. Data is shown as a Kaplan-Meier plot and two groups of patients (S100A9 low/high) were delineated according to their *S100A9* mRNA expression levels in brain metastasis by using the median of *S100A9* expression as a cut-off. *P* value was calculated using log rank (Mantel-Cox) test. **g**, **h**, Analysis of overall survival post-primary tumor diagnosis in the glioblastoma TCGA (**g**) and CGGA (**h**) cohorts treated with the standard-of-care, which includes neurosurgery, radiotherapy and temozolomide [42]. Data is shown as a Kaplan-Meier plot and two groups of patients (S100A9 low/high) were delineated according to their *S100A9* mRNA expression levels in brain metastasis by using the 1st and 4th quartile of *S100A9* expression as a cut-off. *P* value was calculated using log rank (Mantel-Cox) test. **i**, **j**, Analysis of progression-free survival in the TCGA lung cancer (**i**) and breast cancer (**j**) cohort. Only patients that were treated with radiotherapy were included. Data is shown as Kaplan-Meier plots and two groups of patients (S100A9 low/high) were

delineated according to their S100A9 mRNA expression levels in primary lung and breast tumors using the respective median expression as cut-off. *P* value was calculated using log rank (Mantel-Cox) test. **k**, Analysis of survival post-brain metastasis diagnosis in a combined cohort of lung cancer brain metastasis patients (3 cases) and breast cancer brain metastasis patients (25 cases) that did not receive WBRT. Data is shown as a Kaplan-Meier plot and two groups of patients (S100A9 negative/positive) were delineated by their S100A9 positivity in serum samples. *P* value was calculated using log rank (Mantel-Cox) test. **l**, Analysis of patient age at neurosurgery/WBRT of 73 S100A9 high and 128 S100A9 low patients from all cohorts of brain metastasis patients used in this study where age was available (Supplementary Tables 13, 15, 16, 17 and 21). Values are shown in dot plots where each dot represents a patient and the line corresponds to the median. *P* value was calculated using two-tailed t-test. **m**, Analysis of KPS score at neurosurgery/WBRT of S100A9 48 high and 84 S100A9 low patients from all cohorts of brain metastasis patients used in this study where KPS was available (Supplementary Tables 13, 15, 17 and 21). Values are shown in pie charts representing percentages of each respective KPS score in either S100A9 high or low patients. For S100A9 high, 2 patients had a KPS score <50, 15 patients had a score 60-70 and 31 patients had a score >80. For S100A9 low patients, 5 patients had a KPS score <50, 26 patients had a score of 60-70 and 51 patients had a score >80.

Supplementary Figure 6.

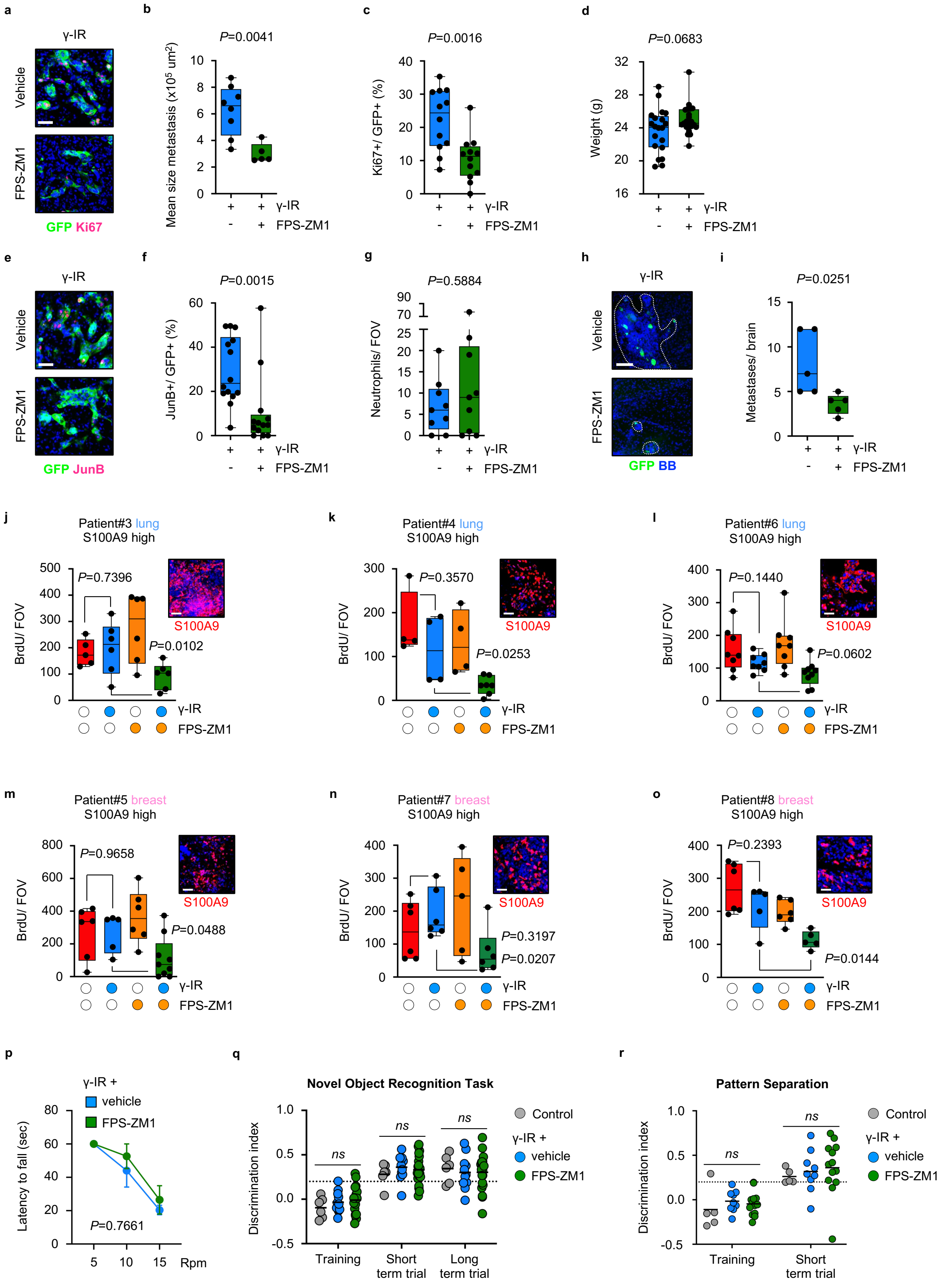

FPS-ZM1 radiosensitizes experimental and human brain metastases. a, Representative images of brain metastases at the experimental endpoint as described in (Fig. 6a). Scale bar: 50 µm. **b**, Quantification of H2030-BrM metastasis mean size in mice treated with WBRT and vehicle or FPS-ZM1 as indicated in (Fig.6a). Values are shown in box-and-whisker plots where every dot represents a brain and the line in the box corresponds to the median. The boxes go from the upper to the lower quartiles and the whiskers go from the minimum to the maximum value (n=8, Vehicle + γ-IR; n=5, FPS-ZM1 + γ-IR). *P* value was calculated using two-tailed t-test. **c**, Quantification of proliferation using Ki67 in H2030-BrM metastases from mice treated with WBRT and vehicle or FPS-ZM1 as indicated in (Fig. 6a). Values are shown in box-and-whisker plots where every dot represents a metastatic lesion obtained from 4 brains in each experimental condition and the line in the box corresponds to the median. The boxes go from the upper to the lower quartiles and the whiskers go from the minimum to the maximum value (n=12, Vehicle + γ-IR; n=12, FPS-ZM1 + γ-IR). *P* value was calculated using two-tailed t-test. **d**, Quantification of mouse weight at the end of the experiment as indicated in (Fig. 6a) comparing mice treated with WBRT and vehicle or FPS-ZM1. Values are shown in box-and-whisker plots where every dot represents a mouse and the line in the box corresponds to the median. The boxes go from the upper to the lower quartiles and the whiskers go from the minimum to the maximum value (n=20, Vehicle + γ-IR; n=21, FPS-ZM1 + γ-IR). *P* value was calculated using two-tailed t-test. **e**, Representative images of brain metastases at the experimental endpoint as described in (Fig. 6a) stained for JunB. Scale bar: 50 µm. **f**, Quantification of JunB+ GFP+ H2030-BrM cells in mice treated with WBRT and vehicle or FPS-ZM1 as indicated in (Fig. 6a). Values are shown in box-and-whisker plots where every dot represents a metastatic lesion obtained from 4 brains in each experimental condition and the line in the box corresponds to the median. The boxes go from the upper to the lower quartiles and the whiskers go from the minimum to the maximum value (n=14 metastases from 4 mice, Vehicle + γ-IR; n=13 metastases from 4 mice, FPS-ZM1 + γ-IR). *P* value was calculated using two-tailed Mann-Whitney Test. **g**, Quantification of neutrophils per FOV in H2030-BrM brain metastasis from mice treated with either WBRT and vehicle or FPS-ZM1*,* using the NIMP-R14 staining. For both conditions equal numbers of lesions of different sizes were chosen. Values are shown in box-and-whisker plots where every dot represents a metastatic lesion and the line in the box corresponds to the median. The boxes go from the upper to the lower quartiles and the whiskers go from minimum to maximum value (n=9 FOV, each experimental condition). *P* value was

calculated using two-tailed t test. **h**, Representative images of brain metastases at the experimental endpoint as described in (Extended Data Fig. 5a). Scale bar: 75 µm. **i**, Quantification of E0771-BrM metastases in mice treated with WBRT and vehicle or FPS-ZM1 as indicated in (Extended Data Fig. 5a). Values are shown in box-and-whisker plots where every dot represents a mouse and the line in the box corresponds to the median. The boxes go from the upper to the lower quartiles and the whiskers go from the minimum to the maximum value (n=5, Vehicle + γ-IR; n=5, FPS-ZM1 + γ-IR). *P* value was calculated using two-tailed t-test. **j**-**o**, Quantification of BrdU+ cancer cells in PDOC treated with FPS-ZM1 and/or γ-IR obtained from patient#3 (**j**), patient#4 (**k**), patient#5 (**m**), patient#6 (**l**), patient#7 (**n**) and patient#8 (**o**). Values are shown in box-and-whisker plots where every dot represents a FOV (4-9 FOV per experimental condition obtained from 4-6 organotypic cultures) and the line in the box corresponds to the median. The boxes go from the upper to the lower quartiles and the whiskers go from the minimum to the maximum value. *P* value was calculated using two-tailed t-test. A representative image in each PDOC show S100A9 levels. Scale bar: 50 µm. **p**, Quantification of latency to fall at three different constant speeds of mice treated with WBRT and either vehicle or FPS-ZM1. Values shown are mean ± SEM (n=5 mice, each experimental condition). *P* value was calculated using two-tailed t-test. **q**, Quantification of discrimination index comparing different groups when trained and when short term and long-term memories were evaluated. Values are shown in a scatter dot plot where every dot represents a mouse and the line corresponds to the mean (n=6, Control; n=13, Vehicle + γ-IR, n=20, FPS-ZM1 + γ-IR). Calculation of *P* values are detailed in Supplementary Table 24. **r**, Quantification of discrimination index comparing different groups when trained and when short term memory was evaluated. Values are shown in a scatter dot plot where every dot represents a mouse and the line corresponds to the mean (n=6, Control; n=13, Vehicle + γ-IR, n=20, FPS-ZM1 + γ-IR). Calculation of *P* values are detailed in Supplementary Table 24.

Supplementary Tables.

Supplementary Table 1.

Supplementary Table 1_Enrichment of a stem cell signature in H2030-BrM in brain organotypic cultures_Corresponds to Fig.1M

Wong et al_PMID: 18397753_WONG_ADULT_TISSUE_STEM_MODULE
H2030-BrM in brain slices vs H2030-BrM in vitro

NAME	PROBE	GENE SYMBOL	GENE_TITLE	RANK IN GENE LIST	RANK METRIC SCORE	RUNNING ES	CORE ENRICHMENT
row_0	S100A4	S100A4	S100 calcium binding protein A4	3	2.923417807	0.00140216	Yes
row_1	GBP2	GBP2	guanylate binding protein 2, interferon-inducible	11	2.189227104	0.00267786	Yes
row_2	NREP	null	null	43	1.613750696	0.00319478	Yes
row_3	TNC	TNC	tenascin C (hexabrachion)	48	1.571207762	0.00456532	Yes
row_4	IL1R1	IL1R1	interleukin 1 receptor, type I	63	1.419473529	0.00561971	Yes
row_5	PKIA	PKIA	protein kinase (cAMP-dependent, catalytic) inhibitor alpha	81	1.363300323	0.00657925	Yes
row_6	LGALS3BP	LGALS3BP	lectin, galactoside-binding, soluble, 3 binding protein	104	1.289586067	0.00738072	Yes
row_7	DAPP1	DAPP1	dual adaptor of phosphotyrosine and 3-phosphoinositides	119	1.265930533	0.00843511	Yes
row_8	KLF2	KLF2	Kruppel-like factor 2 (lung)	147	1.198424339	0.00907849	Yes
row_9	CEBPB	CEBPB	CCAAT/enhancer binding protein (C/EBP), beta	153	1.183876157	0.01041742	Yes
row_10	CEBPD	CEBPD	CCAAT/enhancer binding protein (C/EBP), delta	204	1.115877748	0.01033365	Yes
row_11	ABHD8	ABHD8	abhydrolase domain containing 8	208	1.108138919	0.01173581	Yes
row_12	NKX3-1	NKX3-1	NK3 transcription factor related, locus 1 (Drosophila)	214	1.104024768	0.01307474	Yes
row_13	TRIB3	TRIB3	tribbles homolog 3 (Drosophila)	247	1.059237599	0.01356005	Yes
row_14	TPD52L1	TPD52L1	tumor protein D52-like 1	257	1.042314291	0.01477251	Yes
row_15	RHOU	RHOU	ras homolog gene family, member U	261	1.039263844	0.01617467	Yes
row_16	TTC3	TTC3	tetratricopeptide repeat domain 3	278	1.016176224	0.01716583	Yes
row_17	AK1	AK1	adenylate kinase 1	283	1.013177991	0.01853637	Yes
row_18	LYSMD3	LYSMD3	LysM, putative peptidoglycan-binding, domain containing 3	297	0.999990106	0.01962237	Yes
row_19	RHOB	RHOB	ras homolog gene family, member B	303	0.991709411	0.0209613	Yes
row_20	SH3GLB1	SH3GLB1	SH3-domain GRB2-like endophilin B1	321	0.977687776	0.02192085	Yes
row_21	SPARC	SPARC	secreted protein, acidic, cysteine-rich (osteonectin)	332	0.966426075	0.0231017	Yes
row_22	JUND	JUND	jun D proto-oncogene	338	0.960232317	0.02444062	Yes
row_23	SLC1A1	SLC1A1	solute carrier family 1 (neuronal/epithelial high affinity glutama	342	0.959551215	0.02584278	Yes
row_24	S100A6	S100A6	S100 calcium binding protein A6	348	0.957313418	0.02718171	Yes
row_25	SCN1B	SCN1B	sodium channel, voltage-gated, type I, beta	444	0.891262412	0.02567524	Yes
row_26	TSC22D1	TSC22D1	TSC22 domain family, member 1	477	0.87331748	0.02616055	Yes
row_27	NR4A2	NR4A2	nuclear receptor subfamily 4, group A, member 2	533	0.845843732	0.0259187	Yes
row_28	C1R	C1R	complement component 1, r subcomponent	555	0.834768236	0.02675178	Yes
row_29	PMEPA1	null	null	573	0.828114748	0.02771132	Yes
row_30	IGFBP5	IGFBP5	insulin-like growth factor binding protein 5	621	0.809343934	0.02772239	Yes
row_31	CERS4	null	null	657	0.794150531	0.02811286	Yes
row_32	CREBRF	null	null	659	0.793830037	0.02957825	Yes
row_33	ICK	ICK	intestinal cell (MAK-like) kinase	686	0.783892095	0.03025325	Yes
row_34	GLIS2	GLIS2	GLIS family zinc finger 2	695	0.780984998	0.03149733	Yes
row_35	KLF9	KLF9	Kruppel-like factor 9	700	0.779268622	0.03286787	Yes
row_36	CXXC5	CXXC5	CXXC finger 5	731	0.76964426	0.03341641	Yes
row_37	PBXIP1	PBXIP1	pre-B-cell leukemia transcription factor interacting protein 1	735	0.768651962	0.03481857	Yes
row_38	FOXC1	FOXC1	forkhead box C1	804	0.747692645	0.03416572	Yes
row_39	PNRC1	PNRC1	proline-rich nuclear receptor coactivator 1	849	0.733122766	0.03427164	Yes
row_40	LTBP3	LTBP3	latent transforming growth factor beta binding protein 3	857	0.731958091	0.03554734	Yes
row_41	HOXC4	HOXC4	homeobox C4	902	0.717389166	0.03565326	Yes
row_42	SASH1	SASH1	SAM and SH3 domain containing 1	911	0.714997649	0.03689734	Yes
row_43	IER3	IER3	immediate early response 3	944	0.704852343	0.03738265	Yes
row_44	MARCKSL1	MARCKSL1	MARCKS-like 1	994	0.692952216	0.03733049	Yes
row_45	YES1	YES1	v-yes-1 Yamaguchi sarcoma viral oncogene homolog 1	1000	0.69175458	0.03866942	Yes
row_46	PXDN	PXDN	peroxidasin homolog (Drosophila)	1009	0.69023788	0.0399135	Yes
row_47	RCAN3	null	null	1025	0.686759531	0.04093628	Yes
row_48	GABARAPL1	GABARAPL1	GABA(A) receptor-associated protein like 1	1035	0.684823453	0.04214874	Yes
row_49	RC3H2	null	null	1049	0.682400882	0.04323475	Yes
row_50	SOX4	SOX4	SRY (sex determining region Y)-box 4	1066	0.677369833	0.0442259	Yes
row_51	CYP4V2	CYP4V2	cytochrome P450, family 4, subfamily V, polypeptide 2	1068	0.677023768	0.04569129	Yes
row_52	MSI2	MSI2	musashi homolog 2 (Drosophila)	1110	0.665554702	0.04589206	Yes
row_53	EVL	EVL	Enah/Vasp-like	1162	0.65386188	0.04577667	Yes
row_54	ABL1	ABL1	v-abl Abelson murine leukemia viral oncogene homolog 1	1227	0.640559912	0.04525029	Yes
row_55	TESK1	TESK1	testis-specific kinase 1	1235	0.639185607	0.04652598	Yes
row_56	PBX1	PBX1	pre-B-cell leukemia transcription factor 1	1241	0.638397038	0.04786491	Yes
row_57	MLLT3	MLLT3	myeloid/lymphoid or mixed-lineage leukemia (trithorax homol	1256	0.636099577	0.0489193	Yes
row_58	PHLDA2	PHLDA2	pleckstrin homology-like domain, family A, member 2	1271	0.633083165	0.04997369	Yes
row_59	IMPACT	IMPACT	Impact homolog (mouse)	1287	0.630615652	0.05099646	Yes
row_60	KLF4	KLF4	Kruppel-like factor 4 (gut)	1307	0.625315189	0.05189277	Yes
row_61	NDRG1	NDRG1	N-myc downstream regulated gene 1	1370	0.613167107	0.05142961	Yes
row_62	LPIN1	LPIN1	lipin 1	1466	0.594115734	0.04992314	Yes
row_63	S100A13	S100A13	S100 calcium binding protein A13	1513	0.585552752	0.04996583	Yes
row_64	FHL1	FHL1	four and a half LIM domains 1	1532	0.583661735	0.05089376	Yes
row_65	IL15	IL15	interleukin 15	1541	0.58239162	0.05213784	Yes
row_66	COL18A1	COL18A1	collagen, type XVIII, alpha 1	1548	0.581638277	0.05344515	Yes
row_67	PLEKHA5	PLEKHA5	pleckstrin homology domain containing, family A member 5	1562	0.578913569	0.05453115	Yes
row_68	PHLDA1	PHLDA1	pleckstrin homology-like domain, family A, member 1	1590	0.574503362	0.05517454	Yes
row_69	PDGFA	PDGFA	platelet-derived growth factor alpha polypeptide	1644	0.564889848	0.05499592	Yes
row_70	PKD2	PKD2	polycystic kidney disease 2 (autosomal dominant)	1654	0.563801885	0.05620839	Yes
row_71	HOXA5	HOXA5	homeobox A5	1665	0.561781466	0.05738924	Yes
row_72	TMEM176B	TMEM176B	transmembrane protein 176B	1691	0.558565676	0.05809585	Yes
row_73	NPTXR	NPTXR	neuronal pentraxin receptor	1699	0.557738423	0.05937155	Yes
row_74	SORCS2	SORCS2	sortilin-related VPS10 domain containing receptor 2	1700	0.557206631	0.06086856	Yes
row_75	RORA	RORA	RAR-related orphan receptor A	1702	0.556940556	0.06233395	Yes
row_76	HSPA1B	HSPA1B	heat shock 70kDa protein 1B	1709	0.55580765	0.06364127	Yes
row_77	TGIF2	TGIF2	TGFB-induced factor 2 (TALE family homeobox)	1724	0.554234385	0.06469565	Yes
row_78	DLC1	DLC1	deleted in liver cancer 1	1726	0.554157436	0.06616104	Yes
row_79	PDE8A	PDE8A	phosphodiesterase 8A	1765	0.547209918	0.06645665	Yes
row_80	TAGLN3	TAGLN3	transgelin 3	1773	0.546197951	0.06773235	Yes
row_81	NUPR1	NUPR1	-	1824	0.537502706	0.06764858	Yes
row_82	VDR	VDR	vitamin D (1,25- dihydroxyvitamin D3) receptor	1856	0.533230245	0.0681655	Yes
row_83	CAVIN2	null	null	1862	0.532542825	0.06950443	Yes
row_84	CITED2	CITED2	Cbp/p300-interacting transactivator, with Glu/Asp-rich carbox	1870	0.531621337	0.07078013	Yes
row_85	SESN1	SESN1	sestrin 1	1903	0.52711302	0.07126544	Yes
row_86	SPON1	SPON1	spondin 1, extracellular matrix protein	1905	0.526946545	0.07273083	Yes
row_87	ZMYM4	ZMYM4	zinc finger, MYM-type 4	1930	0.524229586	0.07346906	Yes
row_88	PHC1	PHC1	polyhomeotic homolog 1 (Drosophila)	1933	0.523659348	0.07490283	Yes
row_89	PHLDB2	PHLDB2	pleckstrin homology-like domain, family B, member 2	1946	0.522244751	0.07602046	Yes
row_90	MEF2A	MEF2A	MADS box transcription enhancer factor 2, polypeptide A (myoc	1950	0.520993769	0.07742261	Yes
row_91	SYNPO	SYNPO	synaptopodin	1957	0.520538867	0.07872993	Yes
row_92	CDKN1A	CDKN1A	cyclin-dependent kinase inhibitor 1A (p21, Cip1)	1995	0.515774846	0.07905716	Yes
row_93	ERRFI1	ERRFI1	ERBB receptor feedback inhibitor 1	2014	0.513124406	0.07998508	Yes
row_94	JUNB	JUNB	jun B proto-oncogene	2029	0.510417581	0.08103947	Yes
row_95	KMT2E	null	null	2047	0.508265853	0.08199901	Yes
row_96	DNAJB4	DNAJB4	DnaJ (Hsp40) homolog, subfamily B, member 4	2097	0.502417624	0.08194686	Yes
row_97	ISG15	ISG15	ISG15 ubiquitin-like modifier	2175	0.491102904	0.08100946	Yes
row_98	ARL3	ARL3	ADP-ribosylation factor-like 3	2192	0.489093304	0.08200062	Yes
row_99	NRIP1	NRIP1	nuclear receptor interacting protein 1	2228	0.484845936	0.08239108	Yes
row_100	DNAJB2	DNAJB2	DnaJ (Hsp40) homolog, subfamily B, member 2	2273	0.479141891	0.08249701	Yes
row_101	CCL5	CCL5	chemokine (C-C motif) ligand 5	2287	0.477191895	0.08358301	Yes
row_102	HLA-DMA	HLA-DMA	major histocompatibility complex, class II, DM alpha	2301	0.474969953	0.08466902	Yes
row_103	PPP1R18	null	null	2327	0.471020758	0.08537563	Yes
row_104	IRF6	IRF6	interferon regulatory factor 6	2377	0.466208905	0.08532348	Yes
row_105	PRKCB	null	null	2403	0.463312775	0.08603009	Yes
row_106	GNB5	GNB5	guanine nucleotide binding protein (G protein), beta 5	2409	0.462656498	0.08736902	Yes
row_107	PQLC3	PQLC3	PQ loop repeat containing 3	2437	0.459688634	0.08801241	Yes
row_108	SENP6	SENP6	SUMO1/sentrin specific peptidase 6	2469	0.456705689	0.08852933	Yes
row_109	BEX4	null	null	2479	0.455656588	0.0897418	Yes
row_110	LRRC8A	LRRC8A	leucine rich repeat containing 8 family, member A	2531	0.451029152	0.08962641	Yes
row_111	CDKN1C	CDKN1C	cyclin-dependent kinase inhibitor 1C (p57, Kip2)	2533	0.450862795	0.0910918	Yes
row_112	ELL2	ELL2	elongation factor, RNA polymerase II, 2	2540	0.450018436	0.09239911	Yes
row_113	LMO2	LMO2	LIM domain only 2 (rhombotin-like 1)	2549	0.44924137	0.0936432	Yes
row_114	ACTR3B	ACTR3B	ARP3 actin-related protein 3 homolog B (yeast)	2628	0.440292478	0.09267419	Yes
row_115	ITGAE	ITGAE	integrin, alpha E (antigen CD103, human mucosal lymphocyte a	2652	0.438036323	0.09344403	Yes
row_116	FOS	FOS	v-fos FBJ murine osteosarcoma viral oncogene homolog	2654	0.437566936	0.09490942	Yes
row_117	ARPC1B	ARPC1B	actin related protein 2/3 complex, subunit 1B, 41kDa	2681	0.434596211	0.09558443	Yes
row_118	ADARB1	ADARB1	adenosine deaminase, RNA-specific, B1 (RED1 homolog rat)	2707	0.431386918	0.09629104	Yes
row_119	MPZL1	MPZL1	myelin protein zero-like 1	2711	0.4310233	0.09769321	Yes
row_120	RHOQ	RHOQ	ras homolog gene family, member Q	2721	0.429480225	0.09890567	Yes
row_121	IGFBP7	IGFBP7	insulin-like growth factor binding protein 7	2744	0.427407473	0.09970713	Yes
row_122	GSTK1	GSTK1	glutathione S-transferase kappa 1	2773	0.424469858	0.1003189	Yes
row_123	ZFP1	ZFP1	zinc finger protein 1 homolog (mouse)	2855	0.416058123	0.09925505	Yes
row_124	DBP	DBP	D site of albumin promoter (albumin D-box) binding protein	2874	0.413998216	0.10018297	Yes
row_125	BACH1	BACH1	BTB and CNC homology 1, basic leucine zipper transcription fac	2910	0.410308868	0.10057344	Yes
row_126	WDR78	WDR78	WD repeat domain 78	2914	0.40996477	0.1019756	Yes
row_127	FYN	FYN	FYN oncogene related to SRC, FGR, YES	2986	0.401499957	0.10122789	Yes
row_128	PLEK	PLEK	pleckstrin	3078	0.394426793	0.09984789	Yes
row_129	REEP3	REEP3	receptor accessory protein 3	3100	0.392643571	0.10068097	Yes
row_130	GCH1	GCH1	GTP cyclohydrolase 1 (dopa-responsive dystonia)	3144	0.387548059	0.10081851	Yes
row_131	ZFPM1	ZFPM1	zinc finger protein, multitype 1	3172	0.385518312	0.10146189	Yes
row_132	RIDA	null	null	3214	0.382253975	0.10166266	Yes
row_133	CLK1	CLK1	CDC-like kinase 1	3251	0.378025681	0.10202151	Yes
row_134	ZKSCAN1	ZKSCAN1	zinc finger with KRAB and SCAN domains 1	3274	0.376138538	0.10282297	Yes
row_135	PTGER4	PTGER4	prostaglandin E receptor 4 (subtype EP4)	3297	0.373867482	0.10362443	Yes
row_136	DAB2	DAB2	disabled homolog 2, mitogen-responsive phosphoprotein (Dros	3298	0.373824269	0.10512144	Yes
row_137	COL6A1	COL6A1	collagen, type VI, alpha 1	3355	0.369072229	0.10484798	Yes
row_138	ASTN1	ASTN1	astrotactin 1	3375	0.368160546	0.10574429	Yes
row_139	ZMYND11	ZMYND11	zinc finger, MYND domain containing 11	3396	0.366370767	0.10660898	Yes
row_140	REST	REST	RE1-silencing transcription factor	3407	0.365094125	0.10778983	Yes
row_141	FOXA1	FOXA1	forkhead box A1	3452	0.361526132	0.10789575	Yes
row_142	TRIM32	TRIM32	tripartite motif-containing 32	3543	0.354135871	0.10654736	Yes
row_143	DBNDD2	DBNDD2	dysbindin (dystrobrevin binding protein 1) domain containing 2	3563	0.352644831	0.10744367	Yes
row_144	DUSP4	DUSP4	dual specificity phosphatase 4	3566	0.352443099	0.10887744	Yes
row_145	MDFIC	MDFIC	MyoD family inhibitor domain containing	3601	0.349761009	0.10929952	Yes
row_146	SKI	SKI	v-ski sarcoma viral oncogene homolog (avian)	3602	0.349706203	0.11079653	Yes
row_147	FLT3	FLT3	fms-related tyrosine kinase 3	3609	0.34948349	0.11210384	Yes
row_148	ANTXR2	ANTXR2	anthrax toxin receptor 2	3685	0.343358696	0.11122968	Yes
row_149	TP53I11	TP53I11	tumor protein p53 inducible protein 11	3731	0.338326693	0.11130399	Yes
row_150	STMN3	STMN3	stathmin-like 3	3765	0.334823161	0.11175767	Yes
row_151	SOCS3	SOCS3	suppressor of cytokine signaling 3	3807	0.332044631	0.11195844	Yes
row_152	CBFA2T3	CBFA2T3	core-binding factor, runt domain, alpha subunit 2; translocated	3815	0.331637859	0.11323414	Yes
row_153	LGALSL	null	null	3820	0.331238538	0.11460468	Yes
row_154	DDIT4	DDIT4	DNA-damage-inducible transcript 4	3895	0.324400008	0.11376214	Yes
row_155	BARX2	BARX2	BarH-like homeobox 2	3955	0.320506155	0.11339383	Yes
row_156	NCAM1	NCAM1	neural cell adhesion molecule 1	3982	0.318857342	0.11406883	Yes
row_157	ZBTB20	ZBTB20	zinc finger and BTB domain containing 20	3988	0.318397701	0.11540776	Yes
row_158	SARAF	null	null	4051	0.313290268	0.1149446	Yes
row_159	GRIA3	GRIA3	glutamate receptor, ionotrophic, AMPA 3	4127	0.308445692	0.11407044	Yes
row_160	SCG5	SCG5	secretogranin V (7B2 protein)	4236	0.300506175	0.11215296	Yes
row_161	INVS	INVS	inversin	4316	0.296157509	0.11115234	Yes
row_162	KRBA1	KRBA1	KRAB-A domain containing 1	4332	0.294687569	0.11217511	Yes
row_163	CBX4	CBX4	chromobox homolog 4 (Pc class homolog, Drosophila)	4388	0.290536433	0.11193327	Yes
row_164	LTBP2	LTBP2	latent transforming growth factor beta binding protein 2	4410	0.288968593	0.11276635	Yes
row_165	ANKH	ANKH	ankylosis, progressive homolog (mouse)	4438	0.287340641	0.11340973	Yes
row_166	ITSN1	ITSN1	intersectin 1 (SH3 domain protein)	4449	0.285963267	0.11459058	Yes
row_167	ENTPD1	ENTPD1	ectonucleoside triphosphate diphosphohydrolase 1	4479	0.284385979	0.11517073	Yes
row_168	STAT4	STAT4	signal transducer and activator of transcription 4	4491	0.284057707	0.11631997	Yes
row_169	SSBP2	SSBP2	single-stranded DNA binding protein 2	4505	0.283408999	0.11740597	Yes
row_170	DNAJB9	DNAJB9	DnaJ (Hsp40) homolog, subfamily B, member 9	4527	0.281771451	0.11823905	Yes
row_171	TTPA	TTPA	tocopherol (alpha) transfer protein (ataxia (Friedreich-like) with	4545	0.280426443	0.1191986	Yes
row_172	PEX11A	PEX11A	peroxisomal biogenesis factor 11A	4564	0.279491842	0.12012652	Yes
row_173	ZFP90	ZFP90	zinc finger protein 90 homolog (mouse)	4647	0.273997128	0.11903105	Yes
row_174	PURA	PURA	purine-rich element binding protein A	4674	0.272267073	0.11970606	Yes
row_175	PFN2	PFN2	profilin 2	4677	0.27211231	0.12113983	Yes
row_176	UGDH	UGDH	UDP-glucose dehydrogenase	4714	0.269808888	0.12149867	Yes
row_177	THSD1	THSD1	thrombospondin, type I, domain containing 1	4816	0.262908429	0.11980251	Yes
row_178	MAGED1	MAGED1	melanoma antigen family D, 1	4883	0.25773403	0.11921289	Yes
row_179	GEM	GEM	GTP binding protein overexpressed in skeletal muscle	4909	0.256129831	0.11991951	Yes
row_180	RETREG1	null	null	4938	0.254068881	0.12053128	Yes
row_181	FN1	FN1	fibronectin 1	5010	0.249131665	0.11978358	Yes
row_182	TP53BP1	TP53BP1	tumor protein p53 binding protein, 1	5020	0.248703137	0.12099604	Yes
row_183	NFIX	NFIX	nuclear factor I/X (CCAAT-binding transcription factor)	5036	0.247484893	0.12201881	Yes
row_184	KLRG1	KLRG1	killer cell lectin-like receptor subfamily G, member 1	5144	0.241531044	0.12013296	Yes
row_185	PTOV1	PTOV1	prostate tumor overexpressed gene 1	5178	0.239575043	0.12058665	Yes
row_186	NR2F1	NR2F1	nuclear receptor subfamily 2, group F, member 1	5183	0.238977745	0.1219572	Yes
row_187	NDRG2	NDRG2	NDRG family member 2	5201	0.238086656	0.12291674	Yes
row_188	NIPSNAP1	NIPSNAP1	nipsnap homolog 1 (C. elegans)	5243	0.236113235	0.12311751	Yes
row_189	ITM2C	ITM2C	integral membrane protein 2C	5257	0.235362649	0.12420351	Yes
row_190	TBX1	TBX1	T-box 1	5328	0.232194677	0.12348743	Yes
row_191	GRIA2	GRIA2	glutamate receptor, ionotropic, AMPA 2	5337	0.231619433	0.1247315	Yes
row_192	P3H4	null	null	5356	0.230774581	0.12565944	Yes
row_193	PDLIM3	PDLIM3	PDZ and LIM domain 3	5367	0.230056003	0.12684028	Yes
row_194	NFIB	NFIB	nuclear factor I/B	5368	0.229838893	0.1283373	Yes
row_195	P2RX4	P2RX4	purinergic receptor P2X, ligand-gated ion channel, 4	5424	0.226787686	0.12809543	Yes
row_196	RAB27A	RAB27A	RAB27A, member RAS oncogene family	5482	0.223234564	0.12779036	Yes
row_197	DCUN1D1	DCUN1D1	DCN1, defective in cullin neddylation 1, domain containing 1 (S	5515	0.221255645	0.12827566	Yes
row_198	RASL10A	null	null	5521	0.220959768	0.12961459	Yes
row_199	PTPN12	PTPN12	protein tyrosine phosphatase, non-receptor type 12	5532	0.220367	0.13079545	Yes
row_200	CD59	CD59	CD59 molecule, complement regulatory protein	5550	0.219372809	0.131755	Yes
row_201	TCEAL9	null	null	5598	0.216602489	0.13176607	Yes
row_202	ABCA1	ABCA1	ATP-binding cassette, sub-family A (ABC1), member 1	5623	0.215618521	0.1325043	Yes
row_203	CMTM8	CMTM8	CKLF-like MARVEL transmembrane domain containing 8	5802	0.206830248	0.12837373	Yes
row_204	CNN3	CNN3	calponin 3, acidic	5804	0.206765473	0.12983912	Yes
row_205	BAG3	BAG3	BCL2-associated athanogene 3	5944	0.200570717	0.12694156	Yes
row_206	NRK	NRK	Nik related kinase	5974	0.199645191	0.12752172	Yes
row_207	ICAM1	ICAM1	intercellular adhesion molecule 1 (CD54), human rhinovirus rec	5981	0.199461594	0.12882903	Yes
row_208	SERTAD1	SERTAD1	SERTA domain containing 1	5997	0.198702425	0.1298518	Yes
row_209	EIF1	EIF1	eukaryotic translation initiation factor 1	6064	0.195723683	0.12926218	Yes
row_210	ARHGAP32	null	null	6098	0.194008768	0.12971587	Yes
row_211	HOXA3	HOXA3	homeobox A3	6114	0.192983419	0.13073865	Yes
row_212	HOXA2	HOXA2	homeobox A2	6174	0.189631507	0.13037033	Yes
row_213	HTRA1	HTRA1	HtrA serine peptidase 1	6257	0.184716523	0.12927487	Yes
row_214	GLUL	GLUL	glutamate-ammonia ligase (glutamine synthetase)	6284	0.183617949	0.12994987	Yes
row_215	F2RL3	F2RL3	coagulation factor II (thrombin) receptor-like 3	6286	0.18351686	0.13141526	Yes
row_216	EMP1	EMP1	epithelial membrane protein 1	6324	0.181726754	0.13174249	Yes
row_217	FKBP7	FKBP7	FK506 binding protein 7	6338	0.180965111	0.13282849	Yes
row_218	MMP17	MMP17	matrix metallopeptidase 17 (membrane-inserted)	6366	0.179317415	0.13347188	Yes
row_219	KCNMA1	KCNMA1	potassium large conductance calcium-activated channel, subfar	6393	0.177640527	0.13414688	Yes
row_220	CPQ	null	null	6421	0.17607829	0.13479027	Yes
row_221	RABEP1	RABEP1	rabaptin, RAB GTPase binding effector protein 1	6441	0.1752671	0.13568658	Yes
row_222	ARHGAP35	null	null	6482	0.173375696	0.13591896	Yes
row_223	TBR1	TBR1	T-box, brain, 1	6508	0.171976626	0.13662559	Yes
row_224	GRIK1	GRIK1	glutamate receptor, ionotropic, kainate 1	6536	0.17051056	0.13726896	Yes
row_225	ZNF775	ZNF775	zinc finger protein 775	6557	0.169893995	0.13813366	Yes
row_226	RAB11FIP5	RAB11FIP5	RAB11 family interacting protein 5 (class I)	6560	0.169687197	0.13956743	Yes
row_227	CYP1B1	CYP1B1	cytochrome P450, family 1, subfamily B, polypeptide 1	6564	0.169571638	0.14096959	Yes
row_228	F2R	F2R	coagulation factor II (thrombin) receptor	6589	0.169130981	0.14170782	Yes
row_229	SIRT3	SIRT3	sirtuin (silent mating type information regulation 2 homolog) 3	6643	0.167294323	0.14152922	Yes
row_230	TOB1	TOB1	transducer of ERBB2, 1	6644	0.167261034	0.14302622	Yes
row_231	TTC28	TTC28	tetratricopeptide repeat domain 28	6678	0.165793478	0.14347991	Yes
row_232	PDLIM2	PDLIM2	PDZ and LIM domain 2 (mystique)	6716	0.163880706	0.14380714	Yes
row_233	NBEA	NBEA	neurobeachin	6752	0.162560329	0.1441976	Yes
row_234	PTGER3	PTGER3	prostaglandin E receptor 3 (subtype EP3)	6861	0.160139531	0.14228013	Yes
row_235	VCAM1	VCAM1	vascular cell adhesion molecule 1	6868	0.160139531	0.14358744	Yes
row_236	ACOX1	ACOX1	acyl-Coenzyme A oxidase 1, palmitoyl	6916	0.159049213	0.14359851	Yes
row_237	KLF6	KLF6	Kruppel-like factor 6	6924	0.158396915	0.14487422	Yes
row_238	ARHGAP4	ARHGAP4	Rho GTPase activating protein 4	7092	0.151611477	0.14109142	Yes
row_239	PCLO	PCLO	piccolo (presynaptic cytomatrix protein)	7101	0.151106432	0.1423355	Yes
row_240	DYNLL2	DYNLL2	dynein, light chain, LC8-type 2	7119	0.150281012	0.14329505	Yes
row_241	DUSP1	DUSP1	dual specificity phosphatase 1	7158	0.148194045	0.14359066	Yes
row_242	ZNF503	ZNF503	zinc finger protein 503	7266	0.143094808	0.1417048	Yes
row_243	PCDHB15	PCDHB15	protocadherin beta 15	7272	0.142743364	0.14304373	Yes
row_244	IER2	IER2	immediate early response 2	7275	0.142651558	0.1444775	Yes
row_245	TCF4	TCF4	transcription factor 4	7419	0.13537024	0.14145349	Yes
row_246	LSM14B	LSM14B	LSM14 homolog B (SCD6, S. cerevisiae)	7436	0.134725407	0.14244464	Yes
row_247	TENM3	null	null	7454	0.13428022	0.14340419	Yes
row_248	IL10RB	IL10RB	interleukin 10 receptor, beta	7465	0.133414567	0.14458503	Yes
row_249	CBR3	CBR3	carbonyl reductase 3	7521	0.131067395	0.14434318	Yes
row_250	CXCL12	CXCL12	chemokine (C-X-C motif) ligand 12 (stromal cell-derived factor 1	7554	0.130278334	0.1448285	Yes
row_251	RGS18	RGS18	regulator of G-protein signalling 18	7578	0.129775524	0.14559834	Yes
row_252	PCP4L1	PCP4L1	Purkinje cell protein 4 like 1	7633	0.128467575	0.14538811	Yes
row_253	ADGRG1	null	null	7691	0.12670958	0.14508303	Yes
row_254	MATN2	MATN2	matrilin 2	7735	0.125093624	0.14522056	Yes
row_255	SNX10	SNX10	sorting nexin 10	7743	0.124931008	0.14649627	Yes
row_256	TAC1	TAC1	tachykinin, precursor 1 (substance K, substance P, neurokinin 1	7795	0.123919562	0.14638087	Yes
row_257	HPGD	HPGD	hydroxyprostaglandin dehydrogenase 15-(NAD)	7886	0.122450553	0.14503248	Yes
row_258	HSPA2	HSPA2	heat shock 70kDa protein 2	8002	0.119341679	0.1428937	Yes
row_259	ADGRA3	null	null	8089	0.118687235	0.14167176	Yes
row_260	APOE	APOE	apolipoprotein E	8118	0.117303371	0.14228353	Yes
row_261	NAB2	NAB2	NGFI-A binding protein 2 (EGR1 binding protein 2)	8171	0.115736842	0.14213653	Yes
row_262	NSG2	null	null	8195	0.114801884	0.14290638	Yes
row_263	ADGRD1	null	null	8212	0.114469223	0.14389753	Yes
row_264	MYO5C	MYO5C	myosin VC	8218	0.114107713	0.14523646	Yes
row_265	CAND2	CAND2	cullin-associated and neddylation-dissociated 2 (putative)	8308	0.110294148	0.14391969	Yes
row_266	IFI44	IFI44	interferon-induced protein 44	8350	0.10809581	0.14412045	Yes
row_267	SHANK3	SHANK3	SH3 and multiple ankyrin repeat domains 3	8420	0.106163695	0.14343598	Yes
row_268	RAB20	RAB20	RAB20, member RAS oncogene family	8462	0.104030028	0.14363676	Yes
row_269	EPHA5	EPHA5	EPH receptor A5	8485	0.102780364	0.14443822	Yes
row_270	NACC2	null	null	8528	0.100812078	0.14460737	Yes
row_271	RASD1	RASD1	RAS, dexamethasone-induced 1	8592	0.097314984	0.1441126	Yes
row_272	EPHA7	EPHA7	EPH receptor A7	8607	0.09730041	0.145167	Yes
row_273	RELB	RELB	v-rel reticuloendotheliosis viral oncogene homolog B, nuclear fa	8646	0.095792741	0.1454626	Yes
row_274	AFF4	AFF4	AF4/FMR2 family, member 4	8655	0.095133364	0.14670669	Yes
row_275	SMAD1	SMAD1	SMAD, mothers against DPP homolog 1 (Drosophila)	8657	0.094913952	0.14817208	Yes
row_276	IL18	IL18	interleukin 18 (interferon-gamma-inducing factor)	8700	0.093261279	0.14834122	Yes
row_277	ITM2A	ITM2A	integral membrane protein 2A	8726	0.092783295	0.14904784	Yes

Enrichment of a stem cell signature in H2030-BrM in brain organotypic cultures. Transcriptomic changes experienced by H2030-BrM cells in organotypic cultures include the

upregulation of the gene set signature WONG_Adult_Tissue_Stem_Module. The table shows the upregulated genes belonging to the signature.

Supplementary Table 2.

Supplementary Table 2_Enrichment of a stem cell signature in H2030-BrM oncospheres_Corresponds to Fig.S2C

Ben Porath et al._PMID: 18443585_Geneset ESexp1 (SuppTable1)
Oncospheres vs co-culture with cell-cell contact

NAME	PROBE	GENE SYMBOL	GENE_TITLE	RANK IN GENE LIST	RANK METRIC SCORE	RUNNING ES	CORE ENRICHMENT
row_0	TEAD4	null	null	130	1.024573684	0.010472498	Yes
row_1	FGF2	null	null	132	1.021217823	0.026898688	Yes
row_2	SFRP1	null	null	153	0.930548549	0.040977463	Yes
row_3	SYNGR3	null	null	172	0.893029034	0.054544177	Yes
row_4	AP1M2	null	null	199	0.833103299	0.06677168	Yes
row_5	MCM3	null	null	253	0.750710666	0.07641269	Yes
row_6	CBS	null	null	291	0.70774138	0.08610575	Yes
row_7	SLC29A1	null	null	332	0.676245689	0.095151044	Yes
row_8	PRIM1	null	null	402	0.621987522	0.10197053	Yes
row_9	DIAPH2	null	null	403	0.621907115	0.11200221	Yes
row_10	MCM5	null	null	406	0.621322095	0.12193131	Yes
row_11	CDC6	null	null	443	0.602487028	0.12997313	Yes
row_12	MCM4	null	null	475	0.586127758	0.13798392	Yes
row_13	DHFR	null	null	494	0.577404022	0.14645945	Yes
row_14	CDT1	null	null	543	0.557014167	0.1532089	Yes
row_15	CDC25A	null	null	554	0.551998436	0.16164719	Yes
row_16	GAD1	null	null	600	0.534975529	0.16818087	Yes
row_17	MTHFD1	null	null	602	0.534266949	0.17675228	Yes
row_18	CDC20	null	null	613	0.530476332	0.18484342	Yes
row_19	RRAS2	null	null	626	0.524727225	0.19274867	Yes
row_20	RRM2	null	null	628	0.523922205	0.20115323	Yes
row_21	CHAF1A	null	null	635	0.522079408	0.2092952	Yes
row_22	DEK	null	null	660	0.512214065	0.21643974	Yes
row_23	MRE11A	null	null	661	0.512183785	0.22470152	Yes
row_24	NOLC1	null	null	666	0.509434938	0.23273268	Yes
row_25	PNN	null	null	710	0.496344447	0.23873636	Yes
row_26	MCM2	null	null	720	0.492111713	0.24625522	Yes
row_27	RFC4	null	null	732	0.486566156	0.25359148	Yes
row_28	UNG	null	null	737	0.483106762	0.26119795	Yes
row_29	BOP1	null	null	759	0.477881789	0.26792842	Yes
row_30	PUS1	null	null	797	0.467119783	0.2737401	Yes
row_31	RFC3	null	null	801	0.466049492	0.28111804	Yes
row_32	MCM6	null	null	814	0.461074591	0.28799653	Yes
row_33	NUDT1	null	null	877	0.444472969	0.29227862	Yes
row_34	FEN1	null	null	1022	0.41176331	0.29221416	Yes
row_35	ABCE1	null	null	1045	0.408603996	0.29778057	Yes
row_36	HSPD1	null	null	1076	0.403448015	0.30289122	Yes
row_37	ADD2	null	null	1077	0.403116792	0.3093937	Yes
row_38	RBBP8	null	null	1140	0.38980034	0.31279388	Yes
row_39	TFAM	null	null	1242	0.373224348	0.3141104	Yes
row_40	PFAS	null	null	1268	0.369595081	0.31890783	Yes
row_41	GART	null	null	1276	0.3679488	0.32451704	Yes
row_42	MSH6	null	null	1335	0.360455245	0.32763016	Yes
row_43	MCM7	null	null	1336	0.359999835	0.33343714	Yes
row_44	ILF3	null	null	1379	0.353750765	0.3371873	Yes
row_45	SMS	null	null	1380	0.353628218	0.34289148	Yes
row_46	DDX18	null	null	1469	0.341342807	0.34429917	Yes
row_47	NUP107	null	null	1549	0.3309187	0.34595785	Yes
row_48	CSE1L	null	null	1550	0.330876529	0.35129505	Yes
row_49	CCNA2	null	null	1562	0.329280615	0.3560942	Yes
row_50	DDX21	null	null	1569	0.329038084	0.36112234	Yes
row_51	ITPR3	null	null	1597	0.325574607	0.36511657	Yes
row_52	MICB	null	null	1630	0.321142226	0.36880645	Yes
row_53	MSH2	null	null	1637	0.319912225	0.37368736	Yes
row_54	HSPA8	null	null	1798	0.303086221	0.3711247	Yes
row_55	NTHL1	null	null	1917	0.287750632	0.37027076	Yes
row_56	PSIP1	null	null	1961	0.283292502	0.3728378	Yes
row_57	PODXL	null	null	1971	0.28224498	0.37697142	Yes
row_58	BUB1	null	null	2001	0.280100077	0.38013896	Yes
row_59	PRMT3	null	null	2089	0.271447152	0.38046578	Yes
row_60	MTHFD2	null	null	2149	0.266052991	0.38200957	Yes
row_61	PRPS1	null	null	2209	0.261414319	0.38347855	Yes
row_62	TMEFF1	null	null	2219	0.260237008	0.38725716	Yes
row_63	NFE2L3	null	null	2256	0.256974339	0.3897257	Yes
row_64	BIRC5	null	null	2305	0.251357108	0.39154473	Yes
row_65	KIF4A	null	null	2308	0.25125137	0.3955044	Yes
row_66	HESX1	null	null	2375	0.246089384	0.39640018	Yes
row_67	CRABP2	null	null	2378	0.24569416	0.4002702	Yes
row_68	GABRA5	null	null	2541	0.234240398	0.3965039	Yes
row_69	KCNS3	null	null	2660	0.225325122	0.39464298	Yes
row_70	SNRPA	null	null	2697	0.222264707	0.39655164	Yes
row_71	WDR12	null	null	2713	0.220593929	0.39941135	Yes
row_72	NPM3	null	null	2883	0.20950304	0.39492002	Yes
row_73	CHEK2	null	null	2912	0.207878739	0.39696917	Yes
row_74	BUB3	null	null	2985	0.203758284	0.3969027	Yes
row_75	MARS	null	null	3037	0.200757712	0.39776585	Yes
row_76	KIF5C	null	null	3123	0.195257187	0.3969568	Yes
row_77	PLCB3	null	null	3152	0.193671033	0.39877677	Yes
row_78	KPNA2	null	null	3157	0.193485945	0.40171152	Yes
row_79	NPM1	null	null	3185	0.191731483	0.40354678	Yes

Enrichment of a stem cell signature in H2030-BrM oncospheres. Transcriptomic changes experienced by H2030-BrM oncospheres that are not represented in H2030-BrM cells when grown

in co-culture conditions with cell-cell contacts with glial cells include the upregulation of the stem cell gene set signature described by Ben Porath et al[43]. The table shows the upregulated genes belonging to the signature.

Supplementary Table 3.

Supplementary Table 3_Commonly DEG in radioresistant conditions H2030-BrM in vitro_Corresponds to Fig.2A

10Gy

	Radio-sensitive conditions				Radio-resistant conditions			
	Adherent cells		Co-cultures (insert)		Oncospheres		Co-cultures (cell-cell contact)	
**Gene ID**	**Replica#1**	**Replica#2**	**Replica#1**	**Replica#2**	**Replica#1**	**Replica#2**	**Replica#1**	**Replica#2**
S100A9	7.59439	4.5155	28.5228	3.64529	1580.83	832.755	31.5604	42.0916
CITED4	7.12601	7.82344	8.75282	4.26437	15.209	13.2526	27.4243	26.4535
ADM2	1.06844	0.977898	2.24177	0.69009	0.318587	0.518863	0.610843	0.609238
SLC1A4	13.0187	13.2551	23.2088	10.885	6.15292	5.52887	7.13414	10.2621
B7H6	1.63611	1.20038	2.01825	1.4872	0.735604	0.462581	0.625675	0.963268
ASNS	111.828	123.523	206.443	85.6194	38.4651	63.7346	63.6966	74.4078
PPP1R15A	18.4296	20.1114	33.8886	12.1505	10.6333	11.6891	12.2159	11.0101
FAT4	5.42659	3.90715	7.35616	4.49208	1.1809	0.877306	1.41721	1.54036
SESN2	4.67358	5.33466	16.5771	3.37909	2.00617	3.8349	2.70772	3.44457
MAP1B	7.67632	8.15961	18.5048	9.30292	6.61733	2.98472	2.96531	3.51404

**Abreviations**	
Adherent cells	Adh
Co-cultures (insert)	Insert
Oncospheres	Onco
Co-cultures (cell-cell contact)	Cocult

Commonly DEG in radioresistant conditions H2030-BrM *in vitro*. A signature showing the commonly upregulated (2) and downregulated (8) genes between radioresistant and radiosensitive conditions. FPKM values are shown for each gene, replica and experimental condition.

Supplementary Table 4.

Supplementary Table 4_Radioresistant signature in H2030-BrM in brain organotypic cultures_Corresponds to Fig.2B

	Radio-sensitive conditions		Radio-resistant conditions	
	Adherent cells		Brain slices	
**Gene ID**	**Replica#1**	**Replica#2**	**Replica#1**	**Replica#2**
S100A9	49.8806	30.1378	1887.2	2045.53
CITED4	24.4308	15.2641	43.5374	44.416
ADM2	1.2019	0.676868	0.598335	0.427862
SLC1A4	12.8192	13.0941	5.65409	5.50357
ASNS	192.069	140.292	55.8656	56.816
B7H6	0.992509	1.14978	0.367122	0.409746
FAT4	5.38556	6.71373	1.9204	1.79175
PPP1R15A	26.3816	30.8419	19.4836	19.2743
MAP1B	2.7817	3.75648	2.06515	2.12743

Radioresistant signature in H2030-BrM in brain organotypic cultures. The radio-resistance

signature is represented in H2030-BrM cells growing in organotypic cultures. FPKM values are shown for each gene, replica and experimental condition.

Supplementary Table 5.

Supplementary Table 5_Top 25 upregulated genes in H2030-BrM in brain organotypic cultures

FDR=0.05

**Name**	**baseMean**	**log2FoldChange**	**lfcSE**	**stat**	**pvalue**	**padj**
S100A9	17667.64142	4.345221815	0.2871146	15.13410111	9.64901E-52	3.86829E-49
S100A8	132.8617673	3.957446968	0.2811576	14.07554643	5.36883E-45	1.36658E-42
FAM83A	318.3020428	3.120515852	0.1859135	16.78477409	3.15427E-63	1.94546E-60
S100A4	716.0791138	2.927415658	0.1383012	21.16695316	1.9261E-99	4.41252E-96
SPNS2	137.6005157	2.91168495	0.2470245	11.78703023	4.55315E-32	4.32037E-30
PLP1	37.03272467	2.833083114	0.3424643	8.272638718	1.31026E-16	3.2226E-15
KIF5A	38.35076374	2.550084349	0.3421988	7.452055155	9.18973E-14	1.5966E-12
TMEM132B	59.56452267	2.490892934	0.3085066	8.074033979	6.80129E-16	1.56031E-14
CLDN4	125.2852834	2.440134849	0.2484883	9.819920129	9.24167E-23	4.32068E-21
ITGA10	215.6828891	2.435036971	0.2025878	12.01966465	2.80106E-33	2.87935E-31
NACAD	90.36572445	2.223636054	0.2682053	8.290797446	1.12492E-16	2.78382E-15
GBP2	93.26812085	2.221095795	0.2665493	8.332777828	7.89673E-17	1.98483E-15
MILR1	53.75359509	2.204661121	0.3114474	7.078759352	1.45451E-12	2.15967E-11
ALDH3A1	26.1311218	2.191499188	0.3478726	6.299717318	2.98189E-10	3.24187E-09
SHC2	147.2399142	2.189553474	0.2264569	9.668742929	4.09375E-22	1.79365E-20
DLL4	277.501276	2.177090104	0.1797622	12.11094752	9.24203E-34	9.88035E-32
ZNF295-AS1	53.91570455	2.094110378	0.3093444	6.769511546	1.29218E-11	1.69569E-10
TNFRSF19	259.3403404	2.063482861	0.1873033	11.01680075	3.17131E-28	2.29077E-26
ZNF467	918.9162207	2.01200798	0.1162718	17.3043549	4.36139E-67	4.3712E-64
AC068768.1	117.7816076	1.989998679	0.2436273	8.168209465	3.13E-16	7.41399E-15
SUSD2	806.1718396	1.982671353	0.2853347	6.948582267	3.68975E-12	5.21312E-11
LINC01801	74.59836121	1.902725079	0.2822235	6.741909134	1.56319E-11	2.03138E-10
FAM107B	5171.896475	1.90223444	0.0818393	23.24353298	1.6538E-119	6.6299E-116
LURAP1L	95.69413398	1.892097179	0.2586377	7.315628925	2.56179E-13	4.21774E-12
THRB	256.3074852	1.854725726	0.181294	10.23048867	1.44787E-24	7.68809E-23

Top 25 upregulated genes in H2030-BrM in brain organotypic cultures. Top 25 upregulated genes in H2030-BrM cells cultured on brain organotypic cultures compared to *in vitro* cultured conditions. DESeq2 was used to identify differentially expressed genes between two groups of samples based on a negative binomial distribution model. Values in the table represent normalized base mean expression level, Log2 fold change, shrinkage of effect size (lfcSE), Wald statistic, Wald test p-value and adjusted p-value below a given FDR cutoff, alpha = 0.05.

Supplementary Table 6.

Supplementary Table 6_Enrichment of NF-kB signatures in radioresistant conditions_Corresponds to Fig.S2H

NFKB signature_In vitro_REACTOME_MAP_KINASE_ACTIVATION_IN_TLR_CASCADE

Signature evaluated DEG H2030-BrM resistant (Onc-Cocult) vs sensitive (Adh-Insert) surrogates irradiated (10Gy)

NAME	GENE SYMBOL	RANK IN GENE LIST	RANK METRIC SCORE	RUNNING ES	CORE ENRICHMENT
row_0	DUSP6	57	1.055718184	0.017773056	Yes
row_1	FOS	236	0.597747922	0.029952288	Yes
row_2	MAP2K1	293	0.541081727	0.047771573	Yes
row_3	PPP2R1B	673	0.368947476	0.050658587	Yes
row_4	MAPK7	910	0.319571048	0.060156483	Yes
row_5	MAP2K3	1201	0.279324442	0.06715796	Yes
row_6	DUSP7	1239	0.273496807	0.08585562	Yes
row_7	TAB1	1545	0.240439445	0.092163645	Yes
row_8	CDK1	1622	0.234001532	0.10905833	Yes
row_9	PPP2R1A	1651	0.232045755	0.12817205	Yes
row_10	RPS6KA1	2289	0.187860012	0.119131744	Yes
row_11	MAPK3	2313	0.186190441	0.13847661	Yes
row_12	MAP2K6	2415	0.180324972	0.15421556	Yes
row_13	MAPKAPK3	3437	0.130279168	0.12742294	Yes
row_14	MAP2K4	3862	0.113285482	0.1282296	Yes
row_15	DUSP3	3978	0.108269811	0.14332132	Yes
row_16	PPP2CA	4288	0.096997254	0.14944443	Yes
row_17	ELK1	5069	0.072650999	0.13379323	Yes
row_18	RIPK2	5210	0.068864122	0.14772922	Yes
row_19	DUSP4	5308	0.066248477	0.16365308	Yes
row_20	MAPKAPK2	5846	0.051812474	0.15923575	Yes
row_21	MAP2K7	5911	0.050377205	0.1766852	Yes
row_22	PPP2R5D	5977	0.048563723	0.19408841	Yes
row_23	MAP3K7	6139	0.045003496	0.20705356	Yes
row_24	IRAK1	6338	0.040299404	0.21830818	Yes
row_25	CREB1	6693	0.032273497	0.22235096	Yes

NFKB signature_Ex vivo_REACTOME_MAP_KINASE_ACTIVATION_IN_TLR_CASCADE

Signature evaluated DEG H2030-BrM in brain slices vs H2030-BrM in vitro (Adh)

NAME	GENE SYMBOL	RANK IN GENE LIST	RANK METRIC SCORE	RUNNING ES	CORE ENRICHMENT
row_0	DUSP6	183	1.141023278	0.014733569	Yes
row_1	MAPK7	240	1.068263173	0.033405244	Yes
row_2	MAP2K6	304	0.987556219	0.051859856	Yes
row_3	ATF2	330	0.967172086	0.071492806	Yes
row_4	ELK1	429	0.898986399	0.08886211	Yes
row_5	DUSP3	576	0.826986849	0.104743004	Yes
row_6	TAB3	809	0.74672997	0.117957145	Yes
row_7	MAPK11	1177	0.651373148	0.1269851	Yes
row_8	IRAK2	1527	0.584053278	0.13657123	Yes
row_9	MAPKAPK2	1705	0.556251705	0.15149085	Yes
row_10	MEF2A	1950	0.520993769	0.16433288	Yes
row_11	CREB1	2277	0.478613585	0.1746322	Yes
row_12	MAP2K4	2421	0.461385131	0.19060613	Yes
row_13	TAB2	2573	0.446571827	0.20633197	Yes
row_14	FOS	2654	0.437566936	0.22425944	Yes
row_15	MAPK3	2657	0.437408179	0.24460559	Yes
row_16	MAP2K3	3009	0.399760306	0.25412968	Yes
row_17	RIPK2	3426	0.363707274	0.26163822	Yes
row_18	IRAK1	3437	0.362179995	0.2817363	Yes
row_19	DUSP4	3566	0.352443099	0.29817536	Yes
row_20	MAP3K7	3665	0.344691396	0.31554466	Yes
row_21	MEF2C	4414	0.288518876	0.3127583	Yes
row_22	MAP2K2	4825	0.262437075	0.3204529	Yes
row_23	RPS6KA2	5474	0.223521724	0.3207674	Yes
row_24	TRAF6	6166	0.190309897	0.31974855	Yes
row_25	PPP2R1A	6265	0.184291035	0.33711788	Yes
row_26	DUSP7	6687	0.16543071	0.34447137	Yes
row_27	TAB1	6893	0.159416139	0.35852274	Yes
row_28	MAPK1	8555	0.099293478	0.32742542	Yes
row_29	MAPK14	8560	0.099059172	0.34770957	Yes
row_30	MAPK8	8569	0.098485753	0.36786965	Yes
row_31	MAPK9	8630	0.096635945	0.3864173	Yes

Enrichment of NF-κB signatures in radioresistant conditions. Genes set enrichment analysis performed in radioresistant conditions both *in vitro* (upper table) and *ex vivo* (lower table) showed

gene signatures related to NF-κB. Components of the pathway represented in each experimental condition are shown.

Supplementary Table 7.

Supplementary Table 7_Clusters 1-9 0.8res scRNAseq H2030-BrM

Gene	p_val	avg_logFC	pct.1	pct.2	p_val_adj	cluster
SCG2	0	1.16971289	0.88	0.59	0	0
BASP1	0	-0.888698	0.743	0.971	0	0
ID3	0	-0.9031023	0.712	0.982	0	0
S100A16	0	-1.2066941	0.136	0.769	0	0
S100A4	0	-1.3254825	0.792	0.987	0	0
NOTUM	0	-1.3342398	0.155	0.747	0	0
ID1	5.7165E-299	-0.88083	0.69	0.968	1.9354E-294	0
DUSP1	1.4543E-291	-1.0220182	0.572	0.918	4.9235E-287	0
LGALS3BP	4.14E-268	-1.0016312	0.344	0.807	1.4016E-263	0
CAMK2N1	5.7604E-268	-0.8891273	0.296	0.818	1.9502E-263	0
SLC26A2	2.5467E-267	-0.8584559	0.428	0.879	8.6223E-263	0
DCBLD2	3.4377E-261	-0.8532242	0.253	0.772	1.1639E-256	0
MUC1	3.6109E-242	-0.912265	0.638	0.903	1.2225E-237	0
SYCE1L	3.5698E-226	-0.8188418	0.424	0.825	1.2086E-221	0
TGM2	4.4156E-219	-1.0966095	0.244	0.717	1.495E-214	0
ID2	1.8944E-218	-0.7944906	0.168	0.676	6.4135E-214	0
ALDH3A1	1.0399E-214	-1.0079697	0.161	0.652	3.5207E-210	0
RHOBTB3	1.1973E-212	-0.7456806	0.316	0.755	4.0537E-208	0
TGFBI	3.2458E-204	-0.7109942	0.07	0.551	1.0989E-199	0
CD24	4.2591E-198	-0.7422226	0.035	0.502	1.442E-193	0
SLPI	1.6801E-197	-0.7785397	0.346	0.79	5.6881E-193	0
NEAT1	2.1755E-194	-0.7369084	0.941	0.985	7.3653E-190	0
RPS29	3.2071E-192	0.29000946	1	1	1.0858E-187	0
VGF	7.706E-186	0.98299823	0.313	0.06	2.6089E-181	0
ANXA2	5.7513E-183	-0.418749	0.959	0.995	1.9472E-178	0
SPP1	2.134E-178	0.67422877	0.978	0.974	7.225E-174	0
SUSD2	8.621E-173	-0.6902488	0.27	0.707	2.9187E-168	0
MT2A	4.9859E-162	-0.661188	0.947	0.994	1.688E-157	0
CENPX	2.4154E-159	-0.5537993	0.582	0.89	8.1776E-155	0
RPS2	9.2284E-156	0.28859122	0.999	0.999	3.1244E-151	0
KRT8	2.8532E-153	-0.5717011	0.834	0.963	9.6598E-149	0
CEACAM6	4.4632E-153	-0.7362584	0.033	0.42	1.5111E-148	0
SSR4	4.3132E-152	-0.4799208	0.879	0.976	1.4603E-147	0
APLP2	4.5086E-152	-0.4986345	0.829	0.958	1.5264E-147	0
GUK1	8.2653E-152	-0.4696448	0.782	0.953	2.7983E-147	0
CDKN1A	2.1324E-149	-0.5898689	0.108	0.509	7.2193E-145	0
KRT18	1.0532E-147	-0.5507465	0.884	0.981	3.5657E-143	0
GLG1	1.2506E-145	-0.4915959	0.758	0.935	4.2339E-141	0
BMP7	3.641E-145	-0.570933	0.352	0.731	1.2327E-140	0
TPD52L1	5.6125E-144	-0.5696389	0.327	0.725	1.9002E-139	0
MAL2	8.8371E-144	-0.5128141	0.047	0.423	2.9919E-139	0
ZBTB38	1.3745E-143	-0.54623	0.296	0.688	4.6534E-139	0
FAM83A	4.0946E-142	-0.5307493	0.417	0.816	1.3863E-137	0
C19orf33	2.3048E-141	-0.5070288	0.619	0.893	7.8032E-137	0
FTH1	1.6698E-140	0.41589304	0.998	0.998	5.6534E-136	0
CYP24A1	1.6932E-138	-0.4970423	0.674	0.952	5.7326E-134	0
HLA-C	1.8804E-138	-0.645819	0.468	0.788	6.3661E-134	0
SEMA3A	3.2106E-138	0.58255059	0.465	0.195	1.087E-133	0
TSC22D3	4.2617E-137	-0.591609	0.288	0.679	1.4428E-132	0
LYPD3	7.1395E-137	-0.5584403	0.152	0.543	2.4172E-132	0
S100A13	3.03E-135	-0.5847512	0.239	0.626	1.0259E-130	0
PDLIM1	4.8355E-134	-0.5115273	0.105	0.48	1.6371E-129	0
S100A10	7.752E-133	-0.2855762	0.995	1	2.6245E-128	0
S100A6	1.3323E-127	-0.3600524	0.994	1	4.5108E-123	0
ALDH3B1	1.8716E-122	-0.4521953	0.181	0.559	6.3364E-118	0
SLC2A1	9.4765E-122	-0.5282175	0.2	0.558	3.2084E-117	0
B2M	8.1238E-121	-0.5032911	0.878	0.968	2.7504E-116	0
CST3	1.9665E-120	-0.463427	0.917	0.982	6.6579E-116	0
MSI2	4.9446E-120	-0.4500717	0.22	0.594	1.674E-115	0
FGFBP1	5.9752E-120	-0.505186	0.032	0.359	2.023E-115	0
HLA-B	7.8176E-119	-0.7100992	0.296	0.632	2.6467E-114	0
HSPB1	9.5039E-119	0.46069447	0.928	0.89	3.2177E-114	0
PKM	4.5429E-118	-0.3640567	0.938	0.994	1.538E-113	0
ITPR1	5.1093E-118	-0.5441281	0.139	0.486	1.7298E-113	0
PTPRR	6.2619E-118	-0.4204499	0.046	0.378	2.12E-113	0
KCNF1	8.7168E-116	-0.4927606	0.299	0.676	2.9512E-111	0
DDX59	9.5607E-116	-0.4693751	0.126	0.469	3.2369E-111	0
CD59	1.6124E-114	-0.4218075	0.122	0.471	5.4588E-110	0
SLC20A1	8.2411E-112	-0.5373051	0.239	0.587	2.7901E-107	0
LMNA	3.5497E-111	-0.3564708	0.925	0.986	1.2018E-106	0
ST3GAL4	8.8899E-110	-0.422805	0.113	0.446	3.0098E-105	0
FZD10	1.0339E-109	-0.4215398	0.044	0.357	3.5004E-105	0
GPRC5A	1.0594E-109	-0.466745	0.244	0.594	3.5866E-105	0
SH3KBP1	1.6465E-107	-0.440323	0.331	0.674	5.5745E-103	0
MT-CO1	9.4945E-107	0.28014943	0.996	0.998	3.2145E-102	0
RPS4Y1	3.9086E-106	-0.2783007	0.996	0.999	1.3233E-101	0
FIBCD1	5.4862E-106	-0.4164841	0.081	0.402	1.8574E-101	0
PHLDB2	9.4041E-106	-0.4056519	0.108	0.441	3.1839E-101	0
SMS	3.016E-103	-0.4139019	0.62	0.871	1.02109E-98	0
TOM1L2	6.307E-103	-0.4334291	0.128	0.457	2.13529E-98	0
IGFBP5	1.792E-102	-0.6504406	0.181	0.519	6.06714E-98	0
BAG1	3.1497E-100	-0.4064025	0.464	0.776	1.06636E-95	0
LGALS3	5.4165E-100	-0.3757076	0.035	0.324	1.83382E-95	0
ADGRG2	6.0444E-100	-0.3986328	0.038	0.329	2.0464E-95	0
MARCKSL1	2.7107E-99	0.47178599	0.645	0.419	9.17746E-95	0
IL18	4.7259E-99	-0.4584754	0.476	0.76	1.60001E-94	0
TNS4	7.0035E-99	-0.379956	0.028	0.31	2.37109E-94	0
RNF19A	3.77775E-98	-0.4493912	0.334	0.657	1.279E-93	0
EDIL3	1.03082E-96	0.60503621	0.666	0.475	3.48995E-92	0
NDRG1	1.35973E-96	-0.4653083	0.319	0.652	4.6035E-92	0
DAP	1.37283E-96	-0.4085098	0.398	0.702	4.64785E-92	0
NRIP1	5.1943E-96	-0.361632	0.119	0.44	1.75858E-91	0
PAQR5	6.42193E-96	-0.3848544	0.173	0.495	2.17421E-91	0
ASPSCR1	1.31756E-94	-0.4286302	0.355	0.676	4.46074E-90	0
MGST3	1.36339E-94	-0.2953047	0.934	0.992	4.61588E-90	0
ENO1	2.09172E-94	-0.2937951	0.947	0.992	7.08172E-90	0
CHCHD2	2.36921E-94	0.35481881	0.95	0.942	8.0212E-90	0
SNHG25	4.71701E-94	-0.4338266	0.507	0.78	1.59699E-89	0
ZMYND8	1.53587E-93	-0.398309	0.383	0.695	5.19983E-89	0
RPS15A	2.85144E-93	0.30355012	0.989	0.985	9.65382E-89	0
GM2A	1.00928E-91	-0.4412848	0.258	0.573	3.41703E-87	0
DMKN	1.60433E-91	0.38517757	0.883	0.858	5.43162E-87	0
PCDH7	4.59943E-91	0.38079088	0.312	0.114	1.55718E-86	0
BRI3	1.00302E-90	-0.351423	0.714	0.906	3.39581E-86	0
PALMD	1.34856E-87	-0.3107666	0.141	0.459	4.5657E-83	0
CTB-129O4.1	1.48616E-87	-0.351583	0.144	0.45	5.03154E-83	0
LINC00963	2.46317E-87	-0.4088985	0.349	0.654	8.33932E-83	0
CENPV	3.02982E-87	0.47922817	0.645	0.464	1.02578E-82	0
NPC2	2.01883E-86	-0.3912154	0.446	0.732	6.83496E-82	0
SERPINE2	2.49832E-86	-0.5155291	0.221	0.517	8.4583E-82	0
SPOCK1	6.79809E-86	0.28512615	0.13	0.019	2.30156E-81	0
DSP	9.2331E-86	-0.3581733	0.884	0.973	3.12596E-81	0
MSLN	3.71826E-85	-0.4618037	0.438	0.735	1.25886E-80	0
SNHG29	1.64976E-83	-0.291858	0.988	0.997	5.58543E-79	0
P4HA2	5.59549E-83	-0.3429354	0.168	0.466	1.89441E-78	0
S100A14	8.37494E-83	-0.3839555	0.009	0.245	2.83542E-78	0
AMOTL2	1.01131E-81	-0.3907274	0.284	0.586	3.42388E-77	0
HIF1A	1.5952E-81	-0.3221388	0.47	0.772	5.40071E-77	0
ATOH8	4.38924E-80	-0.3192034	0.023	0.263	1.48602E-75	0
ZNF503	1.96985E-79	-0.4362682	0.258	0.543	6.66912E-75	0
LGALS1	3.92367E-79	-0.2746233	0.977	0.996	1.3284E-74	0
GNG4	9.36539E-79	0.42677268	0.414	0.207	3.17075E-74	0
HEY1	1.00725E-78	-0.3083049	0.045	0.299	3.41013E-74	0
PSORS1C1	2.00978E-78	-0.2961637	0.022	0.257	6.80432E-74	0
PCSK7	3.37346E-78	-0.3525333	0.28	0.58	1.14212E-73	0
SAMD11	3.40353E-78	-0.3221052	0.12	0.407	1.1523E-73	0
EVA1C	4.50545E-78	-0.3074269	0.184	0.481	1.52536E-73	0
CCND3	6.9186E-78	-0.328397	0.117	0.397	2.34236E-73	0
PRDX1	3.89922E-76	-0.3018913	0.971	0.99	1.32012E-71	0
GLRX	2.22113E-75	-0.3363348	0.132	0.403	7.51986E-71	0
CDKN1C	5.47477E-75	-0.4070481	0.087	0.344	1.85354E-70	0
GNAS	1.00718E-74	0.26118197	0.98	0.98	3.40992E-70	0
TFPI2	1.1393E-74	0.3466478	0.982	0.98	3.8572E-70	0
FNBP1	1.31047E-74	-0.3420906	0.253	0.538	4.43672E-70	0
ST3GAL1	1.52737E-74	-0.2792089	0.044	0.282	5.17108E-70	0
KTN1	8.56433E-74	0.28511538	0.986	0.989	2.89954E-69	0
JUN	1.17466E-73	-0.4411852	0.275	0.566	3.97693E-69	0
CREM	2.63743E-73	0.45198922	0.522	0.336	8.92929E-69	0
SOX4	1.34358E-72	0.32903842	0.986	0.981	4.54883E-68	0
PAFAH1B3	1.56556E-72	0.4109178	0.8	0.744	5.30037E-68	0
TACC2	1.75802E-72	-0.3282156	0.252	0.541	5.95197E-68	0
PFN1	9.20038E-72	0.29353441	0.971	0.977	3.11488E-67	0
RETREG1	1.69769E-71	-0.2885376	0.084	0.335	5.74769E-67	0
CDH11	2.14677E-71	0.43651037	0.553	0.371	7.2681E-67	0
MAP2	2.18999E-71	0.55832907	0.534	0.343	7.41443E-67	0
ERGIC1	2.92051E-71	-0.3175959	0.245	0.526	9.88768E-67	0
SCEL	3.36084E-71	-0.3312825	0.124	0.384	1.13785E-66	0
AKR1C3	4.70921E-71	-0.2703965	0.052	0.294	1.59435E-66	0
SERINC2	5.86519E-71	-0.2874633	0.092	0.349	1.98572E-66	0
SPAG1	6.15846E-71	-0.297161	0.145	0.412	2.08501E-66	0
CD109	3.58523E-70	-0.2997242	0.164	0.437	1.21382E-65	0
CEMIP	4.06774E-70	-0.3228715	0.039	0.264	1.37718E-65	0
HMGA1	8.44372E-70	0.40727091	0.737	0.624	2.85871E-65	0
STXBP5	1.7071E-69	-0.2859567	0.082	0.327	5.77955E-65	0
SH3BP5	1.84353E-69	-0.3205317	0.066	0.3	6.24147E-65	0
ZFP36	5.20571E-69	-0.3639908	0.184	0.453	1.76245E-64	0
GADD45A	6.8186E-69	-0.2955598	0.104	0.353	2.30851E-64	0
CDH1	1.29855E-68	-0.2597804	0.026	0.24	4.39638E-64	0
HTRA1	3.78365E-68	-0.3482506	0.159	0.421	1.28099E-63	0
SPECC1	1.27192E-67	-0.2976289	0.216	0.489	4.30622E-63	0
TLE5	1.36577E-67	0.4191152	0.762	0.675	4.62394E-63	0
F2R	6.55457E-67	-0.3086043	0.093	0.331	2.21912E-62	0
MAP1B	6.76928E-67	-0.319967	0.157	0.428	2.29181E-62	0
AC005256.1	8.63662E-67	-0.2950294	0.103	0.353	2.92401E-62	0
LUCAT1	1.31478E-66	-0.4082224	0.297	0.568	4.45131E-62	0
HM13	1.77865E-66	-0.3143296	0.605	0.833	6.02181E-62	0
TNNT1	4.13509E-66	0.47985543	0.725	0.621	1.39998E-61	0
TMEM59	6.83438E-66	-0.3085832	0.443	0.717	2.31385E-61	0
PTMA	2.86328E-65	0.29948458	0.989	0.991	9.69393E-61	0
CCL2	5.60447E-65	-0.2993003	0.151	0.415	1.89745E-60	0
PDK4	2.59378E-64	-0.323983	0.105	0.35	8.7815E-60	0
FLNB	7.63784E-64	-0.3275656	0.433	0.707	2.58587E-59	0
KCNK1	1.03709E-63	-0.2558818	0.051	0.269	3.51116E-59	0
CYB5B	3.01464E-63	-0.3336346	0.45	0.709	1.02064E-58	0
IGSF8	4.36928E-63	-0.3239194	0.258	0.517	1.47926E-58	0
MAFK	6.81421E-63	-0.3031292	0.208	0.475	2.30702E-58	0
CD63	1.07124E-62	-0.2723245	0.833	0.941	3.62678E-58	0
NME4	1.88477E-62	0.34906451	0.808	0.757	6.38109E-58	0
TMOD3	2.43061E-62	-0.3021817	0.518	0.764	8.22907E-58	0
LPGAT1	1.13255E-61	-0.2668246	0.156	0.408	3.83437E-57	0
RBM3	1.50202E-61	-0.2776138	0.791	0.928	5.08526E-57	0
AP2M1	1.81489E-61	-0.2749422	0.742	0.912	6.1445E-57	0
ITPRID2	2.10171E-61	-0.2661509	0.136	0.379	7.11556E-57	0
MT-ATP8	2.64101E-61	0.33109681	0.961	0.961	8.94139E-57	0
PLEC	2.94878E-61	-0.2785783	0.628	0.852	9.9834E-57	0
CPA4	3.92505E-61	-0.2839731	0.032	0.231	1.32886E-56	0
YPEL5	5.66808E-61	-0.3509669	0.472	0.718	1.91899E-56	0
PDXK	1.56267E-60	-0.3047303	0.362	0.627	5.29057E-56	0
JUNB	1.73225E-60	-0.3610843	0.566	0.796	5.86471E-56	0
RNF217	2.01269E-60	-0.2590172	0.113	0.353	6.81415E-56	0
EPHX1	5.42448E-60	-0.2693511	0.209	0.478	1.83651E-55	0
CMIP	1.15984E-59	-0.3131912	0.664	0.864	3.92674E-55	0
RP1-80N2.2	2.62936E-59	-0.2680338	0.014	0.195	8.90196E-55	0
DENND3	5.95188E-59	-0.2713593	0.093	0.316	2.01507E-54	0
LAMA5-AS1	8.31587E-59	-0.2664102	0.143	0.393	2.81542E-54	0
ATP2B1	2.58984E-58	0.4290664	0.686	0.583	8.76816E-54	0
HSP90AA1	3.89582E-58	0.2575163	0.981	0.974	1.31897E-53	0
ATOX1	5.9336E-58	-0.2895515	0.779	0.921	2.00888E-53	0
ATP1A1	3.51146E-57	-0.2853178	0.392	0.658	1.18884E-52	0
PDLIM2	3.93367E-57	-0.2821427	0.334	0.597	1.33178E-52	0
CSRP1	4.28399E-57	-0.2544725	0.175	0.423	1.45039E-52	0
FLNA	2.64381E-56	-0.2858522	0.364	0.626	8.95088E-52	0
LRATD2	9.02958E-56	-0.2789007	0.16	0.4	3.05705E-51	0
PPP1R14B-AS1	1.01528E-55	-0.2563516	0.096	0.311	3.43733E-51	0
CRIM1	1.08472E-55	-0.288142	0.333	0.595	3.67241E-51	0
NCL	2.28043E-55	0.27906279	0.913	0.908	7.72061E-51	0
SERINC5	4.24975E-55	-0.2618926	0.137	0.368	1.4388E-50	0
RAB31	4.37965E-55	-0.288931	0.538	0.763	1.48278E-50	0
UBC	4.6525E-55	-0.2547858	0.855	0.953	1.57515E-50	0
TMC4	6.50589E-55	-0.2644821	0.038	0.225	2.20263E-50	0
SH3GLB1	1.49318E-54	-0.2914936	0.426	0.676	5.0553E-50	0
FAM20C	2.05964E-54	-0.2635039	0.299	0.564	6.97313E-50	0
RABAC1	6.31295E-54	-0.2533954	0.676	0.879	2.13731E-49	0
PDIA4	8.87487E-54	0.36134732	0.746	0.706	3.00468E-49	0
CIB2	1.03657E-53	0.31202714	0.306	0.148	3.5094E-49	0
CTSB	1.5373E-53	-0.288875	0.625	0.822	5.20468E-49	0
NAMPT	1.75494E-53	-0.3592797	0.229	0.47	5.94151E-49	0
DSTN	2.81722E-53	-0.2624246	0.71	0.874	9.53797E-49	0
GAN	3.62409E-53	-0.2754206	0.265	0.51	1.22697E-48	0
APOE	4.09584E-53	0.32034007	0.197	0.07	1.38669E-48	0
SELENOP	1.49523E-52	-0.3066109	0.313	0.565	5.06224E-48	0
HLA-A	1.70007E-52	-0.2828637	0.916	0.971	5.75574E-48	0
EPHA2	1.98855E-52	-0.2735484	0.236	0.473	6.73242E-48	0
NR3C1	2.54761E-52	-0.256037	0.204	0.447	8.6252E-48	0
SYNM	4.63291E-52	-0.2781924	0.288	0.536	1.56852E-47	0
S100P	9.50271E-52	-0.3743025	0.014	0.178	3.21724E-47	0
CMTM4	7.94692E-51	-0.2760895	0.352	0.589	2.69051E-46	0
COL26A1	1.2066E-49	0.37401678	0.343	0.186	4.08506E-45	0
RPL23	2.47869E-49	-0.2502084	0.976	0.992	8.39187E-45	0
ABCC3	2.54823E-49	-0.2898684	0.627	0.824	8.62728E-45	0
PERP	1.0244E-48	-0.2523941	0.607	0.826	3.46821E-44	0
SERPINB6	2.27025E-48	-0.2550543	0.349	0.59	7.68616E-44	0
MEX3A	2.43663E-48	0.29430545	0.302	0.151	8.24944E-44	0
LDHA	3.50759E-48	-0.2513265	0.859	0.958	1.18753E-43	0
PTMS	5.25018E-48	0.34249506	0.878	0.859	1.7775E-43	0
ETFB	5.90595E-48	-0.2611473	0.683	0.852	1.99952E-43	0
AKR1B1	8.64647E-48	-0.2853042	0.569	0.782	2.92735E-43	0
SNHG19	8.70637E-48	-0.2879865	0.773	0.906	2.94763E-43	0
CDCP1	2.33664E-47	-0.2521292	0.303	0.538	7.91093E-43	0
ERRFI1	3.3144E-47	-0.2569246	0.206	0.435	1.12212E-42	0
NNMT	6.8758E-47	-0.2542998	0.665	0.864	2.32787E-42	0
SPTBN1	9.13683E-47	-0.2816493	0.527	0.748	3.09336E-42	0
MAP1LC3B	1.5683E-46	-0.2623001	0.527	0.751	5.30963E-42	0
MCFD2	4.45638E-46	-0.2546566	0.514	0.743	1.5088E-41	0
SIVA1	5.61061E-46	0.31851455	0.773	0.713	1.8995E-41	0
MAP2K1	6.83684E-46	0.39590506	0.366	0.219	2.3147E-41	0
FOSL2	1.0863E-45	-0.2575159	0.464	0.699	3.6778E-41	0
CTSZ	1.7972E-45	-0.2646501	0.678	0.859	6.0846E-41	0
CAST	1.98664E-45	-0.2504473	0.789	0.919	6.726E-41	0
KLF6	7.15836E-45	-0.326089	0.341	0.569	2.4235E-40	0
MRPL52	9.43472E-45	0.25530966	0.864	0.849	3.1942E-40	0
ITGB4	1.84144E-44	-0.2555274	0.38	0.606	6.2344E-40	0
GAS5	4.37847E-44	-0.3582344	0.73	0.845	1.4824E-39	0
CTSD	5.90084E-44	-0.2574643	0.68	0.87	1.9978E-39	0
NOVA1	3.23995E-43	0.3980763	0.556	0.444	1.0969E-38	0
ZFAND5	6.1166E-43	-0.2593036	0.505	0.733	2.0708E-38	0
CCND1	2.7454E-41	-0.2601519	0.521	0.72	9.2948E-37	0
S100A9	2.97474E-41	-0.9516727	0.437	0.634	1.0071E-36	0
RBM25	3.44475E-41	0.26990575	0.823	0.815	1.1663E-36	0
HAS2	3.95187E-41	0.34164908	0.3	0.164	1.3379E-36	0
APOC1	5.99477E-40	0.35902876	0.562	0.439	2.0296E-35	0
CNFN	7.01178E-40	0.38273901	0.537	0.424	2.3739E-35	0
H3-3B	1.67354E-39	0.25013408	0.916	0.932	5.6659E-35	0
SRRM2	2.11829E-39	0.26090535	0.837	0.83	7.1717E-35	0
C12orf57	2.23309E-39	0.27743506	0.865	0.858	7.5603E-35	0
FXYD5	2.50192E-39	-0.2877427	0.408	0.621	8.4705E-35	0
EDIL3-DT	3.8656E-39	0.27631244	0.252	0.125	1.3087E-34	0
STMN1	9.2663E-39	0.29865286	0.834	0.8	3.1372E-34	0
CFDP1	5.28633E-38	0.28189806	0.785	0.761	1.7897E-33	0
NME3	6.60424E-37	0.31667896	0.728	0.691	2.2359E-32	0
SPRY1	2.15688E-36	0.35231254	0.727	0.65	7.3023E-32	0
TIMP1	1.32533E-34	0.50458253	0.986	0.998	4.487E-30	0
MALAT1	1.97357E-34	-0.3346069	0.998	0.998	6.6817E-30	0
HSP90B1	2.90234E-34	0.28058738	0.9	0.909	9.8262E-30	0
FERMT1	7.31205E-33	-0.294194	0.312	0.496	2.4756E-28	0
ARL2	1.20901E-32	0.31077843	0.655	0.606	4.0932E-28	0
NR2F6	1.32825E-32	0.30126865	0.639	0.581	4.4969E-28	0
SIPA1L2	7.05352E-32	0.29513781	0.275	0.16	2.388E-27	0
IGFBP2	1.26095E-30	0.3514039	0.495	0.38	4.2691E-26	0
FBL	1.65801E-29	0.26683823	0.721	0.707	5.6133E-25	0
PRKDC	3.03452E-29	0.25594985	0.825	0.837	1.0274E-24	0
DDAH2	2.77171E-28	0.32795219	0.489	0.395	9.3839E-24	0
PSME2	1.28317E-27	0.26193474	0.731	0.717	4.3443E-23	0
PXDN	7.67341E-27	0.27118307	0.846	0.864	2.5979E-22	0
UBE2I	3.09797E-26	0.2654058	0.602	0.544	1.0488E-21	0
ISG20	4.17867E-26	0.32595842	0.73	0.741	1.4147E-21	0
BANF1	5.08765E-25	0.2573226	0.713	0.698	1.7225E-20	0
LINC02762	1.80543E-24	0.27491386	0.752	0.744	6.1125E-20	0
RFXANK	2.12926E-24	0.28289218	0.485	0.404	7.2088E-20	0
CTD-2126E3.6	2.75182E-24	0.37543052	0.454	0.349	9.3166E-20	0
RNPS1	1.14413E-22	0.27398335	0.584	0.544	3.8736E-18	0
BCL3	1.97049E-22	0.2550468	0.368	0.271	6.6713E-18	0
PLD3	1.86603E-21	0.25909687	0.849	0.867	6.3176E-17	0
PNN	4.38133E-21	0.2543281	0.699	0.698	1.4833E-16	0
NASP	8.42665E-21	0.262061	0.572	0.509	2.8529E-16	0
CRISPLD1	1.94496E-20	0.26592353	0.418	0.33	6.5848E-16	0
ENO3	1.03999E-19	0.2982856	0.316	0.226	3.521E-15	0
ZNF428	1.17115E-19	0.26161601	0.616	0.59	3.965E-15	0
TMPO	2.64678E-19	0.25443236	0.514	0.435	8.9609E-15	0
EGFL7	3.13572E-19	0.2737153	0.486	0.421	1.0616E-14	0
NUDT14	2.81286E-18	0.25145927	0.505	0.45	9.5232E-14	0
S100A8	1.23928E-15	-0.5108261	0.066	0.151	4.1957E-11	0
TENT5A	1.36354E-14	0.27941104	0.553	0.522	4.6164E-10	0
LINC00632	3.81739E-14	0.26223222	0.3	0.23	1.2924E-09	0
FLRT2	3.1475E-13	0.26273519	0.404	0.343	1.0656E-08	0
SLC44A1	3.04985E-11	0.28006129	0.376	0.327	1.0326E-06	0
BCYRN1	3.26965E-11	0.3084688	0.407	0.34	1.107E-06	0
HMGB2	1.64069E-09	0.29282249	0.286	0.228	5.5547E-05	0
CKB	9.44678E-09	0.32621802	0.353	0.314	0.00031983	0
S100A4.1	6.6332E-213	0.61757377	0.999	0.947	2.246E-208	1
CEACAM6.1	7.9063E-161	0.61884817	0.683	0.298	2.677E-156	1
S100A16.1	5.1713E-156	0.52451201	0.939	0.614	1.751E-151	1
MUC1.1	6.7472E-150	0.51026023	0.989	0.836	2.284E-145	1
MT-ND4	4.5059E-148	-0.4899209	0.999	0.997	1.526E-143	1
MT-CO2	7.4536E-142	-0.4490249	1	0.998	2.524E-137	1
S100A6.1	8.4417E-132	0.31742596	1	0.999	2.858E-127	1
LGALS3BP.1	4.8788E-126	0.45873471	0.964	0.688	1.652E-121	1
RPL39	7.1211E-124	-0.6064525	0.998	0.997	2.411E-119	1
HLA-C.1	1.7549E-122	0.49089084	0.937	0.698	5.941E-118	1
RPS24	1.1145E-114	-0.5662558	0.991	0.981	3.773E-110	1
SPINT2	7.2534E-114	0.32086948	0.997	0.978	2.456E-109	1
RPL26	6.4963E-112	-0.496149	0.997	0.993	2.199E-107	1
NEAT1.1	2.9424E-110	0.4583012	0.995	0.974	9.962E-106	1
MT-CO1.1	5.6819E-107	-0.3135181	0.999	0.997	1.924E-102	1
TNS4.1	7.1282E-103	0.35088665	0.509	0.22	2.4133E-98	1
B2M.1	8.1586E-99	0.38506833	0.994	0.946	2.7622E-94	1
ATP5MJ	3.73714E-96	-0.4664825	0.881	0.896	1.2652E-91	1
MT-ND2	7.35049E-95	-0.476388	0.986	0.972	2.4886E-90	1
RPL34	1.83026E-93	-0.4927493	0.995	0.987	6.1965E-89	1
ALDH3A1.1	2.63861E-91	0.55841224	0.804	0.528	8.9333E-87	1
HLA-B.1	8.53816E-89	0.48258607	0.806	0.536	2.8907E-84	1
SYCE1L.1	1.14525E-87	0.37600748	0.935	0.726	3.8773E-83	1
SLPI.1	1.26576E-87	0.42766911	0.919	0.68	4.2853E-83	1
LGALS1.1	1.52462E-87	0.26719358	1	0.991	5.1618E-83	1
MT-RNR2	1.22461E-85	-0.3225545	0.997	0.997	4.146E-81	1
RPS6	2.16395E-84	-0.2560105	0.997	0.996	7.3263E-80	1
MT-RNR1	7.80631E-84	-0.4211308	0.995	0.993	2.6429E-79	1
MT-ATP6	4.02972E-83	-0.3908501	0.994	0.99	1.3643E-78	1
DUSP1.1	1.23266E-82	0.37219694	0.972	0.84	4.1733E-78	1
RPS15A.1	2.29239E-82	-0.3049306	0.984	0.986	7.7611E-78	1
CAMK2N1.1	1.19118E-81	0.42648894	0.91	0.699	4.0329E-77	1
PPP1R14B-AS1	9.50009E-76	0.30570194	0.48	0.239	3.2164E-71	1
C19orf33.1	2.98612E-75	0.3159128	0.956	0.828	1.011E-70	1
SLC2A1.1	6.37873E-75	0.33212018	0.731	0.457	2.1596E-70	1
NOTUM.1	6.47207E-75	0.37979905	0.887	0.606	2.1912E-70	1
GUK1.1	2.82377E-74	0.28158445	0.977	0.915	9.5602E-70	1
MAL2.1	4.17291E-74	0.32409872	0.58	0.321	1.4128E-69	1
HSP90AA1.1	7.65505E-74	-0.5952829	0.992	0.972	2.5917E-69	1
FIBCD1.1	1.90424E-72	0.31458597	0.56	0.311	6.447E-68	1
RPS3A	1.63009E-70	-0.4065486	0.858	0.881	5.5188E-66	1
RPL7	4.75854E-70	-0.4700984	0.869	0.885	1.6111E-65	1
SLC20A1.1	1.32982E-69	0.39658901	0.724	0.494	4.5022E-65	1
SERPINE2.1	4.59247E-68	0.39565255	0.672	0.432	1.5548E-63	1
CST3.1	4.76015E-68	0.26922306	0.997	0.966	1.6116E-63	1
CD24.1	6.11602E-68	0.31732463	0.654	0.384	2.0706E-63	1
CENPX.1	9.27676E-68	0.30026054	0.955	0.818	3.1407E-63	1
HLA-A.1	1.80037E-67	0.28548392	0.995	0.956	6.0953E-63	1
MALAT1.1	1.31816E-66	0.34589907	1	0.998	4.4627E-62	1
PTMA.1	1.0814E-65	-0.3256659	0.992	0.99	3.6612E-61	1
ITGB1	1.28856E-65	0.30269157	0.987	0.933	4.3626E-61	1
KRT18.1	3.04109E-65	0.33245132	0.993	0.96	1.0296E-60	1
LDHB	2.20903E-63	-0.3567576	0.798	0.853	7.4789E-59	1
LINC00963.1	6.46408E-63	0.348913	0.78	0.571	2.1885E-58	1
SUSD2.1	1.24738E-62	0.33161543	0.836	0.598	4.2231E-58	1
SCG2.1	2.89572E-62	-0.6930643	0.506	0.662	9.8037E-58	1
CYP24A1.1	7.04775E-62	0.28411373	0.976	0.893	2.3861E-57	1
LYPD3.1	1.29953E-61	0.31911898	0.689	0.441	4.3997E-57	1
S100A13.1	3.17195E-61	0.33128615	0.763	0.526	1.0739E-56	1
TGM2.1	2.86891E-60	0.31816787	0.839	0.602	9.713E-56	1
POLR2I	4.0259E-60	0.26628472	0.971	0.925	1.363E-55	1
RHOBTB3.1	9.43037E-60	0.2995892	0.87	0.648	3.1927E-55	1
SLC26A2.1	7.15818E-57	0.3222477	0.935	0.78	2.4235E-52	1
HSP90AB1	1.86702E-56	-0.3169488	0.985	0.967	6.321E-52	1
ASPSCR1.1	2.10487E-56	0.29278957	0.799	0.592	7.1263E-52	1
P4HB	3.50072E-56	0.25976893	0.989	0.938	1.1852E-51	1
IL18.1	1.14363E-55	0.27206216	0.879	0.683	3.8719E-51	1
FGFBP1.1	1.6703E-55	0.30973808	0.491	0.271	5.655E-51	1
CYB5B.1	5.39073E-54	0.29898943	0.81	0.64	1.8251E-49	1
MT-ND5	8.67945E-54	-0.2678463	0.995	0.981	2.9385E-49	1
VTN	6.06803E-53	0.2810726	0.37	0.181	2.0544E-48	1
KRT8.1	2.29721E-52	0.3017509	0.98	0.934	7.7774E-48	1
JUNB.1	2.29938E-52	0.29990135	0.889	0.734	7.7848E-48	1
SNHG25.1	5.9376E-51	0.29685077	0.885	0.707	2.0102E-46	1
EPHA2.1	4.08422E-48	0.28107835	0.605	0.403	1.3828E-43	1
HSPE1	9.45077E-48	-0.3744825	0.716	0.768	3.1997E-43	1
S100A14.1	9.04152E-47	0.28777208	0.357	0.179	3.0611E-42	1
CTSD.1	2.71501E-46	0.25375561	0.925	0.823	9.1919E-42	1
NPC2.1	3.60343E-46	0.26440177	0.829	0.658	1.22E-41	1
DAP.1	1.43201E-45	0.25435124	0.798	0.625	4.8482E-41	1
S100A9.1	7.55048E-43	-0.4004028	0.784	0.569	2.5563E-38	1
CCND1.1	8.50863E-42	0.25995222	0.831	0.662	2.8807E-37	1
NCL.1	6.75029E-41	-0.3541295	0.916	0.907	2.2854E-36	1
HTRA1.1	3.24629E-40	0.28349396	0.528	0.351	1.0991E-35	1
AMOTL2.1	2.9483E-39	0.25568268	0.688	0.509	9.9818E-35	1
GM2A.1	7.43397E-39	0.26066358	0.68	0.492	2.5168E-34	1
MTND4P12	3.57484E-38	-0.3247922	0.642	0.716	1.2103E-33	1
TIMP2	5.05888E-38	0.25135473	0.686	0.507	1.7127E-33	1
MARCKSL1.1	2.77096E-37	-0.3224686	0.302	0.484	9.3814E-33	1
MAP2.1	9.82177E-37	-0.3853954	0.226	0.401	3.3253E-32	1
IER3	1.10773E-35	0.2586225	0.855	0.715	3.7503E-31	1
STMN1.1	3.52451E-35	-0.3513138	0.762	0.814	1.1933E-30	1
HSPD1	3.5771E-34	-0.3211126	0.797	0.805	1.2111E-29	1
MT-ND4L	5.19004E-31	-0.3391531	0.711	0.751	1.7571E-26	1
LUCAT1.1	5.32582E-31	0.26525027	0.674	0.496	1.8031E-26	1
NDRG1.1	1.56173E-30	0.27570422	0.721	0.574	5.2874E-26	1
CENPV.1	2.54158E-26	-0.2890999	0.379	0.514	8.6048E-22	1
HMGB1	5.0428E-24	-0.2547857	0.8	0.819	1.7073E-19	1
PTTG1	4.1924E-23	-0.4362211	0.26	0.391	1.4194E-18	1
RASSF8	9.2368E-23	-0.2665499	0.446	0.56	3.1272E-18	1
HSPB1.1	1.82343E-22	-0.2527062	0.888	0.898	6.1734E-18	1
SLIRP	3.83688E-22	-0.2532899	0.938	0.924	1.299E-17	1
SUPT16H	2.16157E-21	-0.2644027	0.635	0.696	7.3182E-17	1
AHSA1	2.33514E-21	-0.2624053	0.555	0.638	7.9058E-17	1
SIVA1.1	2.2732E-20	-0.2751689	0.68	0.73	7.6961E-16	1
TENT5A.1	3.00791E-20	-0.2843562	0.433	0.544	1.0184E-15	1
CENPF	1.4391E-18	-0.3215755	0.235	0.361	4.8722E-14	1
EDIL3.1	1.62833E-18	-0.2860008	0.42	0.522	5.5129E-14	1
DLGAP5	1.61809E-17	-0.2565763	0.114	0.222	5.4782E-13	1
CTD-2126E3.6.1	2.77744E-17	-0.3465115	0.273	0.383	9.4033E-13	1
CDKN3	1.94237E-16	-0.2568028	0.25	0.361	6.5761E-12	1
HMGN2	5.93502E-16	-0.2616074	0.689	0.726	2.0094E-11	1
PCLAF	2.46553E-15	-0.2606689	0.24	0.348	8.3473E-11	1
VGF.1	4.83361E-14	-0.3302908	0.042	0.113	1.6365E-09	1
BCYRN1.1	1.11247E-11	-0.3625405	0.28	0.363	3.7664E-07	1
TUBA1B	3.11363E-11	-0.3085608	0.73	0.751	1.0542E-06	1
H2AZ1	3.6754E-11	-0.3214506	0.791	0.769	1.2443E-06	1
H4C3	3.31954E-08	-0.3799254	0.679	0.698	0.00112386	1
HES1	1.22415E-07	-0.2534151	0.472	0.523	0.00414447	1
GAS5.1	0	0.8267284	0.996	0.798	0	2
RPL34.1	0	0.76395737	1	0.986	0	2
RPL26.1	0	0.67439926	1	0.992	0	2
RPL39.1	1.6389E-307	0.6998358	1	0.997	5.549E-303	2
RPS24.1	5.2839E-282	0.66853131	1	0.98	1.789E-277	2
RPL23.1	1.0653E-273	0.55327534	1	0.988	3.607E-269	2
RPL35A	1.7613E-270	0.41612959	1	0.997	5.963E-266	2
RPS27	4.0978E-267	0.4051903	1	0.999	1.387E-262	2
RPS27A	9.5342E-224	0.37267909	1	0.995	3.228E-219	2
RPL7.1	2.4593E-210	0.62682757	0.99	0.865	8.326E-206	2
RPS4Y1.1	1.2612E-209	0.35147808	1	0.998	4.27E-205	2
MT-ATP6.1	1.0435E-206	0.45321248	1	0.99	3.533E-202	2
RPL10A	1.7786E-205	0.41304608	0.999	0.987	6.022E-201	2
RPS3A.1	8.5779E-193	0.54268505	0.994	0.859	2.904E-188	2
S100A11	1.6621E-176	-0.622639	0.989	0.986	5.627E-172	2
RPL24	5.4538E-176	0.34462952	1	0.986	1.846E-171	2
EEF1A1	2.6595E-170	0.29322478	1	1	9.004E-166	2
MT-RNR1.1	1.4101E-164	0.43621098	1	0.992	4.774E-160	2
GAPDH	1.6071E-163	-0.3918838	1	0.999	5.441E-159	2
RPS6.1	2.3097E-154	0.30040364	1	0.995	7.82E-150	2
RPL13A	1.0672E-148	0.35019586	0.999	0.975	3.613E-144	2
ACTB	7.0277E-144	-0.4573608	0.999	0.997	2.379E-139	2
TFPI2.1	9.7235E-140	-0.6174938	0.983	0.98	3.292E-135	2
IGFBP5.1	6.2567E-138	0.5230168	0.812	0.406	2.118E-133	2
PFN1.1	4.0889E-137	-0.484686	0.971	0.976	1.384E-132	2
MALAT1.2	9.6841E-136	0.43654124	1	0.998	3.279E-131	2
MT-ND2.1	6.3265E-129	0.36490572	1	0.97	2.142E-124	2
PABPC1	1.0089E-124	0.3349767	0.998	0.965	3.416E-120	2
TPI1	4.0544E-122	-0.5270953	0.915	0.922	1.373E-117	2
TPD52L1.1	8.6812E-122	0.42507975	0.917	0.616	2.939E-117	2
RPS25	2.1198E-119	0.30924262	0.998	0.965	7.177E-115	2
SH3BP5.1	4.2609E-118	0.36201465	0.549	0.214	1.443E-113	2
RPL27A	2.1285E-111	0.2999057	0.999	0.975	7.206E-107	2
NOTUM.2	3.3523E-111	0.45817915	0.935	0.602	1.135E-106	2
CCL2.1	5.3612E-108	0.36552488	0.684	0.321	1.815E-103	2
ID2.1	1.5124E-104	0.412982	0.863	0.547	5.121E-100	2
PSMA7	4.8755E-99	-0.3790171	0.986	0.981	1.6507E-94	2
GPX1	1.1419E-98	-0.4377035	0.936	0.927	3.866E-94	2
TAGLN2	1.16693E-98	-0.5567428	0.858	0.883	3.9508E-94	2
CTSL	2.72094E-98	-0.8091346	0.703	0.779	9.212E-94	2
SELENOP.1	2.48874E-97	0.33897764	0.834	0.473	8.4259E-93	2
SNHG5	1.53874E-96	0.29714606	0.996	0.929	5.2096E-92	2
EEF2	1.85497E-94	0.25274844	1	0.983	6.2802E-90	2
NEAT1.2	7.06672E-94	0.32963383	1	0.974	2.3925E-89	2
APLP2.1	1.05161E-91	0.31412179	0.996	0.927	3.5603E-87	2
PFDN2	5.26386E-90	-0.3893017	0.977	0.963	1.7821E-85	2
SLIRP.1	2.72707E-88	-0.4675985	0.936	0.925	9.2328E-84	2
HSP90AA1.2	3.40523E-88	-0.6375371	0.989	0.973	1.1529E-83	2
FKBP5	4.80322E-88	0.27038651	0.464	0.188	1.6262E-83	2
C15orf48	2.76749E-87	-0.6539627	0.876	0.874	9.3696E-83	2
SNTB2	4.90546E-84	0.34276365	0.929	0.694	1.6608E-79	2
TSC22D3.1	7.06916E-84	0.39327002	0.847	0.575	2.3933E-79	2
CDKN1C.1	3.18963E-83	0.32079416	0.556	0.26	1.0797E-78	2
UQCR10	6.58873E-83	-0.3643061	0.949	0.939	2.2307E-78	2
HLA-A.2	1.72412E-82	0.28876225	0.998	0.956	5.8372E-78	2
RPL6	1.03767E-81	0.29192771	0.982	0.888	3.5131E-77	2
BASP1.1	3.26136E-81	0.31923616	0.992	0.923	1.1042E-76	2
DSP.1	1.4742E-80	0.34834506	0.998	0.952	4.991E-76	2
PTPRR.1	1.79607E-80	0.285611	0.579	0.281	6.0808E-76	2
DUSP1.2	2.57091E-80	0.34247279	0.979	0.84	8.7041E-76	2
EIF5A	6.7717E-80	-0.3974083	0.932	0.929	2.2926E-75	2
ID1.1	8.93444E-80	0.29649166	0.994	0.909	3.0248E-75	2
RPL36A	7.96473E-79	0.25035372	0.995	0.96	2.6965E-74	2
EPB41L4A-AS1	1.93844E-78	0.32461629	0.85	0.594	6.5628E-74	2
SPP1.1	4.75742E-78	-0.5261388	0.987	0.972	1.6107E-73	2
GLG1.1	1.91472E-76	0.29517007	0.987	0.892	6.4825E-72	2
RPL22	3.14801E-75	0.26169202	0.992	0.927	1.0658E-70	2
RPS20	9.53004E-75	0.29477411	0.992	0.902	3.2265E-70	2
MZT2B	1.5206E-74	-0.333866	0.958	0.949	5.1481E-70	2
CIB1	1.39214E-73	-0.4039798	0.867	0.869	4.7132E-69	2
H2AZ1.1	1.43762E-73	-0.8008154	0.748	0.776	4.8672E-69	2
CHCHD2.1	3.00961E-73	-0.3670968	0.959	0.94	1.0189E-68	2
YPEL5.1	8.92545E-71	0.33059836	0.876	0.644	3.0218E-66	2
ID3.1	4.87678E-70	0.30187788	0.994	0.927	1.6511E-65	2
ATP5MF	2.75033E-69	-0.3393895	0.932	0.924	9.3115E-65	2
ZKSCAN1	3.3884E-69	0.30700647	0.861	0.591	1.1472E-64	2
CALR	3.8953E-69	-0.3303209	0.96	0.942	1.3188E-64	2
FOSB	7.67529E-69	0.35185557	0.936	0.765	2.5985E-64	2
TACC2.1	8.67545E-68	0.29866366	0.73	0.454	2.9372E-63	2
ABCC3.1	9.04793E-68	0.29506295	0.945	0.766	3.0633E-63	2
HSPA9	4.06194E-65	0.3365457	0.951	0.854	1.3752E-60	2
RNF19A.1	9.09832E-65	0.2984738	0.812	0.569	3.0803E-60	2
TUBA1A	2.18735E-64	0.28359038	0.635	0.356	7.4055E-60	2
TGFBI.1	3.85919E-64	0.30464028	0.728	0.429	1.3066E-59	2
RPL21	2.10868E-63	0.30226874	0.775	0.519	7.1392E-59	2
HMGA1.1	3.01504E-63	-0.4751748	0.522	0.663	1.0208E-58	2
CHMP1B	4.04875E-62	0.27136293	0.983	0.928	1.3707E-57	2
RANBP1	1.84228E-61	-0.4940443	0.74	0.771	6.2372E-57	2
SUSD2.2	2.04999E-61	0.26461786	0.856	0.628	6.9405E-57	2
CDKN1A.1	4.02748E-61	0.31065406	0.664	0.406	1.3635E-56	2
RHOBTB3.2	1.84482E-60	0.29308707	0.887	0.648	6.2458E-56	2
SIVA1.2	4.00741E-60	-0.4596363	0.667	0.732	1.3567E-55	2
NRIP1.1	1.72725E-59	0.26790455	0.607	0.351	5.8478E-55	2
NDUFS6	3.46886E-59	-0.2632771	0.982	0.971	1.1744E-54	2
ZFAND5.1	7.39586E-59	0.28108833	0.879	0.665	2.5039E-54	2
ELOB	1.0513E-58	-0.262596	0.98	0.97	3.5593E-54	2
ALDH3B1.1	1.75429E-58	0.26974575	0.726	0.459	5.9393E-54	2
SCEL.1	2.32503E-58	0.26376991	0.56	0.305	7.8716E-54	2
LAMB1	2.59102E-58	0.27719511	0.848	0.628	8.7722E-54	2
CD320	2.98135E-58	-0.4017922	0.762	0.814	1.0094E-53	2
CYC1	3.41774E-58	-0.3700583	0.841	0.845	1.1571E-53	2
HES1.1	6.6863E-58	0.49156022	0.703	0.485	2.2637E-53	2
MAP1LC3B.1	7.32956E-58	0.3009652	0.876	0.687	2.4815E-53	2
CAST.1	1.41804E-57	0.26970482	0.971	0.886	4.8009E-53	2
PDLIM1.1	2.01891E-57	0.27921437	0.644	0.381	6.8352E-53	2
COTL1	1.00516E-56	-0.5082103	0.277	0.473	3.4031E-52	2
JUN.1	2.13517E-56	0.33975571	0.733	0.482	7.2288E-52	2
RAB31.1	1.03734E-55	0.26659867	0.888	0.69	3.512E-51	2
ZNF503.1	1.13966E-55	0.30972038	0.707	0.461	3.8584E-51	2
SNHG8	1.7522E-55	0.2647566	0.862	0.662	5.9322E-51	2
CASC15	1.09765E-54	0.27279188	0.936	0.819	3.7162E-50	2
PSMB6	1.3369E-54	-0.3097557	0.925	0.918	4.5262E-50	2
RAN	2.65578E-54	-0.2804244	0.983	0.96	8.9914E-50	2
YBX1	1.71976E-53	-0.2888888	0.958	0.938	5.8224E-49	2
FTH1.1	6.49742E-53	-0.318814	1	0.998	2.1998E-48	2
EMP3	7.17483E-53	-0.3016169	0.926	0.923	2.4291E-48	2
MRPL52.1	1.83683E-52	-0.3633664	0.858	0.85	6.2188E-48	2
CIAO2B	3.31364E-52	-0.2839188	0.938	0.918	1.1219E-47	2
TIMP1.1	5.45632E-52	-0.5461269	0.993	0.987	1.8473E-47	2
IL18.2	3.50496E-51	0.25943513	0.892	0.683	1.1866E-46	2
PCLAF.1	4.53282E-50	-0.4665848	0.144	0.362	1.5346E-45	2
PTMA.2	8.0764E-50	-0.3053376	0.998	0.989	2.7343E-45	2
PHF19	2.20784E-49	-0.4328861	0.154	0.364	7.4749E-45	2
AP2S1	6.28036E-49	-0.2685977	0.951	0.914	2.1263E-44	2
BIRC5	6.4018E-49	-0.425514	0.096	0.31	2.1674E-44	2
PRDX5	2.95348E-48	-0.2868002	0.941	0.932	9.9993E-44	2
CPS1	3.99854E-48	-0.4208957	0.616	0.703	1.3537E-43	2
PRKDC.1	5.31965E-48	-0.3602898	0.836	0.835	1.801E-43	2
STMN1.2	2.75782E-47	-0.4625493	0.812	0.805	9.3369E-43	2
PDE10A	3.30581E-47	0.26190264	0.817	0.605	1.1192E-42	2
PTTG1.1	6.34778E-47	-0.5862228	0.197	0.4	2.1491E-42	2
STC1	2.69188E-46	-0.7217382	0.337	0.506	9.1136E-42	2
ACTN1	4.26157E-46	-0.3869471	0.504	0.622	1.4428E-41	2
TYMS	4.47572E-46	-0.378086	0.086	0.286	1.5153E-41	2
HMGB1.1	5.25172E-46	-0.3845394	0.812	0.817	1.778E-41	2
LSM4	5.45576E-46	-0.358753	0.696	0.749	1.8471E-41	2
NUDT1	6.52463E-46	-0.3481534	0.153	0.355	2.209E-41	2
DYNLL1	6.82288E-46	-0.3025381	0.936	0.908	2.31E-41	2
SNRPG	7.48672E-46	-0.3303227	0.847	0.836	2.5347E-41	2
JPT1	8.74255E-45	-0.3731935	0.648	0.712	2.9599E-40	2
HNRNPM	2.13899E-44	-0.3421017	0.815	0.808	7.2418E-40	2
UQCRB	2.40508E-44	0.30334385	0.909	0.784	8.1427E-40	2
SDC1	6.70971E-44	-0.4150033	0.541	0.645	2.2716E-39	2
SNRPF	7.16375E-44	-0.3487532	0.743	0.768	2.4254E-39	2
DBI	8.57681E-44	-0.3654446	0.573	0.664	2.9038E-39	2
NCL.2	3.36727E-42	-0.3774327	0.926	0.906	1.14E-37	2
ERH	6.31025E-42	-0.2820556	0.942	0.913	2.1364E-37	2
DPM3	1.02239E-41	-0.3039611	0.895	0.886	3.4614E-37	2
TOP2A	1.0872E-41	-0.6532479	0.168	0.358	3.6808E-37	2
TPM3	1.1364E-40	-0.2692698	0.981	0.95	3.8474E-36	2
AURKAIP1	1.81555E-40	-0.2626596	0.906	0.889	6.1467E-36	2
CALM2	5.71787E-40	-0.3034357	0.957	0.922	1.9358E-35	2
POMP	9.09201E-40	-0.2630244	0.899	0.872	3.0782E-35	2
CENPW	1.49316E-39	-0.3034319	0.098	0.279	5.0553E-35	2
HNRNPC	1.71504E-39	-0.2866616	0.895	0.871	5.8064E-35	2
PPP1R14B	5.4044E-39	-0.2622569	0.893	0.899	1.8297E-34	2
FABP5	6.77276E-39	-0.3682091	0.324	0.484	2.293E-34	2
HMGN1	9.78051E-39	-0.284768	0.863	0.858	3.3113E-34	2
HNRNPA2B1	1.40686E-38	-0.2570837	0.96	0.935	4.7631E-34	2
BCYRN1.2	3.58476E-38	-0.5473879	0.195	0.376	1.2137E-33	2
PTGES3	4.49901E-38	-0.3460034	0.651	0.698	1.5232E-33	2
SDF2L1	5.89303E-38	-0.359486	0.483	0.595	1.9951E-33	2
MYBL2	1.10238E-37	-0.2991368	0.064	0.232	3.7322E-33	2
APOC1.1	1.16887E-37	-0.3612386	0.309	0.484	3.9573E-33	2
CALM3	1.25407E-37	-0.361107	0.617	0.676	4.2458E-33	2
COX17	4.71987E-37	-0.3400603	0.673	0.719	1.598E-32	2
CDKN3.1	6.7029E-37	-0.3975024	0.193	0.369	2.2693E-32	2
TLE5.1	8.63603E-37	-0.3227764	0.619	0.701	2.9238E-32	2
UBE2S	8.93102E-37	-0.5151439	0.682	0.712	3.0237E-32	2
CCT5	1.1537E-36	-0.266643	0.964	0.939	3.906E-32	2
PCNX4	1.42072E-36	0.25988745	0.734	0.534	4.81E-32	2
ASPH	1.48836E-36	-0.3413065	0.705	0.727	5.039E-32	2
COPS9	3.49046E-36	-0.2577381	0.918	0.888	1.1817E-31	2
MKI67	4.09643E-36	-0.4664827	0.095	0.267	1.3869E-31	2
H3-3B.1	9.35242E-36	-0.2522382	0.947	0.927	3.1664E-31	2
MDH2	1.54735E-35	-0.2992448	0.794	0.789	5.2387E-31	2
RRM2	2.21101E-35	-0.4480341	0.095	0.26	7.4856E-31	2
HSPB1.2	5.0382E-35	-0.3119358	0.906	0.895	1.7057E-30	2
BOLA3	5.53027E-35	-0.3104936	0.682	0.715	1.8723E-30	2
UPP1	7.15152E-35	-0.2588554	0.095	0.259	2.4212E-30	2
TXNDC17	3.07201E-34	-0.2644858	0.808	0.82	1.0401E-29	2
CALM1	5.34822E-34	-0.3194169	0.991	0.974	1.8107E-29	2
HMGN2.1	6.44034E-34	-0.4179581	0.715	0.721	2.1804E-29	2
PGAM1	6.76605E-34	-0.3093848	0.724	0.719	2.2907E-29	2
TUBA1B.1	7.19852E-34	-0.6340641	0.76	0.746	2.4371E-29	2
DLGAP5.1	2.19695E-33	-0.3546495	0.069	0.228	7.438E-29	2
CITED4	4.75539E-33	-0.2714881	0.061	0.21	1.61E-28	2
DTYMK	8.38368E-33	-0.3159978	0.202	0.364	2.8384E-28	2
PSMD2	6.96167E-32	-0.2553064	0.847	0.823	2.3569E-27	2
EIF3J	1.62741E-31	-0.2819244	0.804	0.806	5.5098E-27	2
PDIA4.1	2.60944E-31	-0.3056679	0.68	0.718	8.8345E-27	2
ALYREF	3.88535E-31	-0.322503	0.54	0.616	1.3154E-26	2
PBK	5.07258E-31	-0.2983016	0.079	0.229	1.7174E-26	2
S100A9.2	5.49062E-31	-1.7096249	0.541	0.61	1.8589E-26	2
SNRPB	1.41964E-30	-0.324454	0.601	0.65	4.8063E-26	2
PSMB3	3.02871E-30	-0.2795948	0.834	0.826	1.0254E-25	2
PRELID1	4.54546E-30	-0.2627057	0.777	0.779	1.5389E-25	2
PHB	8.88628E-30	-0.2988777	0.677	0.717	3.0085E-25	2
PA2G4	1.05895E-29	-0.2733475	0.848	0.816	3.5852E-25	2
SNRPD1	1.07596E-29	-0.298786	0.757	0.76	3.6428E-25	2
TOP1	1.24892E-29	-0.2837606	0.864	0.85	4.2283E-25	2
LSM7	8.54334E-29	-0.2683039	0.766	0.773	2.8924E-24	2
MYDGF	1.04572E-28	-0.294423	0.667	0.693	3.5404E-24	2
PHLDA2	1.51617E-28	-0.3024405	0.362	0.49	5.1331E-24	2
DAD1	1.63811E-28	-0.2588602	0.82	0.818	5.546E-24	2
ATP5MC1	2.66824E-28	-0.2536015	0.811	0.801	9.0336E-24	2
DUT	3.46651E-28	-0.3954935	0.604	0.64	1.1736E-23	2
SERPINE2.2	4.76809E-28	-0.435624	0.374	0.482	1.6143E-23	2
MICOS10	2.62331E-27	-0.2529952	0.759	0.762	8.8815E-23	2
MAD2L1	2.99239E-27	-0.2538271	0.106	0.249	1.0131E-22	2
BANF1.1	5.89542E-27	-0.2817255	0.668	0.706	1.996E-22	2
C1QBP	8.38991E-27	-0.2601261	0.876	0.842	2.8405E-22	2
FXYD5.1	5.72784E-26	-0.3670277	0.527	0.594	1.9392E-21	2
LY6E	6.75282E-26	-0.2509588	0.875	0.858	2.2862E-21	2
CCNB1	1.43776E-25	-0.377553	0.164	0.305	4.8677E-21	2
PMEPA1	2.1681E-25	-0.302555	0.738	0.752	7.3403E-21	2
NASP.1	4.32971E-25	-0.3344455	0.443	0.532	1.4659E-20	2
H2AZ2	3.1457E-24	-0.2772612	0.656	0.674	1.065E-19	2
HNRNPAB	3.42719E-24	-0.2885558	0.784	0.772	1.1603E-19	2
CDC20	4.22621E-24	-0.3069649	0.11	0.243	1.4308E-19	2
PSMD14	4.4089E-24	-0.250886	0.746	0.745	1.4927E-19	2
CYTOR	1.0289E-23	-0.2992861	0.591	0.639	3.4835E-19	2
DDX21	3.00818E-23	-0.2514389	0.786	0.778	1.0185E-18	2
DKK1	3.5581E-23	-0.2544695	0.138	0.267	1.2046E-18	2
NME4.1	4.51011E-23	-0.2555783	0.758	0.767	1.5269E-18	2
TPX2	4.94233E-23	-0.3168197	0.147	0.278	1.6733E-18	2
UBE2C	5.41912E-23	-0.4060334	0.113	0.24	1.8347E-18	2
IGFBP4	6.02933E-23	-0.3445814	0.459	0.536	2.0413E-18	2
CENPV.2	1.99445E-21	-0.2843422	0.407	0.508	6.7524E-17	2
NUDC	2.9976E-21	-0.2506053	0.63	0.659	1.0149E-16	2
IGFBP3	3.10211E-21	-0.417972	0.07	0.18	1.0503E-16	2
CDCA4	3.70185E-21	-0.2680738	0.181	0.302	1.2533E-16	2
COMMD4	1.05716E-20	-0.256671	0.635	0.647	3.5791E-16	2
TUBB4B	2.05498E-20	-0.3533446	0.716	0.702	6.9573E-16	2
DNMT1	6.05336E-20	-0.3030356	0.35	0.44	2.0494E-15	2
CENPF.1	2.68193E-19	-0.3824611	0.248	0.357	9.08E-15	2
ISG20.1	4.40466E-19	-0.3240306	0.753	0.737	1.4912E-14	2
CREM.1	5.97753E-19	-0.2606947	0.275	0.382	2.0238E-14	2
FDPS	7.84053E-19	-0.2616341	0.811	0.786	2.6545E-14	2
MCM7	1.16507E-18	-0.3340076	0.368	0.446	3.9445E-14	2
ANP32E	2.32858E-18	-0.3205383	0.494	0.542	7.8836E-14	2
MT2A.1	7.99466E-17	-0.3803651	0.999	0.984	2.7067E-12	2
HMGB2.1	1.51782E-16	-0.2944028	0.148	0.252	5.1387E-12	2
VGF.2	6.967E-16	-0.3327455	0.035	0.113	2.3587E-11	2
AHSA1.1	8.25402E-16	-0.250634	0.613	0.628	2.7945E-11	2
DEK	2.93497E-15	-0.2969479	0.678	0.662	9.9366E-11	2
ATAD2	1.09177E-14	-0.3012962	0.204	0.291	3.6963E-10	2
S100P.1	1.32897E-14	-0.2930221	0.076	0.162	4.4994E-10	2
H1-10	2.61156E-14	-0.2648997	0.596	0.621	8.8417E-10	2
SCG2.2	1.08713E-13	-0.4405742	0.638	0.639	3.6806E-09	2
RSPO3	2.15081E-11	-0.2575645	0.458	0.493	7.2818E-07	2
TUBA1C	3.80572E-11	-0.2675407	0.725	0.687	1.2885E-06	2
AREG	1.3813E-10	-0.4703356	0.664	0.64	4.6765E-06	2
FERMT1.1	1.55448E-10	-0.3429723	0.439	0.469	5.2628E-06	2
TGM2.2	4.98044E-10	-0.5575676	0.698	0.627	1.6862E-05	2
CTD-2126E3.6.2	1.39237E-07	-0.2971602	0.323	0.374	0.00471401	2
S100A8.1	3.47412E-07	-0.6098027	0.092	0.143	0.01176199	2
H4C3.1	9.61615E-05	-0.6059283	0.748	0.686	1	2
MT-ND4.1	3.7301E-265	-0.680187	0.999	0.998	1.263E-260	3
MT-CO2.1	2.5364E-246	-0.6356847	1	0.998	8.587E-242	3
IGFBP5.2	2.3963E-201	0.8757812	0.804	0.41	8.113E-197	3
MT-CO1.2	5.4348E-188	-0.4451714	0.999	0.997	1.84E-183	3
ID1.2	4.8134E-180	0.55475745	0.998	0.909	1.63E-175	3
MALAT1.3	1.5996E-176	0.57986877	1	0.998	5.416E-172	3
SNHG29.1	4.4238E-167	0.37899624	0.999	0.995	1.498E-162	3
RPL10	3.2194E-164	0.28855287	0.999	0.998	1.09E-159	3
TFPI2.2	4.3545E-155	-0.6727946	0.958	0.983	1.474E-150	3
HSP90AA1.3	2.4954E-153	-0.805989	0.953	0.979	8.449E-149	3
ID3.2	4.661E-147	0.48996526	0.998	0.927	1.578E-142	3
MT-CYB	6.8263E-146	-0.3969743	1	0.998	2.311E-141	3
CST3.2	2.6204E-145	0.43312082	0.996	0.967	8.872E-141	3
MT-ND2.2	6.2882E-143	-0.5996095	0.973	0.974	2.129E-138	3
S100A11.1	4.4103E-131	-0.5550404	0.978	0.988	1.493E-126	3
EEF1A1.1	1.0192E-128	0.26237518	1	1	3.451E-124	3
NOTUM.3	7.1511E-127	0.57189995	0.899	0.609	2.421E-122	3
MT-RNR2.1	1.8309E-125	-0.399828	1	0.996	6.199E-121	3
MSLN.1	5.7967E-125	0.53851688	0.877	0.655	1.963E-120	3
ATP5MJ.1	1.421E-123	-0.5571431	0.765	0.914	4.811E-119	3
PPIA	4.6006E-121	-0.4155564	0.961	0.981	1.558E-116	3
CHMP1B.1	1.9804E-119	0.46167466	0.986	0.927	6.705E-115	3
MT-CO3	7.0331E-119	-0.2675563	1	0.998	2.381E-114	3
ID2.2	1.4803E-115	0.57784793	0.8	0.559	5.012E-111	3
RACK1	1.1782E-113	0.25837797	1	0.997	3.989E-109	3
CTSL.1	3.5782E-113	-0.8301651	0.561	0.8	1.211E-108	3
BASP1.2	1.6804E-109	0.40537019	0.987	0.925	5.689E-105	3
FTH1.2	4.9269E-108	-0.4368341	1	0.998	1.668E-103	3
DUSP1.3	1.1025E-107	0.46949942	0.97	0.843	3.733E-103	3
MT-ATP6.2	2.2675E-107	-0.4594565	0.997	0.99	7.677E-103	3
TPD52L1.2	2.4122E-106	0.5117299	0.83	0.631	8.167E-102	3
TPI1.1	2.3546E-103	-0.5041413	0.82	0.937	7.972E-99	3
SPP1.2	2.88372E-98	-0.5847632	0.948	0.979	9.7631E-94	3
SELENOP.2	8.37583E-98	0.49218574	0.738	0.49	2.8357E-93	3
TXN	2.51796E-95	-0.3651306	0.943	0.975	8.5248E-91	3
ACTB.1	8.40395E-93	-0.3771971	0.997	0.997	2.8452E-88	3
C15orf48.1	1.95865E-92	-0.6473462	0.772	0.89	6.6312E-88	3
SLIRP.2	5.93056E-89	-0.4829287	0.865	0.935	2.0078E-84	3
EEF2.1	1.00009E-88	0.29353146	0.996	0.984	3.3859E-84	3
LDHA.1	4.72143E-88	-0.3904765	0.906	0.947	1.5985E-83	3
S100A9.3	1.73373E-87	-2.0659284	0.356	0.638	5.8697E-83	3
FOSB.1	4.05795E-87	0.51295029	0.896	0.772	1.3739E-82	3
HLA-A.3	9.65469E-85	0.31329237	0.995	0.956	3.2687E-80	3
LAPTM4A	2.14142E-83	0.31168496	0.985	0.947	7.25001E-79	3
MT-RNR1.2	2.74569E-83	-0.4448595	0.997	0.993	9.29582E-79	3
GPX1.1	4.8953E-82	-0.4086392	0.851	0.94	1.65735E-77	3
NEAT1.3	6.03628E-82	0.34411939	0.996	0.974	2.04364E-77	3
COTL1.1	2.0565E-81	-0.5300068	0.166	0.488	6.96248E-77	3
MT-ND5.1	3.42728E-81	-0.346336	0.987	0.982	1.16034E-76	3
KCNF1.1	2.70768E-80	0.4500267	0.775	0.587	9.16712E-76	3
TAGLN2.1	2.86202E-80	-0.506929	0.766	0.897	9.68964E-76	3
CIRBP	3.22831E-78	0.36737997	0.912	0.815	1.09298E-73	3
MTND4P12.1	2.06E-77	-0.4616196	0.475	0.74	6.97433E-73	3
RPL10A.1	6.06337E-76	0.2561611	0.996	0.987	2.05281E-71	3
MUC1.2	2.23698E-74	0.33939188	0.98	0.84	7.57352E-70	3
SCG2.3	1.5881E-72	-0.686087	0.399	0.676	5.37668E-68	3
HSP90AB1.1	2.33908E-72	-0.371921	0.968	0.97	7.91918E-68	3
PFDN2.1	3.70095E-72	-0.3518483	0.938	0.967	1.25299E-67	3
PFN1.2	5.25865E-72	-0.3624305	0.961	0.978	1.78037E-67	3
EGR1	5.28447E-72	0.39554575	0.903	0.794	1.78911E-67	3
CFD	1.39888E-70	0.39410015	0.838	0.69	4.73604E-66	3
PSMA7.1	2.9756E-70	-0.3257798	0.972	0.983	1.00742E-65	3
H2AZ1.2	8.44855E-70	-0.7417487	0.632	0.794	2.86034E-65	3
TIMP1.2	1.0386E-69	-0.5962825	0.968	0.991	3.51628E-65	3
MRPL52.2	9.87092E-69	-0.4073836	0.714	0.872	3.3419E-64	3
TMSB4X	1.2794E-68	-0.2920427	0.981	0.987	4.33155E-64	3
APLP2.2	1.44192E-68	0.28583707	0.984	0.929	4.88175E-64	3
HSPE1.1	6.90062E-68	-0.4506233	0.564	0.791	2.33627E-63	3
PDIA6	8.46117E-68	-0.3176955	0.929	0.96	2.86461E-63	3
HMGA1.2	1.30673E-67	-0.460477	0.403	0.68	4.42407E-63	3
MT-ND4L.1	4.76484E-67	-0.470815	0.56	0.773	1.61319E-62	3
CCL2.2	6.47544E-67	0.45588458	0.564	0.341	2.19232E-62	3
TUBA1A.1	1.25856E-66	0.43658347	0.575	0.367	4.26097E-62	3
ATOH8.1	2.20701E-66	0.34483423	0.404	0.195	7.47206E-62	3
GAPDH.1	5.23701E-66	-0.2594391	0.999	0.999	1.77304E-61	3
CYP24A1.2	1.63734E-65	0.32208408	0.971	0.895	5.54338E-61	3
SYCE1L.2	5.80007E-65	0.37694147	0.888	0.737	1.96367E-60	3
PTTG1.2	1.15838E-64	-0.6885131	0.138	0.407	3.92181E-60	3
TPM3.1	1.95038E-64	-0.3295001	0.937	0.957	6.60322E-60	3
STMN1.3	3.52481E-64	-0.5014328	0.66	0.828	1.19336E-59	3
STC1.1	6.99017E-64	-0.7404996	0.245	0.519	2.36659E-59	3
CHCHD2.2	4.35243E-63	-0.3458464	0.888	0.951	1.47356E-58	3
CCT5.1	5.77172E-63	-0.3464672	0.921	0.945	1.95407E-58	3
CEMIP.1	1.07422E-62	0.36250635	0.402	0.199	3.63687E-58	3
ABCC3.2	1.29085E-62	0.348535	0.89	0.775	4.37032E-58	3
PCLAF.2	1.96985E-62	-0.4464929	0.098	0.368	6.66914E-58	3
CEBPD	2.42788E-61	0.41243068	0.805	0.682	8.21984E-57	3
RAN.1	4.20124E-61	-0.3094459	0.946	0.966	1.42237E-56	3
SERPINE2.3	5.30749E-61	-0.4917142	0.229	0.504	1.7969E-56	3
PGAM1.1	1.19595E-60	-0.3844188	0.505	0.752	4.04901E-56	3
CDKN3.2	1.48224E-59	-0.4526667	0.119	0.379	5.01826E-55	3
CALR.1	5.58326E-59	-0.3148764	0.896	0.952	1.89027E-54	3
PHF19.1	3.66472E-58	-0.420897	0.112	0.369	1.24073E-53	3
FABP5.1	7.30045E-58	-0.3975468	0.225	0.498	2.47164E-53	3
RBM3.1	7.48803E-58	0.28058009	0.957	0.897	2.53515E-53	3
SRGN	1.54119E-57	-0.353766	0.895	0.933	5.21784E-53	3
ZFAND5.2	3.79669E-57	0.38981533	0.788	0.68	1.28541E-52	3
VMP1	4.3301E-57	0.33948439	0.874	0.786	1.466E-52	3
JUNB.2	3.06241E-56	0.39206137	0.849	0.743	1.03681E-51	3
TOP2A.1	3.16626E-55	-0.6599887	0.115	0.365	1.07197E-50	3
HSP90B1.1	3.82515E-55	-0.3347111	0.838	0.918	1.29504E-50	3
EPB41L4A-AS1	1.55804E-54	0.34378812	0.75	0.611	5.27491E-50	3
TFRC	1.73854E-54	-0.3068393	0.077	0.318	5.88599E-50	3
CPS1.1	2.1443E-54	-0.4162626	0.487	0.722	7.25976E-50	3
UQCRH	3.80814E-54	-0.2551857	0.958	0.969	1.28928E-49	3
TACC2.2	4.35755E-54	0.36746472	0.627	0.472	1.47529E-49	3
DBI.1	1.82559E-53	-0.3715881	0.44	0.683	6.18072E-49	3
RHOB	2.69042E-53	0.40182135	0.791	0.652	9.1087E-49	3
C1QBP.1	3.0441E-53	-0.3472509	0.731	0.864	1.03061E-48	3
RNF145	3.15788E-53	0.31229694	0.868	0.78	1.06913E-48	3
TMC4.1	3.24213E-53	0.33769775	0.348	0.17	1.09766E-48	3
NUDT1.1	3.34501E-53	-0.333916	0.113	0.36	1.13249E-48	3
APOC1.2	4.80394E-53	-0.3819732	0.224	0.496	1.62642E-48	3
PSMA3	8.58131E-53	-0.3306688	0.599	0.788	2.90529E-48	3
RSPO3.1	1.83096E-52	-0.3826664	0.257	0.523	6.19891E-48	3
SDC1.1	2.71455E-52	-0.3905029	0.407	0.665	9.19039E-48	3
ANP32B	4.17133E-52	-0.3810491	0.625	0.797	1.41225E-47	3
JUND	6.9078E-52	0.27312614	0.94	0.893	2.3387E-47	3
UPP1.1	7.03388E-52	-0.2899726	0.048	0.265	2.38139E-47	3
CENPN	4.47112E-51	-0.3202281	0.074	0.301	1.51374E-46	3
FXYD5.2	6.40903E-51	-0.4204246	0.365	0.618	2.16984E-46	3
SSR4.1	8.65424E-51	0.26084493	0.984	0.956	2.92998E-46	3
PRKDC.2	1.77964E-50	-0.3510744	0.732	0.851	6.02516E-46	3
PA2G4.1	2.0224E-50	-0.3457061	0.688	0.841	6.84702E-46	3
CENPW.1	2.27955E-50	-0.3114146	0.062	0.283	7.71764E-46	3
HSPD1.1	2.49126E-50	-0.3867309	0.676	0.823	8.4344E-46	3
PLD3.1	3.37868E-50	0.26063407	0.916	0.856	1.14389E-45	3
ASPH.1	3.4734E-50	-0.3746082	0.54	0.752	1.17595E-45	3
BIRC5.1	3.52443E-50	-0.4281334	0.085	0.31	1.19323E-45	3
ATP5MF.1	3.76286E-50	-0.2955838	0.865	0.934	1.27395E-45	3
BOLA3.1	6.99077E-50	-0.3508568	0.528	0.738	2.36679E-45	3
ACTN1.1	1.33394E-49	-0.3556525	0.381	0.64	4.51618E-45	3
FOSL2.1	7.12799E-49	0.33824328	0.751	0.645	2.41325E-44	3
ANXA3	1.23384E-48	-0.3312499	0.176	0.421	4.1773E-44	3
SUSD2.3	1.38576E-48	0.33463346	0.764	0.614	4.69163E-44	3
ZNF503.2	1.44572E-48	0.38091091	0.634	0.474	4.89463E-44	3
CIB1.1	1.53626E-48	-0.3216627	0.756	0.886	5.20117E-44	3
BCYRN1.3	1.62404E-48	-0.5106878	0.144	0.383	5.49836E-44	3
CENPF.2	3.68358E-48	-0.5769006	0.141	0.372	1.24711E-43	3
HMGB1.2	7.43863E-48	-0.3748872	0.711	0.832	2.51842E-43	3
TYMS.1	1.11141E-47	-0.3621344	0.07	0.287	3.7628E-43	3
SNRPD1.1	1.13274E-47	-0.3657825	0.61	0.782	3.83501E-43	3
POMP.1	2.44159E-47	-0.2878871	0.793	0.888	8.26625E-43	3
LDHB.1	2.11367E-46	-0.3020109	0.74	0.861	7.15604E-42	3
SNTB2.1	1.20028E-45	0.29275757	0.825	0.712	4.06367E-41	3
RANBP1.1	2.18826E-45	-0.3882843	0.623	0.789	7.40858E-41	3
TOM1L2.1	2.87341E-45	0.32809662	0.544	0.38	9.72823E-41	3
LGALS3BP.2	3.12911E-45	0.28378188	0.894	0.703	1.05939E-40	3
YBX1.1	4.56176E-45	-0.2753575	0.918	0.944	1.54443E-40	3
TSC22D3.2	5.41761E-45	0.34532829	0.74	0.593	1.83419E-40	3
METTL9	1.70871E-44	-0.3100466	0.375	0.636	5.78502E-40	3
NSA2	2.56093E-44	0.27880442	0.924	0.887	8.6703E-40	3
JUN.2	7.76393E-44	0.45204913	0.655	0.496	2.62856E-39	3
NCL.3	1.42866E-43	-0.376717	0.867	0.915	4.83688E-39	3
SERINC5.1	2.78246E-43	0.33042175	0.468	0.308	9.42028E-39	3
ATP5MC3	4.10105E-43	-0.2816989	0.834	0.918	1.38845E-38	3
MKI67.1	4.16568E-43	-0.4832961	0.071	0.269	1.41033E-38	3
CDKN1C.2	4.30466E-43	0.41162936	0.443	0.279	1.45738E-38	3
DYNLL1.1	4.51563E-43	-0.2967441	0.845	0.922	1.52881E-38	3
GM2A.2	8.9836E-43	-0.3670933	0.317	0.55	3.04149E-38	3
HMGN2.2	2.31942E-42	-0.4251709	0.559	0.745	7.85264E-38	3
ANXA1	8.50191E-42	-0.3061962	0.79	0.862	2.87841E-37	3
MYDGF.1	1.25383E-41	-0.3106315	0.496	0.719	4.24496E-37	3
YPEL5.2	3.86706E-41	0.3003167	0.79	0.659	1.30923E-36	3
UQCR10.1	4.13275E-41	-0.2631062	0.896	0.947	1.39918E-36	3
GAS5.2	5.27587E-41	0.25349021	0.919	0.811	1.7862E-36	3
CRIP2	5.79421E-41	0.30541154	0.687	0.551	1.96169E-36	3
MT-ND6	6.69704E-41	-0.3412652	0.562	0.746	2.26735E-36	3
PDIA4.2	7.43689E-41	-0.3138016	0.534	0.74	2.51783E-36	3
HEXB	1.10487E-40	-0.2577605	0.202	0.444	3.74065E-36	3
CCNB1.1	2.38992E-40	-0.4559281	0.111	0.312	8.0913E-36	3
HNRNPM.1	3.02691E-40	-0.3062896	0.698	0.825	1.02479E-35	3
PTMA.3	3.3202E-40	-0.275514	0.998	0.989	1.12409E-35	3
DLGAP5.2	4.74962E-40	-0.4146175	0.051	0.23	1.60803E-35	3
NOP56	5.26601E-40	-0.2937104	0.357	0.599	1.78286E-35	3
RPL39.2	6.29943E-40	-0.4595276	0.998	0.997	2.13273E-35	3
FAM136A	6.86336E-40	-0.3041713	0.448	0.678	2.32366E-35	3
IER2	9.12042E-40	0.32057562	0.837	0.786	3.08781E-35	3
PLIN3	9.8376E-40	-0.2701617	0.287	0.537	3.33062E-35	3
ACOX2	1.01732E-39	-0.2784034	0.249	0.489	3.44424E-35	3
SIVA1.3	1.09332E-39	-0.3376485	0.542	0.75	3.70154E-35	3
RUVBL1	2.37858E-39	-0.3100356	0.345	0.579	8.05292E-35	3
PBK.1	3.75466E-39	-0.3130266	0.054	0.232	1.27118E-34	3
BMP7.1	4.10249E-39	0.28605055	0.787	0.649	1.38894E-34	3
CALM1.1	6.65778E-39	-0.3379109	0.98	0.976	2.25406E-34	3
COX17.1	7.77013E-39	-0.3099667	0.559	0.736	2.63065E-34	3
SDF2L1.1	1.33597E-38	-0.3126522	0.374	0.611	4.52307E-34	3
TGM2.3	1.51679E-38	-0.7166618	0.531	0.653	5.13526E-34	3
DKK1.1	1.66429E-38	-0.2651114	0.083	0.275	5.63462E-34	3
MRPL51	2.11081E-38	-0.2706469	0.544	0.754	7.14635E-34	3
RRM2.1	7.03824E-38	-0.4433909	0.08	0.261	2.38286E-33	3
NOP53	7.1911E-38	0.25037164	0.825	0.754	2.43462E-33	3
DNMT1.1	1.20708E-37	-0.3290669	0.24	0.456	4.08669E-33	3
MYBL2.1	1.37202E-37	-0.2777148	0.057	0.232	4.64511E-33	3
CKLF	2.64836E-37	-0.307991	0.631	0.795	8.96629E-33	3
CCT6A	6.20016E-37	-0.2896823	0.427	0.644	2.09913E-32	3
PGK1	6.42356E-37	-0.2949979	0.403	0.638	2.17476E-32	3
JPT1.1	7.57269E-37	-0.3259319	0.541	0.728	2.56381E-32	3
RNF19A.2	1.6429E-36	0.325583	0.693	0.588	5.5622E-32	3
CCND3.1	3.06429E-36	0.31900514	0.474	0.331	1.03745E-31	3
CD320.1	3.77128E-36	-0.3073711	0.665	0.828	1.27681E-31	3
FERMT1.2	4.19877E-36	-0.4163411	0.28	0.493	1.42154E-31	3
FADS1	4.27674E-36	-0.3030729	0.221	0.445	1.44793E-31	3
TPBG	5.35786E-36	0.30793527	0.693	0.61	1.81396E-31	3
SH3BP5.2	6.69873E-36	0.28351013	0.39	0.241	2.26792E-31	3
NASP.2	7.03854E-36	-0.3089024	0.327	0.549	2.38297E-31	3
DYNC1H1	8.36553E-36	-0.2655862	0.87	0.918	2.83223E-31	3
GRN	1.03344E-35	0.25847456	0.791	0.691	3.49883E-31	3
HSPA9.1	1.8434E-35	0.32638311	0.913	0.86	6.24101E-31	3
ATAD2.1	3.75951E-35	-0.3134864	0.115	0.304	1.27282E-30	3
DTYMK.1	4.63553E-35	-0.2844795	0.169	0.368	1.56941E-30	3
CDC20.1	4.99744E-35	-0.3261724	0.073	0.248	1.69193E-30	3
SNRPG.1	5.40819E-35	-0.2754603	0.733	0.853	1.831E-30	3
F2R.1	5.8037E-35	0.29186217	0.414	0.272	1.9649E-30	3
CITED4.1	9.00218E-35	-0.2525868	0.048	0.21	3.04778E-30	3
BBC3	1.26457E-34	0.26339261	0.351	0.208	4.28132E-30	3
EIF5A.1	1.76741E-34	-0.2616151	0.9	0.934	5.98373E-30	3
KLF2	2.8509E-34	0.36471463	0.578	0.448	9.65202E-30	3
TNRC6B	3.91792E-34	0.27598754	0.778	0.699	1.32645E-29	3
NDUFB3	4.94253E-34	-0.2619787	0.552	0.738	1.67334E-29	3
CALM3.1	8.81614E-34	-0.2979128	0.493	0.694	2.98479E-29	3
CYTOR.1	9.73788E-34	-0.3002776	0.446	0.661	3.29686E-29	3
EIF5	9.91788E-34	-0.2779011	0.487	0.7	3.3578E-29	3
AREG.1	1.03964E-33	-0.6013321	0.508	0.664	3.5198E-29	3
MAD2L1.1	1.06567E-33	-0.2605395	0.081	0.252	3.60793E-29	3
HNRNPC.1	2.68404E-33	-0.2652007	0.799	0.886	9.08708E-29	3
TUBA1B.2	2.84316E-33	-0.6277928	0.66	0.762	9.62579E-29	3
SAMD11.1	3.11804E-33	0.28063465	0.481	0.34	1.05564E-28	3
SRSF7	3.60227E-33	-0.2686693	0.42	0.631	1.21959E-28	3
RPL26.2	4.59642E-33	-0.2874692	0.991	0.994	1.55616E-28	3
IL18.3	4.99424E-33	0.26786348	0.801	0.698	1.69085E-28	3
CCNB2	5.52429E-33	-0.2566847	0.044	0.198	1.8703E-28	3
TPX2.1	7.59121E-33	-0.3530937	0.109	0.283	2.57008E-28	3
RHOBTB3.3	8.96857E-33	0.27906337	0.772	0.667	3.0364E-28	3
CYC1.1	1.16671E-32	-0.2593322	0.739	0.86	3.95002E-28	3
MDH2.1	1.78903E-32	-0.2703108	0.662	0.809	6.05693E-28	3
LENG8	1.94725E-32	0.28312018	0.653	0.548	6.59262E-28	3
CAST.2	3.04573E-32	0.25147235	0.923	0.893	1.03116E-27	3
PSMC1	3.32996E-32	-0.2529121	0.597	0.763	1.12739E-27	3
MTCO1P40	4.52415E-32	-0.2666908	0.581	0.754	1.5317E-27	3
PSMA4	6.35245E-32	-0.2632845	0.517	0.704	2.15069E-27	3
DUT.1	7.3096E-32	-0.3226099	0.463	0.661	2.47474E-27	3
IGFBP3.1	9.31894E-32	-0.4270985	0.038	0.184	3.15502E-27	3
UBXN1	1.0756E-31	0.26411686	0.771	0.712	3.64156E-27	3
PTGR1	1.08326E-31	-0.2653927	0.34	0.558	3.66749E-27	3
SYNM.1	1.30893E-31	0.27663159	0.599	0.479	4.43153E-27	3
SNRPF.1	1.83928E-31	-0.2868886	0.633	0.785	6.22706E-27	3
ANP32E.1	3.4371E-31	-0.2982944	0.361	0.562	1.16366E-26	3
NMB	1.26114E-30	-0.2573914	0.388	0.605	4.26973E-26	3
UBE2C.1	1.87068E-30	-0.4702955	0.085	0.244	6.33336E-26	3
UBE2H	4.31138E-30	0.25231244	0.661	0.579	1.45966E-25	3
ZKSCAN1.1	4.43462E-30	0.27309146	0.7	0.618	1.50139E-25	3
SUPT16H.1	1.08557E-29	-0.2637212	0.535	0.71	3.67529E-25	3
AKR1B1.1	1.24202E-29	-0.2678008	0.613	0.766	4.20499E-25	3
MIR23AHG	1.92853E-29	0.3279028	0.655	0.554	6.52923E-25	3
AMOTL2.2	2.67324E-29	0.29035916	0.62	0.522	9.05051E-25	3
SNHG7	7.18506E-29	0.2746244	0.614	0.526	2.43257E-24	3
PHB.1	1.20279E-28	-0.271765	0.565	0.734	4.07217E-24	3
SSBP3	2.02717E-28	0.28391106	0.487	0.372	6.8632E-24	3
SNRPB.1	2.03363E-28	-0.2727889	0.483	0.668	6.88506E-24	3
IGFBP4.1	2.56972E-28	-0.310088	0.361	0.55	8.70004E-24	3
AHSA1.2	1.14049E-27	-0.2660527	0.459	0.651	3.86123E-23	3
PMEPA1.1	1.23356E-27	-0.2789432	0.615	0.771	4.17633E-23	3
HMGB2.2	2.2271E-27	-0.3175907	0.104	0.258	7.54008E-23	3
CALM2.1	4.95885E-27	-0.2550021	0.908	0.93	1.67887E-22	3
PRTG	1.43845E-26	0.26225981	0.585	0.489	4.87001E-22	3
PER1	3.82593E-26	0.26349131	0.527	0.419	1.29531E-21	3
PDK4.1	5.76952E-26	0.29653212	0.417	0.292	1.95333E-21	3
UBE2S.1	7.2894E-26	-0.4315491	0.593	0.725	2.4679E-21	3
NREP	1.22914E-25	0.26036936	0.556	0.452	4.16137E-21	3
RPS24.2	1.38362E-25	-0.3350618	0.986	0.982	4.68439E-21	3
S100P.2	1.57979E-25	-0.2924214	0.041	0.167	5.34853E-21	3
AURKA	1.67328E-25	-0.2616901	0.095	0.243	5.66507E-21	3
FDPS.1	6.55782E-25	-0.2720019	0.688	0.805	2.22022E-20	3
ZFP36L1	2.72748E-24	0.28916292	0.733	0.666	9.23417E-20	3
S100A8.2	2.82739E-24	-0.6566839	0.036	0.152	9.57242E-20	3
TM4SF1	5.33716E-24	-0.2778876	0.371	0.556	1.80695E-19	3
H4C3.2	5.78811E-23	-0.6511753	0.584	0.712	1.95962E-18	3
SOCS6	6.02665E-23	0.25722931	0.504	0.414	2.04038E-18	3
CD24.2	7.49362E-23	0.29109331	0.517	0.409	2.53704E-18	3
AKAP12	7.79624E-23	-0.3273536	0.296	0.468	2.63949E-18	3
TUBB4B.1	1.01394E-22	-0.3199215	0.584	0.722	3.43278E-18	3
RPL34.2	1.26599E-22	-0.2742498	0.992	0.987	4.28613E-18	3
ERRFI1.1	1.40025E-22	0.26000428	0.48	0.384	4.74069E-18	3
HEY1.1	1.56656E-22	0.26385188	0.35	0.242	5.30375E-18	3
CDKN1A.2	1.40137E-21	0.27806995	0.526	0.429	4.74447E-17	3
BMPR2	2.5776E-21	0.25967346	0.495	0.414	8.7266E-17	3
LUCAT1.2	8.4178E-21	-0.2866808	0.375	0.545	2.8499E-16	3
VGF.3	5.1508E-20	-0.3354061	0.022	0.114	1.7439E-15	3
RPL7.2	1.9374E-19	-0.2826046	0.861	0.886	6.5593E-15	3
ZFP36.1	7.219E-19	0.26237544	0.487	0.395	2.4441E-14	3
KLF6.1	1.0916E-17	0.26778318	0.599	0.52	3.6957E-13	3
ADGRG2.1	1.1755E-17	0.30322122	0.355	0.269	3.9798E-13	3
CEACAM6.2	2.9301E-16	-0.2670431	0.244	0.371	9.9201E-12	3
MT2A.2	4.2906E-13	-0.2992945	0.993	0.985	1.4526E-08	3
PCNX4.1	2.1111E-10	0.2588371	0.572	0.56	7.1472E-06	3
NAMPT.1	1.637E-09	0.26412802	0.465	0.424	5.5421E-05	3
PTTG1.3	1.216E-289	1.08238294	0.844	0.311	4.118E-285	4
H2AZ1.3	2.763E-240	0.89751863	0.992	0.744	9.354E-236	4
HMGN2.3	5.073E-176	0.68358762	0.954	0.69	1.717E-171	4
BIRC5.2	3.07E-174	0.5983157	0.68	0.229	1.039E-169	4
EEF1A1.2	4.313E-160	-0.3966313	1	1	1.46E-155	4
SNHG3	7.748E-153	0.63817321	0.961	0.822	2.623E-148	4
TGM2.4	7.722E-146	0.83772659	0.88	0.606	2.615E-141	4
KRT18.2	1.435E-145	0.54663859	0.994	0.961	4.86E-141	4
KRT8.2	6.648E-141	0.55438133	0.987	0.935	2.251E-136	4
IGFL2-AS1	8.668E-135	0.4721506	0.357	0.083	2.935E-130	4
MALAT1.4	2.919E-132	-0.8120616	0.996	0.999	9.883E-128	4
ITPR1.1	2.882E-124	0.58143973	0.736	0.387	9.757E-120	4
STMN1.4	1.523E-122	0.53610736	0.969	0.785	5.158E-118	4
PCLAF.3	5.152E-120	0.40699378	0.697	0.285	1.744E-115	4
CENPW.2	1.906E-119	0.44768541	0.564	0.214	6.454E-115	4
CCT5.2	2.038E-115	0.39202652	0.99	0.936	6.901E-111	4
NUDT1.2	2.211E-113	0.3974772	0.659	0.284	7.487E-109	4
COX17.2	2.456E-109	0.46565062	0.927	0.685	8.317E-105	4
CALM3.2	6.586E-109	0.47213362	0.907	0.637	2.23E-104	4
CDKN3.3	3.054E-103	0.49625162	0.65	0.305	1.0339E-98	4
B2M.2	6.336E-103	0.42316993	0.994	0.948	2.145E-98	4
RPL10.1	4.5789E-98	-0.2739333	1	0.998	1.5502E-93	4
YBX1.2	9.7986E-95	0.33326376	0.99	0.934	3.3174E-90	4
RAN.2	3.3441E-93	0.33467306	0.998	0.959	1.1322E-88	4
RPA3	1.3268E-91	0.42515156	0.744	0.421	4.4919E-87	4
ENO1.1	2.6725E-91	0.2915189	0.999	0.983	9.048E-87	4
TYMS.2	1.8225E-90	0.37766246	0.547	0.221	6.1702E-86	4
TYMSOS	9.2772E-88	0.29783712	0.4	0.14	3.1409E-83	4
ANAPC11	2.995E-87	0.33583165	0.988	0.941	1.014E-82	4
PSMA7.2	1.6365E-86	0.2968008	0.997	0.98	5.5406E-82	4
HNRNPM.2	3.7641E-86	0.39181243	0.942	0.791	1.2744E-81	4
COX8A	1.5642E-84	0.35473356	0.964	0.865	5.2958E-80	4
LMNA.1	1.9139E-84	0.29930545	0.996	0.973	6.4796E-80	4
DCBLD2.1	3.0961E-82	0.41478858	0.911	0.655	1.0482E-77	4
CENPN.1	1.2911E-81	0.35427255	0.533	0.237	4.371E-77	4
PSMD2.1	1.0174E-80	0.36211176	0.939	0.811	3.4444E-76	4
MBD2	1.8815E-80	0.35786975	0.655	0.357	6.3699E-76	4
HINT1	3.3127E-80	0.25049919	1	0.993	1.1216E-75	4
MAL2.2	5.6082E-80	0.34123098	0.645	0.323	1.8987E-75	4
HNRNPA2B1.1	7.5951E-78	0.32396079	0.986	0.933	2.5714E-73	4
HMGB1.3	1.94E-76	0.36882776	0.947	0.799	6.5679E-72	4
DLGAP5.3	8.6105E-75	0.45561264	0.437	0.177	2.9152E-70	4
CTC-246B18.1	1.325E-72	0.30774262	0.483	0.216	4.4861E-68	4
UBE2S.2	1.8665E-72	0.52055532	0.872	0.686	6.3192E-68	4
PHF19.2	3.3151E-72	0.35282026	0.602	0.3	1.1224E-67	4
RPS27.1	9.5541E-72	-0.2803664	1	0.999	3.2346E-67	4
PRDX1.1	1.1562E-71	0.28719296	0.996	0.986	3.9145E-67	4
EEF2.2	6.7783E-70	-0.3381161	0.978	0.986	2.2949E-65	4
RANBP1.2	1.0892E-68	0.35950518	0.921	0.747	3.6875E-64	4
LINP1	2.0002E-68	0.25883319	0.372	0.145	6.772E-64	4
PRDX2	1.5479E-67	0.26124048	0.996	0.971	5.2406E-63	4
SSRP1	1.8748E-67	0.34365285	0.782	0.517	6.3472E-63	4
BOLA3.2	2.8491E-67	0.35104969	0.872	0.689	9.6459E-63	4
STK32C	4.0097E-67	0.350212	0.758	0.493	1.3575E-62	4
LDHA.2	4.1737E-65	0.29847847	0.982	0.937	1.413E-60	4
S100A16.2	5.1522E-65	0.36000714	0.898	0.632	1.7443E-60	4
HSP90AB1.2	2.0929E-64	0.25270722	0.99	0.967	7.0856E-60	4
TAGLN2.2	1.5929E-63	0.31947558	0.97	0.868	5.393E-59	4
PBK.2	3.3892E-63	0.37518612	0.418	0.182	1.1475E-58	4
PGAM1.2	3.8446E-63	0.33163517	0.879	0.699	1.3016E-58	4
HSPA8	1.3679E-62	-0.3255828	0.902	0.951	4.6311E-58	4
RUVBL2	5.5578E-62	0.34027105	0.718	0.45	1.8816E-57	4
FABP5.2	1.3928E-61	0.34113169	0.708	0.431	4.7154E-57	4
PLD3.2	1.4267E-61	-0.4426975	0.79	0.874	4.8303E-57	4
GGH	1.5702E-61	0.27719338	0.517	0.259	5.3159E-57	4
SOX4.1	7.6966E-61	-0.3681132	0.97	0.983	2.6058E-56	4
CCNB2.1	7.0104E-60	0.30391558	0.371	0.152	2.3734E-55	4
CALR.2	9.6855E-60	0.27660139	0.979	0.941	3.2791E-55	4
TXN.1	4.4825E-58	0.25778197	0.992	0.968	1.5176E-53	4
LSM4.1	2.716E-57	0.32752435	0.907	0.721	9.1953E-53	4
TFDP1	5.9007E-57	0.30673978	0.678	0.41	1.9977E-52	4
HNRNPC.2	6.651E-57	0.26657169	0.947	0.865	2.2518E-52	4
PRR11	9.2323E-57	0.26461147	0.372	0.156	3.1257E-52	4
MZT2B.1	1.2371E-56	0.25402932	0.994	0.944	4.1884E-52	4
RPL10A.2	1.6628E-56	-0.2865464	0.989	0.988	5.6294E-52	4
UQCR10.2	4.7444E-56	0.27344295	0.982	0.935	1.6063E-51	4
MAD2L1.2	7.0991E-56	0.31317155	0.434	0.203	2.4035E-51	4
XRCC6	7.1726E-56	0.3058847	0.875	0.674	2.4284E-51	4
S100P.3	1.1972E-55	0.39870921	0.324	0.128	4.0533E-51	4
CDC20.2	3.5663E-54	0.4295271	0.418	0.2	1.2074E-49	4
CPA4.1	3.5784E-54	0.27147151	0.388	0.172	1.2115E-49	4
ATP5F1B	3.9143E-54	0.27407739	0.962	0.896	1.3252E-49	4
HMMR	4.2609E-54	0.37169952	0.334	0.14	1.4426E-49	4
CHML	4.8472E-54	0.28133583	0.4	0.183	1.6411E-49	4
MACROH2A1	1.364E-53	0.30276079	0.918	0.756	4.6181E-49	4
SLC7A5	6.7614E-53	0.27306612	0.458	0.228	2.2891E-48	4
CCNB1.2	7.3176E-53	0.46544651	0.487	0.26	2.4774E-48	4
IGFBP4.2	2.8901E-52	0.3006031	0.758	0.495	9.7846E-48	4
HMGN1.1	8.1119E-52	0.27591873	0.946	0.848	2.7464E-47	4
CEACAM6.3	1.7579E-51	0.29838424	0.588	0.324	5.9516E-47	4
CST3.3	6.3406E-51	-0.3727983	0.976	0.97	2.1467E-46	4
PDCD5	8.708E-51	0.29102795	0.913	0.763	2.9482E-46	4
OAZ1	1.7853E-50	0.2585221	0.959	0.871	6.0444E-46	4
TUBA1B.3	1.9806E-50	0.48748252	0.863	0.733	6.7057E-46	4
COMMD4.1	5.0808E-50	0.30625563	0.83	0.621	1.7202E-45	4
SNRPD1.2	7.0521E-50	0.28643245	0.919	0.739	2.3876E-45	4
ANP32B.1	7.2321E-50	0.3273755	0.897	0.758	2.4485E-45	4
EDIL3.2	7.8502E-50	-0.4523419	0.3	0.534	2.6578E-45	4
AP2S1.1	8.3452E-50	0.25085038	0.976	0.912	2.8254E-45	4
SEZ6L2	1.0449E-48	-0.3824744	0.677	0.785	3.5376E-44	4
LY6E.1	1.1134E-48	0.28869447	0.956	0.848	3.7696E-44	4
MICOS10.1	1.3279E-48	0.29020101	0.903	0.743	4.4957E-44	4
CLPTM1L	1.3863E-48	0.2797411	0.898	0.744	4.6935E-44	4
TFRC.1	1.7927E-48	0.25072353	0.491	0.26	6.0693E-44	4
CFD.1	1.9301E-48	-0.5459723	0.592	0.724	6.5346E-44	4
SNRPG.2	3.8255E-48	0.27986291	0.947	0.823	1.2952E-43	4
CLTB	5.2298E-48	0.31184954	0.842	0.681	1.7706E-43	4
FDPS.2	1.2304E-47	0.33872501	0.9	0.775	4.1657E-43	4
ALYREF.1	1.4957E-47	0.29677797	0.804	0.58	5.064E-43	4
CENPF.3	4.7794E-47	0.44563193	0.544	0.316	1.6181E-42	4
PSMB3.1	6.1914E-47	0.28305724	0.935	0.814	2.0962E-42	4
SPTBN1.1	2.1203E-46	0.33638792	0.857	0.692	7.1785E-42	4
MRPL36	3.15E-46	0.27625925	0.873	0.69	1.0665E-41	4
EFHD2	4.6093E-46	0.25242283	0.626	0.38	1.5605E-41	4
ANXA3.1	1.1746E-45	0.28907851	0.594	0.362	3.9767E-41	4
CPS1.2	1.9673E-45	0.25843293	0.868	0.668	6.6605E-41	4
S100A13.2	4.1924E-45	0.29985219	0.774	0.533	1.4194E-40	4
FGFBP1.2	4.2999E-45	0.28037288	0.505	0.278	1.4558E-40	4
CTNNAL1	1.1658E-44	0.29706381	0.736	0.539	3.9469E-40	4
MYDGF.2	3.0685E-44	0.25613091	0.861	0.668	1.0389E-39	4
LSM5	3.887E-44	0.27976267	0.887	0.693	1.316E-39	4
MDH1	5.0955E-44	0.28188175	0.853	0.693	1.7251E-39	4
MKI67.2	5.2645E-44	0.2560923	0.446	0.217	1.7823E-39	4
PABPC1.1	2.7116E-43	-0.2605079	0.97	0.97	9.1805E-39	4
ITGAE	5.082E-43	0.27307898	0.715	0.5	1.7206E-38	4
MYL12B	1.3635E-42	0.25249086	0.946	0.849	4.6161E-38	4
ANP32E.2	1.5009E-42	0.34309895	0.713	0.513	5.0816E-38	4
COTL1.2	2.4998E-42	0.29252236	0.651	0.419	8.4635E-38	4
SERPINE2.4	2.9299E-42	0.26744392	0.677	0.44	9.9194E-38	4
DPM3.1	3.2446E-42	0.25746844	0.948	0.879	1.0985E-37	4
NOP56.1	4.0634E-41	0.2667346	0.751	0.543	1.3757E-36	4
FADS1.1	5.503E-41	0.30619428	0.599	0.392	1.8631E-36	4
PA2G4.2	2.3966E-40	0.26571354	0.935	0.806	8.1138E-36	4
H2AZ2.1	5.1277E-40	0.26651242	0.838	0.65	1.736E-35	4
EIF4A2	2.2281E-39	-0.3385804	0.532	0.684	7.5434E-35	4
HMGN3	4.3194E-39	0.25519824	0.839	0.674	1.4624E-34	4
HNRNPH1	6.5317E-39	0.27193385	0.807	0.604	2.2114E-34	4
CKAP4	8.1791E-39	0.2659117	0.792	0.617	2.7691E-34	4
PSAP	1.3404E-38	-0.3223942	0.847	0.876	4.5382E-34	4
DEF8	1.0963E-37	0.25839079	0.748	0.539	3.7115E-33	4
STC1.2	4.0416E-37	0.32463588	0.671	0.458	1.3683E-32	4
SDC1.2	6.0304E-37	0.25938887	0.809	0.608	2.0417E-32	4
NUDC.1	7.1961E-37	0.25100915	0.834	0.632	2.4363E-32	4
GABPB1-AS1	1.4204E-36	0.25929294	0.836	0.69	4.8088E-32	4
PSMA4.1	8.9469E-36	0.25393335	0.821	0.661	3.0291E-31	4
DYNC2I2	1.0183E-35	0.25659021	0.676	0.468	3.4474E-31	4
FXYD5.3	1.7591E-35	0.25380568	0.761	0.562	5.9555E-31	4
HNRNPAB.1	2.7381E-35	0.27478566	0.898	0.758	9.27E-31	4
ITGA10	6.7075E-34	-0.3286173	0.065	0.236	2.2709E-29	4
MALSU1	8.0259E-34	0.26929237	0.7	0.514	2.7172E-29	4
MSLN.2	6.6744E-33	-0.4339389	0.608	0.695	2.2597E-28	4
GPATCH4	1.0203E-32	0.25008383	0.761	0.589	3.4543E-28	4
LRATD2.1	3.285E-31	0.25100782	0.531	0.337	1.1122E-26	4
ISG20.2	6.4443E-31	-0.3750911	0.664	0.749	2.1818E-26	4
DUSP6	7.026E-31	-0.3525325	0.18	0.35	2.3787E-26	4
KLF2.1	1.2074E-30	-0.4092583	0.314	0.485	4.0879E-26	4
IGFBP5.3	3.0684E-30	-0.5080967	0.321	0.481	1.0388E-25	4
TIMP1.3	3.4807E-30	-0.4951914	0.986	0.988	1.1784E-25	4
RHOB.1	4.7072E-30	-0.3893629	0.554	0.685	1.5937E-25	4
AHNAK2	1.7126E-29	-0.3279029	0.615	0.711	5.7981E-25	4
GAS5.3	3.5705E-29	-0.3946944	0.791	0.83	1.2088E-24	4
ABCC3.3	2.5701E-28	-0.3253575	0.751	0.796	8.7013E-24	4
ATP2B1.1	2.7747E-28	-0.3139472	0.484	0.615	9.3939E-24	4
IRF2BPL	4.0026E-28	-0.3055785	0.718	0.781	1.3551E-23	4
NPTX1	5.1896E-28	-0.3175991	0.185	0.349	1.757E-23	4
CYP24A1.3	1.6927E-27	-0.2787306	0.927	0.903	5.7306E-23	4
TUBA1A.2	2.9785E-27	-0.3273223	0.253	0.413	1.0084E-22	4
CHMP1B.2	3.9814E-27	-0.2633482	0.928	0.936	1.3479E-22	4
ERRFI1.2	6.368E-25	-0.3061774	0.266	0.413	2.156E-20	4
JUNB.3	3.2198E-23	-0.3606175	0.726	0.761	1.0901E-18	4
RASSF8.1	8.4387E-23	-0.3138848	0.453	0.555	2.857E-18	4
LENG8.1	1.5696E-22	-0.2898886	0.467	0.574	5.314E-18	4
PCNX4.2	4.9214E-22	-0.3290228	0.462	0.574	1.6662E-17	4
MARCKSL1.2	2.3283E-21	-0.2814741	0.335	0.473	7.8826E-17	4
SELENOP.3	7.1753E-21	-0.3355654	0.443	0.533	2.4293E-16	4
THSD4	9.6929E-21	-0.2659871	0.512	0.613	3.2816E-16	4
NOVA1.1	9.9228E-21	-0.2844599	0.349	0.478	3.3595E-16	4
CRIP2.1	1.8214E-20	-0.3183898	0.485	0.58	6.1664E-16	4
EPB41L4A-AS1	2.7242E-20	-0.2856022	0.559	0.638	9.2229E-16	4
MIDN	3.9786E-20	-0.2608193	0.688	0.751	1.347E-15	4
SPP1.3	5.1695E-19	-0.3537063	0.986	0.973	1.7502E-14	4
SEMA3C	5.8364E-19	-0.2621408	0.67	0.721	1.976E-14	4
GRN.1	3.6338E-17	-0.2532294	0.665	0.709	1.2303E-12	4
S100A9.4	1.4163E-16	-0.6732682	0.756	0.581	4.7952E-12	4
IGFBP2.1	1.8757E-16	-0.2707287	0.296	0.412	6.3504E-12	4
MAP2.2	3.3076E-16	-0.3086411	0.276	0.388	1.1198E-11	4
FOSB.2	6.3498E-16	-0.3269602	0.764	0.792	2.1498E-11	4
CEBPD.1	7.8831E-14	-0.2671963	0.651	0.705	2.6689E-09	4
HES1.2	1.3287E-13	-0.2979648	0.431	0.526	4.4984E-09	4
SCG2.4	3.4776E-05	-0.355512	0.654	0.637	1	4
S100A8.3	0.00023662	-0.4284313	0.182	0.131	1	4
AKAP12.1	0.0013892	-0.25946	0.443	0.446	1	4
IGFBP3.2	0.00308585	-0.2943381	0.138	0.168	1	4
UBE2C.2	0.00444746	-0.3605736	0.204	0.225	1	4
VGF.4	0.00662995	-0.2519267	0.08	0.105	1	4
IGFBP3.3	0	1.29928069	0.683	0.113	0	5
CTSL.2	3.382E-268	1.302876	0.985	0.747	1.145E-263	5
S100A11.2	3.694E-267	0.81428209	1	0.985	1.25E-262	5
TCIM	2.158E-261	0.35473968	0.238	0.009	7.306E-257	5
S100A9.5	1.199E-222	2.75925789	0.905	0.571	4.058E-218	5
HLA-A.4	1.212E-198	-0.9193581	0.888	0.969	4.105E-194	5
HSP90AA1.4	5.448E-196	0.80614242	1	0.973	1.844E-191	5
LGALS3BP.3	1.458E-185	-1.2209903	0.271	0.774	4.935E-181	5
CXCL8	6.933E-174	0.56511622	0.447	0.093	2.347E-169	5
SPP1.4	1.823E-171	0.68438168	0.997	0.972	6.173E-167	5
CALD1	3.393E-169	0.41294787	0.436	0.085	1.149E-164	5
S100A4.2	8.262E-169	-1.1920871	0.937	0.956	2.797E-164	5
AC003092.1	5.542E-165	0.56661918	0.512	0.129	1.876E-160	5
TFPI2.3	3.33E-161	0.63586551	0.999	0.978	1.128E-156	5
C15orf48.2	3.615E-160	0.80969698	0.992	0.863	1.224E-155	5
PFDN2.2	6.984E-159	0.53240152	0.996	0.96	2.364E-154	5
RSPO3.2	2.453E-152	0.66772189	0.837	0.453	8.306E-148	5
RASD1	1.508E-150	0.55571376	0.477	0.12	5.105E-146	5
SMOC1	4.848E-148	0.34864796	0.295	0.044	1.641E-143	5
S100A8.4	1.617E-147	1.95645924	0.433	0.107	5.475E-143	5
NOTUM.4	7.837E-147	-1.2466356	0.236	0.688	2.653E-142	5
TAGLN2.3	3.948E-135	0.65087652	0.985	0.869	1.337E-130	5
MUC1.3	4.256E-133	-0.9375658	0.717	0.873	1.441E-128	5
TMEM158	2.757E-130	0.47137002	0.475	0.137	9.332E-126	5
DKK1.2	5.466E-130	0.49935171	0.604	0.214	1.851E-125	5
PMEPA1.2	2.377E-128	0.60972488	0.95	0.731	8.047E-124	5
MT2A.3	8.635E-124	0.95364236	0.997	0.985	2.923E-119	5
RPN2	5.878E-123	-0.6749186	0.933	0.964	1.99E-118	5
FAM83A.1	3.136E-120	0.81322216	0.926	0.731	1.062E-115	5
ACTB.2	3.321E-120	0.44077073	1	0.997	1.124E-115	5
CHL1	9.306E-120	0.33626316	0.424	0.11	3.151E-115	5
APLP2.3	3.033E-117	-0.601784	0.871	0.943	1.027E-112	5
SNHG29.2	2.544E-116	-0.4782443	0.994	0.996	8.612E-112	5
FTH1.3	8.121E-115	0.4728896	0.999	0.998	2.75E-110	5
IGFBP4.3	7.795E-112	0.6331608	0.819	0.496	2.639E-107	5
CIB1.2	8.397E-112	0.51757576	0.979	0.858	2.843E-107	5
SLIRP.3	1.545E-109	0.46818982	0.985	0.92	5.23E-105	5

Gene	p_val	avg_logFC	pct.1	pct.2	p_val_adj	cluster
SNHG25.2	6.1927E-108	-0.7162978	0.464	0.76	2.0966E-103	5
TPI1.2	1.7839E-107	0.48460783	0.992	0.914	6.0395E-103	5
FTL	6.1988E-106	-0.4595467	0.999	0.999	2.0987E-101	5
UPP1.2	4.8945E-104	0.43123286	0.55	0.205	1.6571E-99	5
TOP1.1	7.7313E-103	0.49645286	0.968	0.841	2.61752E-98	5
ARNT2	6.3275E-102	0.36096622	0.318	0.076	2.14225E-97	5
LUCAT1.3	2.1609E-101	0.71312435	0.801	0.495	7.31589E-97	5
CITED4.2	8.0015E-101	0.42274156	0.481	0.16	2.709E-96	5
PSMA7.3	1.28875E-98	0.37770055	0.999	0.98	4.36319E-94	5
FOSB.3	6.4896E-98	-0.8136978	0.574	0.81	2.19712E-93	5
STC1.3	8.33601E-98	0.93962976	0.769	0.454	2.82224E-93	5
ACTN1.2	8.94107E-97	0.52093279	0.856	0.581	3.02709E-92	5
POMP.2	3.73071E-95	0.40463752	0.978	0.866	1.26307E-90	5
MALAT1.5	9.0413E-95	-0.737303	0.997	0.998	3.06102E-90	5
NT5E	1.02424E-94	0.42615848	0.527	0.206	3.46767E-90	5
[illegible]	[illegible]	[illegible]	[illegible]	[illegible]	[illegible]	[illegible]

Gene	p_val	avg_logFC	pct.1	pct.2	p_val_adj	cluster
CD24.3	1.20729E-30	-0.4124839	0.243	0.442	4.0874E-26	5
BZW1	2.6833E-30	0.25167525	0.802	0.633	9.0846E-26	5
PLIN3.1	3.2213E-30	0.2874764	0.67	0.487	1.0906E-25	5
NOL3	4.52819E-30	-0.3068486	0.554	0.685	1.5331E-25	5
TGM2.5	6.59378E-30	0.50344073	0.78	0.623	2.2324E-25	5
ZNF503.3	1.01123E-29	-0.40717	0.338	0.51	3.4236E-25	5
STK17A	1.01804E-29	0.25684418	0.506	0.315	3.4467E-25	5
PER1.1	1.16188E-29	-0.332674	0.251	0.451	3.9337E-25	5
RHOBTB2	1.52087E-29	-0.2755603	0.097	0.287	5.1491E-25	5
ST3GAL4.1	1.55338E-29	-0.3123938	0.215	0.407	5.2591E-25	5
FOS	1.66164E-29	-0.2748287	0.961	0.964	5.6256E-25	5
VMP1.1	2.39665E-29	-0.3219914	0.714	0.806	8.1141E-25	5
PNRC1	3.38304E-29	-0.3447654	0.466	0.621	1.1454E-24	5
PBXIP1	8.02735E-29	-0.3134816	0.173	0.366	2.7177E-24	5
RPAIN	8.37854E-29	-0.3074139	0.604	0.725	2.8366E-24	5
[illegible]	[illegible]	[illegible]	[illegible]	[illegible]	[illegible]	[illegible]

Gene	p_val	avg_logFC	pct.1	pct.2	p_val_adj	cluster
PHF19.4	6.17083E-53	0.43008928	0.632	0.314	2.0892E-48	6
PAPOLA.1	8.04146E-53	0.42213586	0.857	0.646	2.72252E-48	6
FOSB.4	1.52901E-52	-0.704687	0.607	0.801	5.17662E-48	6
SUPT16H.3	2.548E-52	0.45824144	0.865	0.674	8.62651E-48	6
DTYMK.3	2.74825E-52	0.41138214	0.62	0.323	9.30449E-48	6
ANP32A	3.27203E-52	0.44167292	0.847	0.668	1.10778E-47	6
DDX18	5.79557E-52	0.43786865	0.828	0.631	1.96215E-47	6
RPS25.1	1.25846E-51	-0.3549315	0.951	0.971	4.26063E-47	6
YPEL5.4	2.68574E-51	-0.5824697	0.434	0.693	9.09283E-47	6
UBC.1	2.97747E-51	-0.4370662	0.886	0.94	1.00805E-46	6
RASD1.1	3.13829E-51	0.32975109	0.384	0.136	1.0625E-46	6
COX7C	1.44779E-50	-0.2656983	0.99	0.993	4.90165E-46	6
GPX1.3	2.30685E-49	0.3444236	0.992	0.923	7.81007E-45	6
EIF3J.1	3.27344E-49	0.39216669	0.941	0.796	1.10826E-44	6
AREG.3	3.4867E-49	0.77172224	0.822	0.631	1.18046E-44	6
[illegible]	[illegible]	[illegible]	[illegible]	[illegible]	[illegible]	[illegible]

Gene	p_val	avg_logFC	pct.1	pct.2	p_val_adj	cluster
CENPN.2	8.50843E-27	0.26815603	0.472	0.257	2.88061E-22	6
HMGB2.3	9.31896E-27	0.34784573	0.425	0.224	3.15503E-22	6
WARS1	1.2639E-26	0.28349219	0.489	0.285	4.27907E-22	6
CTSD.2	1.2814E-26	-0.3547098	0.75	0.844	4.33831E-22	6
RHEB.1	1.4225E-26	0.29272915	0.646	0.438	4.81603E-22	6
ATP5MC1.1	1.52335E-26	0.29826518	0.896	0.796	5.15746E-22	6
USP36	1.63606E-26	0.32530125	0.779	0.635	5.53905E-22	6
BCCIP	1.75651E-26	0.26870204	0.519	0.311	5.94684E-22	6
TOMM40	2.06562E-26	0.28244394	0.693	0.489	6.99335E-22	6
EIF5.1	2.37989E-26	0.36536371	0.802	0.663	8.05736E-22	6
RBM25.1	2.46827E-26	0.2822974	0.914	0.809	8.35659E-22	6
LAPTM4B	2.95973E-26	0.25833348	0.91	0.795	1.00205E-21	6
PSMD1	5.09496E-26	0.29434524	0.738	0.561	1.72495E-21	6
POP7	7.22727E-26	0.27135068	0.569	0.355	2.44686E-21	6
AC016739.2	7.92333E-26	0.26998845	0.728	0.533	2.68252E-21	6
[illegible]	[illegible]	[illegible]	[illegible]	[illegible]	[illegible]	[illegible]

Gene	p_val	avg_logFC	pct.1	pct.2	p_val_adj	cluster
TGFBI.3	1.1879E-53	0.38322341	0.801	0.448	4.0219E-49	7
HLA-C.4	1.8144E-53	0.50041304	0.953	0.719	6.1428E-49	7
HEXB.1	2.4113E-53	0.32845001	0.756	0.389	8.1635E-49	7
ITGA1	2.1154E-51	0.25470282	0.484	0.187	7.162E-47	7
NPC2.2	4.1681E-51	0.36523	0.924	0.668	1.4112E-46	7
KRT18.4	4.3947E-51	0.39913726	0.996	0.963	1.4879E-46	7
GLRX.3	9.7218E-50	0.30188719	0.689	0.336	3.2914E-45	7
PTPRR.4	1.5268E-46	0.25490799	0.65	0.3	5.169E-42	7
GM2A.4	2.2495E-46	0.37118116	0.83	0.499	7.6159E-42	7
SRGN.2	7.8415E-46	0.33410676	0.99	0.924	2.6548E-41	7
SLPI.4	1.1787E-45	0.38608026	0.969	0.699	3.9905E-41	7
AKR1B1.3	2.0091E-45	0.37501698	0.969	0.731	6.8021E-41	7
SLC20A1.4	2.4385E-42	0.35629397	0.838	0.508	8.2557E-38	7
ATRAID	3.3887E-42	0.27394515	0.99	0.897	1.1473E-37	7
TMSB4X.1	5.6852E-42	0.25533987	1	0.985	1.9248E-37	7
[illegible]	[illegible]	[illegible]	[illegible]	[illegible]	[illegible]	[illegible]

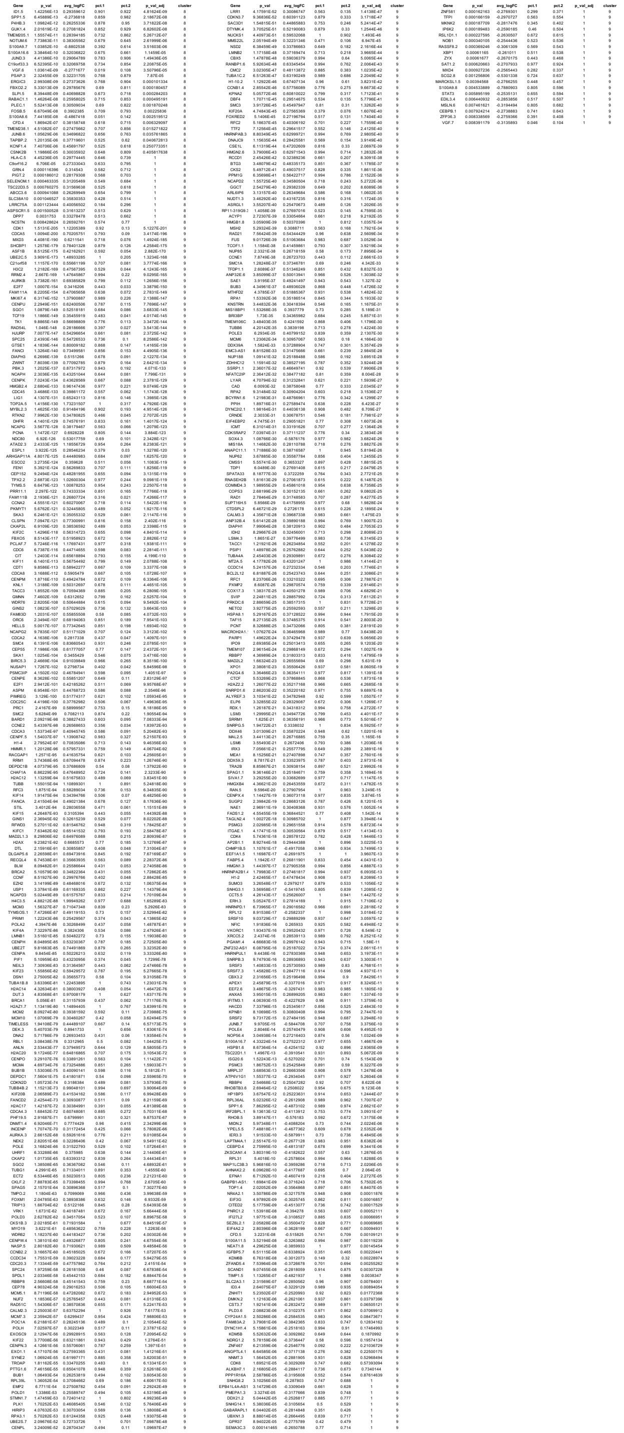

Gene expression clusters defined by scRNAseq in H2030-BrM brain metastasis. 9 clusters with H2030-BrM metastases together with their gene expression patterns are shown. The Seurat function FindAllMarkers was used to find markers specific for each one of the clusters identified, i.e. genes that characterise each population of cells with respect to all others for each obtained

cluster. Differentially expressed genes between the two groups of cells were identified using a non-parametric Wilcoxon Rank Sum test (Seurat default). Pct.1 corresponds to the fraction of cells in column "cluster" in the table, and pct.2 corresponds to the rest of cells in the other clusters/identities. The 'avg_logFC' column is giving the difference found in that gene in that cluster/identity compared to the rest. If the number is positive, it means that is upregulated, and if it is negative, it is downregulated. P_val_adj, adjusted p-value, based on Bonferroni correction using all features in the dataset.

Supplementary Table 8.

Supplementary Table 8_Top 2000 genes with the highest cell-to-cell variation in expression_Corresponds to Fig. S4D

Order	Gene	Mean	Variance.standardized
#1	**S100A9**	11.6733917	30.67273527
#2	**S100A8**	0.930719452	21.94874457
#3	**H4C3**	5.640400964	11.45910826
#4	**TOP2A**	2.820961807	9.949693807
#5	**VGF**	0.317599289	9.859299346
#6	**IGFBP3**	0.506280929	9.111734395
#7	**UBE2C**	1.059383327	8.112935691
#8	**S100P**	0.434462632	7.154256616
#9	**CENPF**	1.632280167	6.142503176
#10	**LTB**	0.031721863	5.516248605
#11	MKI67	1.14833143	5.429362217
#12	TGM2	3.775155437	5.374849886
#13	HP	0.193249588	5.021440824
#14	STC1	2.135642685	4.864548949
#15	CCNB1	1.014845832	4.786691128
#16	RRM2	0.916634945	4.530213809
#17	TUBA1B	5.366958508	4.36049625
#18	SAA1	0.01992133	4.35343259
#19	BCYRN1	1.197436873	4.287025037
#20	AREG	3.479000127	4.257639189
#21	LY6K	0.046694582	4.203580046
#22	TPX2	0.854967644	4.017592906
#23	DLGAP5	0.705748002	4.013255176
#24	IGFL2-AS1	0.26976272	4.008463904
#25	SPARCL1	0.081588631	3.969786296
#26	PTTG1	1.56096942	3.852357776
#27	CDK1	0.403628981	3.80278857
#28	AURKA	0.596497906	3.762884784
#29	GPX2	0.032229413	3.73183267
#30	HSP90AA1	15.65283593	3.684306197
#31	CTD-2126E3.6	1.038573785	3.446537588
#32	PI3	0.03184875	3.441447783
#33	RP11-196G11.3	0.03184875	3.378931561
#34	ATAD2	0.772110138	3.329864529
#35	MT2A	45.86562619	3.297539841
#36	HMGB2	0.694835681	3.284703385
#37	BIRC5	0.887958381	3.258660715
#38	AKAP12	1.221291714	3.235845792
#39	CTSL	3.814871209	3.235419004
[illegible]	[illegible]	[illegible]	[illegible]
#245	NEFL	0.24806497	1.714445736
#246	IL1B	0.00279152	1.714306929
#247	ANXA3	0.76272047	1.713845797
#248	RAD21	1.70054562	1.713586852
#249	SPDL1	0.30351478	1.710734716
#250	EFNA1	0.72528867	1.706695681
#251	HSP90AB1	9.41213044	1.706172249
#252	RNA5SP437	0.00177642	1.705354519
#253	WDR76	0.16000508	1.702864063
#254	SCD	0.81182591	1.700027072
#255	TMPO	0.92932369	1.699767648
#256	RP11-403P17.4	0.03400584	1.698371828
#257	SLC20A1	1.29488644	1.693536144
#258	PRSS56	0.02436239	1.692390424
#259	RFWD3	0.60829844	1.692324585
#260	LRRC75A	0.45793681	1.689829895
#261	S100A16	2.49993656	1.689194095
#262	SYNE2	0.67123461	1.68310166
#263	LYPLAL1-AS1	0.00697881	1.682416246
#264	RFC3	0.26265702	1.680595446
#265	ANLN	0.21659688	1.679625508
#266	GPR183	0.00253775	1.671599125
#267	MCM10	0.0970689	1.670338721
#268	SMOC1	0.09224718	1.669673738
#269	TROAP	0.16051263	1.666496492
#270	RGS2	0.06864611	1.66423291
#271	MSMO1	0.29095292	1.662713672
#272	ARNT2	0.14122573	1.66181657
#273	MT-CO2	75.0019033	1.661578142
#274	CIT	0.26024616	1.660925482
#275	DIAPH3	0.15581779	1.658401459
#276	HBD	0.00266464	1.656891693
#277	KNSTRN	0.25656643	1.652816118
#278	RACGAP1	0.16837965	1.651863305
#279	UCA1	0.00114199	1.651436583
#280	MT-RNR2	26.8358076	1.649871287
#281	TAGLN2	4.51300596	1.649664005
#282	RP11-297N6.4	0.02080954	1.643735116
#283	HLA-C	2.61540414	1.643459311
[illegible]	[illegible]	[illegible]	[illegible]

Top 2000 genes with high variable cell-to-cell expression in H2030-BrM brain metastasis.

Subset of genes that exhibit high cell-to-cell variation in the dataset (i.e., genes with high expression in some cells and low expression in others), obtained after modeling the mean-variance relationship inherent in single-cell data with Seurat. Top 10 genes are in bold.

Supplementary Table 9.

Supplementary Table 9_DEG in cluster 5 vs rest scRNAseq H2030-BrM

Gene	p_val	avg_logFC	pct.1	pct.2	p_val_adj
S100A9	1.1986E-222	2.759257886	0.905	0.571	4.0581E-218
S100A8	1.6171E-147	1.956459241	0.433	0.107	5.4748E-143
CTSL	3.3821E-268	1.302875996	0.985	0.747	1.1451E-263
IGFBP3	0	1.299280688	0.683	0.113	0
MT2A	8.6346E-124	0.953642359	0.997	0.985	2.9233E-119
STC1	8.33601E-98	0.939629756	0.769	0.454	2.82224E-93
S100A11	3.6935E-267	0.814282092	1	0.985	1.2505E-262
FAM83A	3.1357E-120	0.813222162	0.926	0.731	1.0616E-115
C15orf48	3.6152E-160	0.809696976	0.992	0.863	1.224E-155
HSP90AA1	5.4478E-196	0.806142422	1	0.973	1.8444E-191
AREG	4.31711E-75	0.788300277	0.835	0.624	1.4616E-70
LUCAT1	2.1609E-101	0.713124352	0.801	0.495	7.31589E-97
SPP1	1.8232E-171	0.684381677	0.997	0.972	6.1727E-167
FERMT1	1.7419E-75	0.67248022	0.717	0.44	5.89737E-71
RSPO3	2.4532E-152	0.66772189	0.837	0.453	8.3056E-148
TAGLN2	3.9477E-135	0.650876522	0.985	0.869	1.3365E-130
ANGPTL4	1.83425E-82	0.636315602	0.666	0.351	6.21003E-78
TFPI2	3.3304E-161	0.635865506	0.999	0.978	1.1275E-156
IGFBP4	7.7948E-112	0.633160799	0.819	0.496	2.639E-107
TIMP1	1.29854E-78	0.624873869	1	0.987	4.39635E-74
PMEPA1	2.3769E-128	0.609724876	0.95	0.731	8.0474E-124
AC003092.1	5.5417E-165	0.566619185	0.512	0.129	1.8762E-160
IER3	9.97003E-61	0.566331455	0.909	0.718	3.37545E-56
CXCL8	6.9327E-174	0.56511622	0.447	0.093	2.3471E-169
RASD1	1.508E-150	0.555713757	0.477	0.12	5.1053E-146
BCYRN1	1.08595E-59	0.540884992	0.604	0.326	3.67659E-55
PFDN2	6.9839E-159	0.532401519	0.996	0.96	2.3645E-154
COTL1	9.582E-79	0.521952979	0.732	0.417	3.24408E-74
ACTN1	8.94107E-97	0.520932787	0.856	0.581	3.02709E-92
CIB1	8.3972E-112	0.517575759	0.979	0.858	2.843E-107
TM4SF1	1.19645E-60	0.504549987	0.757	0.509	4.05069E-56
TGM2	6.59378E-30	0.50344073	0.78	0.623	2.23239E-25
DKK1	5.4664E-130	0.499351707	0.604	0.214	1.8507E-125
TOP1	7.7313E-103	0.496452857	0.968	0.841	2.61752E-98
ODC1	3.52264E-73	0.484932886	0.933	0.777	1.19262E-68
TPI1	1.7839E-107	0.484607828	0.992	0.914	6.0395E-103
AKAP12	1.33571E-54	0.480349066	0.687	0.421	4.52216E-50
FTH1	8.1215E-115	0.472889602	0.999	0.998	2.7496E-110
TMEM158	2.7565E-130	0.471370017	0.475	0.137	9.3324E-126
SLIRP	1.5447E-109	0.468189822	0.985	0.92	5.2296E-105
ASPH	3.03315E-82	0.456423384	0.906	0.706	1.0269E-77
METTL9	6.55321E-77	0.445328042	0.836	0.579	2.21865E-72
MRPL52	9.26562E-89	0.442449412	0.972	0.839	3.13697E-84
ACTB	3.3211E-120	0.440770727	1	0.997	1.1244E-115
IGSF8	2.62768E-64	0.439280605	0.728	0.448	8.89628E-60
BTG1	1.01678E-71	0.436993508	0.867	0.607	3.44241E-67
CEBPB	5.93126E-54	0.43677747	0.839	0.626	2.00809E-49
ACOX2	1.8372E-82	0.435906297	0.752	0.428	6.22003E-78
MCL1	1.489E-58	0.435905629	0.808	0.585	5.04117E-54
SERPINE2	1.23801E-38	0.434842021	0.673	0.447	4.19142E-34
TNFRSF12A	2.08812E-87	0.433905098	0.488	0.188	7.06953E-83
UPP1	4.8945E-104	0.43123286	0.55	0.205	1.6571E-99
NT5E	1.02424E-94	0.426158476	0.527	0.206	3.46767E-90
TOP2A	1.74276E-33	0.425022882	0.527	0.313	5.9003E-29
MT1X	3.5407E-26	0.42464615	0.665	0.486	1.19874E-21
CITED4	8.0015E-101	0.422741561	0.481	0.16	2.709E-96
DYNC1H1	6.22695E-85	0.420504227	0.978	0.905	2.1082E-80
ISG20	2.32785E-49	0.420315118	0.892	0.724	7.88117E-45
LRP10	1.26572E-44	0.418691238	0.837	0.7	4.28521E-40
CALD1	3.393E-169	0.412947868	0.436	0.085	1.1487E-164
NCL	5.86216E-65	0.412234764	0.973	0.902	1.98469E-60
FXYD5	1.5388E-40	0.411057463	0.78	0.565	5.20977E-36
MOK	3.38915E-91	0.407480003	0.64	0.292	1.14743E-86
POMP	3.73071E-95	0.404637516	0.978	0.866	1.26307E-90
IFITM3	1.39427E-65	0.397113363	0.972	0.906	4.72044E-61
TPM3	3.68765E-83	0.38607286	0.99	0.951	1.24849E-78
DAD1	1.64666E-71	0.385725158	0.942	0.805	5.57492E-67
CA2	3.04262E-70	0.384633576	0.548	0.247	1.03011E-65
PTGR1	6.40452E-60	0.380589908	0.764	0.506	2.16832E-55
HSPD1	7.63655E-57	0.379410769	0.931	0.791	2.58543E-52
HSPE1	1.50199E-58	0.378987424	0.919	0.745	5.08513E-54
PSMA7	1.28875E-98	0.377700546	0.999	0.98	4.36319E-94
AHSA1	3.00749E-60	0.376741386	0.842	0.604	1.01822E-55
SLPI	1.11024E-22	0.376499735	0.849	0.702	3.75881E-18
PRKDC	5.71997E-62	0.36822198	0.951	0.823	1.93655E-57
COPS9	3.27906E-69	0.36574996	0.968	0.885	1.11016E-64
GPX1	3.10918E-73	0.36553489	0.987	0.922	1.05264E-68
PYGL	5.83294E-39	0.364610951	0.581	0.356	1.9748E-34
ARNT2	6.3275E-102	0.360966218	0.318	0.076	2.14225E-97
CHCHD10	4.51326E-57	0.359844826	0.684	0.403	1.52801E-52
GM2A	7.18603E-40	0.359680687	0.722	0.499	2.4329E-35
CKS1B	7.03187E-20	0.356764478	0.781	0.675	2.38071E-15
TCIM	2.1579E-261	0.354739677	0.238	0.009	7.3058E-257
SNRPF	4.37697E-57	0.351463379	0.912	0.75	1.48187E-52
ATP5MC3	2.44929E-61	0.350559567	0.975	0.901	8.2923E-57
SLC20A1	2.85693E-35	0.349863004	0.722	0.509	9.67244E-31
ATP5MJ	9.20705E-53	0.349584722	0.965	0.887	3.11714E-48
SMOC1	4.8475E-148	0.348647956	0.295	0.044	1.6412E-143
PSMD14	1.42612E-53	0.347338321	0.893	0.73	4.82828E-49
BOLA3	3.42707E-51	0.345092661	0.879	0.693	1.16027E-46
CDKN3	1.08006E-36	0.343843367	0.55	0.324	3.65665E-32
DUSP23	2.61517E-38	0.343301677	0.881	0.73	8.85392E-34
GCLM	1.33072E-50	0.342737126	0.652	0.397	4.50527E-46
CALM1	1.80105E-48	0.341763589	0.996	0.974	6.09765E-44
PAPOLA	2.37403E-54	0.339479509	0.847	0.641	8.03753E-50
GNG4	4.86716E-67	0.337436826	0.501	0.217	1.64783E-62
CALB2	1.41925E-46	0.337219081	0.324	0.131	4.80501E-42
CHL1	9.3056E-120	0.33626316	0.424	0.11	3.1505E-115
ATP5MF	6.33621E-63	0.335767967	0.98	0.92	2.14519E-58
PSMA3	4.15021E-50	0.335593798	0.896	0.75	1.4051E-45
TDP2	7.76379E-57	0.333742344	0.534	0.268	2.62851E-52
NDUFC1	6.10268E-65	0.332747739	0.968	0.874	2.06612E-60
TESC	1.57786E-54	0.332112646	0.386	0.159	5.34201E-50
CYTOR	6.24257E-36	0.33156978	0.799	0.616	2.11349E-31
DLGAP5	1.04295E-23	0.326830468	0.348	0.192	3.53102E-19
CCT6A	2.66739E-47	0.32681836	0.79	0.598	9.03073E-43
CCT3	4.63192E-75	0.326111353	0.994	0.949	1.56818E-70
SUPT16H	1.59519E-38	0.324464396	0.83	0.672	5.40066E-34
PHF19	1.51328E-42	0.324378714	0.569	0.312	5.12336E-38
NMB	3.82903E-45	0.324295709	0.783	0.556	1.29636E-40
CNIH1	1.92858E-40	0.323863536	0.827	0.624	6.5294E-36
MAP3K20	1.18045E-42	0.323137109	0.707	0.493	3.99653E-38
MGST1	1.20901E-47	0.321311574	0.961	0.861	4.09323E-43
SDF2L1	9.65051E-36	0.318989507	0.767	0.561	3.26728E-31
APOC1	3.9812E-45	0.316951199	0.694	0.436	1.34787E-40
RHEB	3.17187E-39	0.315950683	0.659	0.43	1.07387E-34
CDCA4	2.86595E-39	0.315868961	0.487	0.265	9.70298E-35
PA2G4	3.12649E-45	0.315400464	0.926	0.81	1.0585E-40
PHLDA2	4.49048E-36	0.315062906	0.672	0.452	1.5203E-31
HMGB3	1.95286E-40	0.313268236	0.554	0.324	6.61159E-36
EIF5B	1.10369E-46	0.311454878	0.924	0.782	3.73665E-42
SNAPC1	2.22294E-46	0.311380534	0.722	0.471	7.52598E-42
SDHC	2.26842E-47	0.308512341	0.962	0.895	7.67995E-43
TPM4	6.14396E-40	0.3069481	0.905	0.78	2.0801E-35
FKBP3	1.05907E-39	0.306245679	0.826	0.659	3.58558E-35
TUBA1B	1.32664E-19	0.305499267	0.839	0.739	4.49146E-15
DTYMK	2.80245E-33	0.304566228	0.527	0.324	9.48797E-29
GLRX	3.82808E-23	0.303747159	0.504	0.343	1.29604E-18
KYNU	1.71207E-43	0.303011504	0.595	0.348	5.79639E-39
GRAMD1B	6.81249E-33	0.301130784	0.884	0.737	2.30644E-28
SAT1	1.9041E-32	0.300945237	0.986	0.92	6.44652E-28
RRM2	7.08355E-24	0.299812711	0.386	0.222	2.39821E-19
DDX24	9.53382E-41	0.299446209	0.919	0.812	3.22777E-36
ZC3H15	1.36273E-40	0.298639839	0.78	0.575	4.61364E-36
TRIB3	7.10664E-48	0.293852702	0.374	0.163	2.40602E-43
EMP3	3.71376E-49	0.292767972	0.983	0.917	1.25733E-44
ANP32E	4.9728E-25	0.291036126	0.684	0.521	1.68359E-20
SIVA1	7.37155E-37	0.290079865	0.867	0.709	2.49571E-32
RAB5IF	5.10758E-35	0.290038319	0.819	0.658	1.72922E-30
LY6K	1.26501E-88	0.289641453	0.136	0.014	4.28282E-84
GAPDH	5.68393E-76	0.289387694	1	0.999	1.92435E-71
PFKP	2.21807E-41	0.288084381	0.59	0.35	7.5095E-37
PLIN3	3.2213E-30	0.287476401	0.67	0.487	1.0906E-25
MESP1	1.62574E-33	0.287328113	0.52	0.315	5.50412E-29
PSMB5	1.55883E-61	0.286913892	0.985	0.949	5.27757E-57
RANBP1	1.58933E-23	0.286521144	0.863	0.757	5.38083E-19
XRCC5	1.95723E-40	0.28640697	0.892	0.787	6.62638E-36
EPAS1	5.95996E-81	0.284604521	0.345	0.101	2.0178E-76
HMGA1	3.77233E-38	0.283423416	0.83	0.625	1.27716E-33
MPHOSPH6	2.37738E-41	0.282838897	0.687	0.441	8.04887E-37
DYNLL1	6.8902E-48	0.282103483	0.979	0.905	2.33275E-43
CXCL3	3.94719E-62	0.281718369	0.222	0.056	1.33636E-57
PTMA	4.8887E-45	0.281515702	0.994	0.99	1.65512E-40
C1QBP	1.23287E-44	0.281096086	0.944	0.836	4.17402E-40
NOP10	2.13894E-46	0.280550352	0.982	0.929	7.24161E-42
SLC25A37	2.41451E-38	0.279709865	0.764	0.55	8.17455E-34
AHNAK2	2.30365E-24	0.279626858	0.806	0.689	7.79923E-20
FKBP4	2.19718E-55	0.279490359	0.492	0.228	7.43877E-51
PFN1	4.77495E-55	0.2791125	0.992	0.974	1.61661E-50
CPS1	5.01882E-36	0.278887272	0.874	0.673	1.69917E-31
CCN2	2.76113E-11	0.278332604	0.387	0.276	9.34807E-07
CREM	1.14449E-40	0.277322911	0.589	0.345	3.8748E-36
ITGA2	4.21192E-14	0.27705465	0.386	0.264	1.42599E-09
CYC1	1.30921E-36	0.277025732	0.938	0.835	4.43247E-32
MIR4435-2HG	9.82811E-37	0.276612056	0.52	0.301	3.32741E-32
RBM8A	2.96629E-43	0.276073218	0.972	0.9	1.00427E-38
VIM	1.02335E-36	0.276058506	0.969	0.904	3.46465E-32
SNRPD1	2.03138E-35	0.275411378	0.9	0.746	6.87743E-31
ANXA1	3.35934E-27	0.273821795	0.937	0.844	1.13734E-22
LDHB	1.74976E-40	0.270335476	0.935	0.836	5.92398E-36
PPIA	3.72583E-55	0.269797191	0.997	0.976	1.26142E-50
CHCHD2	2.02358E-51	0.269654349	0.989	0.938	6.85103E-47
IFITM2	1.14547E-39	0.267445471	0.954	0.877	3.87811E-35
NDUFB3	1.68006E-32	0.267383264	0.851	0.7	5.688E-28
SEC61G	1.12903E-32	0.266807023	0.958	0.877	3.82243E-28
AURKA	4.74328E-25	0.266722578	0.377	0.208	1.60588E-20
UBE2C	4.20251E-17	0.26243942	0.345	0.21	1.4228E-12
HMOX1	9.0115E-27	0.261538564	0.3	0.146	3.05093E-22
MCM7	5.99624E-19	0.26097083	0.568	0.422	2.03009E-14
EIF5A	3.62679E-40	0.260312785	0.971	0.925	1.22789E-35
MT-ND6	1.94062E-26	0.259793735	0.856	0.709	6.57018E-22
CXCL2	6.54966E-67	0.256947348	0.248	0.065	2.21745E-62
STK17A	1.01804E-29	0.256844184	0.506	0.315	3.44669E-25
DBI	2.22486E-32	0.256445077	0.819	0.634	7.5325E-28
PPP2R5C	7.54272E-36	0.255287991	0.604	0.393	2.55366E-31
SGK1	1.74807E-85	0.253574447	0.236	0.048	5.91827E-81
SDC1	1.13068E-20	0.253408448	0.792	0.615	3.82804E-16
HSPB1	5.38822E-38	0.252132426	0.971	0.889	1.82423E-33
UBE2S	7.04465E-14	0.251860228	0.804	0.698	2.38504E-09
CD320	3.41347E-16	0.251830934	0.885	0.799	1.15566E-11
PHLDA1	8.55816E-28	0.251826148	0.743	0.556	2.89745E-23
BZW1	2.6833E-30	0.251675247	0.802	0.633	9.08459E-26
HMGB1	5.76177E-27	0.250018283	0.905	0.807	1.95071E-22
KRAS	6.53728E-32	0.250012944	0.707	0.505	2.21326E-27
BLCAP	6.53812E-19	-0.250093945	0.304	0.455	2.21355E-14
PNISR	4.97383E-19	-0.250613314	0.697	0.758	1.68394E-14
RHOB	1.39282E-08	-0.251830438	0.633	0.674	0.000471552

Gene	p_val	avg_logFC	pct.1	pct.2	p_val_adj
HSPA9	1.6135E-13	-0.25286446	0.871	0.867	5.4626E-09
ZKSCAN1	1.3696E-15	-0.25361499	0.554	0.636	4.637E-11
RPS6KB2	8.2009E-19	-0.25369846	0.341	0.475	2.7765E-14
KANK2	1.5254E-15	-0.25395328	0.488	0.583	5.1643E-11
CPE	1.6394E-20	-0.25406319	0.213	0.37	5.5505E-16
TENT5A	1.41E-12	-0.25464747	0.438	0.537	4.7738E-08
AAMDC	4.3028E-17	-0.25472272	0.243	0.383	1.4568E-12
UBE2H	1.7675E-16	-0.25486968	0.505	0.598	5.9841E-12
FZD10	5.062E-21	-0.25665888	0.16	0.318	1.7138E-16
SOX4	2.8428E-24	-0.25752261	0.979	0.982	9.6246E-20
SQSTM1	9.0026E-23	-0.25799894	0.799	0.837	3.0479E-18
TNRC6B	2.4233E-18	-0.25837441	0.651	0.715	8.2045E-14
SERINC5	6.1351E-17	-0.25898431	0.203	0.342	2.0771E-12
RNF217	5.4443E-25	-0.25986438	0.151	0.329	1.8432E-20
GADD45A	7.2218E-24	-0.26133433	0.154	0.327	2.445E-19
ZFAS1	9.381E-37	-0.2619464	0.95	0.958	3.176E-32
DYNLL2	1.7782E-20	-0.26344386	0.23	0.386	6.0203E-16
PPP1R15A	2.3793E-18	-0.26380755	0.349	0.488	8.0553E-14
TAPBP	1.575E-16	-0.26488686	0.539	0.622	5.3322E-12
PCSK7	1.7827E-16	-0.2651146	0.439	0.539	6.0354E-12
LMAN2	6.9714E-25	-0.26584626	0.799	0.829	2.3602E-20
PABPC1	8.0447E-37	-0.26962215	0.958	0.971	2.7236E-32
PGLS	3.3599E-21	-0.2698296	0.578	0.683	1.1375E-16
EIF4B	5.8985E-22	-0.27026639	0.647	0.732	1.997E-17
UBXN1	6.5508E-21	-0.27182546	0.652	0.726	2.2178E-16
TNS3	2.6579E-23	-0.27184959	0.238	0.413	8.9985E-19
ALDH3A2	2.5469E-17	-0.27257035	0.509	0.601	8.6228E-13
PIGT	8.1137E-20	-0.2729902	0.634	0.701	2.747E-15
FOS	1.6616E-29	-0.2748287	0.961	0.964	5.6256E-25
ANXA2	1.1381E-53	-0.27520733	0.993	0.989	3.8531E-49
RHOBTB2	1.5209E-29	-0.2755603	0.097	0.287	5.1491E-25
TUBA1A	1.0616E-13	-0.27585857	0.29	0.405	3.5942E-09
CMTM4	6.6628E-21	-0.27655073	0.432	0.561	2.2557E-16
ITGB4	1.3923E-19	-0.27715241	0.452	0.579	4.7139E-15
PCNX4	6.21E-13	-0.27829619	0.487	0.569	2.1024E-08
TMC4	8.6101E-33	-0.27858556	0.027	0.21	2.915E-28
KLF2	5.5901E-09	-0.27905897	0.401	0.472	0.00018926
RPS4Y1	1.6944E-68	-0.27918593	1	0.998	5.7366E-64
LIMCH1	2.0082E-42	-0.27919913	0.02	0.244	6.7989E-38
SSBP4	1.7409E-21	-0.28003329	0.603	0.696	5.8938E-17
GUK1	2.6892E-34	-0.28180058	0.902	0.927	9.1046E-30
PPP3CA	6.741E-19	-0.2827346	0.403	0.532	2.2822E-14
RNF19A	5.1621E-19	-0.28607639	0.505	0.612	1.7477E-14
SELENOM	5.8851E-21	-0.28749487	0.589	0.685	1.9925E-16
HMGN3	4.4045E-26	-0.28784422	0.575	0.705	1.4912E-21
TM7SF2	1.5669E-23	-0.2891359	0.216	0.393	5.305E-19
KCNF1	4.8641E-11	-0.28988903	0.578	0.616	1.6468E-06
NDN	3.581E-25	-0.29019311	0.356	0.522	1.2124E-20
RP11-158I9.8	1.4774E-28	-0.29047771	0.163	0.359	5.0017E-24
ATOH8	6.9801E-34	-0.29075506	0.045	0.241	2.3632E-29
SNHG7	4.3389E-20	-0.29180891	0.419	0.549	1.469E-15
MAP1B	6.7856E-21	-0.29959383	0.243	0.396	2.2973E-16
CHMP2A	1.9692E-38	-0.3000052	0.84	0.883	6.667E-34
TPBG	8.4476E-23	-0.30053833	0.505	0.633	2.86E-18
ZFAND2A-DT	1.1739E-25	-0.30103474	0.286	0.46	3.9743E-21
CMBL	5.8011E-31	-0.30116627	0.771	0.842	1.964E-26
FKBP5	3.3284E-41	-0.3024191	0.027	0.246	1.1269E-36
TCEAL3	4.2813E-32	-0.30275419	0.151	0.359	1.4495E-27
EGR1	1.2421E-21	-0.30356072	0.724	0.817	4.2052E-17
DAP	1.2206E-28	-0.30412978	0.526	0.663	4.1325E-24
ALDH3B1	4.5822E-24	-0.30475286	0.355	0.509	1.5513E-19
BAG1	4.3338E-25	-0.3054037	0.649	0.731	1.4672E-20
ZFAND5	2.8201E-22	-0.30580461	0.609	0.703	9.5477E-18
SNHG8	6.5956E-26	-0.30680973	0.612	0.697	2.233E-21
NOL3	4.5282E-30	-0.30684859	0.554	0.685	1.5331E-25
F2R	8.2616E-35	-0.30708237	0.097	0.31	2.7971E-30
RPAIN	8.3785E-29	-0.30741389	0.604	0.725	2.8366E-24
LMNA	7.9822E-54	-0.30892153	0.979	0.975	2.7025E-49
CFD	3.7056E-14	-0.30904663	0.658	0.714	1.2546E-09
CPLANE1	3.1451E-26	-0.31028765	0.321	0.485	1.0648E-21
LENG8	1.6844E-21	-0.31138262	0.439	0.574	5.7026E-17
ST13	1.9182E-31	-0.3114994	0.642	0.736	6.4944E-27
SEMA3C	2.4475E-25	-0.31171876	0.624	0.724	8.2863E-21
ST3GAL4	1.5534E-29	-0.31239378	0.215	0.407	5.2591E-25
RNF145	4.7656E-32	-0.31316191	0.736	0.797	1.6135E-27
PBXIP1	8.0273E-29	-0.31348158	0.173	0.366	2.7177E-24
RPL23	6.246E-43	-0.31387402	0.993	0.989	2.1147E-38
FOSL2	1.2792E-22	-0.31779904	0.565	0.669	4.3308E-18
SH3BP5	1.7995E-34	-0.31807903	0.073	0.279	6.0924E-30
TCF25	4.7182E-45	-0.3182065	0.874	0.904	1.5974E-40
SNHG14	3.6947E-25	-0.3185008	0.381	0.543	1.2509E-20
RPL10A	2.1352E-57	-0.31918792	0.987	0.988	7.229E-53
CKB	2.3238E-26	-0.31996528	0.156	0.337	7.8676E-22
AC005256.1	2.223E-35	-0.32128746	0.111	0.331	7.5262E-31
VMP1	2.3966E-29	-0.32199145	0.714	0.806	8.1141E-25
PRTG	2.9549E-26	-0.32339058	0.342	0.517	1.0004E-21
DANCR	1.5843E-28	-0.32348024	0.506	0.651	5.3637E-24
PDK4	2.5637E-25	-0.32429978	0.144	0.325	8.6798E-21
PSMB8	2.9164E-31	-0.32564443	0.25	0.449	9.8737E-27
S100A14	4.1277E-31	-0.32663542	0.041	0.222	1.3975E-26
RP11-574K11.2	7.313E-33	-0.33029557	0.163	0.372	2.4759E-28
RBM3	5.763E-48	-0.3308076	0.871	0.908	1.9511E-43
EEF1A1	5.651E-94	-0.33206163	1	1	1.9132E-89
PER1	1.1619E-29	-0.33267403	0.251	0.451	3.9337E-25
POLR2I	2.0356E-42	-0.33373222	0.919	0.933	6.8919E-38
IER2	3.1968E-23	-0.33418508	0.739	0.798	1.0823E-18
MFGE8	1.3569E-31	-0.33453483	0.327	0.527	4.5939E-27
CENPX	7.3369E-36	-0.33567805	0.777	0.844	2.484E-31
DDX5	9.806E-40	-0.33855473	0.861	0.896	3.3199E-35
JUND	1.528E-35	-0.33947512	0.851	0.904	5.1731E-31
SNHG16	1.0674E-37	-0.33988448	0.628	0.74	3.6137E-33
SSR4	6.7611E-48	-0.34194466	0.948	0.961	2.289E-43
PNRC1	3.383E-29	-0.34476544	0.466	0.621	1.1454E-24
CCND3	1.6229E-31	-0.34555368	0.171	0.368	5.4946E-27
SPATA20	5.4893E-31	-0.34603821	0.457	0.602	1.8585E-26
TP53I3	1.0132E-31	-0.34621622	0.431	0.595	3.4302E-27
CRIM1	1.9561E-31	-0.34767162	0.386	0.568	6.6225E-27
JUN	2.3101E-13	-0.3477653	0.436	0.525	7.8211E-09
SSBP3	3.8992E-37	-0.35333166	0.184	0.408	1.3201E-32
EPHX1	2.1126E-34	-0.35333609	0.237	0.452	7.1525E-30
RPL35A	2.454E-91	-0.35557852	0.997	0.997	8.3084E-87
SLC2A1	1.9068E-24	-0.36402189	0.374	0.51	6.4557E-20
NOP53	9.1669E-36	-0.36524023	0.648	0.775	3.1035E-31
JUNB	6.0251E-20	-0.36912758	0.732	0.759	2.0399E-15
PDXK	2.8353E-36	-0.37063776	0.425	0.598	9.5991E-32
SYNM	2.0089E-32	-0.37191493	0.32	0.512	6.8014E-28
P4HB	4.1294E-60	-0.37448685	0.921	0.948	1.3981E-55
ID2	4.047E-24	-0.37552656	0.461	0.603	1.3702E-19
PDP1	3.3394E-36	-0.37619495	0.247	0.467	1.1306E-31
NR4A1	2.8311E-39	-0.37646145	0.687	0.809	9.5851E-35
CA11	2.9782E-37	-0.3790867	0.224	0.446	1.0083E-32
KRT8	4.5579E-36	-0.38249268	0.938	0.941	1.5431E-31
CYP4F3	1.4656E-39	-0.38600071	0.202	0.438	4.962E-35
CHMP1B	7.1699E-43	-0.38706495	0.893	0.939	2.4274E-38
C9orf16	6.2964E-45	-0.39454226	0.677	0.795	2.1317E-40
MAP2	2.6707E-27	-0.39663294	0.205	0.392	9.0418E-23
MIR23AHG	1.745E-27	-0.39813934	0.428	0.581	5.908E-23
TMEM238	7.1193E-56	-0.39946597	0.762	0.855	2.4103E-51
TOM1L2	5.3264E-43	-0.39951909	0.175	0.424	1.8033E-38
ZNF503	1.0112E-29	-0.40716998	0.338	0.51	3.4236E-25
GRN	1.8485E-43	-0.40853215	0.546	0.72	6.2583E-39
CD24	1.2073E-30	-0.41248387	0.243	0.442	4.0874E-26
PDE10A	1.21E-40	-0.41955063	0.466	0.651	4.0965E-36
PTPRR	1.9484E-63	-0.42431284	0.041	0.35	6.5964E-59
FBXO2	2.402E-56	-0.42655979	0.68	0.816	8.1322E-52
EEF2	6.8004E-89	-0.42947191	0.975	0.986	2.3023E-84
ID1	1.4517E-45	-0.43142391	0.926	0.92	4.9149E-41
CASC15	1.2541E-53	-0.43271413	0.77	0.842	4.2458E-49
PSAP	2.2596E-62	-0.43569071	0.79	0.88	7.6501E-58
CLU	9.0752E-64	-0.43656293	0.9	0.948	3.0725E-59
ZFP36L1	3.9586E-32	-0.44054155	0.56	0.686	1.3402E-27
FGFBP1	4.9189E-48	-0.44308286	0.069	0.327	1.6653E-43
CDKN1C	1.5645E-41	-0.44601392	0.087	0.322	5.2967E-37
PLD3	3.4999E-53	-0.44715731	0.766	0.874	1.1849E-48
EPB41L4A-AS1	5.1006E-47	-0.45302227	0.445	0.647	1.7269E-42
PDLIM1	2.372E-51	-0.45315274	0.174	0.441	8.0305E-47
B2M	9.4086E-55	-0.45446903	0.957	0.953	3.1854E-50
DDX59	2.3402E-55	-0.45514122	0.154	0.437	7.923E-51
S100A13	6.6861E-50	-0.4579275	0.318	0.585	2.2637E-45
FTL	6.199E-106	-0.45954669	0.999	0.999	2.099E-101
GLG1	1.9995E-74	-0.46316206	0.856	0.91	6.7693E-70
CIRBP	5.6059E-67	-0.46970521	0.698	0.84	1.8979E-62
SNHG29	2.544E-116	-0.47824432	0.994	0.996	8.612E-112
ABCC3	1.4618E-53	-0.48457265	0.683	0.801	4.9492E-49
TSC22D3	3.5734E-41	-0.4880484	0.443	0.63	1.2098E-36
CEACAM6	2.5776E-33	-0.48990045	0.165	0.373	8.7266E-29
SH3KBP1	1.7527E-57	-0.49279654	0.401	0.637	5.9339E-53
CST3	3.0125E-79	-0.50392268	0.955	0.973	1.0199E-74
ASPSCR1	2.4367E-59	-0.5051558	0.393	0.645	8.2497E-55
MAL2	1.403E-77	-0.51282589	0.036	0.392	4.7501E-73
YPEL5	2.2563E-52	-0.51408046	0.509	0.693	7.6389E-48
SNTB2	5.7238E-64	-0.53075164	0.539	0.745	1.9379E-59
SPTAN1	1.6847E-84	-0.54606354	0.668	0.831	5.7038E-80
RHOBTB3	1.1062E-60	-0.55110838	0.511	0.698	3.7451E-56
CCL2	2.0921E-59	-0.5531409	0.091	0.399	7.083E-55
SNHG19	7.6339E-77	-0.56662856	0.825	0.89	2.5845E-72
DUSP1	1.0468E-54	-0.57374415	0.847	0.861	3.5441E-50
IL18	1.521E-76	-0.57429627	0.511	0.732	5.1495E-72
MSLN	6.6314E-66	-0.59313398	0.447	0.708	2.2451E-61
DCBLD2	1.3243E-82	-0.60150478	0.39	0.714	4.4836E-78
APLP2	3.033E-117	-0.60178395	0.871	0.943	1.027E-112
SUSD2	6.5715E-60	-0.63304908	0.439	0.653	2.2248E-55
TPD52L1	4.8738E-78	-0.64256686	0.428	0.68	1.6501E-73
BMP7	1.7215E-91	-0.65220197	0.351	0.699	5.8284E-87
NEAT1	9.9122E-89	-0.65648337	0.976	0.977	3.3559E-84
SELENOP	2.6394E-92	-0.67299649	0.165	0.558	8.936E-88
RPN2	5.878E-123	-0.67491859	0.933	0.964	1.99E-118
GAS5	9.6555E-80	-0.68213286	0.68	0.84	3.269E-75
SYCE1L	3.466E-86	-0.7060473	0.575	0.775	1.1734E-81
SNHG25	6.193E-108	-0.71629776	0.464	0.76	2.097E-103
IGFBP5	2.3785E-62	-0.72553361	0.177	0.491	8.0525E-58
S100A16	1.4783E-86	-0.72911754	0.39	0.689	5.0051E-82
MALAT1	9.0413E-95	-0.73730296	0.997	0.998	3.061E-90
HLA-C	1.7294E-90	-0.73826307	0.509	0.756	5.8551E-86
FOSB	6.4896E-98	-0.8136978	0.574	0.81	2.1971E-93
HLA-B	8.514E-83	-0.83714514	0.278	0.605	2.8825E-78
HLA-A	1.212E-198	-0.91935808	0.888	0.969	4.105E-194
MUC1	4.256E-133	-0.93756583	0.717	0.873	1.441E-128
S100A4	8.262E-169	-1.19208707	0.937	0.956	2.797E-164
LGALS3BP	1.458E-185	-1.22099027	0.271	0.774	4.935E-181
NOTUM	7.837E-147	-1.24663558	0.236	0.688	2.653E-142

DEG in Cluster 5 as defined by scRNAseq in H2030-BrM brain metastasis. Genes differentially expressed by cancer cells contained in cluster 5 respect to the expression of the same genes in the rest of the other clusters. The Seurat function FindAllMarkers was used to find markers specific for each one of the clusters identified, i.e. genes that characterise each population

of cells with respect to all others for each obtained cluster. Differentially expressed genes between the two groups of cells were identified using a non-parametric Wilcoxon Rank Sum test (Seurat default). Pct.1 corresponds to the fraction of cells in column "cluster" in the table, and pct.2 corresponds to the rest of cells in the other clusters/identities. The 'avg_logFC' column is giving the difference found in that gene in that cluster/identity compared to the rest. If the number is positive, it means that is upregulated, and if it is negative, it is downregulated. P_val_adj, adjusted p-value, based on Bonferroni correction using all features in the dataset.

Supplementary Table 10.

Supplementary Table 10_GSEA top 25 in cluster 5 from H2030-BrM brain metastasis scRNAseq_Corresponds to Fig. 4E

UP				
**ID pathway**	**Pathway**	**NES**	**Nominal p-value**	**FDR q-value**
#1	HALLMARK_E2F_TARGETS	2.1856139	0	0
#2	HALLMARK_G2M_CHECKPOINT	2.0777059	0	0
#3	HALLMARK_MYC_TARGETS_V1	2.055294	0	0
#4	REACTOME_CELL_CYCLE_CHECKPOINTS	1.8397183	0	0.20451927
#5	WONG_EMBRYONIC_STEM_CELL_CORE	1.8363819	0	0.001
#6	REACTOME_RHO_GTPASES_ACTIVATE_NADPH_OXIDASES	1.809974	0	0.19855398
#7	REACTOME_ACTIVATION_OF_THE_PRE_REPLICATIVE_COMPLEX	1.8083675	0	0.13883011
#8	REACTOME_DNA_REPLICATION_PRE_INITIATION	1.8076754	0	0.10670663
#9	PID_TOLL_ENDOGENOUS_PATHWAY	1.7938244	0.002475248	0.10997319
#10	REACTOME_DNA_REPLICATION	1.7926991	0	0.09312679
#11	REACTOME_DISEASES_OF_IMMUNE_SYSTEM	1.7911379	0	0.083911404
#12	HALLMARK_EPITHELIAL_MESENCHYMAL_TRANSITION	1.7806511	0	0.003550326
#13	PID_ATR_PATHWAY	1.7769037	0.002512563	0.09429684
#14	REACTOME_SEPARATION_OF_SISTER_CHROMATIDS	1.7714736	0	0.09129486
#15	REACTOME_RHO_GTPASE_EFFECTORS	1.7702826	0	0.083704375
#16	REACTOME_REGULATION_OF_TLR_BY_ENDOGENOUS_LIGAND	1.7702146	0	0.07609489
#17	REACTOME_IRAK4_DEFICIENCY_TLR2_4	1.7552631	0	0.09142979
#18	REACTOME_METABOLISM_OF_POLYAMINES	1.7471399	0.002487562	0.0946637
#19	REACTOME_G2_M_CHECKPOINTS	1.7469846	0	0.08826875
#20	PID_AURORA_B_PATHWAY	1.7385776	0	0.09463558
#21	HALLMARK_MYC_TARGETS_V2	1.7350991	0	0.003878916
#22	REACTOME_DNA_STRAND_ELONGATION	1.7273165	0.002444988	0.10496363
#23	HALLMARK_GLYCOLYSIS	1.7228379	0	0.004394914
#24	KEGG_PROTEASOME	1.7186202	0.002444988	0.114876986
#25	REACTOME_ACTIVATION_OF_ATR_IN_RESPONSE_TO_REPLICATION_STRESS	1.7185838	0	0.108565256

DOWN				
**ID pathway**	**Pathway**	**NES**	**Nominal p-value**	**FDR q-value**
#1	REACTOME_NUCLEAR_EVENTS_KINASE_AND_TRANSCRIPTION_FACTOR_ACTIVATION	-1.5547309	0.006568145	0.24929775
#2	REACTOME_EUKARYOTIC_TRANSLATION_INITIATION	-1.56283	0.001644737	0.22521368
#3	REACTOME_TIGHT_JUNCTION_INTERACTIONS	-1.5694934	0.005454545	0.20631562
#4	REACTOME_RESPONSE_OF_EIF2AK4_GCN2_TO_AMINO_ACID_DEFICIENCY	-1.573468	0	0.20123662
#5	WP_SLEEP_REGULATION	-1.5747778	0.001658375	0.20749201
#6	KEGG_CELL_ADHESION_MOLECULES_CAMS	-1.577083	0	0.21109092
#7	PID_CD8_TCR_DOWNSTREAM_PATHWAY	-1.5795228	0.001686341	0.21431546
#8	REACTOME_NONSENSE_MEDIATED_DECAY_NMD	-1.5991155	0.001577287	0.14895424
#9	REACTOME_COMPLEMENT_CASCADE	-1.6243877	0.006622517	0.08970343
#10	REACTOME_ANTIGEN_PRESENTATION_FOLDING_ASSEMBLY_AND_PEPTIDE_LOADING_OF_CLASS_I_MHC	-1.659439	0	0.03996695
#11	REACTOME_SELENOAMINO_ACID_METABOLISM	-1.676544	0	0.028492492
#12	REACTOME_SRP_DEPENDENT_COTRANSLATIONAL_PROTEIN_TARGETING_TO_MEMBRANE	-1.7009441	0	0.014255231
#13	WP_HEMATOPOIETIC_STEM_CELL_DIFFERENTIATION	-1.7051486	0	0.013734876
#14	KEGG_RIBOSOME	-1.7070721	0	0.014326864
#15	KEGG_ALLOGRAFT_REJECTION	-1.7127821	0	0.014591277
#16	KEGG_GRAFT_VERSUS_HOST_DISEASE	-1.7215322	0	0.013642061
#17	KEGG_AUTOIMMUNE_THYROID_DISEASE	-1.7374891	0	0.010454046
#18	REACTOME_EUKARYOTIC_TRANSLATION_ELONGATION	-1.7566689	0	0.006701358
#19	WP_CYTOPLASMIC_RIBOSOMAL_PROTEINS	-1.7635871	0	0.00643553
#20	REACTOME_IMMUNOREGULATORY_INTERACTIONS_BETWEEN_A_LYMPHOID_AND_A_NON_LYMPHOID_CE	-1.8271065	0	8.07E-04

Cell cycle
Embryonic stem cells
Rho GTPases
DNA replication & elongation
EMT
ATR
Glycolysis

Ribosomes & translation
Cell junctions
Complement
Immune system
Stem cell differentiation

GSEA in Cluster 5 as defined by scRNAseq in H2030-BrM brain metastasis. The GSEAPreranked module was used to conduct gene set enrichment analysis on a pre-ranked gene list. GSEAPreranked calculates an enrichment score for each gene set. A gene set’s enrichment score reflects how often members of that gene set occur at the top or bottom of the ranked data set. Top 15 signatures up or down according to NES (FDR q value <0.25 and nominal p-value <0.01) in cluster 5.

Supplementary Table 11.

Supplementary Table 11_Common DEG in S100A9high patients and radioresistant surrogates_Corresponds to Fig. 4G

DE genes from S100A9 high vs. S100A9 low human breast-to-brain metastasis
count matrix retrieved from: https://github.com/npriedig/jnci_2018/ p-adj alpha = 0.05 NAs were excluded (from either p-adjust thresholding or no gene symbol was matched to ENSEMBL ID)
DE was done with DESeq2. Samples were grouped to S100A9 expression by median. Lowly expressed genes (< 10 counts per gene in all samples) were excluded prior to analysis. experimental design = ~ S100A9

Upregulated genes in S100A9high patients also upregulated in radioresistant surrogates
Only genes with p<0.05

gene_id	Comparison	sample_2	FPKM_2	sample_1	FPKM_1	log2(fold_change)	test_stat	p_value	q_value	significant
S100A8	OncCocult_vs_AdhInsert	OncCocult	33.0004	AdhInsert	1.19231	4.79065	6.16617	5.00E-05	0.00271768	yes
S100A9	OncCocult_vs_AdhInsert	OncCocult	590.45	AdhInsert	47.3804	3.63945	7.16764	5.00E-05	0.00271768	yes
FAM83A	OncCocult_vs_AdhInsert	OncCocult	14.2647	AdhInsert	2.3703	2.58931	5.6807	5.00E-05	0.00271768	yes
FGFBP1	OncCocult_vs_AdhInsert	OncCocult	6.09638	AdhInsert	0.770794	2.98353	4.78436	5.00E-05	0.00271768	yes
ACOX2	OncCocult_vs_AdhInsert	OncCocult	30.6968	AdhInsert	19.7229	0.638215	1.95463	5.00E-05	0.00271768	yes
CD55	OncCocult_vs_AdhInsert	OncCocult	30.505	AdhInsert	20.1764	0.596372	1.77885	2.00E-04	0.00912696	yes
S100A8	brain slice_vs_Adh	brain slice	22.492	Adh	0.605885	5.21422	8.31405	5.00E-05	0.00035859	yes
S100A9	brain slice_vs_Adh	brain slice	1966.36	Adh	40.0092	5.61906	24.8678	5.00E-05	0.00035859	yes
FAM83A	brain slice_vs_Adh	brain slice	5.98214	Adh	0.596183	3.32683	10.5008	5.00E-05	0.00035859	yes
FGFBP1	brain slice_vs_Adh	brain slice	2.69569	Adh	1.19473	1.17398	3.52581	5.00E-05	0.00035859	yes
ACOX2	brain slice_vs_Adh	brain slice	47.2336	Adh	28.786	0.714446	3.35191	5.00E-05	0.00035859	yes
CD55	brain slice_vs_Adh	brain slice	38.6092	Adh	28.3874	0.443693	2.09061	0.0025	0.0102455	yes

ENSEMBL Ids	Names	baseMean	log2FoldChar	lfcSE	stat	pvalue	padj
ENSG00000171209	CSN3	414.619853	8.43894966	2.42797148	3.47572027	0.00050948	0.03212007
ENSG00000142619	PADI3	326.123255	7.67539913	1.10671409	6.93530444	4.05E-12	1.96E-08
ENSG00000143556	S100A7	1667.74213	7.0725007	1.05716888	6.69003868	2.23E-11	7.70E-08
ENSG00000143546	S100A8	1470.35538	6.8728415	0.75802452	9.06677989	1.23E-19	1.48E-15
ENSG00000171564	FGB	13.6705217	6.58121724	1.22923935	5.35389404	8.61E-08	9.90E-05
ENSG00000184330	S100A7A	25.3720543	6.51525222	1.35298321	4.8154716	1.47E-06	0.00082463
ENSG00000163220	S100A9	1287.92003	6.18231906	0.67858065	9.11066214	8.19E-20	1.48E-15
ENSG00000131668	BARX1	80.445599	6.11282541	1.07589439	5.68162216	1.33E-08	2.93E-05
ENSG00000147381	MAGEA4	120.219743	5.99937543	1.48729608	4.03374655	5.49E-05	0.0093731
ENSG00000173809	TDRD12	873.201795	5.94051979	0.96875788	6.13209959	8.67E-10	2.62E-06
ENSG00000197172	MAGEA6	371.820304	5.59073574	1.51670452	3.68610739	0.00022771	0.02126194
ENSG00000164485	IL22RA2	17.396777	5.59009854	1.1434941	4.88861163	1.02E-06	0.00062872
ENSG00000144820	ADGRG7	36.9876456	5.58018188	1.19311176	4.67699848	2.91E-06	0.00135173
ENSG00000206073	SERPINB4	7.43368916	5.38308531	1.36006958	3.95794846	7.56E-05	0.01112131
ENSG00000163218	PGLYRP4	11.6984437	5.01933435	1.07021752	4.69001326	2.73E-06	0.00132998
ENSG00000171557	FGG	7.07570806	4.94827286	1.19563451	4.13861663	3.49E-05	0.00734742
ENSG00000164093	PITX2	5.17349703	4.89509991	1.14929411	4.259223	2.05E-05	0.005384
ENSG00000243955	GSTA1	389.549043	4.83994126	1.25786631	3.84773901	0.00011921	0.01422081
ENSG00000143452	HORMAD1	168.371454	4.83842881	0.89492317	5.40652982	6.43E-08	8.17E-05
ENSG00000187268	FAM9C	29.5183185	4.73759291	1.37685122	3.44088949	0.00057981	0.03439798
ENSG00000164120	HPGD	511.258377	4.65633719	1.04923041	4.43785957	9.09E-06	0.00295686
ENSG00000130600	H19	4069.09319	4.6230221	0.90537035	5.10622212	3.29E-07	0.00029392
ENSG00000178372	CALML5	330.095044	4.54958557	1.04027678	4.37343758	1.22E-05	0.0036459
ENSG00000274391	TPTE	18.1507995	4.4581339	1.31293533	3.39554722	0.00068492	0.03793113
ENSG00000146857	STRA8	27.653481	4.43188724	1.09334973	4.05349461	5.05E-05	0.00902491
ENSG00000178690	DYNAP	6.84708202	4.39014821	1.08692502	4.03905341	5.37E-05	0.0093731
ENSG00000198788	MUC2	3948.56871	4.31391846	1.18480501	3.64103665	0.00027154	0.02319494
ENSG00000009709	PAX7	9.42741916	4.30554636	1.24210591	3.46632788	0.00052762	0.03241705
ENSG00000163209	SPRR3	22.9103591	4.29943842	1.2882407	3.33744961	0.00084551	0.04252457
ENSG00000136695	IL36RN	11.6969031	4.19945482	1.05326806	3.98707129	6.69E-05	0.01056259
ENSG00000137440	FGFBP1	6.33745623	4.19697422	1.05137677	3.99188412	6.56E-05	0.01055187
ENSG00000185479	KRT6B	2079.85017	4.15756427	0.82000378	5.07017696	3.97E-07	0.00032672
ENSG00000171234	UGT2B7	76.2755397	4.09835042	1.21552412	3.37167346	0.00074713	0.03973611
ENSG00000089225	TBX5	24.595807	4.06350737	1.14907073	3.53634224	0.00040571	0.02897859
ENSG00000183733	FIGLA	9.31765732	4.05251131	1.14242302	3.54729485	0.00038921	0.02847249
ENSG00000124143	ARHGAP40	105.600543	4.03925017	1.004393	4.02158336	5.78E-05	0.00962647
ENSG00000187533	PRR27	31.8340866	4.02896808	1.09811566	3.66898336	0.00024352	0.02185859
ENSG00000119915	ELOVL3	10.2313131	4.00633162	1.18699252	3.37519533	0.00073763	0.03958882
ENSG00000169429	CXCL8	173.694851	3.89839482	0.70862882	5.50132133	3.77E-08	5.69E-05
ENSG00000147573	TRIM55	60.0426628	3.89755786	0.97197507	4.00993606	6.07E-05	0.00987922
ENSG00000268864	LOC284379	22.374	3.72154104	0.99914998	3.72470711	0.00019554	0.01959154
ENSG00000166535	A2ML1	766.861643	3.67559423	0.66434331	5.53267287	3.15E-08	5.44E-05
ENSG00000268104	SLC6A14	565.616529	3.65148754	1.08144752	3.37648149	0.00073419	0.03957104
ENSG00000206075	SERPINB5	145.269443	3.65144509	1.07786651	3.38765985	0.00070492	0.038772
ENSG00000163207	IVL	45.6134098	3.61145121	1.04398981	3.45927821	0.00054163	0.0328595
ENSG00000113396	SLC27A6	61.6073033	3.57265834	1.01550575	3.51810745	0.00043464	0.02989951
ENSG00000196549	MME	239.288483	3.53991577	0.66801273	5.29917407	1.16E-07	0.00012767
ENSG00000131686	CA6	16.640248	3.53640962	0.9425332	3.75202656	0.00017541	0.01802328
ENSG00000115009	CCL20	24.5923721	3.5151322	0.68739654	5.1136891	3.16E-07	0.0002934
ENSG00000138678	GPAT3	228.731495	3.41277898	0.71984843	4.74096887	2.13E-06	0.00114129
ENSG00000214336	FOXI3	15.4298545	3.40578511	0.87417978	3.89597793	9.78E-05	0.01229978
ENSG00000197506	SLC28A3	137.585254	3.34350166	0.84963949	3.93520039	8.31E-05	0.0113104
ENSG00000162624	LHX8	8.31426509	3.30702988	1.00223864	3.29964318	0.00096808	0.04512661
ENSG00000204021	LIPK	13.3369327	3.30238349	1.00126811	3.298201	0.00097306	0.04512661
ENSG00000002726	AOC1	89.3360254	3.23817362	0.92769545	3.49055676	0.00048202	0.03111962
ENSG00000169550	MUC15	56.0741794	3.18564352	0.84041687	3.79055161	0.00015031	0.01620295
ENSG00000173432	SAA1	92.1838822	3.18383208	0.87386963	3.64337193	0.00026909	0.02312255
ENSG00000108688	CCL7	6.59354045	3.17726216	0.7542656	4.21239169	2.53E-05	0.00597827
ENSG00000112761	CCN6	56.5192268	3.17610516	0.6465071	4.91271506	8.98E-07	0.00060247
ENSG00000174808	BTC	58.608004	3.16390353	0.55968018	5.65305625	1.58E-08	3.17E-05
ENSG00000214842	RAD51AP2	4.00526459	3.14460803	0.85638017	3.6719767	0.00024068	0.02176591
ENSG00000143278	F13B	3.88961786	3.11533508	0.79811812	3.90335087	9.49E-05	0.01218474
ENSG00000110195	FOLR1	260.724969	3.05727565	0.77478116	3.94598603	7.95E-05	0.0112632
ENSG00000174502	SLC26A9	59.8947616	3.05304458	0.92572112	3.29801763	0.0009737	0.04512661
ENSG00000196542	SPTSSB	94.5500698	3.04701638	0.84708171	3.59707492	0.00032182	0.02556107
ENSG00000128422	KRT17	2579.75436	3.03040347	0.86221101	3.51468892	0.00044027	0.02998857
ENSG00000124610	H1-1	502.868171	2.93591799	0.86242393	3.40426313	0.00066343	0.03751554
ENSG00000165474	GJB2	296.008182	2.92441623	0.72628482	4.02654185	5.66E-05	0.00949125
ENSG00000134339	SAA2	58.3180886	2.91972901	0.77995262	3.7434697	0.0001815	0.01841351
ENSG00000131771	PPP1R1B	12311.9485	2.91823457	0.88261808	3.30633901	0.00094524	0.04512661
ENSG00000081051	AFP	18.991715	2.90846044	0.89552271	3.24777966	0.00116309	0.04955725
ENSG00000109255	NMU	82.1627778	2.89024716	0.70947139	4.0738037	4.63E-05	0.00858465
ENSG00000107984	DKK1	87.9172468	2.88325301	0.78422717	3.6765533	0.00023641	0.02170445
ENSG00000204644	ZFP57	157.704599	2.87665804	0.88581607	3.24746652	0.00116437	0.04955725
ENSG00000118849	RARRES1	1791.80966	2.87163249	0.715173	4.01529771	5.94E-05	0.00975216
ENSG00000172927	MYEOV	26.162615	2.83533661	0.73704636	3.84689047	0.00011963	0.01422081
ENSG00000136155	SCEL	46.2412999	2.82414967	0.86034808	3.28256636	0.00102867	0.04627573
ENSG00000204983	PRSS1	91.2448458	2.82257661	0.76241645	3.70214547	0.00021378	0.02081463
ENSG00000165188	RNF183	10.3955941	2.81989442	0.86758185	3.25029208	0.00115287	0.0495308
ENSG00000165325	DEUP1	31.5150391	2.81103015	0.68331315	4.11382414	3.89E-05	0.00768003
ENSG00000162444	RBP7	243.579347	2.76544048	0.81096383	3.41006635	0.00064947	0.03707357
ENSG00000163739	CXCL1	72.3553018	2.76537384	0.6997232	3.95209682	7.75E-05	0.01118293
ENSG00000113389	NPR3	124.061497	2.76260877	0.74433899	3.71149276	0.00020604	0.02037771
ENSG00000140254	DUOXA1	73.1793133	2.73042968	0.81993602	3.33005213	0.0008683	0.04287007
ENSG00000180438	TPRXL	28.2066008	2.71866619	0.70850417	3.83719152	0.00012445	0.01455347
ENSG00000185038	MROH2A	12.1161443	2.6710443	0.74422963	3.5890056	0.00033194	0.02602294
ENSG00000147689	FAM83A	59.1553877	2.59729452	0.71504724	3.63233976	0.00028086	0.02358098
ENSG00000272620	AFAP1-AS1	38.2244252	2.54299007	0.67007183	3.79510071	0.00014758	0.01605204
ENSG00000165490	DDIAS	136.354478	2.48849063	0.50548901	4.92293712	8.53E-07	0.00058816
ENSG00000158125	XDH	865.676647	2.48767848	0.69892545	3.55929013	0.00037186	0.02788477
ENSG00000158089	GALNT14	170.645865	2.47613732	0.55001259	4.50196478	6.73E-06	0.00250109
ENSG00000171124	FUT3	1134.70205	2.46971153	0.67952759	3.63445366	0.00027857	0.02358098
ENSG00000140511	HAPLN3	709.147509	2.46838797	0.72959878	3.38321284	0.00071643	0.03911101
ENSG00000163347	CLDN1	173.049131	2.45450861	0.69527777	3.53025614	0.00041516	0.02897859
ENSG00000173262	SLC2A14	57.8915702	2.43738493	0.69058794	3.52943454	0.00041645	0.02897859
ENSG00000124882	EREG	19.6980444	2.38892427	0.57568379	4.14971608	3.33E-05	0.00730719
ENSG00000078081	LAMP3	103.759715	2.36605891	0.57167577	4.13881265	3.49E-05	0.00734742
ENSG00000144481	TRPM8	21.8729949	2.36129261	0.71847265	3.28654487	0.00101425	0.04618174
ENSG00000125144	MT1G	130.217549	2.28102804	0.59599721	3.82724618	0.00012958	0.01462129
ENSG00000062282	DGAT2	447.693084	2.27024063	0.60853981	3.73063615	0.000191	0.01929628
ENSG00000275896	PRSS2	37.3730142	2.25882327	0.67680454	3.33748245	0.00084541	0.04252457
ENSG00000227471	AKR1B15	82.4024496	2.25171223	0.66050468	3.4090784	0.00065183	0.03712034
ENSG00000140519	RHCG	56.4358398	2.24058115	0.60383178	3.71060491	0.00020676	0.02037771
ENSG00000086696	HSD17B2	52.123944	2.21271467	0.59034913	3.74814587	0.00017815	0.0181651
ENSG00000181577	C6orf223	49.8051169	2.20948447	0.56009068	3.94486923	7.98E-05	0.0112632
ENSG00000116014	KISS1R	30.7252422	2.19234831	0.67329472	3.25614959	0.00112934	0.04878193
ENSG00000169245	CXCL10	505.909827	2.16353409	0.5977777	3.61929543	0.00029541	0.02417925
ENSG00000141655	TNFRSF11A	912.697312	2.16071317	0.59428695	3.63580789	0.00027711	0.02356027
ENSG00000160111	CPAMD8	482.026837	2.15206257	0.56829863	3.78685158	0.00015257	0.01630049
ENSG00000168306	ACOX2	458.697853	2.10773563	0.62598206	3.36708631	0.00075967	0.04005015
ENSG00000135069	PSAT1	1440.72038	2.09717296	0.56459862	3.71444933	0.00020365	0.02023561
ENSG00000177359	OVOS2	80.6907991	2.08700411	0.46305621	4.50702111	6.57E-06	0.0024804
ENSG00000180346	TIGD2	140.861953	2.07498425	0.47198845	4.39626067	1.10E-05	0.00336613
ENSG00000088726	TMEM40	175.163669	2.05792119	0.62861231	3.27375263	0.00106129	0.04707956
ENSG00000181458	TMEM45A	3496.05034	2.03776431	0.60396537	3.37397539	0.00074091	0.03958882
ENSG00000111846	GCNT2	1209.87589	2.03348777	0.56745107	3.58354731	0.00033896	0.02648709
ENSG00000162782	TDRD5	863.99491	2.0329691	0.51549692	3.94370755	8.02E-05	0.0112632
ENSG00000180938	ZNF572	155.550061	1.98924788	0.59661473	3.33422519	0.00085537	0.0427616
ENSG00000119535	CSF3R	1511.16185	1.98534687	0.54766012	3.62514414	0.0002888	0.02379991
ENSG00000002933	TMEM176A	217.491378	1.98447762	0.56902888	3.48748139	0.00048759	0.03131228
ENSG00000139714	MORN3	81.4804661	1.9611724	0.57054392	3.43737325	0.00058739	0.03459271
ENSG00000046604	DSG2	14005.44	1.9439067	0.51048324	3.80797362	0.00014011	0.01560717
ENSG00000136425	CIB2	112.153615	1.91715688	0.51078437	3.75335859	0.00017448	0.01800435
ENSG00000105173	CCNE1	212.305541	1.90694381	0.4524692	4.21452734	2.50E-05	0.00597827
ENSG00000139618	BRCA2	2094.20745	1.88106276	0.45081229	4.17260752	3.01E-05	0.00685959
ENSG00000024526	DEPDC1	532.458452	1.85271674	0.38333259	4.83318344	1.34E-06	0.00077248
ENSG00000164304	CAGE1	13.9299418	1.85190409	0.52903817	3.50051126	0.00046437	0.03080384
ENSG00000008838	MED24	4263.81059	1.84675145	0.45952145	4.01885794	5.85E-05	0.00967178
ENSG00000137561	TTPA	26.4532515	1.82999498	0.54721611	3.34419061	0.00082523	0.04204472
ENSG00000066279	ASPM	8728.0599	1.81455653	0.45261471	4.00905339	6.10E-05	0.00987922
ENSG00000167419	LPO	34.5008164	1.79148775	0.54629661	3.2793316	0.00104053	0.04661356
ENSG00000169288	MRPL1	487.391581	1.78367043	0.34555327	5.16178138	2.45E-07	0.0002461
ENSG00000176597	B3GNT5	80.1130322	1.74346133	0.43219656	4.03395464	5.48E-05	0.0093731
ENSG00000110660	SLC35F2	171.361516	1.72533346	0.37372628	4.6165698	3.90E-06	0.00174448
ENSG00000112742	TTK	561.014168	1.72245024	0.39740025	4.33429584	1.46E-05	0.00410559
ENSG00000161958	FGF11	61.7453085	1.67760563	0.39831123	4.21179593	2.53E-05	0.00597827
ENSG00000156136	DCK	269.131427	1.67354662	0.38943148	4.29740964	1.73E-05	0.00474155
ENSG00000091436	MAP3K20	1396.15734	1.66085046	0.50617027	3.281209	0.00103363	0.04639045
ENSG00000141664	ZCCHC2	2922.137	1.65099264	0.48934079	3.37391173	0.00074108	0.03958882
ENSG00000154760	SLFN13	651.011004	1.62673624	0.4882763	3.33158956	0.00086352	0.04287007
ENSG00000101670	LIPG	123.346246	1.61013638	0.49305254	3.26564873	0.00109214	0.04777305
ENSG00000123473	STIL	1677.44784	1.60493916	0.27066359	5.92964558	3.04E-09	8.14E-06
ENSG00000254535	PABPC4L	34.9193162	1.60118584	0.48944118	3.27145712	0.00106995	0.04723029
ENSG00000142731	PLK4	470.636198	1.58063671	0.39194772	4.03277436	5.51E-05	0.0093731
ENSG00000120675	DNAJC15	610.031561	1.55665883	0.41079296	3.78939993	0.00015101	0.0162059
ENSG00000154839	SKA1	53.0619101	1.54751266	0.44143204	3.50566458	0.00045547	0.03071124
ENSG00000151470	C4orf33	92.6888008	1.54710816	0.42096558	3.6751417	0.00023772	0.02174214
ENSG00000163017	ACTG2	436.897259	1.52437865	0.46886922	3.25118092	0.00114927	0.0495308
ENSG00000164251	F2RL1	48.9552423	1.51864548	0.4309591	3.52387376	0.00042529	0.02950855
ENSG00000111424	VDR	439.419491	1.5093693	0.40272014	3.74793595	0.0001783	0.0181651
ENSG00000153879	CEBPG	131.506105	1.47745337	0.40426559	3.65466021	0.00025752	0.02263749
ENSG00000170312	CDK1	915.823601	1.4630562	0.36828012	3.97267224	7.11E-05	0.01078525
ENSG00000253352	TUG1	26.7128408	1.45938105	0.37081345	3.93562057	8.30E-05	0.0113104
ENSG00000108691	CCL2	198.680741	1.45833512	0.41519076	3.51244595	0.000444	0.03011483
ENSG00000163738	MTHFD2L	296.954649	1.45646378	0.42420313	3.43341123	0.00059604	0.03487232
ENSG00000070669	ASNS	2061.22223	1.45276429	0.41568764	3.49484599	0.00047434	0.03101955
ENSG00000198814	GK	2469.47336	1.41567635	0.42734911	3.31269288	0.00092402	0.04464649
ENSG00000183765	CHEK2	1016.84288	1.41279256	0.27886878	5.06615531	4.06E-07	0.00032672
ENSG00000173207	CKS1B	361.447371	1.37241746	0.30569561	4.4894902	7.14E-06	0.00257295
ENSG00000137492	THAP12	367.196971	1.36657903	0.33238425	4.11144344	3.93E-05	0.00768003
ENSG00000109674	NEIL3	205.803522	1.36636261	0.35528437	3.84582804	0.00012015	0.01422081
ENSG00000162607	USP1	1031.88978	1.34475388	0.24601239	5.46620382	4.60E-08	6.53E-05
ENSG00000138778	CENPE	1670.76156	1.34418814	0.38596861	3.4826359	0.0004965	0.03171284
ENSG00000166451	CENPN	351.413312	1.3295376	0.36606467	3.63197466	0.00028126	0.02358098
ENSG00000164163	ABCE1	2116.70795	1.32091415	0.29383804	4.49538173	6.94E-06	0.00254064
ENSG00000077152	UBE2T	855.244089	1.31792278	0.35070731	3.75789935	0.00017135	0.01783325
ENSG00000163535	SGO2	283.713457	1.31728398	0.33194429	3.96838878	7.24E-05	0.01078525
ENSG00000072274	TFRC	6033.89923	1.30907761	0.39214339	3.33826259	0.00084304	0.04252457
ENSG00000138750	NUP54	1021.31104	1.29497547	0.26769252	4.83754814	1.31E-06	0.00077248
ENSG00000132646	PCNA	1135.50651	1.28049974	0.34681058	3.6922165	0.00022231	0.02115141
ENSG00000165512	ZNF22	136.27937	1.27761506	0.39077501	3.26943902	0.00107761	0.04739519
ENSG00000164109	MAD2L1	446.050832	1.27670884	0.37224031	3.42979739	0.00060403	0.03522937
ENSG00000104951	IL4I1	159.194103	1.27521849	0.35144082	3.62854401	0.00028502	0.02365612
ENSG00000111247	RAD51AP1	205.355708	1.26903456	0.34062256	3.7256327	0.00019483	0.01959154
ENSG00000174527	MYO1H	27.01367	1.2659882	0.36529701	3.46564071	0.00052897	0.03241752
ENSG00000166803	PCLAF	542.142914	1.25235513	0.37881454	3.30598486	0.00094643	0.04512661
ENSG00000133119	RFC3	485.38866	1.23851982	0.3483517	3.55537182	0.00037744	0.02798987
ENSG00000011426	ANLN	1904.63748	1.2311434	0.31396744	3.92124545	8.81E-05	0.01156022
ENSG00000145337	PYURF	536.605498	1.22091347	0.33269318	3.66978805	0.00024275	0.02185859
ENSG00000100028	SNRPD3	1057.51392	1.21836672	0.26848141	4.53799281	5.68E-06	0.00224558
ENSG00000169607	CKAP2L	408.379739	1.21054205	0.33062545	3.66136988	0.00025087	0.02232458
ENSG00000134690	CDCA8	567.232378	1.19779324	0.36415337	3.28925483	0.00100453	0.04611291
ENSG00000079739	PGM1	2500.0865	1.19715186	0.27576482	4.34120586	1.42E-05	0.00407328
ENSG00000101574	METTL4	277.89051	1.19663767	0.33647233	3.55642227	0.00037594	0.02798987
ENSG00000124795	DEK	2240.09327	1.18722772	0.3339546	3.55505722	0.0003779	0.02798987
ENSG00000146918	NCAPG2	1681.63978	1.18504842	0.33465475	3.54110746	0.00039845	0.02871942
ENSG00000167635	ZNF146	859.407428	1.17761914	0.33363121	3.52970318	0.00041603	0.02897859
ENSG00000197061	H4C3	301.482816	1.17724114	0.32958037	3.57193955	0.00035435	0.02699923
ENSG00000169679	BUB1	1038.57255	1.17523331	0.34231263	3.43321636	0.00059647	0.03487232
ENSG00000074695	LMAN1	9045.78144	1.17030225	0.31675855	3.69461927	0.00022022	0.02110059
ENSG00000176105	YES1	3652.73808	1.16611407	0.3566149	3.26995329	0.00107565	0.04739519
ENSG00000123595	RAB9A	215.164109	1.16263929	0.2479842	4.68836027	2.75E-06	0.00132998
ENSG00000116761	CTH	241.424987	1.1540156	0.31301259	3.68680251	0.00022709	0.02126194
ENSG00000198743	SLC5A3	415.228374	1.14579265	0.33014237	3.47060168	0.00051929	0.03231666
ENSG00000165389	SPTSSA	390.418955	1.14304342	0.35246158	3.24302983	0.00118266	0.04974994
ENSG00000183340	JRKL	301.72931	1.1415208	0.33192696	3.43907224	0.00058371	0.03454485
ENSG00000184588	PDE4B	1131.50332	1.12466413	0.34641126	3.24661537	0.00116786	0.04955725
ENSG00000139514	SLC7A1	998.906554	1.12205073	0.26373589	4.25444828	2.10E-05	0.00544103
ENSG00000167088	SNRPD1	1571.71022	1.12151022	0.27706115	4.04787973	5.17E-05	0.00910916
ENSG00000105185	PDCD5	1195.98523	1.12132796	0.3399762	3.29825431	0.00097288	0.04512661
ENSG00000058804	NDC1	901.430481	1.10977394	0.28754901	3.85942538	0.00011365	0.01386004
ENSG00000153147	SMARCA5	5378.24648	1.09784415	0.27053653	4.05802559	4.95E-05	0.00891768
ENSG00000150787	PTS	776.375469	1.09391354	0.33789696	3.23741757	0.00120617	0.05056272
ENSG00000181544	FANCB	335.020792	1.08317899	0.32874724	3.29486869	0.00098468	0.04537406
ENSG00000171612	SLC25A33	234.665524	1.07970287	0.27435989	3.93535252	8.31E-05	0.0113104
ENSG00000120539	MASTL	2063.23837	1.07263312	0.27295083	3.92976687	8.50E-05	0.01140607
ENSG00000273820	USP27X	41.2735023	1.06286774	0.28999681	3.66510147	0.00024724	0.02211063
ENSG00000072401	UBE2D1	374.709807	1.06051319	0.28532031	3.71692152	0.00020167	0.02012151
ENSG00000170035	UBE2E3	725.239698	1.06041237	0.27498462	3.85626061	0.00011513	0.01390022
ENSG00000076053	RBM7	254.096899	1.05690843	0.28782602	3.67203919	0.00024062	0.02176591
ENSG00000132466	ANKRD17	28363.6888	1.05421566	0.30037629	3.50965004	0.0004487	0.03034799
ENSG00000148572	NRBF2	185.702125	1.05281459	0.29813773	3.53130279	0.00041352	0.02897859
ENSG00000159063	ALG8	2334.30732	1.05155052	0.30779619	3.41638577	0.00063458	0.03656954
ENSG00000170191	NANP	50.9924956	1.0492246	0.29315324	3.57909941	0.00034478	0.0266829
ENSG00000077514	POLD3	534.272417	1.04651742	0.28587546	3.66074589	0.00025148	0.02232458
ENSG00000128309	MPST	296.348157	1.04123289	0.29452985	3.53523719	0.00040741	0.02897859
ENSG00000196352	CD55	1068.27196	1.04053291	0.28265111	3.68133315	0.00023202	0.02146474
ENSG00000176731	RBIS	599.601364	1.03986528	0.31497672	3.30140359	0.00096202	0.04512661
ENSG00000163291	PAQR3	265.502543	1.03906994	0.28765216	3.6122445	0.00030356	0.02467918
ENSG00000119787	ATL2	1371.42242	1.03749235	0.24176729	4.2912851	1.78E-05	0.00481949
ENSG00000133703	KRAS	609.489082	1.03277183	0.29098575	3.54921797	0.00038638	0.02835703
ENSG00000171806	METTL18	59.9097313	1.03110826	0.28870791	3.57145823	0.000355	0.02699923
ENSG00000138768	USO1	3649.56048	1.03064332	0.30698144	3.35734735	0.00078694	0.041218
ENSG00000164024	METAP1	1125.78594	1.03016487	0.29179431	3.53044875	0.00041486	0.02897859
ENSG00000143157	POGK	680.214868	1.02202056	0.26199245	3.90095423	9.58E-05	0.01218855
ENSG00000106615	RHEB	1833.11888	1.02194656	0.25998325	3.93081693	8.47E-05	0.01140607
ENSG00000135845	PIGC	167.798582	1.01586423	0.27902668	3.64074223	0.00027185	0.02319494
ENSG00000134758	RNF138	787.657136	1.00438287	0.29963894	3.35197717	0.00080237	0.04126303
ENSG00000132963	POMP	3583.68385	0.99533218	0.24072727	4.13468805	3.55E-05	0.00737923
ENSG00000143155	TIPRL	856.738584	0.98288187	0.19323745	5.08639438	3.65E-07	0.0003147
ENSG00000146858	ZC3HAV1L	254.955405	0.98244549	0.29134724	3.37207752	0.00074603	0.03973611
ENSG00000085415	SEH1L	1345.43165	0.98052142	0.28261356	3.46947761	0.00052147	0.03236877
ENSG00000111711	GOLT1B	394.398684	0.97601314	0.29025416	3.36261548	0.00077208	0.04052739
ENSG00000126261	UBA2	2867.39196	0.97587325	0.30060888	3.2463221	0.00116906	0.04955725
ENSG00000138385	SSB	1757.97184	0.97569431	0.28707114	3.39878926	0.00067685	0.03774407
ENSG00000197965	MPZL1	1125.19897	0.97342255	0.26332121	3.69671155	0.00021841	0.02109494
ENSG00000120334	CENPL	135.609991	0.9719289	0.25379272	3.82961698	0.00012834	0.01455347
ENSG00000101558	VAPA	2323.02993	0.96755305	0.25478012	3.79760021	0.0001461	0.01596299
ENSG00000162368	CMPK1	1338.30059	0.96672041	0.27852388	3.47087081	0.00051877	0.03231666
ENSG00000127922	SEM1	1560.62348	0.96461026	0.29685478	3.24943484	0.00115635	0.0495308
ENSG00000095951	HIVEP1	1338.2741	0.96378897	0.26851218	3.58936774	0.00033148	0.02602294
ENSG00000152253	SPC25	186.518595	0.9545319	0.28933065	3.29910398	0.00096994	0.04512661
ENSG00000213625	LEPROT	171.898174	0.95390984	0.24473956	3.89765279	9.71E-05	0.012279
ENSG00000122958	VPS26A	1023.70846	0.95194917	0.27617989	3.44684457	0.00056717	0.0340318
ENSG00000186312	CA5BP1	222.876841	0.94994392	0.20596055	4.6122616	3.98E-06	0.00174866
ENSG00000152256	PDK1	1011.45389	0.94777923	0.28685981	3.3039805	0.00095322	0.04512661
ENSG00000164164	OTUD4	1762.88237	0.93663954	0.23026133	4.06772406	4.75E-05	0.00868425
ENSG00000120686	UFM1	259.307329	0.92889637	0.27713194	3.35181996	0.00080282	0.04126303
ENSG00000168813	ZNF507	591.302067	0.92519352	0.27603742	3.35169597	0.00080318	0.04126303
ENSG00000111845	PAK1IP1	862.15221	0.92143811	0.2835814	3.2492897	0.00115694	0.0495308
ENSG00000116750	UCHL5	1035.89557	0.92138701	0.23418287	3.93447649	8.34E-05	0.0113104
ENSG00000066557	LRRC40	458.003232	0.92133541	0.20765939	4.4367625	9.13E-06	0.00295686
ENSG00000136824	SMC2	1386.89269	0.9116139	0.27418361	3.32482999	0.00088472	0.04324405
ENSG00000101236	RNF24	184.731404	0.91011171	0.26751178	3.40213694	0.00066861	0.0375612
ENSG00000081870	HSPB11	445.037714	0.90871594	0.24661668	3.68473027	0.00022894	0.02126194
ENSG00000167695	TLCD3A	129.37577	0.90823289	0.27982066	3.24576776	0.00117134	0.04955725
ENSG00000000460	C1orf112	1014.00143	0.9074504	0.23512358	3.8594614	0.00011364	0.01386004
ENSG00000189308	LIN54	1545.48259	0.90503215	0.26282468	3.44348236	0.00057427	0.03423808
ENSG00000151690	MFSD6	2627.05625	0.90169698	0.27771564	3.24683541	0.00116696	0.04955725
ENSG00000103121	CMC2	293.798236	0.89174616	0.23615218	3.77615038	0.00015927	0.01679369
ENSG00000126067	PSMB2	1689.97277	0.87667436	0.25730224	3.40717738	0.00065638	0.03720437
ENSG00000061676	NCKAP1	8579.1766	0.87512408	0.20279658	4.31528032	1.59E-05	0.00442401
ENSG00000177888	ZBTB41	1710.59378	0.87455795	0.23999017	3.6441407	0.00026829	0.02312255
ENSG00000137500	CCDC90B	355.694344	0.86619888	0.2429778	3.56493019	0.00036395	0.02746253
ENSG00000154582	ELOC	896.628861	0.85847775	0.25504752	3.36595213	0.0007628	0.04012757
ENSG00000151151	IPMK	227.140217	0.85301017	0.23342229	3.65436475	0.00025782	0.02263749
ENSG00000115514	TXNDC9	335.987314	0.8467146	0.2347956	3.60617747	0.00031074	0.02492739
ENSG00000101654	RNMT	955.945883	0.83782331	0.23343923	3.5890425	0.00033189	0.02602294
ENSG00000117862	TXNDC12	589.402234	0.83471465	0.17822296	4.68354169	2.82E-06	0.00133494
ENSG00000110172	CHORDC1	1069.57336	0.8310315	0.21385871	3.88589044	0.00010196	0.01275553
ENSG00000198498	TMA16	297.594955	0.83085954	0.22547931	3.68485941	0.00022883	0.02126194
ENSG00000134851	TMEM165	1025.14335	0.82743228	0.24754083	3.34260933	0.00082995	0.04210061
ENSG00000196591	HDAC2	2641.7622	0.8235402	0.19796422	4.16004569	3.18E-05	0.00707756
ENSG00000090487	SPG21	3118.11365	0.81864974	0.24940343	3.28243178	0.00102916	0.04627573
ENSG00000197498	RPF2	680.761177	0.81373338	0.22966485	3.54313415	0.0003954	0.02867077
ENSG00000153989	NUS1	1139.79917	0.81066398	0.19555458	4.14546158	3.39E-05	0.00731128
ENSG00000042429	MED17	1388.29246	0.81047415	0.23054034	3.51554161	0.00043886	0.02998857
ENSG00000153310	FAM49B	2089.45269	0.80835857	0.23955017	3.37448547	0.00073954	0.03958882
ENSG00000116212	LRRC42	679.679143	0.80512057	0.23357332	3.44697143	0.00056691	0.0340318
ENSG00000150768	DLAT	3552.5917	0.7988956	0.22697442	3.51976046	0.00043194	0.02981073
ENSG00000198331	HYLS1	405.970459	0.79416078	0.24148878	3.28860316	0.00100686	0.04613209
ENSG00000008018	PSMB1	2482.29668	0.78393844	0.23913576	3.27821505	0.00104466	0.04668686
ENSG00000055130	CUL1	1976.9318	0.77907267	0.19597033	3.9754623	7.02E-05	0.01073468
ENSG00000155660	PDIA4	5017.5018	0.7779628	0.21804649	3.56787578	0.00035989	0.02724085
ENSG00000104343	UBE2W	279.047254	0.77551399	0.22371265	3.46656293	0.00052716	0.03241705
ENSG00000004897	CDC27	1530.84837	0.77286359	0.21991905	3.5143095	0.0004409	0.02998857
ENSG00000187676	B3GLCT	332.732958	0.77087098	0.23044318	3.34516724	0.00082233	0.04204472
ENSG00000144320	LNPK	766.38016	0.76856365	0.21194313	3.62627302	0.00028754	0.02377731
ENSG00000117593	DARS2	2042.1142	0.76603924	0.21876085	3.50171994	0.00046227	0.03080384
ENSG00000104231	ZFAND1	376.13712	0.76520395	0.21213716	3.6071189	0.00030962	0.02491994
ENSG00000166266	CUL5	1206.23432	0.76119256	0.18749391	4.05982562	4.91E-05	0.00891575
ENSG00000171792	RHNO1	157.321955	0.75424713	0.22857598	3.29976543	0.00096766	0.04512661
ENSG00000173744	AGFG1	2919.0066	0.72364459	0.15397747	4.69967849	2.61E-06	0.00131078
ENSG00000149308	NPAT	2881.32676	0.7028046	0.15890285	4.42285724	9.74E-06	0.00309463
ENSG00000106028	SSBP1	932.178096	0.6831989	0.20847461	3.27713242	0.00104867	0.04671813
ENSG00000033178	UBA6	3329.30214	0.68181731	0.20177304	3.37912985	0.00072716	0.03932218
ENSG00000088205	DDX18	1453.97913	0.67523406	0.19922971	3.38922382	0.00070091	0.03863954
ENSG00000079332	SAR1A	1140.03222	0.67248791	0.2030809	3.31142865	0.00092821	0.04464649
ENSG00000128534	LSM8	481.707624	0.66987374	0.15044487	4.45261937	8.48E-06	0.00285382
ENSG00000166889	PATL1	1716.66589	0.66928578	0.20546147	3.25747578	0.00112408	0.04867199
ENSG00000116030	SUMO1	642.076774	0.66571026	0.2027694	3.28309028	0.00102676	0.04627573
ENSG00000148835	TAF5	139.677825	0.66431981	0.2038302	3.2591825	0.00111734	0.0485238
ENSG00000080345	RIF1	5535.29665	0.6599658	0.19487484	3.38661368	0.00070761	0.03883168
ENSG00000211460	TSN	993.953439	0.61994019	0.14095654	4.39809454	1.09E-05	0.00336613
ENSG00000155561	NUP205	5251.59568	0.60782566	0.17908049	3.39414788	0.00068843	0.03803824
ENSG00000142864	SERBP1	3430.70278	0.5714682	0.17490474	3.26731112	0.00108574	0.04766609
ENSG00000117523	PRRC2C	24822.0578	0.57049744	0.15439548	3.69503989	0.00021985	0.02110059
ENSG00000086848	ALG9	1176.07137	0.5446345	0.16499835	3.30084828	0.00096393	0.04512661
ENSG00000143337	TOR1AIP1	991.426457	0.54143467	0.15484323	3.49666343	0.00047112	0.03099608
ENSG00000025796	SEC63	7900.76367	0.51632369	0.15760791	3.27600116	0.00105288	0.04681928
ENSG00000177917	ARL6IP6	333.545607	0.48550148	0.13472333	3.60369272	0.00031373	0.02508369

Common DEG in S100A9^{high} patients and radioresistant surrogates. Gene expression profile of human brain metastasis from breast cancer were classified in to high and low expression of

S100A9. DESeq2 was used to identify differentially expressed genes between two groups of samples based on a negative binomial distribution model. Values in the table represent normalized base mean expression level, Log2 fold change, shrinkage of effect size (lfcSE), Wald statistic, Wald test p-value and adjusted p-value below a given FDR cutoff, alpha = 0.05. Those upregulated were evaluated among in vitro and ex vivo surrogates of radio-resistance. In these preparations Cuffdiff (Cufflinks 2.2.1) was used to identify differentially expressed genes between two groups of samples based on the beta negative binomial distribution. The estimated significance level (P value) was corrected to account for multiple hypotheses testing using a Benjamin and Hochberg False Discovery Rate (FDR) adjustment.

Supplementary Table 12.

Supplementary Table 12_Identification of specific NF-kB targets in radioresistant conditions_Corresponds to Fig. 4R

Specific components of the signature hallmark_TNFA_signaling_via_NFKB identified among significantly (P-adjust < 0.05) upregulated genes in both in vitro and ex vivo radio-resistant conditions

**GENE SYMBOL**
CEBPB
DUSP5
FOS
HES1
JUNB
NR4A2
SAT1
SNN
TNC

Identification of specific NF-κB targets in radioresistant conditions. Individual genes of the NF-κB hallmark signature significantly upregulated in both *in vitro* and *ex vivo* radio-resistant conditions are shown.

Supplementary Table 13.

Supplementary Table 13_Cohort of 66 human brain metastases stained for S100A9, RAGE and JunB_Corresponds to Fig. 2J, Fig. S4J

										IHC score		
Samples	ID	H12O/HLP ID	Cohort	Primary tumor	Gender	Age at brain metastasis diagnosis	Date diagnosis brain metastasis	Date surgery brain metastasis	KPS	S100A9	RAGE	JunB
#1	hBrM#41	17-37637	Hospital 12 de Octubre	Lung	Man	68yo	01.12.2017	04.12.2017	60	0	1	2
#2	hBrM#44	18-08235	Hospital 12 de Octubre	Lung	Man	74yo	02.03.2018	09.03.2018	80	1	1	3
#3	hBrM#45	18-8430	Hospital 12 de Octubre	Lung	Man	67yo	03.03.2018	12.03.2018	80	2	1	3
#4	hBrM#52	19-03189	Hospital 12 de Octubre	Lung	Man	62yo	10.01.2019	31.02.2019	80	0	0	3
#5	hBrM#53	B19-10204	Hospital 12 de Octubre	Breast	Woman	60yo	04.03.2019	28.03.2019	80	3	2	3
#6	hBrM#54	B19-11122A	Hospital 12 de Octubre	Breast	Woman	38yo	12.11.2018	04.04.2019	70	1	1	1
#7	hBrM#55	19-14588	Hospital 12 de Octubre	Lung	Man	59yo	28.04.2019	XX.05.20219	70		1	1
#8	hBrM#56	B19-15287	Hospital 12 de Octubre	Lung	Man	42yo	1st diagn: XX.12.2018; Relapse: XX.05.2019	17.05.2019	70	2	2	3
#9	hBrM#57	B-1920190	Hospital 12 de Octubre	Breast	Woman	46yo	17.06.2019	26.06.2019	70	1	0	2
#10	hBrM#58	B1922871	Hospital 12 de Octubre	Breast	Woman	82yo	24.06.2019	19.07.2019	50	2	2	2
#11	hBrM#59	B19-24715	Hospital 12 de Octubre	Lung	Woman	56yo	05.08.2019	12.08.2019	90	0	0	3
#12	hBrM#60	B19-30578	Hospital 12 de Octubre	Colorectal	Woman	48yo	01.07.2019	14.10.2019	70		0	3
#13	hBrM#62	20-2342	Hospital 12 de Octubre	Lung	Man	58yo	01.05.2019	24.01.2020	60	2	2	1
#14	hBrM#63	B-20-4586	Hospital 12 de Octubre	Lung	Man	50yo	11.02.2020	12.02.2020	70	0	1	1
#15	hBrM#67	B20-15783	Hospital 12 de Octubre	Lung	Man	59yo	04.12.2018	24.07.2020	80	1	2	3
#16	hBrM#68	20-16380	Hospital 12 de Octubre	Lung	Man	67yo	25.06.2020	03.08.2020	90	0	2	2
#17	hBrM#69	20-16600	Hospital 12 de Octubre	Lung	Man	56yo	06.02.2020	05.08.2020	80	1	1	2
#18	hBrM#72	B-20-20390-B	Hospital 12 de Octubre	Uterine	Woman	31yo	18.09.2020	01.10.2020	70	0	1	
#19	hBrM#74	B20-223558	Hospital 12 de Octubre	Rectal	Man	79yo	01.08.2020	25.10.2020	60	2	2	2
#20	hBrM#75	B-20-23341	Hospital 12 de Octubre	Lung	Woman	47yo	01.11.2020	05.11.2020	70	0	2	3
#21	hBrM#76	B-20-25228	Hospital 12 de Octubre	Breast now	Woman	30yo	14.11.2020	26.11.2020	80	0		
#22	hBrM#77	CIPA:1406751186/ 375444	Hospital de La Princesa	Lung	Woman	67yo	18.11.2020	27.11.2020	80	0	2	3
#23	hBrM#78.1	20B26116	Hospital 12 de Octubre	Lung	Woman	45yo	27.11.2020	04.12.2020	70	2	2	3
#24	hBrM#79	B-20-26624-D	Hospital 12 de Octubre	Breast	Woman	39yo	12.11.2018	16.12.2020	80	1	2	
#25	hBrM#80	1312627743	Hospital de La Princesa	Breast	Woman	46yo	10.12.2020	21.12.2020	60	3	2	3
#26	hBrM#87	1423781154	Hospital de La Princesa	Lung	Woman	58yo	06.02.2021	18.02.2021	80			1
#27	hBrM#89	B-21-5970	Hospital 12 de Octubre	Breast	Woman		XX.07.2019	11.03.2021				3
#28	hBrM#90	CIPA:1526261856	Hospital de La Princesa	Lung	Woman	58yo	02.08.2019	17.03.2021				3
#29	6		Toulouse	Lung	Woman	68yo		23/09/2011	60	2	2	2
#30	12		Toulouse	Lung	Man	81yo		01/09/2012	90	2	2	1
#31	13		Toulouse	Lung	Man	69yo		24/07/2018	90	0	0	1
#32	18		Toulouse	Lung	Man	46yo		14/09/2011	80	0	1	2
#33	20		Toulouse	Lung	Woman	45yo		25/05/2012	100	0	1	1
#34	23		Toulouse	Lung	Woman	67yo		17/04/2012	80	2	1	2
#35	28		Toulouse	Lung	Man	54yo		24/02/2015	80	3	2	2
#36	35		Toulouse	Lung	Man	63yo		07/06/2012	40	1	1	3
#37	36		Toulouse	Lung	Man	83yo		06/10/2016	90	0	2	2
#38	37		Toulouse	Lung	Man	47yo		20/09/2017	100	0	2	2
#39	39		Toulouse	Lung	Woman	59yo		31/03/2016	100	0	0	2
#40	41		Toulouse	Lung	Woman	57yo		15/10/2014	80	0	1	3
#41	42		Toulouse	Lung	Man	63yo		08/06/2011	80	0		1
#42	43		Toulouse	Lung	Man	60yo		10/09/2018	70	0	0	2
#43	57		Toulouse	Lung	Woman	50yo		24/10/2014	100	0	2	1
#44	58		Toulouse	Lung	Man	47yo		03/05/2012	90	0	2	3
#45	2017/I/2850		Turin	Breast	Woman	50yo	01/01/2017	13/04/2017	100	0	1	1
#46	912/2019/I		Turin	Breast	Woman	43yo	01/12/2018	14/01/2019	80	0	0	1
#47	16063/2018/I		Turin	Breast	Woman	59yo	01/07/2018	13/07/2018	100	0	0	1
#48	2014/I3/4630		Turin	Breast	Woman	68yo	01/01/2013	23/01/2013	90	0	0	3
#49	2016/I/12744		Turin	Breast	Woman	63yo	03/06/2016	03/06/2016	70	0	0	1
#50	2017/I/181		Turin	Breast	Woman	43yo	04/01/2017	04/01/2017	80	0	0	1
#51	2015/I/14860		Turin	Breast	Woman	40yo	08/07/2015	08/07/2015	100	0	2	1
#52	1161/2018/I		Turin	Breast	Woman	61yo	01/12/2017	16/01/2018	100	1	1	3
#53	11320/2018/SI		Turin	Breast	Woman	54yo	01/10/2018	23/10/2018	70	1	1	1
#54	103935/2012/RI		Turin	Breast	Woman	53yo	01/11/2010	12/04/2012	100	2	0	1
#55	9805/2019/I		Turin	Breast	Woman	64yo	01/03/2019	16/04/2019	90	2	2	1
#56	2016/I/22640		Turin	Breast	Woman	50yo	16/11/2015	18/10/2016	80	2	2	1
#57	2016/I/15698		Turin	Breast	Woman	41yo	24/05/2016	08/07/2016	100	3	2	3
#58	110735/2011/RI		Turin	Breast	Woman	74yo	07/05/2010	24/10/2011	100	3	1	1
#59	442/I/15		Turin	Breast	Woman	47yo	01/03/2007	09/01/2015	100	3	2	1
#60	11319/2018/SI		Turin	Breast	Woman	46yo	01/10/2018	23/10/2018	100	3	0	1
#61	2014/I3/853		Turin	Breast	Woman	57yo	27/01/2014	27/01/2014	100	3	2	1
#62	12455/2017/I		Turin	Breast	Woman	39yo	01/05/2017	30/05/2017	70	3	2	1
#63	2014/I3/4631		Turin	Breast	Woman	57yo	27/04/2017	27/04/2017	80	3		
#64	106240/2015/RI		Turin	Breast	Woman	45yo	01/05/2015	09/06/2015	90	3	0	1
#65	103047/2011/RI		Turin	Breast	Woman	58yo	01/02/2011	23/03/2011	80	3	2	1
#66	11869/2019/I		Turin	Breast	Woman	46yo	01/05/2019	24/05/2019	80	3	2	1

Cohort of 66 human brain metastases stained for S100A9, RAGE and JunB. 26 human brain metastases stained with indicated antibodies and their respective score.

Supplementary Table 14.

Supplementary Table 14_Cohort of 140 human brain metastases to determine the expression of S100A9 in different brain metastases of different primary origin_Corresponds to Fig.5B

Sample	ID	Cohort	Retrospective study	Primary tumor	Gender	S100A9 Score
#1	244	Manchester	S100A9 expression by primary origin	Breast	female	0
#2	490	Manchester	S100A9 expression by primary origin	Breast	female	0
#3	560	Manchester	S100A9 expression by primary origin	Breast	female	1
#4	707	Manchester	S100A9 expression by primary origin	Breast	female	0
#5	712	Manchester	S100A9 expression by primary origin	Breast	female	0
#6	722	Manchester	S100A9 expression by primary origin	Breast	female	0
#7	725	Manchester	S100A9 expression by primary origin	Breast	female	0
#8	788	Manchester	S100A9 expression by primary origin	Breast	female	0
#9	943	Manchester	S100A9 expression by primary origin	Breast	female	0
#10	972	Manchester	S100A9 expression by primary origin	Breast	female	0
#11	1040	Manchester	S100A9 expression by primary origin	Breast	female	2
#12	1148	Manchester	S100A9 expression by primary origin	Breast	female	0
#13	1158	Manchester	S100A9 expression by primary origin	Breast	female	0
#14	1170	Manchester	S100A9 expression by primary origin	Breast	female	3
#15	1709	Manchester	S100A9 expression by primary origin	Breast	female	0
#16	2302	Manchester	S100A9 expression by primary origin	Breast	female	2
#17	2401	Manchester	S100A9 expression by primary origin	Breast	female	0
#18	2586	Manchester	S100A9 expression by primary origin	Breast	female	0
#19	2597	Manchester	S100A9 expression by primary origin	Breast	female	0
#20	2925	Manchester	S100A9 expression by primary origin	Breast	female	2
#21	B19-10204	Hospital 12 de Octubre	S100A9 expression by primary origin	Breast	female	3
#22	B19-11122A	Hospital 12 de Octubre	S100A9 expression by primary origin	Breast	female	1
#23	B-1920190	Hospital 12 de Octubre	S100A9 expression by primary origin	Breast	female	1
#24	B1922871	Hospital 12 de Octubre	S100A9 expression by primary origin	Breast	female	2
#25	B-20-25228	Hospital 12 de Octubre	S100A9 expression by primary origin	Breast	female	0
#26	B-20-26624-D	Hospital 12 de Octubre	S100A9 expression by primary origin	Breast	female	1
#27	1312627743	Hospital de La Princesa	S100A9 expression by primary origin	Breast	female	3
#28	2017/I/2850	Turin	S100A9 expression by primary origin	Breast	female	0
#29	912/2019/I	Turin	S100A9 expression by primary origin	Breast	female	0
#30	16063/2018/I	Turin	S100A9 expression by primary origin	Breast	female	0
#31	2014/I3/4630	Turin	S100A9 expression by primary origin	Breast	female	0
#32	2016/I/12744	Turin	S100A9 expression by primary origin	Breast	female	0
#33	2017/I/181	Turin	S100A9 expression by primary origin	Breast	female	0
#34	2015/I/14860	Turin	S100A9 expression by primary origin	Breast	female	0
#35	1161/2018/I	Turin	S100A9 expression by primary origin	Breast	female	1
#36	11320/2018/SI	Turin	S100A9 expression by primary origin	Breast	female	1
#37	103935/2012/RI	Turin	S100A9 expression by primary origin	Breast	female	2
#38	9805/2019/I	Turin	S100A9 expression by primary origin	Breast	female	2
#39	2016/I/22640	Turin	S100A9 expression by primary origin	Breast	female	2
#40	2016/I/15698	Turin	S100A9 expression by primary origin	Breast	female	3
#41	110735/2011/RI	Turin	S100A9 expression by primary origin	Breast	female	3
#42	442/I/15	Turin	S100A9 expression by primary origin	Breast	female	3
#43	11319/2018/SI	Turin	S100A9 expression by primary origin	Breast	female	3
#44	2014/I3/853	Turin	S100A9 expression by primary origin	Breast	female	3
#45	12455/2017/I	Turin	S100A9 expression by primary origin	Breast	female	3
#46	2014/I3/4631	Turin	S100A9 expression by primary origin	Breast	female	3
#47	106240/2015/RI	Turin	S100A9 expression by primary origin	Breast	female	3
#48	103047/2011/RI	Turin	S100A9 expression by primary origin	Breast	female	3
#49	11869/2019/I	Turin	S100A9 expression by primary origin	Breast	female	3
#1	78	Manchester	S100A9 expression by primary origin	lung	female	3
#2	215	Manchester	S100A9 expression by primary origin	lung	male	0
#3	221	Manchester	S100A9 expression by primary origin	lung	male	0
#4	517	Manchester	S100A9 expression by primary origin	lung	female	3
#5	541	Manchester	S100A9 expression by primary origin	lung	male	0
#6	666	Manchester	S100A9 expression by primary origin	lung	female	0
#7	684	Manchester	S100A9 expression by primary origin	lung	female	0
#8	847	Manchester	S100A9 expression by primary origin	lung	female	0
#9	865	Manchester	S100A9 expression by primary origin	lung	female	0
#10	866	Manchester	S100A9 expression by primary origin	lung	male	0
#11	940	Manchester	S100A9 expression by primary origin	lung	male	3
#12	1011	Manchester	S100A9 expression by primary origin	lung	female	0
#13	1031	Manchester	S100A9 expression by primary origin	lung	male	3
#14	1060	Manchester	S100A9 expression by primary origin	lung	male	0
#15	1311	Manchester	S100A9 expression by primary origin	lung	female	3
#16	1348	Manchester	S100A9 expression by primary origin	lung	female	0
#17	1438	Manchester	S100A9 expression by primary origin	lung	female	0
#18	1601	Manchester	S100A9 expression by primary origin	lung	female	3
#19	1757	Manchester	S100A9 expression by primary origin	lung	male	0
#20	17-37637	Hospital 12 de Octubre	S100A9 expression by primary origin	lung	male	0
#21	18-08235	Hospital 12 de Octubre	S100A9 expression by primary origin	lung	male	1
#22	18-8430	Hospital 12 de Octubre	S100A9 expression by primary origin	lung	male	2
#23	19-03189	Hospital 12 de Octubre	S100A9 expression by primary origin	lung	male	0
#24	B19-15287	Hospital 12 de Octubre	S100A9 expression by primary origin	lung	male	2
#25	B19-24715	Hospital 12 de Octubre	S100A9 expression by primary origin	lung	female	0
#26	20-2342	Hospital 12 de Octubre	S100A9 expression by primary origin	lung	male	2
#27	B-20-4586	Hospital 12 de Octubre	S100A9 expression by primary origin	lung	male	0
#28	B20-15783	Hospital 12 de Octubre	S100A9 expression by primary origin	lung	male	1
#29	20-16380	Hospital 12 de Octubre	S100A9 expression by primary origin	lung	male	0
#30	20-16600	Hospital 12 de Octubre	S100A9 expression by primary origin	lung	male	1
#31	B-20-23341	Hospital 12 de Octubre	S100A9 expression by primary origin	lung	female	0
#32	CIPA:1406751186/ 375444	Hospital de La Princesa	S100A9 expression by primary origin	lung	female	0
#33	20B26116	Hospital 12 de Octubre	S100A9 expression by primary origin	lung	female	2
#34	6	Toulouse	S100A9 expression by primary origin	lung	female	2
#35	12	Toulouse	S100A9 expression by primary origin	lung	male	2
#36	13	Toulouse	S100A9 expression by primary origin	lung	male	0
#37	18	Toulouse	S100A9 expression by primary origin	lung	male	0
#38	20	Toulouse	S100A9 expression by primary origin	lung	female	0
#39	23	Toulouse	S100A9 expression by primary origin	lung	female	2
#40	28	Toulouse	S100A9 expression by primary origin	lung	male	3
#41	35	Toulouse	S100A9 expression by primary origin	lung	male	1
#42	36	Toulouse	S100A9 expression by primary origin	lung	male	0
#43	37	Toulouse	S100A9 expression by primary origin	lung	male	0
#44	39	Toulouse	S100A9 expression by primary origin	lung	female	0
#45	41	Toulouse	S100A9 expression by primary origin	lung	female	0
#46	42	Toulouse	S100A9 expression by primary origin	lung	male	0
#47	43	Toulouse	S100A9 expression by primary origin	lung	male	0
#48	57	Toulouse	S100A9 expression by primary origin	lung	female	0
#49	58	Toulouse	S100A9 expression by primary origin	lung	male	0
#50	87	Toulouse	S100A9 expression by primary origin	lung	female	2
#51	88	Toulouse	S100A9 expression by primary origin	lung	female	0
#52	90	Toulouse	S100A9 expression by primary origin	lung	male	2
#53	91	Toulouse	S100A9 expression by primary origin	lung	male	2
#1	1431	Manchester	S100A9 expression by primary origin	Melanoma	male	0
#2	1465	Manchester	S100A9 expression by primary origin	Melanoma	male	0
#3	1700	Manchester	S100A9 expression by primary origin	Melanoma	male	1
#4	1708	Manchester	S100A9 expression by primary origin	Melanoma	male	0
#5	2216	Manchester	S100A9 expression by primary origin	Melanoma	Female	0
#6	2388	Manchester	S100A9 expression by primary origin	Melanoma	male	1
#7	2392	Manchester	S100A9 expression by primary origin	Melanoma	male	3
#8	2574	Manchester	S100A9 expression by primary origin	Melanoma	male	2
#9	2588	Manchester	S100A9 expression by primary origin	Melanoma	Female	0
#10	2635	Manchester	S100A9 expression by primary origin	Melanoma	male	0
#11	2768	Manchester	S100A9 expression by primary origin	Melanoma	Female	0
#12	2772	Manchester	S100A9 expression by primary origin	Melanoma	male	0
#13	3045	Manchester	S100A9 expression by primary origin	Melanoma	Female	3
#14	3142	Manchester	S100A9 expression by primary origin	Melanoma	male	0
#15	H001068T1PTa	Manchester	S100A9 expression by primary origin	Melanoma	Male	0
#16	H001604T1PTa	Manchester	S100A9 expression by primary origin	Melanoma	Female	0
#17	H001664T1PTa	Manchester	S100A9 expression by primary origin	Melanoma	Female	3
#18	H001669T1PTa 28/01/2015	Manchester	S100A9 expression by primary origin	Melanoma	Male	0
#19	H001682T1PTa	Manchester	S100A9 expression by primary origin	Melanoma	Male	0
#20	H001686T1PTa 11/02/2015	Manchester	S100A9 expression by primary origin	Melanoma	Male	0
#21	H001835T1PTa 14/01/2016	Manchester	S100A9 expression by primary origin	Melanoma	Female	0
#22	H001839T1PTa 22/01/2016	Manchester	S100A9 expression by primary origin	Melanoma	Male	0
#23	H001845T1PTa	Manchester	S100A9 expression by primary origin	Melanoma	Female	3
#24	H001936T1PTa 22/06/2016	Manchester	S100A9 expression by primary origin	Melanoma	Male	0
#25	H003034T1PTa	Manchester	S100A9 expression by primary origin	Melanoma	Male	0
#26	H003275T1PTa	Manchester	S100A9 expression by primary origin	Melanoma	Female	0
#27	H000576T1PTa	Manchester	S100A9 expression by primary origin	Melanoma	Male	2
#28	H001576T1PTa	Manchester	S100A9 expression by primary origin	Melanoma	Male	0
#29	H003084T1PTa	Manchester	S100A9 expression by primary origin	Melanoma	Male	2
#30	H003166T1PTa	Manchester	S100A9 expression by primary origin	Melanoma	Male	0
#31	H003220T1PTa	Manchester	S100A9 expression by primary origin	Melanoma	Male	0
#32	H003229T1PTa	Manchester	S100A9 expression by primary origin	Melanoma	Male	0
#33	H003274T1PTa	Manchester	S100A9 expression by primary origin	Melanoma	Male	0
#34	H003306T1PTa	Manchester	S100A9 expression by primary origin	Melanoma	Female	0
#35	H003309T1PTa	Manchester	S100A9 expression by primary origin	Melanoma	Male	0
#36	H003332T1PTa	Manchester	S100A9 expression by primary origin	Melanoma	Female	0
#37	H003407T1PTa	Manchester	S100A9 expression by primary origin	Melanoma	Male	1
#38	H003428T1PTa	Manchester	S100A9 expression by primary origin	Melanoma	Male	1

Cohort of 140 human brain metastases to determine the expression of S100A9 in different

brain metastases of different primary origin. The levels of S100A9 were analyzed respect to the primary tumor source of the brain metastasis.

Supplementary Table 15.

Supplementary Table 15_Cohort of 22 lung cancer human brain metastases to determine the prognostic value of S100A9 to radiotherapy_Corresponds to Fig.5D

ID	Sample	Cohort	Retrospective study	Primary tumor	Gender	Age at neurosurgery	KPS	S100A9 IHC	Date neurosurgery	Date of local relapse	Date of last follow-up	Time to local relapse from neurosurgery/WBRT (days)
6	#1	Toulouse	Relapse after adjuvant radiotherapy	Lung	female	68	60	high	23/09/2011	29/11/2011		67
12	#2	Toulouse	Relapse after adjuvant radiotherapy	Lung	male	81	90	high	01/09/2012	14/02/2013		166
13	#3	Toulouse	Relapse after adjuvant radiotherapy	Lung	male	69	90	low	24/07/2018	no relapse	26/08/2020	764
18	#4	Toulouse	Relapse after adjuvant radiotherapy	Lung	male	46	80	low	14/09/2011	no relapse	22/07/2012	312
20	#5	Toulouse	Relapse after adjuvant radiotherapy	Lung	female	45	100	low	25/05/2012	24/01/2013		244
23	#6	Toulouse	Relapse after adjuvant radiotherapy	Lung	female	67	80	high	17/04/2012	no relapse	09/11/2012	206
28	#7	Toulouse	Relapse after adjuvant radiotherapy	Lung	male	54	80	high	24/02/2015	16/07/2015		142
33	#8	Toulouse	Relapse after adjuvant radiotherapy	Lung	male	63	100	low	07/06/2012	27/12/2013		568
35	#9	Toulouse	Relapse after adjuvant radiotherapy	Lung	male	83	40	high	06/10/2016	no relapse	08/01/2018	459
36	#10	Toulouse	Relapse after adjuvant radiotherapy	Lung	male	47	90	high	20/09/2017	04/01/2019		471
37	#11	Toulouse	Relapse after adjuvant radiotherapy	Lung	female	59	100	low	31/03/2016	13/06/2017		439
39	#12	Toulouse	Relapse after adjuvant radiotherapy	Lung	female	57	100	low	15/10/2014	25/05/2016		588
41	#13	Toulouse	Relapse after adjuvant radiotherapy	Lung	male	63	80	low	08/06/2011	no relapse	04/11/2013	880
42	#14	Toulouse	Relapse after adjuvant radiotherapy	Lung	male	53	80	low	29/10/2012	no relapse	16/08/2021	3213
43	#15	Toulouse	Relapse after adjuvant radiotherapy	Lung	male	60	70	low	10/09/2018	no relapse	11/06/2019	274
57	#16	Toulouse	Relapse after adjuvant radiotherapy	Lung	female	50	100	low	24/10/2014	no relapse	08/04/2016	532
58	#17	Toulouse	Relapse after adjuvant radiotherapy	Lung	male	47	90	low	03/05/2012	13/02/2013		286
66	#18	Toulouse	Relapse after adjuvant radiotherapy	Lung	male	53	100	low	02/03/2016	no relapse	13/07/2021	1959
87	#19	Toulouse	Relapse after adjuvant radiotherapy	Lung	female	50	60	low	27/08/2019	no relapse	06/07/2021	679
88	#20	Toulouse	Relapse after adjuvant radiotherapy	Lung	female	52	80	high	30/04/2021	no relapse	17/08/2021	109
90	#21	Toulouse	Relapse after adjuvant radiotherapy	Lung	male	69	60	high	01/02/2020	no relapse	07/06/2020	127
91	#22	Toulouse	Relapse after adjuvant radiotherapy	Lung	male	70	70	high	03/12/2019	no relapse	12/06/2020	192

Cohort of 22 lung cancer human brain metastases to determine the prognostic value of S100A9 to radiotherapy. The levels of S100A9 immunohistochemistry (low <5% positive cancer cells) were evaluated with the time to local relapse to determine their correlation to the response to WBRT.

Supplementary Table 16.

Supplementary Table 16_Cohort of 42 breast cancer human brain metastases to determine the prognostic value of S100A9 to radiotherapy_Corresponds to Fig.5E and Fig. S5B-C

Sample	ID	Cohort	Retrospective study	Primary tumor	Gender	Age at brain met diagnosis	S100A9 mRNA (log2 (TMM-CPM+1)) in brain metastasis	S100A8 mRNA (log2 (TMM-CPM+1)) in brain metastasis	Cut--Off group (S100A9)	OS from brain met diagnosis (months)
#1	17_Pitt	Vareslija, Priedigkeit *et al*, JNCI 2019	Relapse after adjuvant radiotherapy II	Breast	female	37	0.06	0.04	low	30
#2	MAYO_3	GSE173661	Relapse after adjuvant radiotherapy II	Breast	female	26	0.18	0.49	low	29
#3	6_RCS	Vareslija, Priedigkeit *et al*, JNCI 2019	Relapse after adjuvant radiotherapy II	Breast	female	49	0.19	0.00	low	21
#4	MAYO_2	GSE173661	Relapse after adjuvant radiotherapy II	Breast	female	40	0.24	0.63	low	33
#5	MAYO_20	GSE173661	Relapse after adjuvant radiotherapy II	Breast	female	58	0.28	0.78	low	20
#6	47_Pitt	Vareslija, Priedigkeit *et al*, JNCI 2019	Relapse after adjuvant radiotherapy II	Breast	female	66	0.32	0.27	low	74
#7	MAYO_1	GSE173661	Relapse after adjuvant radiotherapy II	Breast	female	62	0.45	1.35	low	7
#8	MAYO_23	GSE173661	Relapse after adjuvant radiotherapy II	Breast	female	62	0.66	0.10	low	9
#9	4_RCS	Vareslija, Priedigkeit *et al*, JNCI 2019	Relapse after adjuvant radiotherapy II	Breast	female	59	0.66	0.00	low	24
#10	MAYO_13	GSE173661	Relapse after adjuvant radiotherapy II	Breast	female	29	0.68	0.80	low	10
#11	2_RCS	Vareslija, Priedigkeit *et al*, JNCI 2019	Relapse after adjuvant radiotherapy II	Breast	female	64	0.72	0.25	low	48
#12	3_RCS	Vareslija, Priedigkeit *et al*, JNCI 2019	Relapse after adjuvant radiotherapy II	Breast	female	64	0.80	0.72	low	67
#13	MAYO_5	GSE173661	Relapse after adjuvant radiotherapy II	Breast	female	46	0.80	0.68	low	13
#14	68_Pitt	Vareslija, Priedigkeit *et al*, JNCI 2019	Relapse after adjuvant radiotherapy II	Breast	female	53	1.05	0.43	low	114
#15	72_Pitt	Vareslija, Priedigkeit *et al*, JNCI 2019	Relapse after adjuvant radiotherapy II	Breast	female	58	1.17	0.39	low	5
#16	MAYO_10	GSE173661	Relapse after adjuvant radiotherapy II	Breast	female	44	1.22	0.74	low	4
#17	51_Pitt	Vareslija, Priedigkeit *et al*, JNCI 2019	Relapse after adjuvant radiotherapy II	Breast	female	65	1.37	0.62	low	9
#18	MAYO_6	GSE173661	Relapse after adjuvant radiotherapy II	Breast	female	47	1.61	0.88	low	21
#19	71_Pitt	Vareslija, Priedigkeit *et al*, JNCI 2019	Relapse after adjuvant radiotherapy II	Breast	female	28	1.66	1.68	low	147
#20	MAYO_15	GSE173661	Relapse after adjuvant radiotherapy II	Breast	female	53	1.95	2.40	low	5
#21	MAYO_22	GSE173661	Relapse after adjuvant radiotherapy II	Breast	female	37	2.07	2.61	low	59
#22	1_RCS	Vareslija, Priedigkeit *et al*, JNCI 2019	Relapse after adjuvant radiotherapy II	Breast	female	51	2.72	1.75	low	11
#23	7_Pitt	Vareslija, Priedigkeit *et al*, JNCI 2019	Relapse after adjuvant radiotherapy II	Breast	female	40	2.93	3.08	low	13
#24	MAYO_19	GSE173661	Relapse after adjuvant radiotherapy II	Breast	female	50	3.18	3.87	low	65
#25	64_Pitt	Vareslija, Priedigkeit *et al*, JNCI 2019	Relapse after adjuvant radiotherapy II	Breast	female	46	3.46	2.72	high	5
#26	5_RCS	Vareslija, Priedigkeit *et al*, JNCI 2019	Relapse after adjuvant radiotherapy II	Breast	female	40	3.77	2.88	high	17
#27	MAYO_14	GSE173661	Relapse after adjuvant radiotherapy II	Breast	female	40	4.12	2.90	high	35
#28	MAYO_18	GSE173661	Relapse after adjuvant radiotherapy II	Breast	female	53	4.13	2.29	high	4
#29	MAYO_16	GSE173661	Relapse after adjuvant radiotherapy II	Breast	female	44	4.13	0.54	high	49
#30	MAYO_21	GSE173661	Relapse after adjuvant radiotherapy II	Breast	female	51	4.73	3.94	high	18
#31	MAYO_7	GSE173661	Relapse after adjuvant radiotherapy II	Breast	female	40	4.76	3.94	high	7
#32	MAYO_24	GSE173661	Relapse after adjuvant radiotherapy II	Breast	female	35	5.18	5.58	high	3
#33	12_Pitt	Vareslija, Priedigkeit *et al*, JNCI 2019	Relapse after adjuvant radiotherapy II	Breast	female	41	5.27	5.26	high	14
#34	52_Pitt	Vareslija, Priedigkeit *et al*, JNCI 2019	Relapse after adjuvant radiotherapy II	Breast	female	67	5.30	4.30	high	7
#35	MAYO_11	GSE173661	Relapse after adjuvant radiotherapy II	Breast	female	67	5.54	5.43	high	26
#36	MAYO_9	GSE173661	Relapse after adjuvant radiotherapy II	Breast	female	45	5.73	5.33	high	13
#37	6_Pitt	Vareslija, Priedigkeit *et al*, JNCI 2019	Relapse after adjuvant radiotherapy II	Breast	female	68	5.95	6.38	high	12
#38	MAYO_17	GSE173661	Relapse after adjuvant radiotherapy II	Breast	female	40	6.13	4.57	high	18
#39	MAYO_4	GSE173661	Relapse after adjuvant radiotherapy II	Breast	female	55	6.80	7.53	high	16
#40	MAYO_8	GSE173661	Relapse after adjuvant radiotherapy II	Breast	female	57	7.35	8.16	high	32
#41	62_Pitt	Vareslija, Priedigkeit *et al*, JNCI 2019	Relapse after adjuvant radiotherapy II	Breast	female	67	7.77	8.30	high	6
#42	192_Pitt	Vareslija, Priedigkeit *et al*, JNCI 2019	Relapse after adjuvant radiotherapy II	Breast	female	58	7.78	7.27	high	16

Cohort of 42 breast cancer human brain metastases to determine the prognostic value of S100A9 to radiotherapy. *S100A9* expression in brain metastasis was collected from a published cohort and evaluated with overall survival (OS) from the diagnosis of the brain metastasis.

Supplementary Table 17.

Supplementary Table 17_Cohort of 34 human brain metastases to determine the prognostic value of S100A9 to radiotherapy_Corresponds to Fig.5F

Sample	ID	Cohort	Retrospective study	Primary tumor	Gender	Age at time of neurosurgery	KPS	S100A9 IHC	OS from brain met diagnosis (days)	OS from brain met diagnosis (months)
#1	2574	Manchester/Preston	Relapse after adjuvant radiotherapy III	melanoma	male	60s		high	500	17
#2	3045	Manchester/Preston	Relapse after adjuvant radiotherapy III	melanoma	female	40s		high	178	6
#3	1170	Manchester/Preston	Relapse after adjuvant radiotherapy III	breast	female	60s		high	196	7
#4	2302	Manchester/Preston	Relapse after adjuvant radiotherapy III	breast	female	70s		high	77	3
#5	1700	Manchester/Preston	Relapse after adjuvant radiotherapy III	melanoma	male	50s		high	115	4
#6	2014/I3/4630	Turin	Relapse after adjuvant radiotherapy III	breast	female	68	90	high	793	26
#7	2016/I/12744	Turin	Relapse after adjuvant radiotherapy III	breast	female	63	70	high	248	8
#8	2017/I/181	Turin	Relapse after adjuvant radiotherapy III	breast	female	43	80	high	294	10
#9	2014/I3/853	Turin	Relapse after adjuvant radiotherapy III	breast	female	57	100	high	383	13
#10	11320/2018/SI	Turin	Relapse after adjuvant radiotherapy III	breast	female	54	70	high	191	6
#11	103935/2012/RI	Turin	Relapse after adjuvant radiotherapy III	breast	female	53	100	high	873	29
#12	9805/2019/I	Turin	Relapse after adjuvant radiotherapy III	breast	female	64	90	high	315	11
#13	12455/2017/I	Turin	Relapse after adjuvant radiotherapy III	breast	female	39	70	high	161	5
#14	2014/I3/4631	Turin	Relapse after adjuvant radiotherapy III	breast	female	57	80	high	94	3
#15	2216	Manchester/Preston	Relapse after adjuvant radiotherapy III	melanoma	female	60s		low	153	5
#16	2401	Manchester/Preston	Relapse after adjuvant radiotherapy III	breast	female	50s		low	1372	46
#17	2586	Manchester/Preston	Relapse after adjuvant radiotherapy III	breast	female	50s		low	993	33
#18	3142	Manchester/Preston	Relapse after adjuvant radiotherapy III	melanoma	male	70s		low	194	6
#19	707	Manchester/Preston	Relapse after adjuvant radiotherapy III	breast	female	60s		low	544	18
#20	943	Manchester/Preston	Relapse after adjuvant radiotherapy III	breast	female	60s		low	759	25
#21	1158	Manchester/Preston	Relapse after adjuvant radiotherapy III	breast	female	50s		low	479	16
#22	H001068	Manchester/Preston	Relapse after adjuvant radiotherapy III	melanoma	male	49	80	low	638	21
#23	H001604	Manchester/Preston	Relapse after adjuvant radiotherapy III	melanoma	female	40		low	623	21
#24	H001682	Manchester/Preston	Relapse after adjuvant radiotherapy III	melanoma	male	80	100	low	291	10
#25	H001835	Manchester/Preston	Relapse after adjuvant radiotherapy III	melanoma	female	45	100	low	358	12
#26	H001839	Manchester/Preston	Relapse after adjuvant radiotherapy III	melanoma	male	64	60	low	566	19
#27	H003275	Manchester/Preston	Relapse after adjuvant radiotherapy III	melanoma	female	37		low	560	19
#28	H003229	Manchester/Preston	Relapse after adjuvant radiotherapy III	melanoma	male	44	100	low	737	25
#29	H003274	Manchester/Preston	Relapse after adjuvant radiotherapy III	melanoma	male	87	100	low	665	22
#30	H003306	Manchester/Preston	Relapse after adjuvant radiotherapy III	melanoma	female	46		low	567	19
#31	2014/I3/4630	Turin	Relapse after adjuvant radiotherapy III	breast	female	68	90	low	793	26
#32	2016/I/12744	Turin	Relapse after adjuvant radiotherapy III	breast	female	63	70	low	248	8
#33	2017/I/181	Turin	Relapse after adjuvant radiotherapy III	breast	female	43	80	low	294	10
#34	2015/I/14860	Turin	Relapse after adjuvant radiotherapy III	breast	female	40	100	low	2297	77

Cohort of 34 human brain metastases from breast cancer and melanoma to determine the prognostic value of S100A9 to radiotherapy. The levels of S100A9 immunohistochemistry (low <5% positive cancer cells) were evaluated with the time to local relapse to determine their correlation to the response to WBRT.

Supplementary Table 18.

Supplementary Table 18_Cohort of 36 lung cancer human brain metastases to determine the prognostic value of S100A9 to SRS_Corresponds to Fig.S5B

ID	Sample	Cohort	Retrospective study	Primary tumor	S100A9 IHC	Date neurosurgery	Date of local relapse	Date of last follow-up	Time to local relapse from neurosurgery/SRS
2	#1	Toulouse	Relapse after adjuvant SRS	Lung	high	21/07/2017		13/11/2019	845
8	#2	Toulouse	Relapse after adjuvant SRS	Lung	high	16/02/2018		05/04/2019	413
10	#3	Toulouse	Relapse after adjuvant SRS	Lung	high	08/09/2014		22/10/2019	1870
11	#4	Toulouse	Relapse after adjuvant SRS	Lung	high	12/07/2018		16/10/2019	461
14	#5	Toulouse	Relapse after adjuvant SRS	Lung	high	21/04/2017		27/12/2017	250
16	#6	Toulouse	Relapse after adjuvant SRS	Lung	high	12/11/2018		20/11/2019	373
22	#7	Toulouse	Relapse after adjuvant SRS	Lung	low	05/07/2016		18/07/2017	378
26	#8	Toulouse	Relapse after adjuvant SRS	Lung	low	09/04/2018		27/11/2019	597
27	#9	Toulouse	Relapse after adjuvant SRS	Lung	low	11/05/2018	04/01/2019		238
29	#10	Toulouse	Relapse after adjuvant SRS	Lung	low	15/11/2018		01/10/2019	320
30	#11	Toulouse	Relapse after adjuvant SRS	Lung	low	10/01/2018		26/11/2019	685
31	#12	Toulouse	Relapse after adjuvant SRS	Lung	low	19/06/2014		14/10/2016	848
34	#13	Toulouse	Relapse after adjuvant SRS	Lung	low	26/08/2015		04/06/2017	648
40	#14	Toulouse	Relapse after adjuvant SRS	Lung	low	15/04/2015		24/02/2017	681
46	#15	Toulouse	Relapse after adjuvant SRS	Lung	low	13/12/2016	24/12/2018		741
47	#16	Toulouse	Relapse after adjuvant SRS	Lung	low	27/06/2014		11/02/2018	1325
48	#17	Toulouse	Relapse after adjuvant SRS	Lung	low	05/09/2018		20/10/2019	410
50	#18	Toulouse	Relapse after adjuvant SRS	Lung	low	01/12/2015		08/03/2018	828
51	#19	Toulouse	Relapse after adjuvant SRS	Lung	low	02/03/2015		24/08/2019	1636
54	#20	Toulouse	Relapse after adjuvant SRS	Lung	low	29/05/2017		05/06/2019	737
55	#21	Toulouse	Relapse after adjuvant SRS	Lung	low	10/08/2015		30/12/2016	508
56	#22	Toulouse	Relapse after adjuvant SRS	Lung	low	06/11/2018		03/12/2019	392
60	#23	Toulouse	Relapse after adjuvant SRS	Lung	low	09/02/2016		15/10/2017	614
61	#24	Toulouse	Relapse after adjuvant SRS	Lung	low	10/08/2017		17/05/2018	280
67	#25	Toulouse	Relapse after adjuvant SRS	Lung	low	01/10/2018		15/11/2019	410
68	#26	Toulouse	Relapse after adjuvant SRS	Lung	low	03/08/2018		18/02/2019	199
70	#27	Toulouse	Relapse after adjuvant SRS	Lung	low	29/09/2011		01/07/2019	2832
71	#28	Toulouse	Relapse after adjuvant SRS	Lung	low	07/06/2018		03/12/2019	544
72	#29	Toulouse	Relapse after adjuvant SRS	Lung	low	28/01/2018		30/08/2019	579
73	#30	Toulouse	Relapse after adjuvant SRS	Lung	low	27/02/2017		29/11/2019	1005
74	#31	Toulouse	Relapse after adjuvant SRS	Lung	low	17/11/2017		20/11/2018	368
75	#32	Toulouse	Relapse after adjuvant SRS	Lung	low	07/12/2015		28/11/2019	1452
76	#33	Toulouse	Relapse after adjuvant SRS	Lung	low	30/11/2016		20/11/2019	1085
77	#34	Toulouse	Relapse after adjuvant SRS	Lung	low	06/11/2018		27/11/2019	386
78	#35	Toulouse	Relapse after adjuvant SRS	Lung	low	11/02/2019		23/05/2019	101
79	#36	Toulouse	Relapse after adjuvant SRS	Lung	low	22/02/2019		30/11/2019	281

Cohort of 36 lung cancer human brain metastases to determine the prognostic value of S100A9 to SRS. The levels of S100A9 immunohistochemistry (low <5% positive cancer cells) were evaluated with the time to local relapse to determine their correlation to the response to SRS.

Supplementary Table 19.

Supplementary Table 19_Cohort of human glioblastomas to estimate the prognostic value of S100A9 to radiotherapy_Corresponds to Fig.S5G-H

Sample ID	Experiment	Cohort	Primary tumor	S100A9 mRNA (log2(normalized counts + 0.5))	Cut-Off group	OS from diagnosis of 1ry tumor
TCGA.02.0001	S100A9 in GBM	TCGA	Glioblastoma	11.753	high	11.8
TCGA.02.0003	S100A9 in GBM	TCGA	Glioblastoma	9.822	high	4.7
TCGA.02.0007	S100A9 in GBM	TCGA	Glioblastoma	11.303	high	23.2
TCGA.02.0027	S100A9 in GBM	TCGA	Glioblastoma	11.004	high	12.2
TCGA.02.0028	S100A9 in GBM	TCGA	Glioblastoma	11.362	high	90.6
TCGA.02.0033	S100A9 in GBM	TCGA	Glioblastoma	11.234	high	2.8
TCGA.02.0034	S100A9 in GBM	TCGA	Glioblastoma	10.204	high	14.1
TCGA.02.0043	S100A9 in GBM	TCGA	Glioblastoma	11.135	high	18.3
TCGA.02.0046	S100A9 in GBM	TCGA	Glioblastoma	10.973	high	6.9
TCGA.02.0051	S100A9 in GBM	TCGA	Glioblastoma	9.049	high	15.1
TCGA.02.0052	S100A9 in GBM	TCGA	Glioblastoma	11.289	high	12.6
TCGA.02.0054	S100A9 in GBM	TCGA	Glioblastoma	10.781	high	6.5
TCGA.02.0059	S100A9 in GBM	TCGA	Glioblastoma	8.941	high	9.6
TCGA.02.0060	S100A9 in GBM	TCGA	Glioblastoma	9.779	high	6
TCGA.02.0064	S100A9 in GBM	TCGA	Glioblastoma	10.670	high	19.7
TCGA.02.0086	S100A9 in GBM	TCGA	Glioblastoma	9.219	high	8.8
TCGA.02.0332	S100A9 in GBM	TCGA	Glioblastoma	9.152	high	25.7
TCGA.02.0446	S100A9 in GBM	TCGA	Glioblastoma	9.804	high	9.3
TCGA.02.0451	S100A9 in GBM	TCGA	Glioblastoma	9.317	high	16.2
TCGA.02.2486	S100A9 in GBM	TCGA	Glioblastoma	9.794	high	20.3
TCGA.06.0122	S100A9 in GBM	TCGA	Glioblastoma	9.195	high	6.2
TCGA.06.0128	S100A9 in GBM	TCGA	Glioblastoma	9.940	high	22.7
TCGA.06.0132	S100A9 in GBM	TCGA	Glioblastoma	9.941	high	25.4
TCGA.06.0138	S100A9 in GBM	TCGA	Glioblastoma	9.585	high	24.2
TCGA.06.0139	S100A9 in GBM	TCGA	Glioblastoma	11.711	high	11.9
TCGA.06.0141	S100A9 in GBM	TCGA	Glioblastoma	10.421	high	10.3
TCGA.06.0143	S100A9 in GBM	TCGA	Glioblastoma	9.229	high	11.7
TCGA.06.0147	S100A9 in GBM	TCGA	Glioblastoma	10.351	high	17.8
TCGA.06.0152	S100A9 in GBM	TCGA	Glioblastoma	9.498	high	12.3
TCGA.06.0154	S100A9 in GBM	TCGA	Glioblastoma	9.526	high	13.9
TCGA.06.0156	S100A9 in GBM	TCGA	Glioblastoma	9.641	high	5.8
TCGA.06.0162	S100A9 in GBM	TCGA	Glioblastoma	10.484	high	3.4
TCGA.06.0168	S100A9 in GBM	TCGA	Glioblastoma	9.584	high	19.7
TCGA.06.0169	S100A9 in GBM	TCGA	Glioblastoma	10.368	high	3.3
TCGA.06.0175	S100A9 in GBM	TCGA	Glioblastoma	9.242	high	4
TCGA.06.0176	S100A9 in GBM	TCGA	Glioblastoma	9.272	high	91
TCGA.06.0179	S100A9 in GBM	TCGA	Glioblastoma	10.343	high	20.2
TCGA.06.0182	S100A9 in GBM	TCGA	Glioblastoma	9.110	high	3.6
TCGA.06.0189	S100A9 in GBM	TCGA	Glioblastoma	9.795	high	15.4
TCGA.06.0190	S100A9 in GBM	TCGA	Glioblastoma	8.960	high	10.4
TCGA.06.0192	S100A9 in GBM	TCGA	Glioblastoma	9.096	high	39
TCGA.06.0194	S100A9 in GBM	TCGA	Glioblastoma	9.834	high	4.7
TCGA.06.0197	S100A9 in GBM	TCGA	Glioblastoma	9.374	high	5.6
TCGA.06.0201	S100A9 in GBM	TCGA	Glioblastoma	11.887	high	0.4
TCGA.06.0210	S100A9 in GBM	TCGA	Glioblastoma	9.659	high	7.4
TCGA.06.0213	S100A9 in GBM	TCGA	Glioblastoma	11.132	high	0.5
TCGA.06.0219	S100A9 in GBM	TCGA	Glioblastoma	9.490	high	0.7
TCGA.06.0221	S100A9 in GBM	TCGA	Glioblastoma	9.108	high	19.8
TCGA.06.0394	S100A9 in GBM	TCGA	Glioblastoma	9.020	high	10.8
TCGA.06.0409	S100A9 in GBM	TCGA	Glioblastoma	10.351	high	72.4
TCGA.06.0412	S100A9 in GBM	TCGA	Glioblastoma	10.537	high	9.6
TCGA.06.0644	S100A9 in GBM	TCGA	Glioblastoma	9.169	high	12.6
TCGA.06.0645	S100A9 in GBM	TCGA	Glioblastoma	10.590	high	5.8
TCGA.06.0646	S100A9 in GBM	TCGA	Glioblastoma	9.650	high	5.8
TCGA.06.0650	S100A9 in GBM	TCGA	Glioblastoma	9.251	high	23.6
TCGA.06.0745	S100A9 in GBM	TCGA	Glioblastoma	9.096	high	7.9
TCGA.06.0750	S100A9 in GBM	TCGA	Glioblastoma	9.018	high	0.9
TCGA.06.0881	S100A9 in GBM	TCGA	Glioblastoma	10.665	high	16.6
TCGA.06.0882	S100A9 in GBM	TCGA	Glioblastoma	8.901	high	20.8
TCGA.06.0939	S100A9 in GBM	TCGA	Glioblastoma	11.433	high	26.8
TCGA.06.1084	S100A9 in GBM	TCGA	Glioblastoma	9.577	high	23.9
TCGA.06.1802	S100A9 in GBM	TCGA	Glioblastoma	9.270	high	15.3
TCGA.06.2562	S100A9 in GBM	TCGA	Glioblastoma	9.440	high	12.6
TCGA.06.2567	S100A9 in GBM	TCGA	Glioblastoma	9.776	high	4.4
TCGA.06.5410	S100A9 in GBM	TCGA	Glioblastoma	11.254	high	3.5
TCGA.06.5411	S100A9 in GBM	TCGA	Glioblastoma	8.991	high	8.3
TCGA.06.5412	S100A9 in GBM	TCGA	Glioblastoma	9.402	high	4.5
TCGA.06.5413	S100A9 in GBM	TCGA	Glioblastoma	9.184	high	8.8
TCGA.06.5414	S100A9 in GBM	TCGA	Glioblastoma	9.419	high	9
TCGA.06.5418	S100A9 in GBM	TCGA	Glioblastoma	9.869	high	2.7
TCGA.06.6390	S100A9 in GBM	TCGA	Glioblastoma	9.524	high	5.4
TCGA.08.0246	S100A9 in GBM	TCGA	Glioblastoma	9.153	high	4.2
TCGA.08.0345	S100A9 in GBM	TCGA	Glioblastoma	9.533	high	1.7
TCGA.08.0354	S100A9 in GBM	TCGA	Glioblastoma	9.340	high	17.9
TCGA.08.0356	S100A9 in GBM	TCGA	Glioblastoma	9.317	high	31.1
TCGA.08.0359	S100A9 in GBM	TCGA	Glioblastoma	9.421	high	3.4
TCGA.08.0360	S100A9 in GBM	TCGA	Glioblastoma	10.008	high	15.4
TCGA.08.0390	S100A9 in GBM	TCGA	Glioblastoma	9.034	high	14
TCGA.08.0392	S100A9 in GBM	TCGA	Glioblastoma	8.909	high	0.7
TCGA.08.0509	S100A9 in GBM	TCGA	Glioblastoma	8.910	high	12.6
TCGA.08.0510	S100A9 in GBM	TCGA	Glioblastoma	9.110	high	4.3
TCGA.08.0520	S100A9 in GBM	TCGA	Glioblastoma	8.890	high	10.8
TCGA.08.0525	S100A9 in GBM	TCGA	Glioblastoma	9.232	high	16
TCGA.12.0619	S100A9 in GBM	TCGA	Glioblastoma	9.384	high	34.9
TCGA.12.0772	S100A9 in GBM	TCGA	Glioblastoma	9.838	high	53.9
TCGA.12.0776	S100A9 in GBM	TCGA	Glioblastoma	11.024	high	9.7
TCGA.12.1094	S100A9 in GBM	TCGA	Glioblastoma	9.011	high	12.2
TCGA.12.1096	S100A9 in GBM	TCGA	Glioblastoma	9.728	high	9.1
TCGA.12.1602	S100A9 in GBM	TCGA	Glioblastoma	10.409	high	6.8
TCGA.12.3644	S100A9 in GBM	TCGA	Glioblastoma	9.196	high	59.8
TCGA.12.5299	S100A9 in GBM	TCGA	Glioblastoma	9.506	high	3.2
TCGA.12.5301	S100A9 in GBM	TCGA	Glioblastoma	9.221	high	2
TCGA.14.0783	S100A9 in GBM	TCGA	Glioblastoma	9.595	high	6.2
TCGA.14.0789	S100A9 in GBM	TCGA	Glioblastoma	9.603	high	11.2
TCGA.14.0812	S100A9 in GBM	TCGA	Glioblastoma	9.326	high	3.2
TCGA.14.0817	S100A9 in GBM	TCGA	Glioblastoma	9.228	high	5.4
TCGA.14.0866	S100A9 in GBM	TCGA	Glioblastoma	9.031	high	26.3
TCGA.14.1037	S100A9 in GBM	TCGA	Glioblastoma	9.165	high	19.3
TCGA.14.1455	S100A9 in GBM	TCGA	Glioblastoma	9.703	high	0.9
TCGA.14.1823	S100A9 in GBM	TCGA	Glioblastoma	9.388	high	17.9
TCGA.14.1829	S100A9 in GBM	TCGA	Glioblastoma	8.933	high	7.2
TCGA.14.2554	S100A9 in GBM	TCGA	Glioblastoma	9.500	high	17.5
TCGA.16.1045	S100A9 in GBM	TCGA	Glioblastoma	9.302	high	29
TCGA.19.0957	S100A9 in GBM	TCGA	Glioblastoma	8.978	high	21.9
TCGA.19.1790	S100A9 in GBM	TCGA	Glioblastoma	9.668	high	5.1
TCGA.19.5947	S100A9 in GBM	TCGA	Glioblastoma	9.159	high	6.6
TCGA.26.5139	S100A9 in GBM	TCGA	Glioblastoma	11.213	high	1.6
TCGA.27.2524	S100A9 in GBM	TCGA	Glioblastoma	9.440	high	7.6
TCGA.28.1751	S100A9 in GBM	TCGA	Glioblastoma	9.027	high	7.6
TCGA.28.1752	S100A9 in GBM	TCGA	Glioblastoma	9.579	high	8.5
TCGA.28.1760	S100A9 in GBM	TCGA	Glioblastoma	9.287	high	4.7
TCGA.28.2513	S100A9 in GBM	TCGA	Glioblastoma	9.627	high	7.3
TCGA.28.5208	S100A9 in GBM	TCGA	Glioblastoma	8.869	high	17.9
TCGA.28.5213	S100A9 in GBM	TCGA	Glioblastoma	10.301	high	31.3
TCGA.28.5214	S100A9 in GBM	TCGA	Glioblastoma	8.915	high	23.4
TCGA.28.5215	S100A9 in GBM	TCGA	Glioblastoma	13.095	high	11
TCGA.28.5216	S100A9 in GBM	TCGA	Glioblastoma	10.809	high	13.6
TCGA.28.5218	S100A9 in GBM	TCGA	Glioblastoma	12.234	high	5.2
TCGA.28.5219	S100A9 in GBM	TCGA	Glioblastoma	9.194	high	22.7
TCGA.32.1979	S100A9 in GBM	TCGA	Glioblastoma	10.283	high	19.5
TCGA.32.1980	S100A9 in GBM	TCGA	Glioblastoma	9.254	high	1.2
TCGA.32.2494	S100A9 in GBM	TCGA	Glioblastoma	9.827	high	20.8
TCGA.41.3392	S100A9 in GBM	TCGA	Glioblastoma	9.474	high	1
TCGA.41.3393	S100A9 in GBM	TCGA	Glioblastoma	10.377	high	4.4
TCGA.41.3915	S100A9 in GBM	TCGA	Glioblastoma	9.824	high	11.8
TCGA.76.4926	S100A9 in GBM	TCGA	Glioblastoma	9.506	high	4.5
TCGA.76.4928	S100A9 in GBM	TCGA	Glioblastoma	9.328	high	3.1
TCGA.76.4929	S100A9 in GBM	TCGA	Glioblastoma	8.856	high	3.6
TCGA.76.6193	S100A9 in GBM	TCGA	Glioblastoma	9.412	high	2.7
TCGA.76.6282	S100A9 in GBM	TCGA	Glioblastoma	9.011	high	17.1
TCGA.02.0011	S100A9 in GBM	TCGA	Glioblastoma	6.297	low	20.7
TCGA.02.0014	S100A9 in GBM	TCGA	Glioblastoma	6.027	low	82.6
TCGA.02.0016	S100A9 in GBM	TCGA	Glioblastoma	6.022	low	87.1
TCGA.02.0023	S100A9 in GBM	TCGA	Glioblastoma	5.854	low	20.1
TCGA.02.0024	S100A9 in GBM	TCGA	Glioblastoma	5.571	low	53.1
TCGA.02.0026	S100A9 in GBM	TCGA	Glioblastoma	6.439	low	24.6
TCGA.02.0047	S100A9 in GBM	TCGA	Glioblastoma	6.013	low	14.7
TCGA.02.0048	S100A9 in GBM	TCGA	Glioblastoma	5.128	low	3.2
TCGA.02.0069	S100A9 in GBM	TCGA	Glioblastoma	5.964	low	28.7
TCGA.02.0070	S100A9 in GBM	TCGA	Glioblastoma	6.277	low	25.1
TCGA.02.0080	S100A9 in GBM	TCGA	Glioblastoma	4.949	low	89.7
TCGA.02.0113	S100A9 in GBM	TCGA	Glioblastoma	5.747	low	92.7
TCGA.02.0114	S100A9 in GBM	TCGA	Glioblastoma	6.031	low	100
TCGA.02.0116	S100A9 in GBM	TCGA	Glioblastoma	5.574	low	49
TCGA.02.0258	S100A9 in GBM	TCGA	Glioblastoma	5.397	low	16.5
TCGA.02.0260	S100A9 in GBM	TCGA	Glioblastoma	5.296	low	16.9
TCGA.02.0269	S100A9 in GBM	TCGA	Glioblastoma	5.399	low	10.8
TCGA.02.0271	S100A9 in GBM	TCGA	Glioblastoma	6.013	low	14.5
TCGA.02.0281	S100A9 in GBM	TCGA	Glioblastoma	6.176	low	4
TCGA.02.0289	S100A9 in GBM	TCGA	Glioblastoma	5.481	low	14.2
TCGA.02.0317	S100A9 in GBM	TCGA	Glioblastoma	6.153	low	12.2
TCGA.02.0325	S100A9 in GBM	TCGA	Glioblastoma	6.477	low	10.6
TCGA.02.0326	S100A9 in GBM	TCGA	Glioblastoma	6.314	low	7.3
TCGA.02.0338	S100A9 in GBM	TCGA	Glioblastoma	5.436	low	10.6
TCGA.02.0339	S100A9 in GBM	TCGA	Glioblastoma	4.821	low	12.4
TCGA.02.0432	S100A9 in GBM	TCGA	Glioblastoma	5.476	low	47.1
TCGA.02.2485	S100A9 in GBM	TCGA	Glioblastoma	5.571	low	15.4
TCGA.06.0129	S100A9 in GBM	TCGA	Glioblastoma	5.995	low	33.7
TCGA.06.0160	S100A9 in GBM	TCGA	Glioblastoma	6.462	low	11.8
TCGA.06.0195	S100A9 in GBM	TCGA	Glioblastoma	5.637	low	7.4
TCGA.06.0237	S100A9 in GBM	TCGA	Glioblastoma	5.927	low	13.6
TCGA.06.0747	S100A9 in GBM	TCGA	Glioblastoma	5.737	low	2.7
TCGA.06.1086	S100A9 in GBM	TCGA	Glioblastoma	5.729	low	6.8
TCGA.06.1087	S100A9 in GBM	TCGA	Glioblastoma	6.238	low	4
TCGA.06.1801	S100A9 in GBM	TCGA	Glioblastoma	5.475	low	11.8
TCGA.06.2565	S100A9 in GBM	TCGA	Glioblastoma	6.413	low	16.6
TCGA.06.2569	S100A9 in GBM	TCGA	Glioblastoma	5.294	low	0.4
TCGA.06.5415	S100A9 in GBM	TCGA	Glioblastoma	6.098	low	8.6
TCGA.06.5416	S100A9 in GBM	TCGA	Glioblastoma	6.064	low	6.7
TCGA.06.5417	S100A9 in GBM	TCGA	Glioblastoma	5.554	low	5.1
TCGA.06.5859	S100A9 in GBM	TCGA	Glioblastoma	5.534	low	4.6
TCGA.06.6391	S100A9 in GBM	TCGA	Glioblastoma	5.381	low	1.5
TCGA.08.0344	S100A9 in GBM	TCGA	Glioblastoma	5.501	low	115.9
TCGA.08.0350	S100A9 in GBM	TCGA	Glioblastoma	6.164	low	29.2
TCGA.08.0351	S100A9 in GBM	TCGA	Glioblastoma	6.200	low	65.3
TCGA.08.0380	S100A9 in GBM	TCGA	Glioblastoma	6.064	low	14.9
TCGA.08.0385	S100A9 in GBM	TCGA	Glioblastoma	4.923	low	2.7
TCGA.08.0517	S100A9 in GBM	TCGA	Glioblastoma	6.008	low	58.7
TCGA.08.0518	S100A9 in GBM	TCGA	Glioblastoma	6.365	low	19.3
TCGA.08.0524	S100A9 in GBM	TCGA	Glioblastoma	5.670	low	7.3
TCGA.12.0618	S100A9 in GBM	TCGA	Glioblastoma	6.451	low	13
TCGA.12.0688	S100A9 in GBM	TCGA	Glioblastoma	6.116	low	26.7
TCGA.12.0818	S100A9 in GBM	TCGA	Glioblastoma	4.685	low	91.8
TCGA.12.0819	S100A9 in GBM	TCGA	Glioblastoma	5.342	low	24.8
TCGA.12.0820	S100A9 in GBM	TCGA	Glioblastoma	5.954	low	18.5
TCGA.12.0826	S100A9 in GBM	TCGA	Glioblastoma	6.333	low	27.8
TCGA.12.1089	S100A9 in GBM	TCGA	Glioblastoma	4.774	low	5.8
TCGA.12.1090	S100A9 in GBM	TCGA	Glioblastoma	5.673	low	7.6
TCGA.12.1091	S100A9 in GBM	TCGA	Glioblastoma	6.430	low	33.2
TCGA.12.1098	S100A9 in GBM	TCGA	Glioblastoma	5.617	low	4
TCGA.12.1597	S100A9 in GBM	TCGA	Glioblastoma	6.096	low	22.2
TCGA.12.3646	S100A9 in GBM	TCGA	Glioblastoma	5.437	low	44
TCGA.12.3648	S100A9 in GBM	TCGA	Glioblastoma	5.503	low	26.9
TCGA.12.3649	S100A9 in GBM	TCGA	Glioblastoma	5.942	low	15.2
TCGA.12.3650	S100A9 in GBM	TCGA	Glioblastoma	6.429	low	10.9
TCGA.12.3653	S100A9 in GBM	TCGA	Glioblastoma	5.928	low	14.5
TCGA.14.0790	S100A9 in GBM	TCGA	Glioblastoma	4.961	low	13.8
TCGA.14.0813	S100A9 in GBM	TCGA	Glioblastoma	5.799	low	1.4
TCGA.14.0871	S100A9 in GBM	TCGA	Glioblastoma	5.315	low	28.9
TCGA.14.1454	S100A9 in GBM	TCGA	Glioblastoma	6.024	low	30.2
TCGA.14.1458	S100A9 in GBM	TCGA	Glioblastoma	5.377	low	6.7
TCGA.14.1821	S100A9 in GBM	TCGA	Glioblastoma	5.321	low	17.8
TCGA.14.1825	S100A9 in GBM	TCGA	Glioblastoma	5.632	low	7.6
TCGA.14.3477	S100A9 in GBM	TCGA	Glioblastoma	5.877	low	3.8
TCGA.14.4157	S100A9 in GBM	TCGA	Glioblastoma	4.687	low	3.4
TCGA.15.1446	S100A9 in GBM	TCGA	Glioblastoma	5.817	low	19.1
TCGA.16.0849	S100A9 in GBM	TCGA	Glioblastoma	5.775	low	26.1
TCGA.16.0850	S100A9 in GBM	TCGA	Glioblastoma	5.282	low	16.4
TCGA.16.1047	S100A9 in GBM	TCGA	Glioblastoma	6.494	low	4.6
TCGA.16.1056	S100A9 in GBM	TCGA	Glioblastoma	6.502	low	14
TCGA.16.1062	S100A9 in GBM	TCGA	Glioblastoma	6.427	low	21.2
TCGA.19.0960	S100A9 in GBM	TCGA	Glioblastoma	5.890	low	8.6
TCGA.19.1386	S100A9 in GBM	TCGA	Glioblastoma	6.453	low	9.1
TCGA.19.1388	S100A9 in GBM	TCGA	Glioblastoma	5.951	low	12.9
TCGA.19.1390	S100A9 in GBM	TCGA	Glioblastoma	6.116	low	25.4
TCGA.19.1786	S100A9 in GBM	TCGA	Glioblastoma	5.861	low	7.2
TCGA.19.1787	S100A9 in GBM	TCGA	Glioblastoma	6.480	low	12.7
TCGA.19.1788	S100A9 in GBM	TCGA	Glioblastoma	5.344	low	3.7
TCGA.19.1789	S100A9 in GBM	TCGA	Glioblastoma	5.475	low	3.2
TCGA.19.1791	S100A9 in GBM	TCGA	Glioblastoma	4.938	low	0.1
TCGA.19.2621	S100A9 in GBM	TCGA	Glioblastoma	5.697	low	1.1
TCGA.19.2624	S100A9 in GBM	TCGA	Glioblastoma	5.182	low	0.2
TCGA.19.2629	S100A9 in GBM	TCGA	Glioblastoma	6.009	low	24.2
TCGA.19.4068	S100A9 in GBM	TCGA	Glioblastoma	5.532	low	4.5
TCGA.19.5950	S100A9 in GBM	TCGA	Glioblastoma	5.371	low	17.2
TCGA.19.5951	S100A9 in GBM	TCGA	Glioblastoma	6.248	low	8
TCGA.19.5956	S100A9 in GBM	TCGA	Glioblastoma	5.283	low	22.5
TCGA.19.5960	S100A9 in GBM	TCGA	Glioblastoma	4.736	low	15
TCGA.26.1442	S100A9 in GBM	TCGA	Glioblastoma	6.048	low	31.3
TCGA.26.1443	S100A9 in GBM	TCGA	Glioblastoma	6.484	low	7.1
TCGA.26.5133	S100A9 in GBM	TCGA	Glioblastoma	5.519	low	14.9
TCGA.26.5136	S100A9 in GBM	TCGA	Glioblastoma	5.301	low	19
TCGA.27.1833	S100A9 in GBM	TCGA	Glioblastoma	6.509	low	24.2
TCGA.27.1834	S100A9 in GBM	TCGA	Glioblastoma	6.117	low	40.5
TCGA.27.1835	S100A9 in GBM	TCGA	Glioblastoma	5.399	low	21.3
TCGA.27.1836	S100A9 in GBM	TCGA	Glioblastoma	6.136	low	30.1
TCGA.27.1837	S100A9 in GBM	TCGA	Glioblastoma	6.120	low	14
TCGA.27.2518	S100A9 in GBM	TCGA	Glioblastoma	6.227	low	24.8
TCGA.27.2521	S100A9 in GBM	TCGA	Glioblastoma	6.117	low	10.4
TCGA.27.2526	S100A9 in GBM	TCGA	Glioblastoma	5.705	low	2.9
TCGA.27.2528	S100A9 in GBM	TCGA	Glioblastoma	6.083	low	15.8
TCGA.28.1746	S100A9 in GBM	TCGA	Glioblastoma	6.230	low	0.2
TCGA.28.1747	S100A9 in GBM	TCGA	Glioblastoma	5.910	low	2.5
TCGA.28.1749	S100A9 in GBM	TCGA	Glioblastoma	4.663	low	9.2
TCGA.28.1756	S100A9 in GBM	TCGA	Glioblastoma	6.189	low	2.8
TCGA.28.2506	S100A9 in GBM	TCGA	Glioblastoma	5.958	low	6.7
TCGA.28.5204	S100A9 in GBM	TCGA	Glioblastoma	5.941	low	14.9
TCGA.28.5220	S100A9 in GBM	TCGA	Glioblastoma	5.483	low	10.5
TCGA.32.1970	S100A9 in GBM	TCGA	Glioblastoma	5.918	low	15.4
TCGA.32.1987	S100A9 in GBM	TCGA	Glioblastoma	6.224	low	14.9
TCGA.32.2495	S100A9 in GBM	TCGA	Glioblastoma	6.368	low	15
TCGA.32.2616	S100A9 in GBM	TCGA	Glioblastoma	6.147	low	7.4
TCGA.32.2634	S100A9 in GBM	TCGA	Glioblastoma	5.919	low	22.8
TCGA.32.4719	S100A9 in GBM	TCGA	Glioblastoma	5.347	low	10.8
TCGA.32.5222	S100A9 in GBM	TCGA	Glioblastoma	6.007	low	19.2
TCGA.41.5651	S100A9 in GBM	TCGA	Glioblastoma	5.963	low	15.1
TCGA.76.4925	S100A9 in GBM	TCGA	Glioblastoma	5.592	low	4.8
TCGA.76.4931	S100A9 in GBM	TCGA	Glioblastoma	6.325	low	9.2
TCGA.76.4932	S100A9 in GBM	TCGA	Glioblastoma	5.884	low	47.9
TCGA.76.4934	S100A9 in GBM	TCGA	Glioblastoma	5.712	low	2.5
TCGA.76.6285	S100A9 in GBM	TCGA	Glioblastoma	5.970	low	8.3
TCGA.87.5896	S100A9 in GBM	TCGA	Glioblastoma	5.199	low	26.3

Sample ID	Experiment	Cohort	Primary tumor	S100A9 mRNA (log2(normalized counts + 0.5))	Cut-Off group	OS from diagnosis of 1ry tumor
CGGA_1001	S100A9 in GBM	CGGA	Glioblastoma	7.199	high	112.7
CGGA_1019	S100A9 in GBM	CGGA	Glioblastoma	6.956	high	7
CGGA_1035	S100A9 in GBM	CGGA	Glioblastoma	8.341	high	18.6
CGGA_1036	S100A9 in GBM	CGGA	Glioblastoma	7.787	high	26.5
CGGA_1045	S100A9 in GBM	CGGA	Glioblastoma	8.391	high	11.4
CGGA_1077	S100A9 in GBM	CGGA	Glioblastoma	9.137	high	19.4
CGGA_1106	S100A9 in GBM	CGGA	Glioblastoma	7.722	high	13.8
CGGA_1114	S100A9 in GBM	CGGA	Glioblastoma	8.097	high	15.1
CGGA_1119	S100A9 in GBM	CGGA	Glioblastoma	7.883	high	13.9
CGGA_1124	S100A9 in GBM	CGGA	Glioblastoma	8.692	high	15.9
CGGA_1134	S100A9 in GBM	CGGA	Glioblastoma	7.289	high	1.9
CGGA_1135	S100A9 in GBM	CGGA	Glioblastoma	9.264	high	40.9
CGGA_1136	S100A9 in GBM	CGGA	Glioblastoma	7.415	high	7.8
CGGA_1171	S100A9 in GBM	CGGA	Glioblastoma	8.499	high	13.5
CGGA_1172	S100A9 in GBM	CGGA	Glioblastoma	7.105	high	90.2
CGGA_1177	S100A9 in GBM	CGGA	Glioblastoma	10.505	high	14.8
CGGA_1208	S100A9 in GBM	CGGA	Glioblastoma	9.248	high	5.3
CGGA_1224	S100A9 in GBM	CGGA	Glioblastoma	7.404	high	8.9
CGGA_1240	S100A9 in GBM	CGGA	Glioblastoma	8.022	high	0.6
CGGA_1270	S100A9 in GBM	CGGA	Glioblastoma	7.363	high	6.2
CGGA_1313	S100A9 in GBM	CGGA	Glioblastoma	7.171	high	12.5
CGGA_1338	S100A9 in GBM	CGGA	Glioblastoma	7.576	high	36.9
CGGA_1342	S100A9 in GBM	CGGA	Glioblastoma	8.843	high	6.1
CGGA_1392	S100A9 in GBM	CGGA	Glioblastoma	9.013	high	15.6
CGGA_1402	S100A9 in GBM	CGGA	Glioblastoma	8.554	high	77.4
CGGA_1415	S100A9 in GBM	CGGA	Glioblastoma	8.790	high	76.9
CGGA_1420	S100A9 in GBM	CGGA	Glioblastoma	7.021	high	12
CGGA_1433	S100A9 in GBM	CGGA	Glioblastoma	7.991	high	13
CGGA_1441	S100A9 in GBM	CGGA	Glioblastoma	8.699	high	61.9
CGGA_1451	S100A9 in GBM	CGGA	Glioblastoma	7.541	high	14.4
CGGA_1452	S100A9 in GBM	CGGA	Glioblastoma	9.588	high	15.4
CGGA_1462	S100A9 in GBM	CGGA	Glioblastoma	7.637	high	5.7
CGGA_1480	S100A9 in GBM	CGGA	Glioblastoma	8.677	high	13.3
CGGA_1492	S100A9 in GBM	CGGA	Glioblastoma	7.124	high	8.8
CGGA_1501	S100A9 in GBM	CGGA	Glioblastoma	7.021	high	7.3
CGGA_1503	S100A9 in GBM	CGGA	Glioblastoma	6.806	high	25.5
CGGA_1507	S100A9 in GBM	CGGA	Glioblastoma	7.842	high	23.5
CGGA_1535	S100A9 in GBM	CGGA	Glioblastoma	8.943	high	7.6
CGGA_1543	S100A9 in GBM	CGGA	Glioblastoma	7.615	high	23.8
CGGA_1546	S100A9 in GBM	CGGA	Glioblastoma	9.505	high	7.3
CGGA_1551	S100A9 in GBM	CGGA	Glioblastoma	10.497	high	11.4
CGGA_1572	S100A9 in GBM	CGGA	Glioblastoma	8.489	high	5.3
CGGA_1586	S100A9 in GBM	CGGA	Glioblastoma	8.220	high	7.6
CGGA_1597	S100A9 in GBM	CGGA	Glioblastoma	7.042	high	5.7
CGGA_1601	S100A9 in GBM	CGGA	Glioblastoma	7.690	high	23.3
CGGA_1603	S100A9 in GBM	CGGA	Glioblastoma	6.994	high	11.7
CGGA_1604	S100A9 in GBM	CGGA	Glioblastoma	7.838	high	24.3
CGGA_1612	S100A9 in GBM	CGGA	Glioblastoma	6.940	high	23.6
CGGA_1613	S100A9 in GBM	CGGA	Glioblastoma	8.468	high	8.2
CGGA_1626	S100A9 in GBM	CGGA	Glioblastoma	7.142	high	22.9
CGGA_1658	S100A9 in GBM	CGGA	Glioblastoma	10.092	high	51.7
CGGA_1663	S100A9 in GBM	CGGA	Glioblastoma	9.131	high	9.5
CGGA_1698	S100A9 in GBM	CGGA	Glioblastoma	7.100	high	12.8
CGGA_1706	S100A9 in GBM	CGGA	Glioblastoma	9.530	high	55.5
CGGA_1744	S100A9 in GBM	CGGA	Glioblastoma	8.855	high	51.2
CGGA_1811	S100A9 in GBM	CGGA	Glioblastoma	8.318	high	10
CGGA_1857	S100A9 in GBM	CGGA	Glioblastoma	9.212	high	5.2
CGGA_1901	S100A9 in GBM	CGGA	Glioblastoma	7.971	high	17.8
CGGA_1912	S100A9 in GBM	CGGA	Glioblastoma	9.097	high	16.7
CGGA_1946	S100A9 in GBM	CGGA	Glioblastoma	7.622	high	4.7
CGGA_1953	S100A9 in GBM	CGGA	Glioblastoma	7.391	high	2.4
CGGA_1972	S100A9 in GBM	CGGA	Glioblastoma	9.574	high	30.1
CGGA_318	S100A9 in GBM	CGGA	Glioblastoma	9.754	high	7.3
CGGA_342	S100A9 in GBM	CGGA	Glioblastoma	7.800	high	16.4
CGGA_525	S100A9 in GBM	CGGA	Glioblastoma	7.241	high	4.5
CGGA_530	S100A9 in GBM	CGGA	Glioblastoma	7.135	high	16.6
CGGA_568	S100A9 in GBM	CGGA	Glioblastoma	11.957	high	9.4
CGGA_604	S100A9 in GBM	CGGA	Glioblastoma	8.630	high	12.5
CGGA_624	S100A9 in GBM	CGGA	Glioblastoma	8.142	high	4
CGGA_719	S100A9 in GBM	CGGA	Glioblastoma	7.446	high	3.3
CGGA_731	S100A9 in GBM	CGGA	Glioblastoma	7.367	high	16.5
CGGA_789	S100A9 in GBM	CGGA	Glioblastoma	7.384	high	11.3
CGGA_802	S100A9 in GBM	CGGA	Glioblastoma	7.057	high	22.4
CGGA_837	S100A9 in GBM	CGGA	Glioblastoma	7.279	high	14.2
CGGA_842	S100A9 in GBM	CGGA	Glioblastoma	7.514	high	6.7
CGGA_859	S100A9 in GBM	CGGA	Glioblastoma	7.860	high	12.7
CGGA_869	S100A9 in GBM	CGGA	Glioblastoma	9.363	high	17.7
CGGA_876	S100A9 in GBM	CGGA	Glioblastoma	7.604	high	5.2
CGGA_902	S100A9 in GBM	CGGA	Glioblastoma	7.022	high	6.3
CGGA_D30	S100A9 in GBM	CGGA	Glioblastoma	6.752	high	7.1
CGGA_D35	S100A9 in GBM	CGGA	Glioblastoma	7.394	high	21.7
CGGA_D36	S100A9 in GBM	CGGA	Glioblastoma	7.923	high	1.1
CGGA_D37	S100A9 in GBM	CGGA	Glioblastoma	7.276	high	31.7
CGGA_D51	S100A9 in GBM	CGGA	Glioblastoma	8.158	high	4.4
CGGA_P102	S100A9 in GBM	CGGA	Glioblastoma	8.283	high	41.7
CGGA_P143	S100A9 in GBM	CGGA	Glioblastoma	8.102	high	8.6
CGGA_P160	S100A9 in GBM	CGGA	Glioblastoma	7.503	high	7.2
CGGA_P182	S100A9 in GBM	CGGA	Glioblastoma	7.105	high	37.8
CGGA_P199	S100A9 in GBM	CGGA	Glioblastoma	6.883	high	6.7
CGGA_P28	S100A9 in GBM	CGGA	Glioblastoma	7.131	high	3.5
CGGA_P415	S100A9 in GBM	CGGA	Glioblastoma	9.389	high	6.4
CGGA_P585	S100A9 in GBM	CGGA	Glioblastoma	6.834	high	14.2
CGGA_P7	S100A9 in GBM	CGGA	Glioblastoma	9.548	high	3.6
CGGA_P89	S100A9 in GBM	CGGA	Glioblastoma	8.133	high	9.7
CGGA_1008	S100A9 in GBM	CGGA	Glioblastoma	1.856	low	10.4
CGGA_1017	S100A9 in GBM	CGGA	Glioblastoma	2.498	low	25.2
CGGA_1023	S100A9 in GBM	CGGA	Glioblastoma	2.392	low	22.4
CGGA_1039	S100A9 in GBM	CGGA	Glioblastoma	3.206	low	23.9
CGGA_1041	S100A9 in GBM	CGGA	Glioblastoma	2.876	low	105.3
CGGA_1060	S100A9 in GBM	CGGA	Glioblastoma	1.791	low	7.5
CGGA_1103	S100A9 in GBM	CGGA	Glioblastoma	3.398	low	19.2
CGGA_1116	S100A9 in GBM	CGGA	Glioblastoma	2.864	low	8.2
CGGA_1129	S100A9 in GBM	CGGA	Glioblastoma	3.029	low	5.5
CGGA_1130	S100A9 in GBM	CGGA	Glioblastoma	2.254	low	13.2
CGGA_1138	S100A9 in GBM	CGGA	Glioblastoma	3.004	low	13.5
CGGA_1139	S100A9 in GBM	CGGA	Glioblastoma	2.658	low	39.3
CGGA_1142	S100A9 in GBM	CGGA	Glioblastoma	1.860	low	33
CGGA_1164	S100A9 in GBM	CGGA	Glioblastoma	0.832	low	33.6
CGGA_1214	S100A9 in GBM	CGGA	Glioblastoma	3.167	low	19.9
CGGA_1216	S100A9 in GBM	CGGA	Glioblastoma	2.446	low	10.3
CGGA_1219	S100A9 in GBM	CGGA	Glioblastoma	1.531	low	69.7
CGGA_1236	S100A9 in GBM	CGGA	Glioblastoma	0.956	low	6.3
CGGA_1248	S100A9 in GBM	CGGA	Glioblastoma	3.234	low	4.5
CGGA_1257	S100A9 in GBM	CGGA	Glioblastoma	2.570	low	6.3
CGGA_1271	S100A9 in GBM	CGGA	Glioblastoma	1.043	low	17.2
CGGA_1275	S100A9 in GBM	CGGA	Glioblastoma	3.237	low	6
CGGA_1283	S100A9 in GBM	CGGA	Glioblastoma	0.111	low	0.7
CGGA_1299	S100A9 in GBM	CGGA	Glioblastoma	3.213	low	18.1
CGGA_1320	S100A9 in GBM	CGGA	Glioblastoma	0.740	low	1.9
CGGA_1332	S100A9 in GBM	CGGA	Glioblastoma	1.787	low	16.8
CGGA_1337	S100A9 in GBM	CGGA	Glioblastoma	2.602	low	71.4
CGGA_1370	S100A9 in GBM	CGGA	Glioblastoma	1.433	low	10.7
CGGA_1375	S100A9 in GBM	CGGA	Glioblastoma	1.521	low	3
CGGA_1378	S100A9 in GBM	CGGA	Glioblastoma	3.013	low	12.4
CGGA_139	S100A9 in GBM	CGGA	Glioblastoma	1.521	low	22.8
CGGA_1394	S100A9 in GBM	CGGA	Glioblastoma	1.975	low	4.6
CGGA_1403	S100A9 in GBM	CGGA	Glioblastoma	2.114	low	22.3
CGGA_1409	S100A9 in GBM	CGGA	Glioblastoma	-0.152	low	76.8
CGGA_1410	S100A9 in GBM	CGGA	Glioblastoma	2.457	low	27.1
CGGA_1412	S100A9 in GBM	CGGA	Glioblastoma	2.685	low	9.7
CGGA_1418	S100A9 in GBM	CGGA	Glioblastoma	3.265	low	9.4
CGGA_1422	S100A9 in GBM	CGGA	Glioblastoma	3.209	low	6.7
CGGA_1425	S100A9 in GBM	CGGA	Glioblastoma	1.444	low	21
CGGA_1429	S100A9 in GBM	CGGA	Glioblastoma	2.599	low	75.7
CGGA_1430	S100A9 in GBM	CGGA	Glioblastoma	3.116	low	5.3
CGGA_1450	S100A9 in GBM	CGGA	Glioblastoma	2.138	low	31.5
CGGA_1460	S100A9 in GBM	CGGA	Glioblastoma	2.854	low	6.9
CGGA_1467	S100A9 in GBM	CGGA	Glioblastoma	1.195	low	28.5
CGGA_1476	S100A9 in GBM	CGGA	Glioblastoma	1.868	low	9.8
CGGA_1478	S100A9 in GBM	CGGA	Glioblastoma	1.949	low	17.8
CGGA_1486	S100A9 in GBM	CGGA	Glioblastoma	2.548	low	6
CGGA_1494	S100A9 in GBM	CGGA	Glioblastoma	2.121	low	8.8
CGGA_1496	S100A9 in GBM	CGGA	Glioblastoma	2.170	low	10.8
CGGA_1521	S100A9 in GBM	CGGA	Glioblastoma	3.299	low	6.7
CGGA_1529	S100A9 in GBM	CGGA	Glioblastoma	2.696	low	19.2
CGGA_1539	S100A9 in GBM	CGGA	Glioblastoma	2.039	low	68.6
CGGA_1571	S100A9 in GBM	CGGA	Glioblastoma	3.328	low	13.5
CGGA_1611	S100A9 in GBM	CGGA	Glioblastoma	2.025	low	38.1
CGGA_1635	S100A9 in GBM	CGGA	Glioblastoma	-0.252	low	10.9
CGGA_1650	S100A9 in GBM	CGGA	Glioblastoma	2.818	low	42.2
CGGA_1656	S100A9 in GBM	CGGA	Glioblastoma	1.628	low	4.9
CGGA_1697	S100A9 in GBM	CGGA	Glioblastoma	3.392	low	2.9
CGGA_1699	S100A9 in GBM	CGGA	Glioblastoma	3.415	low	56.2
CGGA_1702	S100A9 in GBM	CGGA	Glioblastoma	2.546	low	5.5
CGGA_1713	S100A9 in GBM	CGGA	Glioblastoma	2.828	low	10.9
CGGA_1735	S100A9 in GBM	CGGA	Glioblastoma	2.978	low	26.7
CGGA_1740	S100A9 in GBM	CGGA	Glioblastoma	1.807	low	11.9
CGGA_1758	S100A9 in GBM	CGGA	Glioblastoma	3.291	low	13.6
CGGA_1769	S100A9 in GBM	CGGA	Glioblastoma	3.284	low	48.5
CGGA_1780	S100A9 in GBM	CGGA	Glioblastoma	2.830	low	47.5
CGGA_1812	S100A9 in GBM	CGGA	Glioblastoma	3.392	low	25.6
CGGA_1819	S100A9 in GBM	CGGA	Glioblastoma	3.236	low	43.5
CGGA_1870	S100A9 in GBM	CGGA	Glioblastoma	1.960	low	38.7
CGGA_2038	S100A9 in GBM	CGGA	Glioblastoma	2.580	low	
CGGA_2053	S100A9 in GBM	CGGA	Glioblastoma	1.480	low	21
CGGA_2106	S100A9 in GBM	CGGA	Glioblastoma	0.872	low	16.5
CGGA_374	S100A9 in GBM	CGGA	Glioblastoma	2.064	low	16.2
CGGA_494	S100A9 in GBM	CGGA	Glioblastoma	2.011	low	135.1
CGGA_518	S100A9 in GBM	CGGA	Glioblastoma	2.727	low	7
CGGA_669	S100A9 in GBM	CGGA	Glioblastoma	3.349	low	20
CGGA_700	S100A9 in GBM	CGGA	Glioblastoma	3.252	low	5.6
CGGA_747	S100A9 in GBM	CGGA	Glioblastoma	1.930	low	31.9
CGGA_804	S100A9 in GBM	CGGA	Glioblastoma	3.089	low	20.2
CGGA_822	S100A9 in GBM	CGGA	Glioblastoma	1.852	low	73.5
CGGA_878	S100A9 in GBM	CGGA	Glioblastoma	2.467	low	28.4
CGGA_D03	S100A9 in GBM	CGGA	Glioblastoma	2.342	low	13.9
CGGA_D09	S100A9 in GBM	CGGA	Glioblastoma	2.757	low	7.7
CGGA_D57	S100A9 in GBM	CGGA	Glioblastoma	3.211	low	25.2
CGGA_P116	S100A9 in GBM	CGGA	Glioblastoma	2.742	low	10
CGGA_P136	S100A9 in GBM	CGGA	Glioblastoma	1.144	low	27.7
CGGA_P15	S100A9 in GBM	CGGA	Glioblastoma	1.930	low	23.8
CGGA_P164	S100A9 in GBM	CGGA	Glioblastoma	1.251	low	39.1
CGGA_P178	S100A9 in GBM	CGGA	Glioblastoma	2.511	low	35.5
CGGA_P205	S100A9 in GBM	CGGA	Glioblastoma	3.227	low	19.2
CGGA_P22	S100A9 in GBM	CGGA	Glioblastoma	2.709	low	13.3
CGGA_P280	S100A9 in GBM	CGGA	Glioblastoma	3.036	low	32.2
CGGA_P283	S100A9 in GBM	CGGA	Glioblastoma	2.982	low	31.8
CGGA_P596	S100A9 in GBM	CGGA	Glioblastoma	3.230	low	13.5
CGGA_P609	S100A9 in GBM	CGGA	Glioblastoma	1.428	low	12.5

Cohort of human glioblastomas to estimate the prognostic value of S100A9 to radiotherapy. Both TCGA and CGGA cohorts were used to evaluate the correlation between *S100A9* gene expression and overall survival (OS) from glioblastoma diagnosis.

Supplementary Table 20.

Supplementary Table 20_Cohorts of human primary breast and lung cancers to estimate the prognostic value of S100A8 to radiotherapy_Corresponds to Fig.S9I-J

Study ID	Patient ID	Sample ID	S100A8 mRNA Expression, RSEM (Batch normalized)	grouped	Radiation Therapy	Tumor Type	PFS status	PFS (months)
[illegible]	[illegible]	[illegible]	[illegible]	[illegible]	[illegible]	[illegible]	[illegible]	[illegible]

[illegible]	[illegible]	[illegible]	[illegible]	[illegible]	[illegible]	[illegible]	[illegible]	[illegible]
[illegible]	[illegible]	[illegible]	[illegible]	[illegible]	[illegible]	[illegible]	[illegible]	[illegible]

Cohorts of human primary breast and lung cancers to estimate the prognostic value of

S100A9 to radiotherapy. TCGA data on primary breast and lung cancer was filtered by the presence of WBRT and levels of S100A9 evaluated for any clinical correlation.

Supplementary Table 21.

Supplementary Table 21_Cohort of 71 human brain metastases with available blood liquid biopsy_Corresponds to Fig.5H and Fig.S5K

Sample	Sample ID	Retrospective study	Cohort	Primary tumor	Gender	Age at brain met diagnosis	KPS	Date diagnosis brain metastasis	WBRT	Date WBRT	SRS	Date neurosurgery	Date of blood collection	S100A9 ELISA (pg/ml)	Date of death	OS from Dx brain metastasis
#1	0907/008/1811	S100A9 liquid biopsies from patients with WBRT	RCSI Dublin, Ireland	Breast	female	50		04/03/2013	Yes	14/03/2013	no	28/02/2013	14/05/2013	0	15/09/2014	560
#2	0907/008/187	S100A9 liquid biopsies from patients with WBRT	RCSI Dublin, Ireland	Breast	female	34		06/06/2014	Yes	07/07/2014	no	04/06/2014	04/06/2014	0	26/12/2018	1664
#3	0907/008/2239	S100A9 liquid biopsies from patients with WBRT	RCSI Dublin, Ireland	Breast	female	54		14/11/2018	Yes	NA	no	09/11/2018	09/11/2018	0	04/03/2019	110
#4	LCBrM2	S100A9 liquid biopsies from patients with WBRT	UKE Hamburg, Germany	Lung	male	78	70	29/05/2017	Yes	NA	no	06/06/2017	01/06/2017	0	12/12/2017	197
#5	LCBrM3	S100A9 liquid biopsies from patients with WBRT	UKE Hamburg, Germany	Lung	female	65	80	12/03/2018	Yes	NA	no	10/04/2018	10/04/2018	0	29/07/2018	139
#6	LCBrM9	S100A9 liquid biopsies from patients with WBRT	UKE Hamburg, Germany	Lung	male	57	80	01/05/2018	Yes	NA	no	NA	29/05/2018	0	12/01/2019	256
#7	BCBrM3	S100A9 liquid biopsies from patients with WBRT	UKE Hamburg, Germany	Breast	female	67	80	16/04/2018	Yes	NA	no	09/05/2018	02/05/2018	0	24/05/2018	38
#8	BCBrM9	S100A9 liquid biopsies from patients with WBRT	UKE Hamburg, Germany	Breast	female	49	70	01/03/2016	Yes	NA	no	NA	25/07/2018	0	02/08/2019	1249
#9	1	S100A9 liquid biopsies from patients with WBRT	US Zuerich, Switzerland	melanoma	female	64	60	15/01/2010	Yes	15/06/2010	no		11/06/2010	0	21/07/2010	187
#10	2	S100A9 liquid biopsies from patients with WBRT	US Zuerich, Switzerland	breast	female	53	60	05/03/2010	Yes	28/04/2010	no		09/07/2010	0	04/02/2012	701
#11	4	S100A9 liquid biopsies from patients with WBRT	US Zuerich, Switzerland	melanoma	female	49	90	16/12/2009	Yes	18/12/2009	no		22/12/2009	0	18/05/2010	153
#12	6	S100A9 liquid biopsies from patients with WBRT	US Zuerich, Switzerland	lung	male	75	70	01/03/2011	Yes	28/03/2011	no		04/03/2011	0	13/07/2011	134
#13	7	S100A9 liquid biopsies from patients with WBRT	US Zuerich, Switzerland	lung	male	75	70	06/01/2015	Yes	10/03/2015	no		28/03/2014	0	05/08/2017	942
#14	0907/008/1125	S100A9 liquid biopsies from patients with WBRT	RCSI Dublin, Ireland	breast	female	31		19/01/2012	Yes	U	no	NA	13/06/2011	0	25/4/13	462
#15	0907/008/2447	S100A9 liquid biopsies from patients with WBRT	RCSI Dublin, Ireland	breast	female	47		02/09/2019	Yes	23/01/2020	no	25-Oct-19	25/10/2019	0	3/12/20	458
#16	0907/008/1728	S100A9 liquid biopsies from patients with WBRT	RCSI Dublin, Ireland	breast	female	58		05/07/2018	Yes	23/01/2019	no	18-Jul-18	05/07/2018	0	4/5/19	303
#17	0907/008/2186	S100A9 liquid biopsies from patients with WBRT	RCSI Dublin, Ireland	breast	female	51		01/08/2018	Yes	27/09/2018	no	13-Aug-18	13/09/2018	0	30/12/18	151
#18	0907/008/1891	S100A9 liquid biopsies from patients with WBRT	RCSI Dublin, Ireland	breast	female	70		05/11/2012	Yes	19/10/2015	no	NA	26/09/2012	0	13/12/15	1133
#19	ONC182	S100A9 liquid biopsies from patients with WBRT	IDBGI Girona, Spain	lung	female	40	80	20/06/2018	Yes	11/07/2018	no		22/06/2018	0	27/10/2018	129
#20	ONC183	S100A9 liquid biopsies from patients with WBRT	IDBGI Girona, Spain	lung	male	51	60	21/08/2018	Yes	05/11/2018	no		22/08/2018	0	27/06/2019	310
#21	ONC184	S100A9 liquid biopsies from patients with WBRT	IDBGI Girona, Spain	lung	female	61	70	16/08/2018	Yes	20/08/2018	no		22/08/2018	0	12/11/2018	88
#22	ONC196	S100A9 liquid biopsies from patients with WBRT	IDBGI Girona, Spain	lung	male	58	90	26/06/2019	Yes	04/07/2019	no		05/03/2019	0	18/09/2019	84
#23	ONC198	S100A9 liquid biopsies from patients with WBRT	IDBGI Girona, Spain	lung	female	64	90	01/03/2019	Yes	20/03/2019	no		25/03/2019	0	07/01/2021	678
#24	ONC199	S100A9 liquid biopsies from patients with WBRT	IDBGI Girona, Spain	lung	male	63	70	01/02/2019	Yes	09/07/2019	no		22/03/2019	0	15/09/2019	226
#25	ONC1910	S100A9 liquid biopsies from patients with WBRT	IDBGI Girona, Spain	lung	male	63	80	18/04/2019	Yes	28/05/2019	no		29/03/2019	0	09/08/2019	113
#26	ONC1912	S100A9 liquid biopsies from patients with WBRT	IDBGI Girona, Spain	lung	male	62	90	26/04/2019	Yes	20/06/2019	no		25/04/2019	0	05/03/2020	314
#27	ONC1915	S100A9 liquid biopsies from patients with WBRT	IDBGI Girona, Spain	lung	female	75	80	07/06/2019	Yes	29/06/2020	no		13/06/2019	0	13/09/2020	464
#28	ONC1920	S100A9 liquid biopsies from patients with WBRT	IDBGI Girona, Spain	lung	female	66	70	10/07/2019	Yes	01/08/2019	no		17/07/2019	0	09/09/2019	61
#29	ONC1921	S100A9 liquid biopsies from patients with WBRT	IDBGI Girona, Spain	lung	female	55	90	20/07/2019	Yes	22/08/2019	no		02/08/2019	0	04/02/2020	199
#30	ONC1922	S100A9 liquid biopsies from patients with WBRT	IDBGI Girona, Spain	lung	male	74	90	27/07/2019	Yes	29/08/2019	no		06/08/2019	0	11/02/2020	199
#31	ONC1926	S100A9 liquid biopsies from patients with WBRT	IDBGI Girona, Spain	lung	male	70	70	01/08/2019	Yes	26/09/2019	no		27/09/2019	0	13/12/2019	134
#32	ONC1927	S100A9 liquid biopsies from patients with WBRT	IDBGI Girona, Spain	lung	male	62	80	07/10/2019	Yes	23/10/2019	no		07/10/2019	0	26/11/2019	50
#33	ONC2036	S100A9 liquid biopsies from patients with WBRT	IDBGI Girona, Spain	lung	female	56	70	31/12/2019	Yes	06/02/2020	no		31/01/2020	0	31/12/2020	366
#34	ONC2038	S100A9 liquid biopsies from patients with WBRT	IDBGI Girona, Spain	lung	female	60	100	01/12/2019	Yes	04/02/2020	no		21/02/2020	0	12/12/2020	377
#35	H001835T1Pa	S100A9 liquid biopsies from patients with WBRT	UoM Manchester, UK	melanoma	female	45	100	07/01/2016	Yes	17/02/2016	no	14/01/2016	14/01/2016	0	30/12/2016	358
#36	H001845T1Pb	S100A9 liquid biopsies from patients with WBRT	UoM Manchester, UK	melanoma	female	68	100	07/12/2015	Yes		no	23/12/2015	23/12/2015	0	09/10/2016	307
#37	H001839TPa	S100A9 liquid biopsies from patients with WBRT	UoM Manchester, UK	melanoma	male	64	60	23/12/2015	Yes		no	22/01/2016	22/01/2016	0	11/07/2017	566
#38	3045	S100A9 liquid biopsies from patients with WBRT	UoM Manchester, UK	melanoma	female	40s		17/05/2019	Yes		no	07/06/2019		0	02/12/2019	199
#39	2574	S100A9 liquid biopsies from patients with WBRT	UoM Manchester, UK	melanoma	male	60s		07/06/2017	Yes		no	21/06/2017		0	03/11/2018	514
#40	2016/148	S100A9 liquid biopsies from patients with WBRT	Universidad de Navarra, Spain	lung	male	59	50	11/05/2016	Yes	16/05/2016	no		11-05-2016	0	12-09-2016	124
#41	2019/475	S100A9 liquid biopsies from patients with WBRT	Universidad de Navarra, Spain	lung	female	46	30	05/11/2019	Yes	07-11-2019	no		16-10-2019	0	17-12-2019	42
#42	2019/262	S100A9 liquid biopsies from patients with WBRT	Universidad de Navarra, Spain	lung	male	65	80	26/04/2019	Yes	15/07/2019	no		11-07-2019	0	05-12-2019	223
#43	2017/114	S100A9 liquid biopsies from patients with WBRT	Universidad de Navarra, Spain	lung	male	69	60	19/11/2019	Yes	November 2019	no		07-08-2019	0	06-12-2019	17
#44	2018/071	S100A9 liquid biopsies from patients with WBRT	Universidad de Navarra, Spain	lung	male	63	60	13/03/2019	Yes	18/03/2019	no		06-03-2019	0	10-07-2019	119
#45	2016/032	S100A9 liquid biopsies from patients with WBRT	Universidad de Navarra, Spain	lung	male	49	90	22/01/2016	Yes	25/01/2016	no		21-01-2016	0	23-02-2016	32
#46	2016/132	S100A9 liquid biopsies from patients with WBRT	Universidad de Navarra, Spain	lung	female	55	80	23/11/2016	Yes		no		23-11-2016	0	16-12-2019	1118
#47	2016/135	S100A9 liquid biopsies from patients with WBRT	Universidad de Navarra, Spain	lung	female	46	80	28/04/2016	Yes	27-05-2016	no		28-04-2016	0	11-06-2016	44
#48	2016/220	S100A9 liquid biopsies from patients with WBRT	Universidad de Navarra, Spain	melanoma	female	53	90	28/07/2016	Yes	01/08/2016	no		29-07-2016	0	26-12-2016	151
#49	2018/070	S100A9 liquid biopsies from patients with WBRT	Universidad de Navarra, Spain	Lung	female	51	80	26/02/2018	Yes	30-10-2019	no		04-09-2019	0	30-04-2020	794
#50	2016/299	S100A9 liquid biopsies from patients with WBRT	Universidad de Navarra, Spain	Lung	male	63	90	23/09/2016	Yes	30/01/2017	no		23-01-2017	0	22-05-2017	241
#51	2018/090	S100A9 liquid biopsies from patients with WBRT	Universidad de Navarra, Spain	Lung	female	41	70	14/02/2018	Yes	12/03/2018	no		09-03-2018	0	15-11-2018	274
#52	2018/479	S100A9 liquid biopsies from patients with WBRT	Universidad de Navarra, Spain	renal carcinoma	female	59	80	27/12/2018	Yes	20-02-2019	no		29-01-2019	0	03-06-2019	158
#53	2019/238	S100A9 liquid biopsies from patients with WBRT	Universidad de Navarra, Spain	colorectal carcinoma	male	56	70	22/10/2019	Yes	24/10/2019	no		22-05-2019	0	10-01-2020	80
#54	2020/120	S100A9 liquid biopsies from patients with WBRT	Universidad de Navarra, Spain	osteosarcoma	male	20	50	04/03/2021	Yes	05-03-2021	no		01-03-2021	0	03-06-2021	91
#55	2019/204	S100A9 liquid biopsies from patients with WBRT	Universidad de Navarra, Spain	lung	male	72	90	09/05/2019	Yes	05/05/2019	no		07-05-2019	0	15-10-2019	159
#56	2020/150	S100A9 liquid biopsies from patients with WBRT	Universidad de Navarra, Spain	Lung	male	47	40	27/11/2019	Yes	02/04/2020	no		24-02-2020	0	16-04-2020	141
#57	2020/231	S100A9 liquid biopsies from patients with WBRT	Universidad de Navarra, Spain	melanoma	female	64	80	03/06/2020	Yes	08/06/2020	no		26-05-2020	0	30-01-2021	241
#58	2020/390	S100A9 liquid biopsies from patients with WBRT	Universidad de Navarra, Spain	Lung	male	63	60	15/09/2020	Yes	16/09/2020	no		08-09-2020	0	30-10-2020	45
#59	2020/516	S100A9 liquid biopsies from patients with WBRT	Universidad de Navarra, Spain	Lung	male	65	50	19/11/2020	Yes	12/04/2021	no		08-04-2021	0	25-08-2021	279
#60	0907/008/347	S100A9 liquid biopsies from patients with WBRT	RCSI Dublin, Ireland	Breast	female	42		29/05/2015	Yes	NA	no	29/05/2015	28/05/2015	17.34	13/10/2015	137
#61	5	S100A9 liquid biopsies from patients with WBRT	US Zuerich, Switzerland	prostate	male	80	70	17/09/2014	Yes	02/10/2014	no		19/09/2014	23.86	19/01/2015	124
#62	2216	S100A9 liquid biopsies from patients with WBRT	UoM Manchester, UK	melanoma	female	60s		15/12/2015	Yes		no	26/01/2016		140.6319	27/06/2016	195
#63	BCBrM2	S100A9 liquid biopsies from patients with WBRT	UKE Hamburg, Germany	Breast	female	59	80	06/04/2018	Yes	NA	no	10/04/2018	10/04/2018	153.52	01/05/2018	25
#64	EXO-T-00193	S100A9 liquid biopsies from patients with WBRT	H12O Madrid, Spain	Lung	male	69	80	01/11/2019	Yes	NA	no	NA	07/11/2019	162.8692	03/07/2020	245
#65	2020/306	S100A9 liquid biopsies from patients with WBRT	Universidad de Navarra, Spain	lung	male	67	90	20/07/2020	Yes	22/07/2020	no		20-07-2020	194.63	18-01-2021	182
#66	BCBrM1	S100A9 liquid biopsies from patients with WBRT	UKE Hamburg, Germany	Breast	female	46	90	27/10/2017	Yes	NA	no	14/11/2017	07/11/2017	427.71	03/12/2017	37
#67	ONC185	S100A9 liquid biopsies from patients with WBRT	IDBGI Girona, Spain	lung	male	55	80	27/08/2018	Yes	05/09/2018	no		30/08/2018	604.54	28/02/2019	185
#68	Exo_T_00154	S100A9 liquid biopsies from patients with WBRT	H12O Madrid, Spain	Lung	female	64	90	01/08/2019	Yes	NA	no	NA	12/08/2019	687.3477	06/05/2020	279
#69	Exo_T_00172	S100A9 liquid biopsies from patients with WBRT	H12O Madrid, Spain	Lung	male	71	80	01/09/2019	Yes	NA	no	NA	01/10/2019	1513.727	12/11/2019	72
#70	2016/383	S100A9 liquid biopsies from patients with WBRT	Universidad de Navarra, Spain	Lung	male	73	70	31/05/2017	Yes	26/06/2017	no		31-05-2017	2000.00	06-08-2017	67
#71	2019/524	S100A9 liquid biopsies from patients with WBRT	Universidad de Navarra, Spain	Lung	male	64	70	02/06/2020	Yes	08/06/2020	no		02-06-2020	2000.00	15-11-2020	166
#1	0907/008/914	S100A9 liquid biopsies from patients without WBRT	RCSI Dublin, Ireland	Breast	female	50		08/02/2018	No	NA	no	05/02/2018	05/02/2018	0	22/02/2018	0.5
#2	0907/008/2216	S100A9 liquid biopsies from patients without WBRT	RCSI Dublin, Ireland	Breast	female	48		12/11/2018	No	NA	no	NA	03/10/2018	0	ALIVE	28.8
#3	0907/008/1746	S100A9 liquid biopsies from patients without WBRT	RCSI Dublin, Ireland	Breast	female	59		08/08/2013	No	NA	yes	08/08/2016	22/02/2012	0	28/07/2014	11.8
#4	0907/008/328	S100A9 liquid biopsies from patients without WBRT	RCSI Dublin, Ireland	Breast	female	29		16/04/2015	No	NA	yes	13/04/2015	13/04/2015	0	22/06/2015	2.2
#5	0907/008/298	S100A9 liquid biopsies from patients without WBRT	RCSI Dublin, Ireland	Breast	female	75		10/02/2015	No	NA	yes	17/02/2015	12/02/2015	0	24/09/2016	19.7
#6	0907/008/306	S100A9 liquid biopsies from patients without WBRT	RCSI Dublin, Ireland	Breast	female	28		31/12/2014	No	NA	yes	03/03/2015	02/03/2015	0	22/04/2015	3.7
#7	0907/008/1082	S100A9 liquid biopsies from patients without WBRT	RCSI Dublin, Ireland	Breast	female	50		09/05/2014	No	NA	yes	09/05/2014	17/09/2008	0	31/10/2017	42.4
#8	0907/008/848	S100A9 liquid biopsies from patients without WBRT	RCSI Dublin, Ireland	Breast	female	33		07/11/2017	No	NA	yes	07/11/2017	07/11/2017	0	01/08/2018	8.9
#9	0907/008/1728	S100A9 liquid biopsies from patients without WBRT	RCSI Dublin, Ireland	Breast	female	58		08/06/2018	No	NA	yes	06/07/2018	18/11/2016	0	04/05/2019	11.0
#10	0907/008/645	S100A9 liquid biopsies from patients without WBRT	RCSI Dublin, Ireland	Breast	female	51		28/05/2019	No	NA	yes	28/05/2019	13/01/2017	0	19/06/2020	12.9
#11	0907/008/2228	S100A9 liquid biopsies from patients without WBRT	RCSI Dublin, Ireland	Breast	female	73		22/09/2017	No	NA	yes	19/09/2017	23/10/2018	0	30/01/2020	28.7
#12	0907/008/2241	S100A9 liquid biopsies from patients without WBRT	RCSI Dublin, Ireland	Breast	female	73		16/11/2018	No	NA	yes	13/11/2018	13/11/2018	0	28/02/2020	15.6
#13	0907/008/2215	S100A9 liquid biopsies from patients without WBRT	RCSI Dublin, Ireland	Breast	female	45		09/11/2018	No	NA	NA	NA	17/10/2018	0	ALIVE	28.9
#14	0907/008/1685	S100A9 liquid biopsies from patients without WBRT	RCSI Dublin, Ireland	Breast	female	80		18/03/2014	No	NA	NA	NA	11/01/2012	0	16/05/2014	2.0
#15	0907/008/175	S100A9 liquid biopsies from patients without WBRT	RCSI Dublin, Ireland	Breast	female	56		04/03/2011	No	NA	NA	04/03/2011	02/05/2014	0	27/07/2019	102.2
#16	0907/008/232	S100A9 liquid biopsies from patients without WBRT	RCSI Dublin, Ireland	Breast	female	52		09/10/2014	No	NA	NA	03/10/2014	03/10/2014	0	07/10/2014	0.0
#17	0907/008/2170	S100A9 liquid biopsies from patients without WBRT	RCSI Dublin, Ireland	Breast	female	66		30/07/2018	No	NA	NA	25/07/2018	24/07/2018	0	04/11/2018	3.2
#18	0907/008/2217	S100A9 liquid biopsies from patients without WBRT	RCSI Dublin, Ireland	Breast	female	51		12/11/2018	No	NA	NA	NA	03/10/2018	0	ALIVE	28.8
#19	Exo_T_00147	S100A9 liquid biopsies from patients without WBRT	H12O Madrid, Spain	Lung	male	56	80	01/12/2018	No	NA	yes	NA	17/07/2019	0	ALIVE	28.2
#20	0907/008/2203	S100A9 liquid biopsies from patients without WBRT	RCSI Dublin, Ireland	Breast	female	67		24/09/2018	No	NA	NA	18/09/2018	18/09/2018	6.76	30/01/2019	4.3
#21	0907/008/1816	S100A9 liquid biopsies from patients without WBRT	RCSI Dublin, Ireland	Breast	female	51		11/04/2012	No	NA	NA	NA	18/05/2012	15.78	ALIVE	109.0
#22	0907/008/992	S100A9 liquid biopsies from patients without WBRT	RCSI Dublin, Ireland	Breast	female	58		30/05/2018	No	NA	yes	30/05/2018	30/05/2018	21.86	05/05/2019	11.3
#23	0907/008/884	S100A9 liquid biopsies from patients without WBRT	RCSI Dublin, Ireland	Breast	female	56		02/12/2015	No	NA	yes	NA	19/12/2017	22.47	01/07/2018	31.4
#24	0907/008/2214	S100A9 liquid biopsies from patients without WBRT	RCSI Dublin, Ireland	Breast	female	76		05/10/2018	No	NA	NA	28/09/2018	28/09/2018	171.26	ALIVE	30.1
#25	0907/008/054	S100A9 liquid biopsies from patients without WBRT	RCSI Dublin, Ireland	Breast	female	38		12/06/2015	No	NA	yes	12/06/2015	12/06/2015	194.24	11/09/2020	63.9
#26	Exo_T_00140	S100A9 liquid biopsies from patients without WBRT	H12O Madrid, Spain	Lung	male	62	80	01/06/2019	No	NA	no	NA	19/06/2019	229.4866	30/01/2020	8.1
#27	0907/008/235	S100A9 liquid biopsies from patients without WBRT	RCSI Dublin, Ireland	Breast	female	NA		13/10/2013	No	NA	NA	NA	13/10/2013	258.45	31/12/2015	27.0
#28	EXO-T-00241-T	S100A9 liquid biopsies from patients without WBRT	H12O Madrid, Spain	Lung	male	65	70	01/01/2020	No	NA	yes	NA	07/02/2020	877.2345	ALIVE	15.0

Cohort of 71 human brain metastases with available blood liquid biopsy. Blood liquid biopsies from three independent institutions were collected and evaluated for S100A9 circulating levels using ELISA. Samples were obtained from patients with brain metastasis that received or not WBRT. The correlation of S100A9 levels with overall survival (OS) from brain metastasis diagnosis was later evaluated. N/A: data not available.

Supplementary Table 22.

Supplementary Table 22_Human brain metastases processed as patient-derived organotypic cultures (PDOC)_Corresponds to Fig.6I-K and Fig.S6J-O

Sample	ID	hBrM	H12O ID	Gender	Birthdate	KPS 2nd surgery	Experiment	Primary tumor	S100A9 IF score	Date of 1st brain met surgery	Date of 1st brain met radiation	Date of relapsed brain met surgery
#1	Patient #1	hBrM#41	17-37637	Man	68yo	60	PDOC	Lung	Negative (0)	04/12/2017	Unknown	No relapse
#2	Patient #2	hBrM#62	20-2342	Man	58yo	60	PDOC	Lung	Positive (2)	XX/07/2019	30/07/2019	24/01/2020
#3	Patient #3	hBrM#67	B20-15783	Man	59yo	80	PDOC	Lung	Positive (1)	None	04/12/2020	24/07/2020
#4	Patient #4	hBrM#69	20-16600	Man	56yo	80	PDOC	Lung	Positive (1)	Unknown	Unknown	05/08/2020
#5	Patient #5	hBrM#79	B-20-26624-D	Woman	39yo	80	PDOC	Breast	Positive (1)	04/04/2019	25/02/2019	16/12/2020
#6	Patient #6	hBrM#45	18-8430	Man	67yo	80	PDOC	Lung	Positive (2)	Unknown	Unknown	12/03/2018
#8	Patient #7	hBrM#89	B-21-5970	Woman			PDOC	Breast	Positive (1)			11.03.2021
#9	Patient #8	hBrM#98	B-21-12482	Woman			PDOC	Breast	Positive (1)			19.05.2021

Human brain metastases processed as patient-derived organotypic cultures (PDOC). 8 PDOC established from brain metastasis were evaluated for S100A9 levels by

immunofluorescence and for their response to radiotherapy and FPS-ZM1. Samples from patients #2 to #8 correspond to relapsed metastases that received previous radiotherapy.

Supplementary Table 23.

Supplementary Table 23_Assesment of general health status in tumor free mice one month after completing the treatment with irradiation alone or with FPS-ZM1

	Irradiated + vehicle					Irradiated + FPS-ZM1					Note
Mice tag	#1	#2	#3	#4	#5	#6	#7	#8	#9	#10	
Skin	0	0	0	0	0	0	0	0	0	0	
Fur	0	0	0	0	0	0	0	0	0	0	Presence of white hair in the head (not present pre-treatment) of all mice
Whiskers	0	0	0	0	0	0	0	0	0	0	
Eyes	0	0	0	0	0	0	0	0	0	0	
Movement_regular	0	0	0	0	0	0	0	0	0	0	
Movement_circles	0	0	0	0	0	0	0	0	0	0	
Movement_Unstable	0	0	0	0	0	0	0	0	0	0	
Movement_Other alterations	0	0	0	0	0	0	0	0	0	0	
Agressive response post-manipulation	0	0	0	0	0	0	0	0	0	0	
Any other aspect	0	0	0	0	0	More active	More active	More active	More active	More active	

0: no alteration/ 1: alteration

Assessment of general health status in tumor free mice one month after completing the treatment with irradiation alone or with FPS-ZM1. Results from health status evaluation of two cohorts of mice, one irradiated and treated with a vehicle and the other also irradiated but receiving FPS-ZM1.

Supplementary Table 24.

Supplementary Table 24_Significant P values of behavioral tests

Contextual Fear Conditioning (Extended Data Figure 6g)	Mixed ANOVA	Significant effect of Trial (F(2,70)=125,659; p = 6.89E-24)					
	Pairwise Comparisons (Sidak)	Acquisition/Short Term Memory	Control (p=6.66E-16)				
			Irradiation(p=1.16E-07)				
			Irradiation+FPS-ZM1(p=1.15E-13)				
		Short Term Memory/Context Change	Control (p=0.00022)				
			Irradiation(p=0.022)				
			Irradiation+FPS-ZM1(p=0.001)				
Elevated Plus Maze (Extended Data Fig. 6f)	Intragroup (t test)	Control (p=1E-08), Irradiation (p=4.15E-07), Irradiation+FPS-ZM1 (p=0.000028)					
Morris Water Maze (Fig.6h)	Learning trials	Mixed ANOVA	Significant effect of trial (F(3,108)=90.482; p = 2.4E-29)				
		Pairwise Comparisons (Sidak)	Intragroup	Control	1/2 (p=0.0006)		
					1/3 (p=1.73E-06)		
					1/4 (p=4.92E-14)		
					2/4 (p=0.00014)		
				Irradiation	1/2 (p=0.013)		
					1/3 (p=0.000003)		
					1/4 (p=1.41E-12)		
					2/4 (p=0.00018)		
				Irradiation+FPS-ZM1	1/2 (p=0.00021)		
					1/3 (p=2.33E-12)		
					1/4 (p=0)		
					2/3 (p=0.00022)		
					2/4 (p=6.82E-08)		
			Intergroup	Day 4 Control/Irradiation (p=0.042)			
	Probe	One sample T test (probe quadrant vs rest of quadrants)	Irradiation+FPS-ZM1 (t(19)=2.778, p=0.012) (more time on probe quadrant)				
Pattern Separation (Supplementary Fig.6r)	Mixed ANOVA	Significant effect of trial (F(1,35)=32.953; p=1.69E-06)					
	Pairwise Comparisons (Sidak)	Control (p=0.005)					
		Irradiation (p=0.012)					
		Irradiation+FPS-ZM1 (p=0.00001)					
Novel Object Recognition (Supplementary Fig.6q)	Discrimination Index	Mixed ANOVA	F(2,68)=57.42; p<0.0001				
		Pairwise comparisons (Sidak)	Intragroup	Control	ACQ/STM	0.000034	
					ACQ/LTM	0.00019	
				Irradiation	ACQ/STM	5.00E-08	
					ACQ/LTM	0.00012	
				Irradiation+FPS-ZM1	ACQ/STM	1.8E-09	
					ACQ/LTM	3.2E-06	
	Time Exploring	Mixed ANOVA	F(2,68)=101.955; p<0.0001				
		Pairwise comparisons (Sidak)	Intragroup	Control	ACQ/STM	2.00E-04	
					STM/LTM	6.00E-03	
				Irradiation	ACQ/STM	5.50E-06	
					ACQ/LTM	6.30E-11	
					STM/LTM	3.00E-06	
				Irradiation+FPS-ZM1	ACQ/STM	1.60E-06	
					ACQ/LTM	8.10E-12	
					STM/LTM	5.00E-07	

Significant *P* values of behavioral tests. This table reflects statistical tests applied to Fig. 6h, Extended Data Figure 6f-g, Supplementary Figure 6q-r and their results. Only those reaching statistical significance are shown.

RENACER:

Cecilia Sobrino[39], Nuria Ajenjo[39], Maria-Jesus Artiga[39], Eva Ortega-Paino[39], Patricia Baena[2], Juan Manuel Sepúlveda[3], Ángel Pérez-Núñez[4,5], Jose Fernández-Alén[6], Manuel Valiente[2].

[39]Biobank; CNIO; Madrid, 28029; Spain.

[2]Brain Metastasis Group, CNIO; Madrid, 28029, Spain.

[3]Neuro-Oncology Unit, Hospital Universitario 12 de Octubre; Madrid, 28041, Spain.

[4]Neurosurgery Unit, Hospital Universitario 12 de Octubre; Madrid, 28041, Spain.

[5]Department of Surgery, Universidad Complutense de Madrid, Madrid, 28040; Spain.

[6]Department of Neurosurgery, Hospital Universitario de La Princesa; Madrid, 28006, Spain.
