# Supplementary material for: Stratification of radiosensitive brain metastases based on an actionable S100A9/RAGE resistance mechanism
Source: Nat Med. 2022 Apr 11;28(4):752–65. doi: 10.1038/s41591-022-01749-8 (PMC9018424; doi:10.1038/s41591-022-01749-8)
Supplement: Supplementary file 2 — Reporting Summary [file 41591_2022_1749_MOESM2_ESM.pdf]

nature research

Corresponding author(s): Manuel Valiente

Last updated by author(s): Feb 13, 2022

Reporting Summary

Nature Research wishes to improve the reproducibility of the work that we publish. This form provides structure for consistency and transparency in reporting. For further information on Nature Research policies, see our Editorial Policies and the Editorial Policy Checklist.

Statistics

For all statistical analyses, confirm that the following items are present in the figure legend, table legend, main text, or Methods section.

n/a	Confirmed	
☐	☒	The exact sample size (*n*) for each experimental group/condition, given as a discrete number and unit of measurement
☐	☒	A statement on whether measurements were taken from distinct samples or whether the same sample was measured repeatedly
☐	☒	The statistical test(s) used AND whether they are one- or two-sided<br>*Only common tests should be described solely by name; describe more complex techniques in the Methods section.*
☐	☒	A description of all covariates tested
☐	☒	A description of any assumptions or corrections, such as tests of normality and adjustment for multiple comparisons
☐	☒	A full description of the statistical parameters including central tendency (e.g. means) or other basic estimates (e.g. regression coefficient) AND variation (e.g. standard deviation) or associated estimates of uncertainty (e.g. confidence intervals)
☐	☒	For null hypothesis testing, the test statistic (e.g. *F*, *t*, *r*) with confidence intervals, effect sizes, degrees of freedom and *P* value noted<br>*Give P values as exact values whenever suitable.*
☒	☐	For Bayesian analysis, information on the choice of priors and Markov chain Monte Carlo settings
☒	☐	For hierarchical and complex designs, identification of the appropriate level for tests and full reporting of outcomes
☒	☐	Estimates of effect sizes (e.g. Cohen's *d*, Pearson's *r*), indicating how they were calculated

Our web collection on statistics for biologists contains articles on many of the points above.

Software and code

Policy information about availability of computer code

Data collection	Living Image 4.5 for Bioluminescence Imaging acquision, Quantstudio 6 and 7 for qPCR acquision, FACS DIVA v6.1.3 for flow cytometry, MUX_XYZ16L-8 Animal Activity software for recording animal movement, Illumina Real Time Analysis Software (RTA v2) for sequencing.
Data analysis	GraphPad Prism 8 for statistical analysis, Living Image 4.5 for Bioluminescence Imaging analysis, Rstudio 1.3 for bioinformatic analysis, GSEA 4.1.0 for Gene-Set enrichment analysis, Zen 3.1 for microscopic imaging analysis, Fiji 1.0 for analysis of immunoflourescent images, FlowJo v10.1 for flow cytometry analysis, Quantstudio 6 and 7, Definiens developer XD 2.5 for image analysis, Zen Blue Software v3 for IHC image analysis, Matlab R2018b, MUX_XYZ16L-8 Animal Activity sofware for analysing animal movement, SPSS v.27.<br>For Transcriptomic analysis, Single read sequences were analysed by Nextpresso pipeline as follows: Sequencing quality was analysed with FastQC (http://www.bioinformatics.babraham.ac.uk/projects/fastqc/); reads were aligned to the human genome (GRCh37/hg19) using TopHat-2.0.10 13, Bowtie 1.0.0 14 and Samtools 0.1.19.0 15; transcripts assembly, abundances estimation and differential expression were calculated with Cufflinks 2.2.1 16. Venn diagrams of the commonly up-regulated and down-regulated genes between resistant and sensitive conditions was obtained using Venny v2.0 (bioinfogp.cnb.csic.es/tools/venny). Two groups of patients with low or high gene expression were delineated using the 'survminer' R package v0.4.9.<br>For Single-Cell-RNA-sequencing analysis, Xenocell version 1.0 was used to classify reads between host (mouse) and graft (human). The bollito pipeline was used to perform read analysis, as follows: Sequencing quality was checked with FastQC (http://www.bioinformatics.babraham.ac.uk/projects/fastqc/). Reads were aligned to the human reference genome (GRCh38_p13 from GENCODE 19) with STARsolo (STAR 2.7.3a 20). Seurat 3.2.2 21 was used to check the quality of sequenced cells, explore and quantify single-cell data, obtain cell clusters and specific gene markers.

For manuscripts utilizing custom algorithms or software that are central to the research but not yet described in published literature, software must be made available to editors and reviewers. We strongly encourage code deposition in a community repository (e.g. GitHub). See the Nature Research guidelines for submitting code & software for further information.

Data

Policy information about availability of data

All manuscripts must include a data availability statement. This statement should provide the following information, where applicable:

- Accession codes, unique identifiers, or web links for publicly available datasets
- A list of figures that have associated raw data
- A description of any restrictions on data availability

Access to RNA-seq data is provided from the Gene Expression Omnibus, under the ID GSE173554. Access to scRNA-seq data is provided from the Gene Expression Omnibus, under the ID GSE189024. Access to the extended RNA-seq of breast cancer patients is provided from the Gene expression Omnibus, under the ID GSE184869. References genomes used (GRCh38_p13 and GRCh37/hg19) are available under https://www.ncbi.nlm.nih.gov/assembly/GCF_000001405.39 and https://www.ncbi.nlm.nih.gov/assembly/GCF_000001405.13/. TCGA and CGGA Glioblastoma data used is accesible through http://gliovis.bioinfo.cnio.es. TCGA lung and breast primary cancer data used is accesible through https://www.cbioportal.org.

Field-specific reporting

Please select the one below that is the best fit for your research. If you are not sure, read the appropriate sections before making your selection.

☒ Life sciences ☐ Behavioural & social sciences ☐ Ecological, evolutionary & environmental sciences

For a reference copy of the document with all sections, see nature.com/documents/nr-reporting-summary-flat.pdf

Life sciences study design

All studies must disclose on these points even when the disclosure is negative.

Sample size	Sample size was determined according to our previous experience with both brain organotypic cultures (Martini, Valiente et al. Development. 2009; Valiente et al. Journal of Neuroscience. 2011; Valiente et al. Cell. 2014; Chen et al. Nature. 2016, Priego et al. Nature Medicine 2018) and in vivo models of brain metastasis (Valiente et al. Cell. 2014; Chen et al. Nature. 2016). Organotypic culture includes, at least, 4 individual replicas measured with bioluminescence and histological validation. In vivo brain metastasis models requires, at least, 8 individual animals measured with bioluminescence ex vivo and histological validation. This sample size requirements were achieved in each individual experiment presented in the manuscript. For In vitro experiments ((co-)culture of cancer cells and glia cells), sample size was not based on formal power calculations, but choosen to assure statistical significance and reproducibility of the results. At least three independent experiments were included.
Data exclusions	No data has been excluded from any of the experiments presented in the manuscript
Replication	All data incorporated in the paper has been replicated. The number of replicates is stated in the figure legends.
Randomization	Brain cultures or animals receiving treatments were randomized into control or experimental arms according to:<br>-Brain organotypic cultures: Bioluminiscence imaging at day 0 allowed to generate two groups with homogeneous values so no biased in any of them could interfere with the hypothesis being tested through the use of the inhibitor or genetic manipulations.<br>-Mice were homogenized regarding age (4-8 weeks) to be present in both control and experimental groups at similar percentages so no biased can influence the effect of the inhibitor or genetic manipulation. When mice were treated with inhibitors, mice were randomized according to their bioluminescent signal before treatment was started, so both treatment and vehicle group had comparable tumor-size at starting point.<br>For in vitro experiments no randomization was necessary since cells were counted before seeding, so equal amounts of cells were assured in all treatment or control wells. All cells of control/treatment groups were grown in the same incubator, so equal conditions were assured.
Blinding	For evaluation of human brain metastasis stainings, pathologists were blinded to interpret the staining of human samples. Investigators were not blinded during data collection, however data collection occurs simultaneously to both control and experimental group in the case of organotypic brain cultures, given that both are located within the same plate that was imaged, so exactly the same settings were applied to both groups. The same simultaneous data collection applies to in vitro cancer cell culture with for number analysis. Data collection was blinded in the case of in vivo brain metastasis experiments.<br>For data analysis of stainings of ex vivo cultures or in vivo samples, sample names did not contain information about mouse genoype or treatment to prevent bias. No blinded was applied to other experiments.

Reporting for specific materials, systems and methods

We require information from authors about some types of materials, experimental systems and methods used in many studies. Here, indicate whether each material, system or method listed is relevant to your study. If you are not sure if a list item applies to your research, read the appropriate section before selecting a response.

Materials & experimental systems

n/a	Involved in the study
☐	☒ Antibodies
☐	☒ Eukaryotic cell lines
☒	☐ Palaeontology and archaeology
☐	☒ Animals and other organisms
☐	☒ Human research participants
☒	☐ Clinical data
☒	☐ Dual use research of concern

Methods

n/a	Involved in the study
☒	☐ ChIP-seq
☐	☒ Flow cytometry
☒	☐ MRI-based neuroimaging

Antibodies

Antibodies used

Primary antibodies: GFP (1:1000; GFP-1020, Aves Labs), BrdU (1:500; ab6326, Abcam), Ki67 (1:500; ab15580, Abcam), GFAP (1:1000; MAB360, Millipore), S100A9 (1:200; M0747, Dako), s100a9 (1:100; 73425, Cell Signaling), JunB (1:100; C37F9, Cell Signaling), mCherry (1:500; ab167453, Abcam), RAGE (1:100; AF1179, R&D Systems), S100A8 (1:200; ab92331, Abcam), Iba1 (1:500; 19-19741, Wako), NIMP-R14 (1:100; ab2557, Abcam), TGFα (1:100; ab9585, Abcam), Topo IIα (1:100; sc-365916, Santa Cruz), Transglutaminase II (1:100; 3557, Cell Signaling), CXCR2 (1:100; ab65968, Abcam), HMB-45 (1:500; ab732, Abcam), anti-human CD55-APC (1:200; 555696, BD), anti-MRP14 antibody (1:200; 350705, Biolegend). Secondary antibodies: Alexa-Fluor anti-chicken 488 (A11039), anti-rabbit 555 (A21429), anti-mouse 555 (A21422; all 1:300, Invitrogen), anti-rat 633 (A21094)

Validation

All antibodies used have been used based on its previous validation as provided by the vendor and/or previous publications.
Validation procedures as described on manufacturers website:
GFP-1020, Aves Labs: Antibodies were analyzed by western blot analysis (1:5000 dilution) and immunohistochemistry (1:500 dilution) using transgenic mice expressing the GFP gene product. Western blots were performed using BlokHen® (Aves Labs) as the blocking reagent, and HRP-labeled goat anti-chicken antibodies (Aves Labs, Cat. #H-1004) as the detection reagent. Immunohistochemistry used tetramethyl rhodamine-labeled anti-chicken IgY (https://www.aveslabs.com/products/anti-green-fluorescent-protein-antibody-gfp).
BrdU ab6326, Abcam: Rat monoclonal [BU1/75 (ICR1)] to BrdU - Proliferation Marker, Suitable for: ICC/IF, IHC-P, Flow Cyt, Reacts with: Species independent
Ki67, ab15580, Abcam: Rabbit polyclonal to Ki67, Suitable for: IHC-P, ICC, Knockout validated, Reacts with: Mouse, Human
GFAP, MAB360, Millipore: Routinely evaluated by Western Blot on Mouse brain lysates, Reacts with Bovine, Chicken, Human, Mouse, Porcine (Pig), Rabbit and Rat. Reactivity with other species has not been determined.
S100A9, M0747, Dako: reactivity : horses, dog, pig, cat, Rhesus monkey, application : immunocytochemistry, immunohistochemistry - paraffin section.
S100A9, 73425, Cell Signaling: S100A9 (D3U8M) Rabbit mAb (Rodent Specific) recognizes endogenous levels of total S100A9 protein.
Species Reactivity: Mouse, Rat.
JunB, C37F9, Cell Signaling: JunB (C37F9) Rabbit mAb detects endogenous levels of total JunB protein.
Species Reactivity: Human, Mouse, Rat, Monkey
mCherry, ab167453, Abcam: Rabbit polyclonal to mCherry, Suitable for: WB, ICC/IF, Reacts with: Species independent.
RAGE, AF1179, R&D Systems: Detects human, mouse, and rat RAGE in Western blots. In direct ELISAs, less than 2% cross-reactivity with recombinant canine RAGE is observed. Applications: Western Blot, Immunohistochemistry, Blockade of Receptor-ligand Interaction.
S100A8, ab92331, Abcam: Produced recombinantly (animal-free) for high batch-to-batch consistency and long term security of supply, Rabbit monoclonal [EPR3554] to MRP8, Suitable for: WB, IP, IHC-P, Reacts with: Mouse, Human.
Iba1, 19-19741, Wako: Application Immunohistochemistry, Immunocytochemistry 1:500-1,000 Species reactivity Mouse, Rat, Human, others.
NIMP-R14, ab2557, Abcam: Rat monoclonal [NIMP-R14] to Neutrophil, Suitable for: IHC-Fr, Reacts with: Mouse.
TGFα, ab9585, Abcam: Rabbit polyclonal to TGF alpha, Suitable for: ELISA, IHC-P, Neutralising, Sandwich ELISA, WB, Reacts with: Mouse, Human.
Topo IIα, sc-365916, Santa Cruz: Anti-Topo IIα Antibody (F-12) is a mouse monoclonal IgG1 κ, specific for an epitope mapping between amino acids 1511-1530 at the C-terminus of Topo IIα of human origin. Anti-Topo II alpha Antibody (F-12) is recommended for detection of Topo IIα of mouse, rat and human origin by WB, IP, IF, IHC(P) and ELISA; also reactive with additional species, including porcine.
Transglutaminase II, 3557, Cell Signaling: Species Reactivity: Human, Mouse, Rat, Monkey. Applications: Western Blotting, Immunohistochemistry (Paraffin), Immunofluorescence (Immunocytochemistry).
CXCR2, ab65968, Abcam: Species reactivity: Mouse, Rat, Human, Specificity: No cross reactivity with other proteins.
HMB-45, ab732, Abcam: Suitable for: IHC-Fr, IHC-P, Flow Cyt, Reacts with: Human
CD55-APC, 555696, BD: Reactivity:Human (QC Testing), Application: Flow cytometry (Routinely Tested).
anti-MRP14, 350705, Biolegend: Reactivity Human, Application ICFC - Quality tested.

Eukaryotic cell lines

Policy information about cell lines

Cell line source(s)

MDA231-BrM2, ErbB2-BrM2, 393N1, 482N1, H2030-BrM3, PC9-BrM3, HCC1954-BrM1, CN34-BrM2 were obtained from MSKCC (Joan Massagué lab). E0771-BrM3 and B16/F10-BrM4 was generated in the Valiente Lab. BT-RMS was obtained from Neta Erez (Tel Aviv University). YUMM1.1 was obtained from Marcus Bosenberg (Yale). HEK 293T were obtained from Marcos Malumbres (CNIO Madrid).

Authentication	Cancer cell lines used have been obtained from the same batches that were previously published (Valiente et al. Cell 2014). Cell lines were validated by morphological analysis and their behavior in vivo. To discard any cell line specific mechanism we have incorporated to our study a variety of brain metastatic models.
Mycoplasma contamination	All cell lines were tested to be free from Mycoplasma contamination.
Commonly misidentified lines (See ICLAC register)	No commonly misidentified cell lines were use in this study.

Animals and other organisms

Policy information about studies involving animals; ARRIVE guidelines recommended for reporting animal research

Laboratory animals	We have used nude animals (Hsd:Athymic Nude-Foxn1nu) bough to Envigo and C57BL/6 animals obtained from the inbred colony at CNIO (This colony was established from C57BL/6JOlaHsd from Envigo and it is renewed every two years with new animals from the original colony). Mice used in experiments have 4-10 weeks of age. Nude mice are females. C57BL/6 mice are males and females, with an equal distribution between control and experimental cohorts. S100A9-/- mice were obtained from an inbred colony at CNIO and the strain was generated by Sergei Grivennikov. All genetically modified mice were genotyped.<br>All mice have been housed in pathogen-free conditions, with a 12h light/dark-cycle between 8:00 and 20:00 in a temperature-controlled room 23 +/- 1° Celsius) following the recommendations of the EUMORPHIA consortium for animal housing. Food and water were provided ad libitum.
Wild animals	No wild animals were used in this study.
Field-collected samples	This study did not involve field-collected samples.
Ethics oversight	All animal experiments were performed in accordance with protocol approved by the CNIO, Instituto de Salud Carlos III and Comunidad de Madrid Institutional Animal Care and Use Committee (PROEX 168/15, PROEX 211/17 and PROEX 135/19)

Note that full information on the approval of the study protocol must also be provided in the manuscript.

Human research participants

Policy information about studies involving human research participants

Population characteristics	Available population characteristics of the different retrospective cohorts used in this study are outlined in the corresponding tables.
Recruitment	All brain metastasis patients at participating centres were invited to donate tissue and blood as part as continous bio-banking efforts. Samples used in this study were choosen according to the inclusion criteria outlined in the material and method sections. For the Toulouse Cohort, Inclusion criteria were presence of brain metastases, surgery of at least one brain metastasis, histological diagnosis of lung cancer (all histological subtypes were included) and administration of WBRT within 2.5 months post-surgery as well as follow-up MRI.<br>For cohorts from Dublin, Manchester and Turin, patients with histological diagnosis of brain metastasis that received WBRT and had follow-up OS data were included.<br>For samples used in the liquid biopsy study, Inclusion criteria were presence of brain metastasis, confirmed administration of WBRT, annotated clinical history related to time of death from diagnosis of brain metastasis, collection of serum sample before or within 2.5 months of radiotherapy. In order to evaluate definite survival, only patients that had passed away were included.
Ethics oversight	All samples were in compliance with protocols approved by their respective Institutional Review Board (IRB) and/ or national laws (4270-CEI22/20, Hospital de La Princesa; CEI PI 64_2016-v3 and CEI PI 25_2020-v2, Hospital 12 de Octubre-CNIO; Institutional Review Board of Department of Neuroscience, University of Turin; 8/NW/0092 and 13_RIMA_01, Manchester Cancer Research Centre (MCRC) Biobank ethics application 18/NW/0092 with written informed consent from the patients at The Christie NHS Foundation Trust, the study was approved by MCRC Biobank Access Committee application 13_RIMA_01, 20/EE/0002 (Preston cohort), University of Pittsburgh IRB#PRO15050502, Royal College of Surgeons in Ireland IRB#13/09/ ICORG09/07 and the Mayo Clinic Cancer Center Institutional Review Board, PV4904/PV5392, University Medical Center Hamburg-Eppendorf; REC reference 13/09, RCSI Beumont Hospital; CEI PI 25_2020-v2, Clínical Universitaria de Navarra; KEK 2021-00652, University Hospital Zurich; 017/2021, Doctor Josep Trueta University Hospital).

Note that full information on the approval of the study protocol must also be provided in the manuscript.

Flow Cytometry

Plots

Confirm that:

☒ The axis labels state the marker and fluorochrome used (e.g. CD4-FITC).

☒ The axis scales are clearly visible. Include numbers along axes only for bottom left plot of group (a 'group' is an analysis of identical markers).

☒ All plots are contour plots with outliers or pseudocolor plots.

☒ A numerical value for number of cells or percentage (with statistics) is provided.

Methodology

Sample preparation	For flow cytometry staining and cell sorting of S100A9/CD55, Brains were digested in RPMI 2%FBS with Collagenase IV (C5138, Sigma-Aldrich) for 30 min at 37ºC. Red blood cells were lysed with ACK Lysing Buffer (10-548E, Lonza). Myelin was removed with Percoll 22%. Single-cell suspensions were resuspended in DPBS containing 2% FBS and 1mM EDTA and incubated with FC-Block (553141, BD Biosciences). For the intracellular staining of S100A9, cells were fixed and permeabilized (BD Fixation/Permeablization Kit, 554714) and stained with primary-conjugated anti-MRP14 antibody (1:200; 350705, Biolegend).<br>For scRNA-Seq of sorted H2030-BrM cells from in vivo brain metastasis, mice with intracardiacally injected H2030-BrM cells at endpoint (5 weeks) were sacrificed and brains were extracted in pre-cooled D-PBS 1x. Established metastatic lesions were dissected and processed with the Brain Tumor Dissociation Kit (130-095-942, Miltenyi) using gentleMACS C Tubes (130-093-237, Miltenyi) and the gentleMACS™ Octo Dissociator (130-096-427, Miltenyi). Mice brains were washed in cold D-PBS, cut into 8 sagital slices and transferred into a gentleMACS C Tube, with the enzymatic mixed provided by the kit, to be digested in the gentleMACS Octo Dissociator with Heaters (gentleMACS Program 37C_BTDK_01). Cell suspension was filtered with a 70 µm strainer and centrifuged at 300 x g for 10 minutes at 4 °C. For myelin removal, debris removal solution included in the Adult Brain Dissociation Kit (130-107-677, Miltenyi) was applied. The supernatant was discarded and the cells ready for FACS were diluted in cold D-PBS/BSA buffer 0.04%.
Instrument	CD55+ and CD55- cells were isolated using BD Influx cell sorter. For the intracellular staining of S100A9, samples were acquired in a LSR Fortessa (BD Bioscience).<br>For sorting of GFP+ cells for scRNA-Seq, cells of interest were isolated according to GFP expression using the BD Influx™ cell sorter.
Software	FlowJo software v10 (Flowjo LLC)
Cell population abundance	More than 20,000 GFP+ CD55+ /GFP+ CD55- events were sorted for the oncosphere formation assay.<br>More than 50,000 GFP+ events were acquired to analyze the colocalization between CD55 and S100A9 in GFP+ H2030-BrM cells.
Gating strategy	Pulse processing and DAPI were used to exclude cell aggregates and dead cells and Fluorescence minus one controls were used to perform the gating of the different subpopulations analyzed.<br>For the isolation of GFP+ H2030-BrM cells, the live single population, excluding debris, was gated to select GFP+ events. The GFP+ population was gated to sort CD55+ and CD55- cells.

☒ Tick this box to confirm that a figure exemplifying the gating strategy is provided in the Supplementary Information.
